# P3H4 Overexpression Serves as a Prognostic Factor in Lung Adenocarcinoma

**DOI:** 10.1155/2021/9971353

**Published:** 2021-06-23

**Authors:** Xiangfeng Jin, Haiqing Zhou, Jianfang Song, Hong Cui, Yiren Luo, Haiping Jiang

**Affiliations:** ^1^Department of Thoracic Surgery, The Affiliated Hospital of Qingdao University, Qingdao, Shandong Province 266003, China; ^2^Department of Anesthesiology, The Affiliated Hospital of Qingdao University, Qingdao, Shandong Province 266003, China; ^3^Operation Room, The Affiliated Hospital of Qingdao University, Qingdao, Shandong Province 266003, China; ^4^Department of Oncology, The Affiliated Hospital of Qingdao University, Qingdao, Shandong Province 266003, China

## Abstract

**Background:**

The present study is aimed at evaluating the functional and clinical values of P3H4 in lung adenocarcinoma. Moreover, we also investigated the downstream pathways that P3H4 might participate in.

**Methods:**

The differential expression analysis was used to identify genes differentially expressed in lung adenocarcinoma tissues as compared with normal tissues. Survival analysis was used to test the association between P3H4 and survival time. Gene set enrichment analysis was conducted to explore the downstream pathways. CCK8 and transwell were employed to examine the impact of P3H4 on cell phenotypes.

**Results:**

P3H4 was highly upregulated in LUAD tissues at both RNA and protein levels. Moreover, the LUAD patients, who had high expression of P3H4, were also observed to have shorter disease-free survival and overall survival. These results demonstrated that P3H4 could be used as a prognostic biomarker for LUAD. Moreover, we also found that it was the copy number alterations (CNAs), not DNA methylation, that regulated the RNA expression of P3H4, indicating that its upregulation might be partially resulted from the CNAs. Furthermore, functional experiments revealed that the A549 and H1299 cells with siRNA treatment (siP3H4) exhibited significantly decreased cell proliferation after 24 hours, migratory ability, and invasiveness. Functionally, the upregulated proteins in the P3H4 high expression group were mainly enriched in tumor microenvironment-related pathways such as phagosome, focal adhesion, and ECM-receptor interaction and cancer-related pathways such as bladder cancer pathway, proteoglycans in cancer, and hippo signaling pathway.

**Conclusion:**

The present study systematically evaluated the functional and clinical values of P3H4 in LUAD, and explored the related biological pathways. P3H4 might promote LUAD progression through regulating tumor microenvironment-related pathways.

## 1. Introduction

Lung adenocarcinoma (LUAD) is a major type of non-small-cell lung cancers (NSCLC) [1]. Developed in the epithelial cells of the lung, it is also the most common type of lung cancer, which accounts for nearly 40% of all lung cancer cases [2]. Compared to the other subtypes of lung cancers, lung adenocarcinoma is less aggressive, and favorable prognoses are observed in patients with small, localized adenocarcinoma (stage I) [3]. However, most patients with LUAD are diagnosed at advanced stages, where metastasis has occurred, which leads to a disheartening survival rate [4, 5].

Tumor microenvironment (TME) and the organization of local extracellular matrix (ECM) are found to be essential players in tumor progression and metastasis in various cancers [6–8]. The crosstalk between tumor cells and the immune cells modulates multiple aspects of tumorigenesis, and targeting important pathways in TME is considered a promising therapeutic strategy [9]. Also, tumor-associated macrophages (TAMs), which are an abundant cell population in the TME, could regulate the expression of immunosuppressive molecules such as PD-L1 and phagocytosis inhibitors, thereby promoting tumor progression and resistance to therapy [10].

Prolyl 3-hydroxylation (P3H) is a rare but conserved posttranslational modification in many collagen types [11] and may be implicated in a tumor microenvironment [12]. Specifically, two members of P3H genes, P3H2 and P3H3, are identified as novel targets for epigenetic silencing in breast cancer [13]. In bladder cancer, knockdown of P3H4 would result in arrested cell cycle and decreased expression levels of EMT-related proteins, suggesting that silence of P3H4 could efficiently inhibit the uncontrolled proliferation and invasiveness of bladder cancer [14]. Also, an association between increased P3H4 expressions and the high pathological stage and worse survival has been observed in bladder cancer [15]. Moreover, P3H4 is inferred from sequence similarity to be in a complex essential for cross-linking of collagen fibrils, and collagen cross-linking is reported to increase tumor cell proliferation and promote metastasis [16, 17]. Herein, we conducted a systematic analysis of P3H4 in tumor tissues at both RNA and protein levels and demonstrated its impact on cancer cell functionalities, anticipating to shed light on the potential function and mechanism of P3H4 in LUAD.

## 2. Materials and Methods

### 2.1. Gene Expression Data Collection

The gene expression dataset was obtained from public databases including UCSC Xena (https://xena.ucsc.edu/) with accession numbers: TCGA-LUAD [18]. The Fragment Per Kilo Million- (FPKM-) based gene expression was calculated for RNA sequencing data, respectively. In brief, the reads were aligned to human reference genome by STAR v2 [19], and gene expression levels were measured by HTSeq [20]. The protein expression data of LUAD and adjacent normal tissues were collected from LinkedOmics (http://linkedomics.org).

### 2.2. Differential Expression Analysis

The differential gene expression levels were tested by Student's *t-*test and fold change method. The *P* values by Student's *t*-test was adjusted by the Bonferroni method. The genes, which had adjusted *P* value < 0.05 and fold change > 2, were identified as dysregulated genes.

### 2.3. Survival Analysis

The univariable Cox regression model was built to evaluate the association between P3H4 expression levels and LUAD survival time. The samples were stratified into the high and low P3H4 expression groups using the median expression of P3H4 as the cutoff. The statistical significance of the association of P3H4 with survival time was evaluated by a log-rank test. The survival analysis was implemented in R survival package [21].

### 2.4. The Gene Set Analysis

The overrepresentation enrichment analysis (ORA) was used to identify the Reactome pathways enriched by the upregulated genes [22]. The enrichment degree of the ribosomal proteins in the genes highly correlated with P3H4 was tested by gene set enrichment analysis (GSEA). The ORA was implemented in R clusterProfiler package [23].

### 2.5. Cell Culture and Transfection

The two human lung cancer cell lines (A549 and H1299) were purchased from Shanghai Institute of Materia Medica, Chinese Academy of Sciences (CAS). These cells were cultured in RPMI-1640 medium supplemented with 10% fetal bovine serum (Gibco) and 1% penicillin-streptomycin and incubated at 37°C with 5% CO_2_. The two small-interface RNAs specifically binding P3H4 mRNA and the negative control were denoted as si-P3H4-1, si-P3H4-2, and si-NC. The transfection was conducted on the cells in logarithmic phase using Lipofectamine 2000 (Thermo Fisher Scientific). The following are the sequences of siRNAs: si-P3H4-1—GGGCUGUGAAGCUCUACAACA; si-P3H4-2—GGCACGCUCUGGAGCAGUACG.

### 2.6. Real-Time Reverse Transcription PCR

Total RNAs from A549 and H1299 cells were separated using a TRizol reagent. Following the manufacturer's protocol, we performed the reverse transcription using PrimeScript RT reagent kit (TaKaRa, Tokyo, Japan). P3H4 mRNA expression was quantitatively analyzed using an ABI Prism 7900HT (Applied Biosystems, Foster City, CA), with GAPDH as an internal reference. The following are the primers for P3H4: forward—5′-CATGAGCAGGTGGACTTCAAGG-3′, reverse—5′-ACTTGTCCACGAAGTAGCCACC-3′; and the primers for GAPDH: forward—5′-AGGCTGTTGGGAAAGTTCTTC-3′, reverse—5′-ACTGTTGGAACTCGGAATGC-3′. All these experiments were conducted in triplicate.

### 2.7. Cell Counting Kit-8 (CCK8) Assay

The CCK8 assays were used to detect the cell proliferation. The experiments were conducted in 96-well plates with 2 × 10^3^ cells/well. Using a microplate reader (Bio-Rad, Shanghai, China), we detected the absorbance at 450 nm following the manual. All these experiments were conducted in triplicate.

### 2.8. Transwell Assay

The transwell chambers (8 *μ*m pore size; Millipore) and chambers coated with Matrigel were used to conduct cell migration and invasion assays, respectively. The cells (5 × 10^4^ cells) with 48 h of transfection were planted into the upper chamber, and 500 *μ*L of medium containing 10% FBS was filled into the lower chambers. The migrated or invaded cells with 4% paraformaldehyde, which were incubated at 37°C for 24 h, were fixed for 30 min and strained with 0.1% crystal violet for another 20 min. All these experiments were conducted in triplicate.

### 2.9. Statistical Analyses

The multiple-sample and two-sample comparisons were tested by the analysis of variance (ANOVA) and Student's *t*-test in R language. The data are visualized as the mean value and 95% confidence interval. The hypothesis tests, which had *P* value < 0.05, were considered statistically significant.

## 3. Results

### 3.1. The mRNA Expression of P3H4 Is Highly Upregulated in Lung Adenocarcinoma Tissues

**Figure 1:**
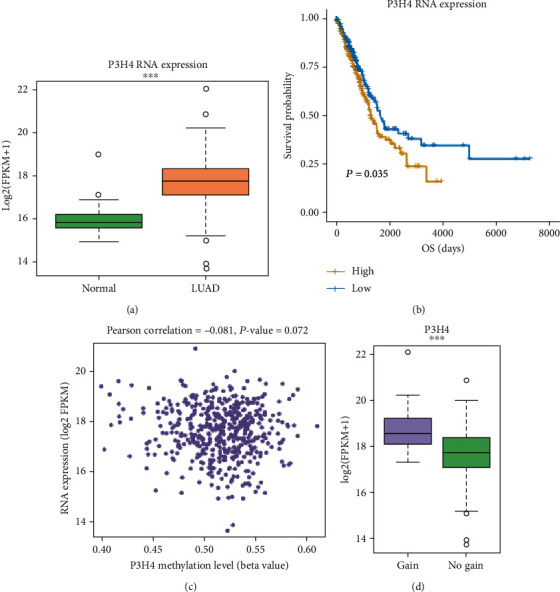
The expression pattern of P3H4 in lung adenocarcinoma (LUAD). (a) The RNA expression levels of P3H4 in LUAD and normal tissues of TCGA cohort. The orange and green colors represent the LUAD and normal samples. (b) The Kaplan Meier curves for the LUAD samples with high (yellow) and low (blue) P3H4 expression. (c) The correlation between P3H4 methylation levels and RNA expression levels. (d) The differential expression levels of P3H4 in LUAD with and without P3H4 gains.

To investigate the RNA expression levels of P3H4 in lung adenocarcinoma (LUAD) and normal tissues, we compared its expression of LUAD with that of normal tissues using The Cancer Genome Atlas (TCGA) LUAD cohort. Specifically, P3H4 RNA expression was highly upregulated in LUAD (Figure 1(a), Wilcoxon rank sum test, *P* value < 0.001), with about 2-fold than the normal tissues. The survival analysis revealed that shorter overall survival was observed in samples with high mRNA expression of P3H4 as compared with those with low mRNA expression (Figure 1(b), *P* value < 0.05).

To further explore the transcription of P3H4, we investigated whether the copy number alteration (CNAs) and DNA methylation levels were associated with its RNA expression. The Pearson correlation between the DNA methylation and RNA expression of P3H4 was about -0.081, suggesting that P3H4 mRNA expression was poorly associated with DNA methylation (Figure 1(c), *P* value = 0.072). Moreover, the comparison of P3H4 RNA expression between the tumor samples with P3H4 gain and those without P3H4 gain revealed that P3H4 was expressed higher in those with P3H4 gain (Figure 1(d), *P* value < 0.001). These results indicated that P3H4 was highly upregulated in LUAD tissues and its upregulation might be partially resulted from the CNAs.

### 3.2. Validation of High P3H4 Expression in LUAD at Protein Level

**Figure 2:**
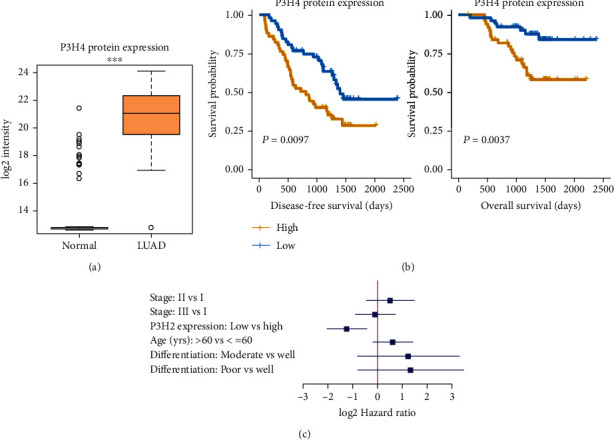
The protein expression patterns of P3H4 in LUAD and normal tissues. (a) The differential expression levels of P3H4 protein between LUAD and normal tissues of Xu et al. cohort. (b) The association of P3H4 protein with disease-free survival (DFS) and overall survival (OS). (c) The independence of P3H4 expression about the LUAD risk prediction. The forest plot indicates the hazard ratios and 95% confidence intervals for the prognostic factors in the multivariable Cox model.

As P3H4 was highly upregulated in LUAD at the RNA level, we then examined its protein expression using mass spectrum proteomics data from Xu et al. [24]. Consistently, the protein expression of P3H4 was also upregulated in LUAD as compared with the normal tissues (Figure 2(a), *P* value < 0.001). The further survival analysis of the P3H4 protein expression confirmed that P3H4 protein expression was also highly associated with both disease-free survival (DFS) and overall survival (OS) (Figure 2(b), log-rank test, *P* value < 0.05), suggesting that P3H4 protein expression was an indicator of poor prognosis in LUAD. To test the independence of P3H4 protein expression about the prediction of survival in LUAD, we conducted multivariable Cox regression analysis using TNM stage, age, and differentiation levels as cofactors. Remarkably, the P3H4 protein expression was also statistically significant in the multivariable Cox model (Figure 2(c)), suggesting that P3H4 protein expression was an independent prognostic factor in LUAD. These results further indicated that P3H4 was highly upregulated and might serve as an independent prognostic factor in LUAD.

### 3.3. Silence of P3H4 Inhibits Lung Cancer Cell Proliferation

**Figure 3:**
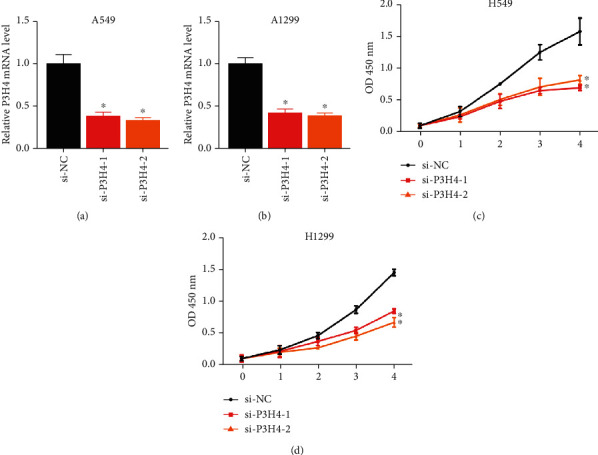
The impact of P3H4 silence on the A549 and H1299 cell proliferation. The RNA expression of P3H4 in the A549 (a) and H1299 (b) cells with and without siRNAs knockdown was measured by qPCR. The cell proliferation levels of A549 (c) and H1299 (d) with siP3H4-1, siP3H4-2, and siNC (negative control) treatments. The red, orange, and black bars represent the siP3H4-1, siP3H4-2, and siNC groups, respectively.

To explore whether P3H4 regulated the functionalities of lung cancer cells, we silenced the P3H4 gene by two small interface RNAs (siRNAs) using A549 and H1299 cell lines. Specifically, the RNA expression levels of P3H4 were significantly suppressed by the siRNAs (Figures 3(a) and 3(b), *P* value < 0.05), indicating that this siRNA could efficiently decrease the P3H4 RNA expression. Compared with the negative control (siNC), the two cell lines with siRNAs treatment (si-P3H4-1 and si-P3H4-2) exhibited significantly decreased cell proliferation (Figures 3(c) and 3(d), *P* value < 0.05), suggesting that P3H4 gene silence could suppress the uncontrolled proliferation of A549 and H1299 cells.

### 3.4. Silence of P3H4 Inhibits Lung Cancer Cell Invasion and Migration

**Figure 4:**
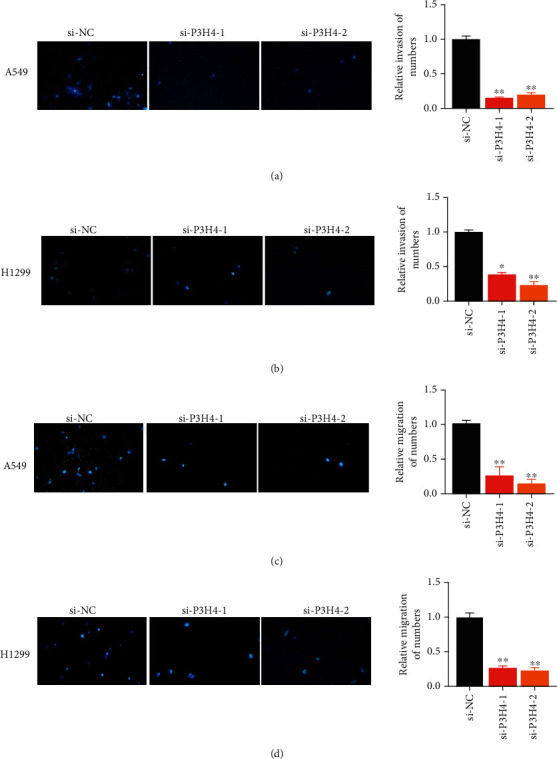
The impact of P3H4 silence on migratory and invasive abilities of A549 and H1299 cells. The cell invasion (a, b) and migration (c, d) of the siNC, siP3H4-1, and siP3H4-2 groups are were detected and compared by transwell assays. The relative number of invaded and migratory cells is visualized as a bar plot on the right panel.

Furthermore, we also detected the impact of P3H4 silence on cancer cell migration and invasion. Specifically, transwell assay revealed that knockdown of P3H4 could significantly suppress the invasiveness of A549 and H1299 cell lines (Figures 4(a) and 4(b)). Furthermore, the decreased migratory ability of A549 and H1299 with P3H4 silence was also observed by the transwell assay (Figures 4(c) and 4(d)). These results indicated that P3H4 silence could significantly inhibit lung cancer cell invasion and migration.

### 3.5. P3H4-Related Signaling Pathways

**Figure 5:**
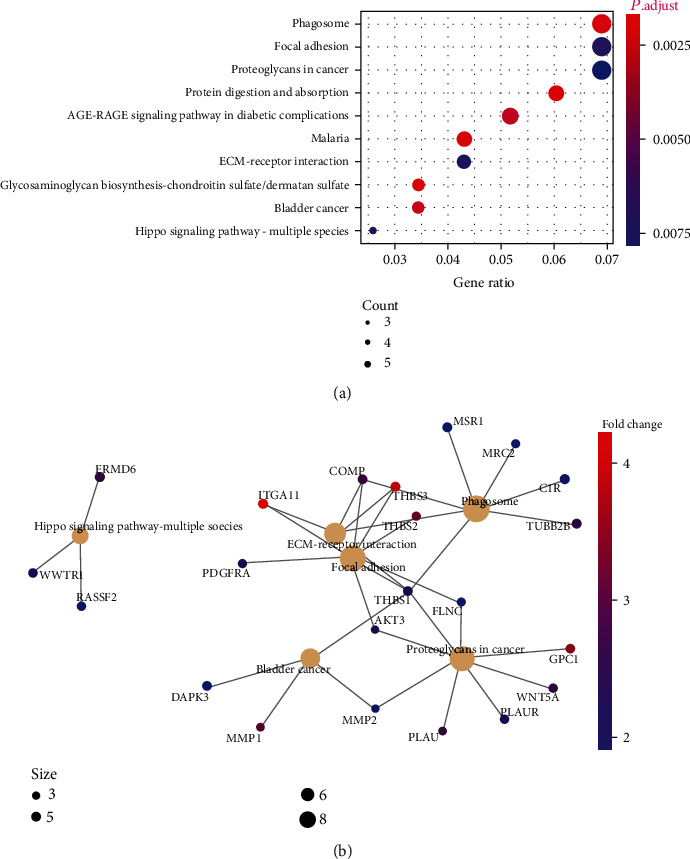
The biological function of P3H4 predicted by gene set enrichment analysis. (a) The pathways enriched by the upregulated genes in tumor samples with high P3H4 expression. The node size and color represent the number of upregulated genes within the pathway and the *P* value for the enrichment. (b) The key genes involved in the pathways are displayed in a network, which was constructed by the gene-pathway relationship. The node size and color represent the gene number and log2 fold change.

To further explore the biological function of P3H4 protein in LUAD, the tumor samples of the proteome cohort were stratified into the high and low P3H4 expression groups. The differential expression analysis identified 305 upregulated and 11 downregulated proteins (Supplementary Table S1, adjusted *P* value < 0.05 and fold change > 2). Notably, only the upregulated proteins were successfully enriched in the KEGG pathways by gene set enrichment analysis, as the number of downregulated proteins was small. Specifically, the upregulated proteins in the high P3H4 protein expression group were mainly enriched in the pathways regulating the tumor microenvironment such as phagosome, focal adhesion, and ECM-receptor interaction and cancer-related pathways such as bladder cancer pathway, proteoglycans in cancer, and hippo signaling pathway (Figure 5(a)). The network visualization of the pathways and related genes showed that FLNC, PLAU, WNT5A, THBS1, MSR1, FRMD6, PLAUR, MMP2, DAPK3, AKT3, THBS2, TUBB2B, GPC1, MMP1, WWTR1, PDGFRA, RASSF2, THBS3, C1R, MRC2, COMP, and ITGA11 were the hub genes involved in these cancer or tumor microenvironment-related pathways (Figure 5(b)). These results indicated that P3H4 might promote LUAD progression through regulating cancer and tumor microenvironment-related pathways.

## 4. Discussion

In this study, in order to investigate the expression pattern and clinical values of P3H4 in lung adenocarcinoma, we conducted differential expression analysis and survival analysis on both TCGA and proteome cohort. Specifically, P3H4 was highly upregulated in LUAD tissues at both RNA and protein levels. Moreover, the LUAD patients, who had high expression of P3H4, were also observed to have shorter disease-free survival and overall survival. These results demonstrated that P3H4 could be used as a prognostic biomarker for LUAD. Notably, P3H4 was also observed to be closely associated with the prognosis of bladder cancer [15, 25]. As the cancer was initiated by genomic and epigenomic alterations like CNAs and abnormal DNA methylation [26, 27], we investigated the regulation of P3H4 by genomic and epigenomic alterations. The comparative analysis and correlation analysis revealed that it was the CNAs, not DNA methylation, that regulated the RNA expression of P3H4, indicating that its upregulation might be partially resulted from the CNAs. As the DNA is more stable with respect to RNA, the relative copy numbers of P3H4 might be used to evaluate P3H4 expression levels and patients' risk using tumor tissues or circulating tumor cells (CTC) [28].

As P3H4 was highly expressed in LUAD, we then explored whether the functionalities of lung cancer cells could be significantly altered by silencing P3H4. Specifically, compared with the negative control (siNC), the cells with siRNA treatment (siP3H4) exhibited significantly decreased cell proliferation, migratory ability, and invasiveness using A549 and H1299 cell lines. Consistently, the inhibition of proliferation and invasion by the knockdown of P3H4 were also observed in bladder cancer [14].

Functionally, we also explored the biological function of P3H4 protein in LUAD. The upregulated proteins in the P3H4 high expression group were mainly enriched in tumor microenvironment-related pathways such as phagosome, focal adhesion, and ECM-receptor interaction and cancer-related pathways such as bladder cancer pathway, proteoglycans in cancer, and hippo signaling pathway. Notably, the molecules localizing to the extracellular matrix such as ITGA11, PDGFRA, THBS1/2/3, and COMP were coexpressed with P3H4 and might be the direct target of P3H4. ITGA11 was found to enhance tumorigenicity of human non-small-cell lung cancer cells by regulating IGF2 expression in fibroblasts [14]. PDGFRA is a famous growth factor receptor and has been widely reported to transfer signaling in various cancers [29, 30]. The thrombospondins (TSPs) are multifaced proteins and serve as important components of the tumor microenvironment [31], indicating that P3H4 might be a regulator of tumor microenvironment. Consistently, previous studies also found that P3H4 was involved in regulating the tumor microenvironment and implicated in sensitivity to targeted therapy and immunotherapy [12, 32].

In conclusion, the present study systematically evaluated the expression levels and clinical values of P3H4 in LUAD and explored the related biological pathways. P3H4 might promote LUAD progression through regulating tumor microenvironment-related pathways.

## Data Availability

The datasets used and/or analyzed during the current study are available from public databases including TCGA and GEO, which have been cited as references in Materials and Methods.

## Supplementary Materials

Supplementary Materials Supplementary Table S1: the differentially expressed genes between tumor samples with high and low P3H4 expression.
