# Supplementary material for: P3H4 Overexpression Serves as a Prognostic Factor in Lung Adenocarcinoma
Source: Comput Math Methods Med. 2021 Jun 23;2021:9971353. doi: 10.1155/2021/9971353 (PMC8249155; doi:10.1155/2021/9971353)
Supplement: Supplementary Materials — Supplementary Table S1: the differentially expressed genes between tumor samples with high and low P3H4 expression. [file 9971353.f1.pdf]

gene	pvalue	log2FoldCl	padj
IGLV4-69	0.299797	1.083581	0.602797
IGLV8-61	0.970476	-0.41099	0.997592
IGLV4-60	0.546526	0.47421	0.778915
IGLV10-54	0.421971	0.556096	0.691467
IGLV7-46	0.893443	-0.1612	0.967693
IGLV5-37;IGLV5-52	0.291769	0.482756	0.595183
IGLV2-18	0.500085	0.747559	0.74591
IGLV3-12	0.594656	-0.13647	0.806337
IGLV3-10	0.336136	-0.99725	0.623617
IGLV3-9	0.35064	0.05375	0.635657
IGKV2D-28	0.425261	0.664893	0.693599
IGHV3-64	0.807504	-0.22214	0.921159
IGHV4-4	0.777334	-0.34037	0.906372
IGKV2D-29;IGKV2D-26	0.175647	-1.27344	0.46717
IGKV1-27	0.394718	0.587275	0.672349
IGLV5-45	0.09703	1.424561	0.354489
IGKV3D-15	0.07985	1.6346	0.325919
IGKV1D-8	0.976419	0.027518	0.998921
TMEM265	0.661071	0.241823	0.844596
IGKV2-40	0.405867	1.14474	0.680084
MGC50722	0.993518	-0.02343	1
IGHV1-45	0.125999	1.168751	0.403148
IGHV3-49	0.42656	0.307003	0.693599
IGKV6D-21	0.39952	0.818149	0.67622
IGLV1-36	1	-0.13283	1
IGHV6-1	0.205339	0.745804	0.49957
IGHV3-15	0.674379	-0.42597	0.853175
IGHV2-26	0.549247	0.608279	0.781327
IGHV3-73	0.25401	0.93626	0.557593
IGHV3-74	0.488796	0.522654	0.738656
IGHV3-43	0.550402	-0.48894	0.781327
IGLV9-49	0.933301	-0.1332	0.984157
IGHV3-72	0.34427	-0.95649	0.630561
PIGBOS1	0.078866	-2.66723	0.32578
IGHV1-69-2	0.310183	0.352186	0.609864
IGKV6-21	0.421207	-0.34783	0.691066
IGKV3D-20	0.525205	0.821965	0.764479
IGHV1-3	0.197877	1.155886	0.491413
IGHV1-18	0.268217	1.142603	0.570868
IGHV3-20	0.508688	0.21031	0.752448
IGHV1-24	0.310396	0.797754	0.609894
IGHV4-28	0.36079	1.322977	0.644411
IGHV5-51	0.57727	-0.47729	0.796634
IGHV1-58	0.626197	0.252646	0.828579
IGHV2-70D	0.798306	-0.17902	0.916091
IGKV1-8;IGKV1-9	0.282704	0.722061	0.585849
IGKV2-24	0.794066	-0.09637	0.913708
IGKV1-6	0.887219	-0.10956	0.96416
IGHV1-69-2	0.556057	0.179569	0.783439
IGLV5-39	0.693787	0.285096	0.861478
IGHV7-4-1	0.819598	-0.0726	0.92687
IGHV3-64D	0.121951	1.393081	0.397343
PNMA6E	0.041397	0.564903	0.240826
IGHV5-10-1	0.094648	-1.50382	0.350381
MMP24OS	0.170868	-1.37417	0.461822
CSNKA2IP	0.128633	-0.54511	0.408202
PRRT1B	0.988991	0.02042	1

SLC12A8	0.253685	0.236165	0.557407
RBM47	0.226131	-0.21121	0.525131
TTC26	0.752138	0.138952	0.893334
TMEM129	0.841169	-0.10194	0.939802
UBA6	0.019715	0.406901	0.1684
ESYT2	0.09193	0.212712	0.345626
ESYT3	0.344404	-0.23079	0.630639
MED19	0.148163	1.181937	0.437508
UHRF1BP1L	0.086585	0.987944	0.337307
IGLC7	0.0483	-1.72718	0.258042
KIAA1598	0.709387	0.089624	0.869179
TMEM223	0.116113	0.936421	0.38883
GXYLT2	0.001668	1.83223	0.042235
TMEM120B	0.69753	-0.31481	0.863813
ARHGAP10	0.709384	0.090154	0.869179
ANO9	0.684743	0.157059	0.85709
SLC22A23	0.298494	0.34649	0.601676
CCDC64B	0.493883	-0.45517	0.741997
ARHGEF37	0.713801	0.135015	0.87164
MEX3A	0.511124	0.286912	0.754376
ILVBL	0.43056	0.132778	0.695651
TSPAN11	0.96897	-0.00285	0.997592
C1orf226	0.465112	0.605008	0.721065
C17orf89	0.635921	-0.24693	0.833511
PLEKHG3	0.512478	0.297214	0.755334
ELOVL7	0.079566	-1.07624	0.325919
SSC5D	0.306214	0.259027	0.607661
SH3PXD2B	6.45E-09	1.844608	3.40E-06
FAM92A1	0.060633	0.787108	0.287262
MACROD2	0.980555	-0.11765	1
ZC3H12D	0.38755	0.437779	0.666378
FRMD3		0	
CXorf23	0.529495	0.20692	0.768066
IGKV A18	0.051749	0.822481	0.266457
NBAS	0.528742	0.07462	0.767576
TARSL2	0.502916	-0.24555	0.747596
SMCO3	0.452579	0.362391	0.712397
KIAA1467	0.869166	0.080666	0.955488
TYW5	0.103724	0.745939	0.366863
DENND3	0.934082	0.077617	0.984157
FUOM	0.494892	-0.31997	0.742037
KIAA0922	0.208518	0.514418	0.503054
VWA8	0.641914	-0.14472	0.835764
SBNO1	0.769114	-0.17403	0.901915
ISPD	0.538387	-0.27776	0.774281
FAM221A	0.380925	-0.49771	0.661698
GSAP	0.608713	0.018681	0.816529
GTPBP10	0.845683	0.114184	0.942239
WDR91	0.794443	0.029339	0.913708
KLRG2	0.648995	0.173108	0.839222
RAB19	0.648077	-0.53159	0.838523
LCHN	0.908605	-0.10424	0.973845
MBLAC1	0.74145	0.069305	0.887483
SH3D21	0.881851	0.056157	0.961515
EFCAB5	0.501113	0.400806	0.74652
VPS37C	0.094452	0.526378	0.349959
CCDC151	0.724989	-0.19523	0.877248
TMEM189	0.000862	2.516248	0.027972

TRAPPC13	0.845694	-0.20219	0.942239
CNOT1	0.000641	0.370828	0.023555
ARHGEF35	0.777937	-0.14277	0.906856
MEGF11	1	-0.02585	1
CCDC88B	0.083229	1.075928	0.332962
KRT87P		0	
FAM83G	0.030715	0.927185	0.211475
ASPDH	0.974393	-0.0948	0.998921
PALM3	0.685856	-0.05478	0.857881
PGP	0.739056	0.255847	0.886253
C5orf51	0.179497	0.319647	0.471072
RCCD1	0.913914	0.330536	0.97604
PLEKHD1	0.377974	-0.68189	0.659304
SOWAHB	0.942624	0.070702	0.988547
ZNF316	0.276259	0.575233	0.579642
TCAF2	0.737731	0.18278	0.886077
MFSD2B	0.331588	-0.11214	0.619341
CCDC160	0.393617	-0.37482	0.671013
GLTPD2	0.657906	-0.19469	0.842898
CAPN8	0.952829	0.040122	0.993074
TUBAL3	0.018367	0.31847	0.163766
FBLL1	0.670122	0.310322	0.850681
SMCHD1	0.121915	0.237706	0.397343
GATSL2	0.190786	0.523382	0.484103
ARHGAP42	0.324802	0.402809	0.61537
NCF1B	0.725845	-0.18409	0.877996
CCDC69	0.90576	0.042372	0.973334
UNC119B	0.724083	0.422958	0.876654
WTIP	0.980555	0.011016	1
METTL15	0.629237	0.156301	0.830125
SOWAHD	0.40269	-0.27213	0.678038
C2orf70		0	
IRG1	0.683728	-0.05326	0.85709
GLOD5	0.000637	-1.98553	0.023555
LIPT2	0.33402	-0.76994	0.620956
RASSF10	0.714696	0.265922	0.871878
CCDC85C	0.511224	0.323585	0.754376
PCP4L1	0.584108	-0.79792	0.80019
RGPD3	0.553712	-0.21315	0.783439
TTC36	0.163438	-0.25217	0.453012
COL6A6	0.014932	-1.44975	0.150049
BTBD11	0.796103	-0.14917	0.914603
ANKRD36	0.352362	0.346547	0.637424
ZSWIM8	0.703285	0.117349	0.866874
ARHGAP32	0.752808	-0.20576	0.893843
TBKBP1	0.021806	0.38819	0.176756
POM121C	0.011828	1.555673	0.13136
A2ML1	0.155961	0.575745	0.44616
CLCA1	0.49063	0.06203	0.738656
YDJC	0.61918	-0.36968	0.822829
LYRM9	0.093078	-1.03109	0.347256
STRA13	0.44489	0.389773	0.706713
FAM166B	0.08528	-0.43645	0.335537
HIDE1	0.674267	-0.23064	0.853175
NCF1C	0.563678	-0.13157	0.789479
PHF20L1	0.201881	0.645316	0.495039
KIAA1324L	0.825831	0.153803	0.930917
NUDT19	0.474191	0.297501	0.727018

ODF3B	0.310183	-0.37232	0.609864
COL6A5	0.037638	-1.37307	0.232686
MIF4GD	0.241663	0.262379	0.544139
FER1L4	0.511124	-0.35218	0.754376
MYO1G	0.392997	0.10373	0.671013
HACD1		0	
ESPN	0.029786	1.655951	0.208193
MYO9A	0.447716	0.245863	0.708118
SMIM1	0.031257	-1.77237	0.212999
RABGAP1L	0.747677	0.094777	0.890803
PPP1R3G	0.174631	0.698012	0.466796
IGLL5;IGLC1	0.609213	-0.33712	0.816529
RASA4B	0.78983	0.147975	0.911741
UMAD1	0.635414	0.332814	0.833511
TMEM238	0.615967	0.346296	0.821456
FAM195B	0.053649	0.497172	0.271145
INAFM1	0.638802	0.206865	0.834211
UPK3BL	0.041397	0.621785	0.240826
ANKRD65	0.632486	-0.15926	0.830965
NACA	0.085745	0.210865	0.335537
C11orf98	0.538099	-1.17252	0.773967
CNKSR3;IPCEF1	0.254274	0.425716	0.557593
STMND1	0.163438	-0.28282	0.453012
CROCC2	0.92066	-0.05654	0.98039
SMIM22	0.63742	-0.00858	0.833511
SLC35A4	0.490639	0.31332	0.738656
ASNSD1	0.946701	-0.18098	0.989712
SMCR7L	0.029813	2.082825	0.208196
DNASE2	0.286754	-0.26361	0.590053
AGPS	0.059675	0.311519	0.285693
UBXN8	0.005399	1.437916	0.086909
KIF2A	0.169043	0.184545	0.459676
SGK1	0.992139	-0.02809	1
TK2	0.123514	0.50648	0.399387
DDX39A	0.05451	0.469	0.273313
PDLIM1	0.103963	0.174212	0.366863
ACOT7	0.006009	0.883941	0.090802
MYO1C	0.301936	0.054817	0.603275
MYO1F	0.226131	0.155311	0.525131
SNAP23	0.415291	0.185307	0.686028
HAX1	0.955289	-0.09958	0.993074
EYA2	0.137489	0.513329	0.422524
FXYD1	1	0.002518	1
AIP	0.494799	0.090756	0.741997
GTPBP1	0.161013	0.240511	0.453012
KCNK1	0.09103	1.315451	0.344815
LGALS9	0.161013	0.252198	0.453012
STXBP3	0.72417	0.069644	0.876654
MASP2	0.316309	-0.361	0.613139
AP4M1	0.246696	0.707905	0.549922
ARVCF	0.16691	-0.52363	0.457139
SMAP	0.151385	0.701961	0.44125
RAB27B	0.415291	-0.39124	0.686028
AP3B1	0.098469	0.175788	0.357719
SULT2B1	0.490199	0.585906	0.738656
TLR4	0.079293	0.229176	0.32578
RHOD	0.971656	0.043076	0.997592
APBB1	0.137878	0.747214	0.423394

LGALS8	0.223612	0.513676	0.521792
NDUFS8	0.981581	-0.0348	1
HAS3	0.003276	1.080963	0.065524
TNFRSF10A	0.208847	0.563548	0.503054
NFKBIE	0.579442	0.180989	0.797047
PSMD11	0.043207	0.232115	0.246126
PSMD12	0.019032	0.222464	0.166597
PSMD9	0.206534	0.22669	0.500256
SIRPB1	0.638802	0.109512	0.834211
ATOX1	0.882017	-0.31391	0.961515
MEN1	0.928648	-0.1763	0.983115
CBX4	0.022589	1.454494	0.180886
WRB	0.310963	0.754782	0.609894
PGRMC1	0.871617	0.070336	0.956098
SUPT5H	0.118765	0.173135	0.392409
TAF4	0.389244	0.505994	0.667643
DFFA	0.684992	-0.07326	0.85709
RFXAP	0.376926	0.55249	0.658447
HIP1	0.934288	0.15798	0.984157
LEFTY2	0.417872	-0.62743	0.688796
CLIC1	0.06524	0.268032	0.298136
EIF3F	0.112657	0.221183	0.382523
WWP2	0.943939	0.067893	0.988547
CDC7	0.204864	0.535873	0.499264
ARNTL	0.019021	1.117253	0.166597
PIK3CD	0.228518	0.618201	0.528484
PDHX	0.11568	-0.22719	0.387731
SULT1C2	0.920426	-0.02134	0.980269
MATN2	0.096798	-0.96879	0.354489
FOXE1	0.540968	0.263629	0.775521
QSOX1	0.061485	0.534186	0.289565
DCTN6	0.528737	-0.42876	0.767576
SLC33A1	0.072266	0.401882	0.313701
WASL	0.955289	0.057599	0.993074
PDE2A	0.179677	-0.41076	0.471223
IPO5	0.051278	0.297224	0.265139
POLRMT	0.020073	1.399748	0.169555
EEF2K	0.779586	0.227422	0.907186
CCRL2	0.163438	-0.18135	0.453012
SAP18	0.871617	-0.08005	0.956098
EML1	0.424783	-0.003	0.693599
IGF2BP3	0.023733	1.802582	0.186286
DNM1L	0.034511	0.245576	0.222148
RTCA	0.081043	0.286095	0.328405
PIK3C2A	0.537409	0.137302	0.773175
LST1	0.105925	-0.71421	0.370605
IFRD1	0.008601	0.884441	0.111593
PIK3R2	0.664258	0.433219	0.847289
GOLIM4	0.277896	0.191231	0.579677
MANBA	0.480311	0.534985	0.732554
TRAF5	0.004576	1.437211	0.078202
AGRN	0.000741	-0.60374	0.025686
PLOD2	1.71E-06	2.537964	0.000231
MEIS1	0.090516	1.164223	0.343901
EXOC5	0.330591	0.141771	0.619341
BTN3A3	0.67051	-0.20746	0.850681
HMGN4	0.742831	0.35373	0.888303
BTN3A1	0.081448	1.24913	0.329697

NDUFA4	0.164991	0.042817	0.454525
PSMD14	0.992106	-0.0101	1
ZNF593	0.329628	0.803	0.619341
BIN1	0.997369	0.117302	1
CLDN5	0.267929	-1.02516	0.570364
KPNA3	0.06524	0.331423	0.298136
STK25	0.601312	0.477928	0.811336
USP9Y	0.866049	-0.07064	0.953042
BCL9	0.942883	-0.13354	0.988547
LAD1	0.929021	0.252136	0.983115
FAAH	0.263524	-0.37623	0.565873
KRIT1	0.182493	0.766225	0.473679
CHL1	0.070292	-1.53975	0.309369
VWA5A	0.54177	-0.02415	0.775521
PES1	0.498977	0.178629	0.744839
EBAG9	0.413309	1.264444	0.685053
SDCBP	0.239034	0.185888	0.541047
PITPNM1	0.731541	-0.03295	0.881584
MPHOSPH10	0.029502	0.621023	0.207615
NOP56	0.1882	0.148586	0.480067
DDX3X	1.40E-06	0.499347	0.000194
RNASET2	0.656149	-0.12141	0.84209
CCL21	0.649073	0.193308	0.839222
MFNG	0.794772	0.087592	0.913708
PODXL	0.067185	-0.8624	0.302058
FCN1	0.219743	-0.93944	0.517665
CYR61	0.017624	1.71284	0.161008
PEX12	0.41034	0.634488	0.683141
PIR	0.277785	-0.39819	0.579677
PEX7	0.40774	-0.38464	0.680612
KPNA4	0.003027	0.401003	0.062294
TRIM38	0.597715	0.118621	0.808505
NFIB	0.611442	-0.29286	0.818417
PPP6C	0.118765	0.172172	0.392409
NME4	0.475968	-0.62094	0.728924
CES2	0.898276	-0.19772	0.970275
PIK3C2B	0.594251	0.147617	0.806337
MAN2B1	0.039909	0.267324	0.23845
FBP2	0.441947	-0.38718	0.703704
UBE2C	0.513979	0.507948	0.756193
PDXK	0.063339	0.207514	0.294055
SCD	0.628711	-0.54285	0.829728
DLGAP1	0.677587	-0.30014	0.854568
CLDN4	0.108082	-1.50518	0.374055
PPAP2A	0.443256	0.659036	0.704724
PPAP2B	0.887208	-0.13967	0.96416
ARID1A	0.840557	0.081615	0.939212
ISLR	2.08E-06	1.610698	0.000262
BHLHE40	0.109471	0.910134	0.377423
CDK2AP1;CDK2AP2	0.047127	1.774969	0.25572
SDHD	0.843152	0.31638	0.941448
C2CD2L	0.728536	-0.0561	0.879656
TMEM194A	0.532114	0.543552	0.770856
TXNDC9	0.30191	0.419336	0.603275
DPYSL4	0.077124	0.977536	0.324464
TRAFD1	0.222253	0.978929	0.520592
COX7A2L	0.751531	0.53317	0.893092
HSPB6	0.413404	-0.35616	0.685053

NDUFAB1	0.494799	-0.11764	0.741997
UBFD1	0.06182	0.897439	0.290811
CYB561D2	0.905487	-0.39178	0.973135
DYNC1I1	0.576148	0.365202	0.796634
CIT	0.586215	0.379986	0.801141
COPE	0.00344	0.386326	0.067417
RFXANK	0.58572	-0.38125	0.801141
CTDSP2	0.306086	0.31736	0.607626
VCY	0.331588	-0.16502	0.619341
EIF1AY	0.127585	-1.0867	0.406389
GNGT2	0.966966	-0.04903	0.996989
CDC42EP2	0.556057	0.111256	0.783439
AP3D1	0.000275	0.374601	0.013262
CCS	0.936911	-0.01703	0.986451
ENPP3	0.236409	0.942813	0.537858
ABLIM1	0.009173	-0.561	0.116274
DVL1;DVL1P1	0.001081	2.071299	0.031645
DVL2	0.017599	1.999796	0.160979
DNALI1	0.195154	-0.66889	0.489252
CHD1	0.345732	0.371371	0.632445
CHD2	0.699592	0.092832	0.864146
KCNK3		0	
GOSR2	0.280828	0.030787	0.583434
IRS4	0.349307	-0.13382	0.635421
TOR1A	0.047465	0.363561	0.255923
TOR1B	0.112657	0.404907	0.382523
STX16	0.096468	0.373338	0.354164
ADAM10	0.213733	0.230145	0.509228
ABCD4	0.617435	0.30846	0.822194
EI24	0.809658	0.250804	0.921586
ENC1	0.095134	0.499236	0.35136
TP53I11	0.503174	0.006494	0.747596
PTGES	0.693534	0.679435	0.861478
KMT2D	0.610081	0.123131	0.817298
ITGB1BP1	0.257067	0.665718	0.560462
RGPD8	0.062371	0.472351	0.292127
APAF1	0.060968	0.902405	0.288605
RIOK3	0.973565	-0.03215	0.998921
IMPA2	0.528892	-0.25848	0.767592
MAP2K7	0.700375	0.332366	0.864922
ACOT8	0.955286	0.39131	0.993074
CDIPT	0.161013	0.473349	0.453012
PDCD5	0.533067	0.113347	0.7709
PRMT5	0.627809	0.112779	0.828932
SLC9A3R1	0.135171	-0.20956	0.417833
HSD17B6	0.008178	-2.3139	0.108497
CHEK1	0.198062	0.654939	0.491653
TNFRSF10B	0.114869	0.990949	0.387168
FPGT	0.437458	0.575711	0.701933
TPP1	0.223612	0.163051	0.521792
GNB5	0.683128	0.144629	0.85709
TCERG1	0.204174	0.145909	0.497963
NDC80	0.028166	0.921309	0.203235
KIF3C	0.022384	0.783331	0.179899
NRP1	0.067188	0.353646	0.302058
TNPO2	0.382127	0.198881	0.661698
TNFSF11	0.988991	0.051303	1
APOL1	0.599446	-0.57064	0.810262

HS3ST1	0.547409	0.297229	0.779573
UNC13B	0.124833	-0.70849	0.401334
TNFRSF10C	0.366543	-0.54293	0.649206
POLR3A	0.27498	0.480541	0.577625
MRAS	0.187053	1.294285	0.479235
CAPN9	0.976419	0.008145	0.998921
TSPAN4	0.804328	0.15907	0.918848
PSMA7	0.882017	0.044267	0.961515
RASGRF2	0.008984	1.502477	0.11506
SCAMP3	0.856059	0.032814	0.946993
PHYH	0.509829	0.135935	0.753633
OPLAH	0.206534	-0.40071	0.500256
AIM2	0.241325	0.347304	0.544139
SLC30A4	0.992139	-0.09811	1
BACH1	0.005001	1.21767	0.082994
BCKDK	0.651389	0.093231	0.839222
IFIT3	0.72417	0.058473	0.876654
MGST3	0.117214	0.419577	0.389929
GEMIN2	0.004197	2.1639	0.073945
IRF6	0.126059	1.047349	0.403148
WNT9A	0.40269	-0.3351	0.678038
WNT9B	0.384669	0.389184	0.66413
TAX1BP3	0.039276	0.7199	0.236232
GIPC1	0.714303	0.035792	0.87164
LIN7A	0.881708	-0.12742	0.961515
PCDH17	0.375687	-0.28203	0.657392
IKBKB	0.008914	0.503859	0.114289
RGS12	0.325226	0.34065	0.615753
TIMM23;TIMM23B	0.146729	-0.03386	0.435709
HAT1	0.040551	0.756982	0.240216
UBE2L6	0.0799	0.374536	0.325919
CASK	0.32085	0.218186	0.613139
PLD2	0.623778	0.424236	0.826562
TFEC	0.300882	0.277926	0.602797
UQCRQ	0.044589	-0.57429	0.24851
MYL12B	0.000289	0.455626	0.013838
UQCR11	0.933998	-0.1139	0.984157
LECT2	0.906751	-0.02712	0.9734
HGS	0.177368	0.195124	0.468602
AURKA	0.023492	0.936937	0.184921
RAB29	0.616124	0.213766	0.821456
CLGN	0.461342	0.397742	0.718747
DSCR3	0.627809	0.044763	0.828932
PPP1R12A	0.001789	0.424369	0.044281
SLC27A2	0.700994	0.57489	0.865518
GAK	0.494799	0.103272	0.741997
HNRNPDL	0.159052	0.275941	0.449897
XPO1	0.274985	0.143303	0.577625
BTAF1	0.019371	0.738937	0.167192
ATP2A1	0.186982	-0.32846	0.479162
ARHGEF10	0.003581	0.70323	0.068752
ZNF609	0.234582	0.559607	0.536443
PDZD2	0.988072	0.067865	1
SPTBN2	0.482372	-0.30775	0.733586
MAST4	0.074743	0.560655	0.318513
SEC16A	0.093209	0.253039	0.347256
PLXNB2	0.357491	0.185415	0.641472
KHNYN	0.029995	1.288143	0.209097

TECPR2	0.090902	0.718277	0.344565
U2SURP	0.226131	0.218884	0.525131
SETD1A	0.036833	0.961097	0.229759
N4BP3	0.430232	-0.36577	0.695651
TRANK1	0.335919	0.413759	0.623545
SYNJ2	0.005023	1.42899	0.083138
SYNM	0.520142	-0.35801	0.760272
ZBTB5	0.011513	0.626085	0.129242
KIAA0355	0.028695	0.76711	0.204946
KIF3B	0.122702	0.60641	0.398971
PFAS	0.524435	0.098205	0.764023
MCF2L	0.352984	0.398863	0.638094
NACAD	0.499616	0.038897	0.745391
DCLK1	0.350635	0.217414	0.635657
CEP290	0.154165	0.718807	0.444958
ERC2	0.001508	1.688191	0.039821
ANKRD28	0.118765	0.490905	0.392409
ARHGEF11	0.000502	1.223555	0.020488
KIAA0391	0.446145	0.31667	0.706795
CHUK	0.156143	0.283997	0.446449
LSM1	0.000212	0.675082	0.011116
FYB	0.672844	-0.37493	0.85208
NPC1	0.665709	-0.00441	0.847351
TBX3	0.556057	0.101268	0.783439
AGPAT2	0.744027	-0.35854	0.888303
DEGS1	0.085745	0.392033	0.335537
ANGPT2	0.273609	0.618445	0.577625
SCAMP1	0.494799	0.142723	0.741997
SCAMP2	0.856059	0.079557	0.946993
KPNA5	0.319672	-0.32449	0.613139
ARPC1B	0.120331	0.186409	0.39497
ARPC2	0.045294	0.167192	0.250872
ARPC3	0.45805	0.082404	0.715224
BET1	0.046732	0.807781	0.254194
ZBTB7B	0.703791	-0.55645	0.867291
POLR1C	0.364426	0.092554	0.646996
PLSCR1	0.06524	0.471962	0.298136
TRIM24	0.145421	0.965503	0.434185
AXIN1	0.455572	0.218774	0.71417
PGRMC2	0.031791	0.250603	0.214212
CETN3	0.028045	1.690572	0.203111
CTDSPL	0.409977	0.367114	0.682809
VILL	0.922175	-0.17246	0.980751
ADAMDEC1	0.156241	0.975374	0.446614
UBD	1	-0.02556	1
RGL2	0.644245	0.034069	0.837353
PFDN6	0.347246	0.177313	0.632606
WDR46	0.193446	0.987758	0.487504
GSTA4	0.073739	-0.94561	0.316871
NKRF	0.139177	0.96752	0.425119
GNPAT	0.071226	0.407837	0.311134
KMO	0.621836	0.286753	0.825962
LAMA5	0.006911	-0.73825	0.099675
ZNF185	0.374984	-0.21907	0.656862
MATN3	0.035703	0.934614	0.225628
CASC3	0.105994	1.006988	0.370631
MRPS12	0.818856	0.050013	0.92687
NDUFA1	0.931372	-0.06036	0.984157

LEPROT	0.835355	-0.5379	0.935999
CLIC2	0.007628	-0.86923	0.10525
ACOX3	0.902877	-0.21536	0.971583
RER1	0.709387	0.02662	0.869179
SURF4	0.00266	0.591127	0.057508
MAPK13	0.008089	-0.45094	0.107782
ATXN7	0.435689	0.157737	0.700018
SPTLC1	0.136904	0.153124	0.421544
SPTLC2	0.546147	-0.26037	0.778738
OGT	0.003823	0.392995	0.070612
ALOX15B	0.531923	-0.39436	0.77068
PMM2	0.845718	-0.01606	0.942239
POLR3G	0.338495	0.699363	0.625708
TM9SF1	0.06524	0.334404	0.298136
INPP4B	0.009804	1.917975	0.119724
CHAD	0.306086	0.216649	0.607626
MID1	0.134935	0.86706	0.417833
HMGB3	0.871617	0.023821	0.956098
PPM1G	0.923804	0.000222	0.980751
INPPL1	0.155183	0.278994	0.444958
FANCA	0.749046	-0.02248	0.890902
EIF3D	0.470127	0.005523	0.72456
EIF3H	0.923804	-0.02995	0.980751
SLC16A4	0.71099	0.24672	0.870136
HDAC3	0.016733	1.124408	0.156698
NVL	0.325683	0.40424	0.616409
BCAT2	0.2958	-0.1822	0.598344
SIGLEC5	0.416936	0.256248	0.687847
TMPRSS2	0.099213	-1.1258	0.358779
NCAM2	0.110383	1.048768	0.378842
IPO8	0.085137	0.661496	0.335537
STX7	0.577274	0.091619	0.796634
TOX3	0.296639	-0.46753	0.599275
TNRC18	0.745068	0.118274	0.888646
SLC16A3	0.015629	1.039418	0.152856
SLC31A1	0.009172	2.567118	0.116274
ABCC3	0.400346	-0.39429	0.67622
ABCC4	0.789358	0.251115	0.911483
ABCC5	0.744634	0.134465	0.888419
CD3EAP	0.343341	0.794715	0.630156
TLR3	0.40398	0.673099	0.678271
P4HA2	3.86E-07	1.427876	7.02E-05
MAGEB2	0.437275	0.526071	0.70193
CAPN5	0.604483	-0.41551	0.813002
GYG2	0.038799	1.014271	0.236048
PLA2G10	0.046045	-0.90376	0.252441
YKT6	0.031791	0.321941	0.214212
NUPL2	0.768442	-0.20244	0.901915
ARPC5	0.103963	0.232673	0.366863
POLR2D	0.309724	0.414976	0.609864
CLOCK	0.547842	-0.27834	0.779889
CFLAR	0.969389	0.020734	0.997592
DDX3Y	0.757524	0.190479	0.896847
MAFG	0.760503	0.354101	0.89779
CYP27B1	0.310183	0.239434	0.609864
PDPK1	0.960544	-0.04386	0.994122
TAPBP	0.764084	0.00873	0.899242
FABP7	0.657906	-0.23813	0.842898

RNF113A	0.970476	0.481154	0.997592
KDM6A	0.534152	0.352486	0.771496
CLDN3	0.444482	-0.7574	0.706268
KCNN4	0.062362	1.142286	0.292127
DHX15	0.066208	0.176271	0.300455
RNMT	0.010002	0.458275	0.120967
ZZEF1	0.084549	0.249679	0.335025
ASAP2	0.833363	0.275692	0.935999
FLRT2	0.416636	0.739175	0.687635
TTI1	0.030344	0.987995	0.209948
PLXNB1	0.277875	-0.6052	0.579677
RRP8	0.213689	0.537114	0.509228
PJA2	0.019721	1.209684	0.1684
SIPA1L1	0.320557	0.717868	0.613139
CYB5B	0.118765	0.222101	0.392409
PRPF4	0.213733	0.189483	0.509228
PHGDH	0.029257	0.469196	0.20681
NDUFS4	0.241676	0.422243	0.544139
ARHGAP6	0.987478	-0.0725	1
ADAM12	0.052184	1.065124	0.267704
CRX	0.988991	0.11987	1
IRAK2	0.744555	0.172518	0.888419
PHF1	0.04624	0.943734	0.253009
GPR39	0.720513	0.108057	0.87575
SEPTIN4	0.41301	-0.17221	0.684707
DYNC1LI2	0.136904	0.195827	0.421544
KLK10	0.787673	-0.08961	0.911483
PSMD3	0.043893	0.218694	0.247494
SLC7A4	0.563678	-0.18445	0.789479
RBFOX2	0.00018	1.726634	0.00976
PAPSS1	0.015914	0.519539	0.153751
ZNHIT1	0.681268	0.202308	0.856391
ZW10	0.206534	0.200551	0.500256
PRODH	0.697084	0.54064	0.863357
SPINT1	0.332231	0.320789	0.620025
EFS	0.33352	0.607559	0.620902
B4GALT5	0.152354	0.6685	0.443165
SART1	0.048208	0.414412	0.257798
SPINT2	0.786816	-0.13268	0.911452
GPAA1	0.02152	0.364022	0.175496
DAPK3	8.57E-05	1.073568	0.005524
TGFB1I1	0.000116	0.982389	0.007119
AP5Z1	0.021329	0.586319	0.174974
HSPA12A	0.025979	1.007031	0.193808
CCP110	0.368302	0.435393	0.651177
ADCY6	0.481556	0.213438	0.733586
CTIF	0.0002	1.68458	0.010594
MTSS1	0.682281	0.400319	0.85709
ATMIN	0.705459	0.060927	0.868107
PPIP5K2	0.086906	0.911335	0.337854
MAP3K7	0.095005	0.775109	0.35136
EEF1E1	0.374984	-0.04589	0.656862
LYRM1	0.782206	0.271913	0.909001
MSI1	0.315978	0.577408	0.613139
RIPK2	0.032442	1.171609	0.216498
WDR62	0.383477	0.347848	0.663206
HNRNPR	0.088177	0.19223	0.339301
PRPF3	0.613839	0.08107	0.819264

TXNL1	0.392997	0.085246	0.671013
TPD52L2	0.324076	0.092151	0.614309
EMC8	0.490639	0.076646	0.738656
COCH	0.032251	1.288815	0.215938
ERI3	0.563832	0.124316	0.789479
PRKRIR	0.352067	0.393845	0.637306
SYNJ1	0.016813	1.182753	0.156698
FIBP	0.71923	-0.20889	0.874478
EIF4G3	0.000554	1.317181	0.021722
PPIH	0.149515	0.445997	0.438811
MGAM	0.401633	-0.56167	0.677774
MBTPS2	0.041014	1.128964	0.240826
HTRA2	0.374984	0.147378	0.656862
KLF4	1	-0.03619	1
AKR7A2	0.744041	-0.10607	0.888303
PROM1	0.603077	-0.36516	0.812922
EPB41L2	0.157109	0.22635	0.447022
TGOLN2	0.23772	-0.33666	0.540388
RAD51C	0.248359	0.358939	0.551794
LAMTOR5	0.709387	0.041237	0.869179
B4GAT1	0.497152	-0.57694	0.744118
MED7	0.022424	1.329379	0.179962
WIPF1	0.007758	1.089641	0.105988
ATP8B1	0.396617	-0.43464	0.67382
BCL2L11	0.018083	1.419181	0.16292
FOXO3	0.22454	0.604553	0.523628
CHST10	0.321952	0.380586	0.613139
XRCC3	0.744387	-0.14202	0.888419
TGM5	0.34344	-0.39375	0.630156
SGCE	0.045674	1.737799	0.251649
RGS14	0.000699	1.953174	0.024703
RNF13	0.833924	-0.38657	0.935999
CA12	0.20274	1.096877	0.496815
AKAP10	0.109779	1.141609	0.377536
SYT7	0.77903	0.115125	0.906856
DENR	0.020781	0.338547	0.172048
PSTPIP1	0.817979	0.012874	0.92687
XPOT	0.018045	0.757065	0.162744
SPRY2	0.322009	0.082639	0.613139
DNPH1	0.511623	-0.17795	0.754376
SPRY1	0.993518	-0.04229	1
TIMM44	0.144006	0.266716	0.431666
TRAPPC3	0.684992	0.107693	0.85709
CHMP2A	0.709387	0.06569	0.869179
NCK2	0.146729	0.626063	0.435709
PSCA	1	-0.04668	1
TSPAN6	0.350635	0.437216	0.635657
PLRG1	0.105374	0.303492	0.369374
PRC1	0.485202	0.360474	0.735764
RGS10	0.032318	0.922029	0.215938
ZNF207	0.112657	0.254946	0.382523
NDUFB5	0.577274	0.110401	0.796634
NDUFB3	0.149515	0.231003	0.438811
NDUFC1	0.632674	0.415529	0.831021
NDUFA2	0.415291	-0.13917	0.686028
ASNA1	0.181644	0.130677	0.472575
BUB1	0.241325	0.212074	0.544139
BUB3	0.33719	0.054622	0.624371

AKAP7	0.683728	-0.0727	0.85709
PPAP2C	0.140042	1.404554	0.426568
PI15	0.001066	3.085932	0.031612
SULT1B1	0.898675	-0.28723	0.970517
ACTN4	0.003666	0.258517	0.06938
GSTZ1	0.786821	-0.22572	0.911452
WBSCR22	0.37135	0.890672	0.65423
TRIAP1	0.392711	0.431946	0.671013
GATC	0.548162	-0.17369	0.780144
HTATSF1	0.470127	0.250471	0.72456
KDELR3	0.040466	1.796998	0.240216
TRAF3IP2	0.041397	0.408425	0.240826
ITM2A	0.993518	-0.1329	1
CYTH3	0.450092	-0.06142	0.709598
PRKAB2	0.002281	1.692587	0.051363
AP1G1	0.199513	0.173184	0.49217
STX6	0.709387	0.068275	0.869179
SYNGR1	0.395026	-0.90626	0.672485
SYNGR2	0.454064	-0.16519	0.712914
SYNGR3	0.244375	-0.38656	0.547425
SGTA	0.09193	0.51795	0.345626
LIAS	0.231403	0.717272	0.532247
ENSA	0.931664	0.401859	0.984157
SLC25A20	0.241676	-0.1861	0.544139
NARS	0.023873	0.232274	0.186463
KRT86	0.145657	1.122461	0.434185
SPOP	0.684849	0.2929	0.85709
MYO1B	0.286754	-0.10162	0.590053
SSNA1	0.236593	-0.71961	0.538047
SLC25A17	0.213774	0.781889	0.509228
NUDT21	0.039276	0.263677	0.236232
DUX1	0.322009	0.112696	0.613139
LANCL1	0.913333	-0.04492	0.975706
STRN	0.082198	0.144089	0.330671
RRP9	0.308157	0.257799	0.609277
SCO2	0.037972	-0.56577	0.233324
C21orf2	0.066681	1.325345	0.301803
AKAP8	0.496547	0.924931	0.74366
GTPBP6	0.873982	0.301614	0.957716
SLC37A4	0.799289	-0.6403	0.916173
ZBTB14	0.536411	0.234291	0.773175
IDH3B	0.563832	-0.09365	0.789479
NRD1	0.159051	0.740346	0.449897
CALU	0.000242	0.613544	0.012083
EDIL3	0.122791	1.344676	0.399027
ATP9B	0.970852	0.18849	0.997592
AHCYL1	0.950035	0.015798	0.990863
CD5L	0.253772	0.915178	0.557407
XPNPEP2	0.221132	0.462898	0.519064
KIF1C	0.533238	0.451244	0.7709
GAS2	0.019926	-1.03326	0.169351
EXTL3	0.684184	0.262139	0.85709
ORC5	0.291142	0.766776	0.595183
TYROBP	0.160231	0.878303	0.452801
FIGF	0.014023	-0.97701	0.144006
NDUFS5	0.825116	0.031781	0.930395
PDE6D	0.156143	0.243886	0.446449
CXCL13	0.681785	-0.49684	0.856945

ORC4		0.992101	-0.16537	1
PEX1		0.960456	-0.36259	0.994122
RAD21		0.118765	0.281178	0.392409
AKR1B10		0.503264	0.623434	0.747616
TIMM8A		0.426712	-0.11423	0.693599
KALRN		0.771987	0.207115	0.903653
DHX16		0.052873	0.72422	0.269122
SSSCA1		0.056188	0.448345	0.276683
GMFG		0.764083	0.256464	0.899242
PPP1R12B		0.950012	-0.15265	0.990863
BNIP3L		0.539196	0.679614	0.775042
SH3BP5		0.224923	0.305348	0.52386
PLIN1		0.655601	-0.37151	0.84209
HS6ST1		0.903844	0.22373	0.972246
MED14		0.041201	0.682216	0.240826
PCDH7		1.49E-05	2.557049	0.001321
PRPSAP2		0.190424	-0.14849	0.483515
GNG7		0.062864	-0.90633	0.293445
SMARCA5		0.850885	0.064446	0.944945
ADCY3		0.582301	0.193926	0.798306
KIAA0513		0.15686	-0.9213	0.447022
SPAG9		0.632496	0.076771	0.830965
SUSD5		0.010501	1.09452	0.124559
ZNF292		0.352147	0.294646	0.637347
KIF5C		0.725945	-0.03347	0.878021
URB1		0.286741	0.474276	0.590053
MGRN1		0.282269	0.706827	0.58545
SIPA1L3		0.305454	0.392247	0.606804
ZFC3H1		0.901249	-0.00783	0.971583
TRAK2		0.145176	0.324562	0.433769
LZTS3		0.6787	-0.31985	0.855378
AQR		0.081043	0.340005	0.328405
MAST3		0.683359	0.334135	0.85709
OPA1		0.257958	0.10197	0.560462
ZEB2		0.197877	0.743224	0.491413
MCM3AP		0.467999	0.279852	0.722799
PIP5K1C		0.054581	0.793546	0.273507
KIF1B		0.437742	0.193376	0.701933
MAPKBP1		0.332234	-0.43749	0.620025
	6-Mar	0.517175	0.300951	0.75923
KDM1A		0.651389	0.086792	0.839222
TBC1D4		0.018771	0.852781	0.165951
FZD6		0.530339	0.433146	0.768764
HBP1		0.354428	0.350041	0.63835
FADS1		0.002196	2.125782	0.05007
PPL		0.0334	-0.40204	0.219585
DFNA5		0.019032	0.968032	0.166597
EVI5		0.027102	1.289576	0.199066
LY75		0.947409	0.100904	0.990063
NRP2		0.001488	1.493103	0.039474
MAN1A2		0.175259	0.279604	0.466796
GPR137B		0.003187	0.979467	0.064739
PLXNC1		0.00094	1.035808	0.02929
MPZL2		0.679473	-1.00383	0.85545
ACSL4		0.474191	0.132938	0.727018
SNX3		0.074384	0.171732	0.317894
DOK2		0.233787	0.12024	0.535403
STX10		0.162993	0.284296	0.453012

MGEA5	0.004707	0.934189	0.079703
ADCY9	0.911261	-0.10443	0.975706
SORBS3	0.637197	0.368989	0.833511
SYNCRIP	0.016499	0.280808	0.155558
TPST1	5.15E-05	1.631723	0.003688
CDC40	0.280828	0.229921	0.583434
B4GALT3	0.646844	0.190194	0.837416
B4GALT4	0.098077	1.048365	0.357585
EIF4EBP3	0.092612	-0.73538	0.347013
RANBP6	0.295726	0.610941	0.598344
NEMF	0.600007	0.270361	0.810262
GMDS	0.613839	-0.09589	0.819264
NMT2	0.060068	1.44061	0.286914
CCNT1	0.01624	1.659128	0.154611
GREM1	0.002918	2.714969	0.061522
PLOD3	0.000329	0.653303	0.015317
EIF4E2	0.189109	0.324366	0.481831
CCNT2	0.24005	0.795517	0.542902
TLR5	0.012744	2.04187	0.137944
TLR2	0.97106	-0.02685	0.997592
GFRA3	0.260033	0.907133	0.563617
DIAPH1	0.856059	-0.01648	0.946993
SELENOF	0.029749	0.528871	0.208169
TSPAN1	0.519655	-0.66018	0.760272
TSPAN2	0.879408	0.134482	0.96104
EXOC3	0.866426	-0.02213	0.953042
PDE8A	0.650137	0.138463	0.839222
PLIN3	0.001057	0.534138	0.031507
SLC16A7	0.592958	-0.39675	0.806337
RAD1	0.347512	0.527249	0.632849
JAK2	0.014	1.207747	0.144006
MAFK	0.715128	-0.40804	0.872263
PRMT3	0.689296	0.604155	0.859429
PEX10	0.530339	0.479523	0.768764
KPNA6	0.009812	0.371338	0.119724
SRPX2	2.19E-05	2.886332	0.001855
UGDH	0.327323	0.325179	0.617719
TPST2	0.552221	0.146724	0.783108
ABCC9	0.608679	0.175569	0.816529
LPXN	0.041861	0.52012	0.242066
CTNND1	0.503174	0.099112	0.747596
ICMT	0.214949	0.694637	0.511258
MAGEC1	0.300882	0.380856	0.602797
PLA2G6	0.123186	-0.71369	0.399387
EIF1B	0.568426	0.527188	0.791838
SNX2	0.025563	0.176038	0.193268
HPGDS	0.913813	-0.07889	0.976031
DPM1	0.577274	0.114921	0.796634
USO1	0.357491	0.179625	0.641472
SLC19A2	0.172617	0.385401	0.464157
MRPS14	0.417074	-0.00949	0.687847
TOM1	0.051278	0.195367	0.265139
PFKFB2	0.845716	0.068457	0.942239
CCDC22	0.908103	0.018998	0.9734
PQBP1	0.085043	1.862459	0.335537
TIMM17B	0.934266	-0.28635	0.984157
PRAF2	0.067188	0.463111	0.302058
DKC1	0.568296	-0.02963	0.791838

EIF5B	0.130075	0.226895	0.410899
ZG16	0.440215	-0.24916	0.70314
TRIM13	0.678898	0.398326	0.855378
GAS7	0.357273	0.9921	0.641472
EDF1	0.54177	-0.05756	0.775521
KIN	0.041408	1.056606	0.240826
DIAPH2	0.190424	-0.40731	0.483515
SH2D1A	0.456054	0.237836	0.714622
DNAJA2	0.142204	0.248429	0.428816
BRD4	0.017412	0.457636	0.159467
CUTA	0.404052	0.269855	0.678271
OPHN1	0.315665	-0.68904	0.613139
RAMP2	0.592691	0.352011	0.806337
SMPD2	0.558794	-0.33374	0.78633
TBL1X	0.577652	0.297822	0.796675
CTSV	0.721191	0.154653	0.876242
HUS1	0.028124	1.773564	0.203207
PFDN1	0.400346	0.037177	0.67622
PPP1R11	0.604318	0.744149	0.813002
RNASEH1	0.150211	0.855239	0.440175
NBN	0.00187	0.893526	0.045694
NOL3	0.680152	0.268417	0.85545
DTNB	0.011406	-0.7593	0.128971
RNGTT	0.075091	0.875224	0.319336
FCGR3B	0.180549	0.805721	0.472575
LILRB3	0.229894	0.680495	0.529875
LILRB5	0.55076	0.422213	0.781335
ABCB7	0.850885	-0.07938	0.944945
MITF	0.06743	0.964941	0.30217
KIF21B	0.420517	-0.65521	0.690548
SRGAP2	0.000107	1.134634	0.006638
PLXNA2	0.435753	0.507504	0.70002
NOS1AP	0.959748	-0.03963	0.994122
IGSF3	0.105675	1.070287	0.370197
SDC3	0.81719	-0.06362	0.926828
FAM20B	0.404026	0.551997	0.678271
DENND4B	0.515427	0.326334	0.757825
EFCAB14	0.971031	0.171604	0.997592
WDR1	0.131757	0.166911	0.413476
FZD7	0.053192	1.307596	0.270374
SLIT3	0.896724	-0.00116	0.969793
MEGF6	0.763945	-0.02581	0.899242
LRP4	0.581954	-0.02437	0.798127
ATP9A	0.79444	-0.12545	0.913708
LDB3	0.201508	-0.85778	0.49489
N4BP1	0.971054	0.25031	0.997592
ROCK2	0.61848	0.101403	0.822194
CLASP2	0.084548	0.556185	0.335025
PTCD1	0.457123	0.446749	0.715224
COBL	0.231217	-0.59083	0.531962
CPNE3	0.729121	-0.12061	0.879656
DEPDC5	0.627472	0.290894	0.828932
ATG13	0.204196	0.910563	0.497963
ICOSLG	0.066061	0.970632	0.300455
HIP1R	0.466082	-0.18274	0.721065
OBSL1	0.057916	0.82156	0.280499
RNF40	0.434427	0.365905	0.698194
PHF2	0.501832	0.15665	0.747291

ZC3H11A	0.12513	0.610253	0.401334
CLUH	0.1882	0.457682	0.480067
CAND2	0.505015	0.293934	0.749514
TSC22D2	0.258333	0.684573	0.560808
NPHP4	0.210617	-0.23133	0.506604
KDM4A	0.018308	1.003702	0.163766
DNAJC13	0.001008	0.383423	0.030643
PHACTR2	0.808416	0.273424	0.921586
PPP6R2	0.324076	0.308126	0.614309
ADAMTS4	0.000645	2.665785	0.023592
CNOT3	0.003904	0.818786	0.071121
SS18L1	0.197201	-1.02049	0.490309
ANKRD17	0.010382	1.044248	0.123374
SIN3B	0.052577	0.983313	0.268973
ATP2C2	0.216819	-0.65481	0.513662
DNAJB6	0.430547	0.77477	0.695651
XYLB	0.017236	1.634543	0.158939
PEX11A	0.589979	-0.30688	0.804875
LRP5	0.221359	0.480101	0.519377
TMEM127	0.472922	0.245363	0.727018
COQ9	0.082198	-0.31194	0.330671
GGCT	0.734083	-0.06784	0.882858
NDUFS7	0.45805	0.08324	0.715224
RTN2	0.013356	1.614845	0.141484
NDUFS2	0.108241	0.21311	0.374055
UGT2B11	0.300882	0.26458	0.602797
ZPR1	0.371443	0.405432	0.65423
USP12	0.5073	-0.2771	0.75153
DUSP11	0.033976	1.20215	0.221145
GBAS	0.794443	-0.02074	0.913708
SEMA7A	0.002889	1.298559	0.061037
HMMR	0.128993	1.302515	0.409111
CILP	1.69E-08	4.880654	6.25E-06
PDCD6	0.520146	0.059982	0.760272
TBCA	0.61848	-0.10269	0.822194
ATP6V1G1	0.749036	-0.02915	0.890902
VPS4B	0.308157	0.12362	0.609277
MPDU1	0.0799	0.382938	0.325919
ENTPD6	0.021149	1.3754	0.173882
ENTPD3	0.411372	0.421644	0.683152
ENTPD5	0.891226	-0.02631	0.966699
ZNF217	0.015511	1.077821	0.15278
BCAS1	0.905133	-0.25223	0.972966
PTP4A3	0.606873	0.178616	0.815541
AVIL	0.047645	0.671866	0.256521
H2AFY	0.173168	-0.18352	0.464476
SH3BGRL	0.515876	0.073855	0.757825
FLNB	0.197211	0.10751	0.490309
NCOR1	0.340302	0.444703	0.627258
VAMP4	0.014171	1.033792	0.145123
NDUFS6	0.641914	0.016561	0.835764
PEX14	0.72417	0.088894	0.876654
TRIM3	0.033947	0.831902	0.221141
ULK1	0.262147	0.237536	0.565636
TULP3	0.073475	0.766518	0.316346
SLC43A1	0.622736	-0.28123	0.825978
CS	0.892438	0.014102	0.966699
SPAG7	0.768774	-0.45285	0.901915

MRPL33	0.872945	-0.31184	0.956872
SEC22B	0.213733	0.198984	0.509228
DEAF1	0.729045	0.149956	0.879656
PRPF40A	0.131757	0.197901	0.413476
TACC1	0.009264	0.581628	0.116462
NME6	0.646168	-0.29532	0.837353
CDC45	0.385801	0.664544	0.664326
GIGYF1	0.062711	1.108139	0.292924
MOSPD3	0.09909	-0.48146	0.358568
LRCH4	0.774166	-0.05106	0.903913
MTX2	0.72417	-0.00202	0.876654
VPS26A	0.244338	0.18561	0.547425
NDUFB1	0.289748	0.116943	0.593134
PMPCB	0.680152	-0.06885	0.85545
SAP30	1	-0.06621	1
MED24	0.217399	0.281783	0.514768
KATNA1	0.24217	0.976307	0.545141
RDH16	0.050224	-0.70801	0.263221
ERN1	0.720872	0.22126	0.876089
CRLF1	0.53582	-0.40702	0.773095
LGR5	0.178261	0.258354	0.469506
PSIP1	0.729121	0.104732	0.879656
ERLIN1	0.515876	0.204681	0.757825
TADA2A	0.041397	0.402976	0.240826
SUPT3H	0.010713	1.361852	0.125864
GPC4	0.069183	0.560343	0.306571
NDUFS3	0.35064	0.142921	0.635657
CA11	0.746543	-0.11661	0.889644
SRSF10	0.272095	0.190206	0.575039
CLN5	0.255193	-0.18162	0.557593
HSBP1	0.001272	0.641311	0.034924
TNFRSF21	0.710459	-0.00676	0.870109
ECI2	0.059675	-0.34966	0.285693
KHDRBS3	0.432742	0.794089	0.697709
TADA3	0.076472	1.03456	0.322705
TAF5L	0.980873	0.056617	1
EED	0.498013	-0.03422	0.744803
BANF1	0.016204	-0.28102	0.154611
SF3B1	0.097132	0.209745	0.354489
CSDE1	0.411524	0.126933	0.683152
WBP4	0.317391	0.615955	0.613139
STX11	0.586298	0.044177	0.801141
SKAP2	0.396189	0.790325	0.67382
PRKRA	0.97106	-0.06733	0.997592
MTRF1	0.718667	0.205275	0.874478
CRCP	0.670702	0.166462	0.850681
ITGA10	0.949005	0.055186	0.990863
RPS6KA5	0.330298	0.226452	0.619341
MED6	0.034175	1.315494	0.221721
MYCBP2	0.396661	0.07752	0.67382
PGLYRP1	0.884537	-0.52814	0.963599
GCAT	0.548032	-0.39488	0.780059
SPAG6	0.995583	-0.04885	1
NPM3	0.025549	0.981977	0.193268
LYPLA1	0.955289	-0.0985	0.993074
ERAL1	0.277156	0.373096	0.579677
REM1	0.768139	-0.01827	0.901915
CREG1	0.048136	1.738373	0.257798

SERPINB7	0.602618	0.310835	0.8126
FCN3	0.956176	0.319331	0.99381
SNRNP200	0.031791	0.279154	0.214212
TRMU	0.526332	-0.39589	0.765982
TIPRL	0.00719	0.483943	0.101457
OFD1	0.635785	-0.3479	0.833511
TOM1L1	0.198683	-0.98137	0.49217
RPS6KA4	0.011202	0.71339	0.128397
SURF6	0.895412	0.284221	0.969069
PPM1B	0.247021	-0.22314	0.549922
ADAP1	0.083377	0.949515	0.332962
UTP20	0.415291	0.26389	0.686028
NUP155	0.073319	0.223063	0.31592
RP2	0.764084	0.012456	0.899242
GJB3	0.57907	-0.10514	0.796675
STK16	0.555709	0.457602	0.783439
WDHD1	0.015127	1.553504	0.150467
CRTAP	2.58E-10	1.328131	4.08E-07
SLC25A12	0.005656	0.453706	0.088608
PIK3C2G	0.666736	0.174866	0.848351
SLC22A3	0.464421	-0.62723	0.721065
B3GALNT1	0.038481	0.901964	0.235279
TCEA3	0.083234	-0.99072	0.332962
PALM	0.923792	0.438038	0.980751
ATP6AP2	0.330591	0.302705	0.619341
GRAP2	0.614909	-0.5164	0.820298
RNASEH2A	0.601849	0.592536	0.81176
CDC123	0.216839	0.622569	0.513662
UGT2B17	0.331588	-0.18105	0.619341
CAPN15	0.118817	0.442322	0.392434
BCAR3	0.183026	0.534949	0.474175
POP7	0.350846	0.402224	0.635927
RPP40	0.05702	0.675064	0.278658
EIF3G	0.699592	0.015948	0.864146
EIF3J	0.067188	0.317869	0.302058
CBR3	0.239034	-0.18032	0.541047
LECT1	0.179677	0.689168	0.471223
PSMD10	0.503174	0.177344	0.747596
UPK1B	0.310183	0.344883	0.609864
AP1G2	0.581788	-0.04492	0.797998
ZMPSTE24	0.026447	0.487186	0.195939
PPP1R37	0.503037	0.332763	0.747596
CEACAM4	0.08528	-0.41395	0.335537
IDH1	0.078771	-0.19919	0.32578
GATB	0.43761	0.115902	0.701933
SCO1	0.364413	-0.22004	0.646996
CYP7B1	0.061485	0.593731	0.289565
ATRN	0.038006	0.916326	0.233324
RBBP9	0.706933	-0.2035	0.869179
STAM2	0.009812	0.367294	0.119724
TNFSF13	0.494367	0.291184	0.741997
ALDH1L1	0.058956	0.941067	0.283643
TUSC2	0.562547	-0.30761	0.789479
SULT1C4	0.571349	0.083634	0.793891
MMP23A	0.304619	0.767856	0.606083
DGAT1	0.734069	0.27719	0.882858
CCNK	0.001133	1.006208	0.032731
DHRS3	0.430489	-0.21601	0.695651

ARL6IP5	0.043893	0.306012	0.247494
DYSF	0.866426	-0.00032	0.953042
PIAS1	0.036623	1.290234	0.229485
BCAS2	0.308157	0.239197	0.609277
DCTN3	0.192667	0.033875	0.486094
BBOX1	0.319672	-0.21336	0.613139
DNAJC8	0.774166	0.127842	0.903913
SMNDC1	0.050885	0.418764	0.264901
RAD17	0.91353	-0.00409	0.975822
ATP5H	0.340521	-0.05919	0.627258
DNAJB5	0.126551	0.460666	0.403998
TSPAN9	0.858616	-0.16232	0.949134
FLOT1	0.016799	-0.2332	0.156698
TRIO	0.002385	0.693144	0.053103
ATP5L	0.609213	-0.04678	0.816529
MPDZ	0.024558	1.21845	0.189804
CPD	0.056188	0.442927	0.276683
SASH3	0.726643	0.084511	0.878483
GLRX3	0.010589	0.549979	0.124802
KRT36	0.319672	0.186658	0.613139
RSL1D1	0.651389	-0.14856	0.839222
WFS1	0.316049	0.214135	0.613139
ANXA9	0.344056	-0.68013	0.630315
CLPX	0.651389	0.222372	0.839222
SCGN	0.421207	-0.31018	0.691066
NEBL	0.018882	-0.81294	0.166597
SEC14L2	0.056538	1.8898	0.277829
STC2	0.849305	0.255545	0.944945
TM7SF2	0.060238	-1.77455	0.287134
SNCG	0.889133	0.605923	0.96576
CIAO1	0.199513	0.184774	0.49217
PDE5A	0.343366	-0.4689	0.630156
DFFB	0.907303	-0.05335	0.9734
WISP2	0.383711	1.171346	0.663509
ZFAND5	0.504761	0.339927	0.749338
SRP72	0.249724	0.162982	0.552833
DXO	0.46818	0.190111	0.722799
TRPM2	0.320828	0.497198	0.613139
DDAH1	0.005889	-0.83916	0.089865
RECQL4	0.054852	0.318463	0.274162
RECQL5	0.40369	0.632629	0.678271
URI1	0.141805	0.806216	0.428816
B3GAT3	0.24165	0.457083	0.544139
STK17B	0.021982	1.328091	0.177307
ECM2	1.40E-06	3.189353	0.000194
LY6H	1	0.002188	1
MTA2	0.012773	0.301239	0.137944
ALDH1A2	0.04791	1.03457	0.257569
STK10	0.699592	-0.00135	0.864146
PRKD3	0.15102	0.776849	0.44125
GFPT2	0.014667	1.757077	0.148149
TPPP	0.234778	-0.85587	0.536782
SLIT2	0.075598	0.77802	0.32039
KBTBD11	0.705956	-0.17408	0.868525
LTN1	0.030754	0.766998	0.211475
ATP10B	0.640002	-0.31903	0.835224
TOMM70A	0.017412	0.285241	0.159467
IPO13	0.000897	2.049242	0.028766

DDHD2	0.024168	1.398294	0.188238
MYO1D	0.102567	0.344621	0.364372
TOX4	0.422171	0.48352	0.691467
MICAL2	0.00187	0.81783	0.045694
KIAA0754	0.09909	0.286197	0.358568
SEC24D	1.14E-05	0.63896	0.001067
NFASC	0.072874	-0.88108	0.315444
SUPT7L	0.182865	0.729534	0.474175
FCHSD2	0.174952	0.539083	0.466796
UFL1	0.12841	0.205676	0.407846
SORBS2	0.856059	0.175009	0.946993
TMCC1	0.002688	1.620083	0.057778
PHF14	0.036226	0.783452	0.227448
SASH1	0.132125	0.79893	0.414307
TMEM63A	0.081043	-0.48265	0.328405
FARP2	0.699462	0.001077	0.864146
UBXN7	0.193795	0.413727	0.488273
SUN1	0.997369	0.037769	1
PROSC	0.680152	0.054295	0.85545
ERLIN2	0.550543	0.084428	0.781327
PRPF6	0.173168	0.213783	0.464476
DKK1	0.310183	0.270727	0.609864
ABCA8	0.007698	-1.64641	0.105935
PCF11	0.550229	0.424318	0.781327
FRYL	0.364426	0.300505	0.646996
NFAT5	0.974406	0.115225	0.998921
ENDOD1	0.572777	-0.02193	0.794481
CDK14	0.005306	1.282801	0.086166
GLCE	0.403018	-0.35582	0.678271
GLS	0.804634	0.0668	0.918848
HAUS5	0.708113	-0.1784	0.869179
ABLIM3	0.661762	-0.20167	0.844895
FBXO21	0.147601	0.936191	0.436386
KDM4B	0.157405	0.572049	0.447293
RHOBTB3	0.060497	0.669913	0.287134
SLCO2B1	0.052499	1.080799	0.268947
SOGA1	0.118516	0.689705	0.392409
USP19	0.151364	0.386084	0.44125
WDR47	0.146282	0.815564	0.435196
TRIM37	0.749046	0.009881	0.890902
AP2A2	0.568296	0.074616	0.791838
SEC31A	0.006775	0.266843	0.098484
CLSTN1	0.001967	2.031839	0.047009
CEP152	0.128538	0.445375	0.408017
FAM13A	0.991426	0.01243	1
ARHGEF15	0.168344	-0.73219	0.459086
HEXIM1	0.036826	0.633674	0.229759
TJP3	0.002491	-1.18842	0.054809
INMT	0.647731	0.268916	0.838174
RPP14	0.096858	0.922944	0.354489
CCNB2	0.545822	-0.04291	0.778738
YIF1A	0.131753	0.444173	0.413476
UBR5	0.052873	0.609505	0.269122
AGFG2	0.491774	-0.32837	0.739862
PRSS23	0.040727	1.603846	0.240826
SCAF4	0.272095	0.183807	0.575039
S1PR2	0.668509	-0.34082	0.849597
NDUFB6	0.887225	0.25325	0.96416

MFN2	0.845718	-0.05254	0.942239
DUSP14	0.897443	0.100127	0.969793
SNUPN	0.195986	0.720909	0.490309
AKR7A3	0.739224	0.142108	0.886253
UBE4B	0.011851	0.9244	0.13136
ZFPL1	0.498977	-0.19633	0.744839
IKBKAP	0.277896	0.189964	0.579677
UBL3	0.292597	-0.98725	0.595788
GABARAP	0.511368	-0.78741	0.754376
NDUFA3	0.170026	0.349645	0.461404
NDUFB4	0.939534	0.04353	0.986498
NDUFB8	0.241676	0.261697	0.544139
SCEL	0.300647	-0.7372	0.602797
NDUFA7	0.289748	-0.30617	0.593134
VAMP5	0.694617	-0.14697	0.861478
RTN3	0.09982	0.245811	0.359216
KLHL2	0.995826	-0.06436	1
RCBTB2	0.267796	0.612937	0.570195
LETM1	0.680152	-0.03973	0.85545
EPN2	0.124024	0.616638	0.400153
STBD1	0.078725	-0.62427	0.32578
LEPROTL1	0.209546	0.893468	0.504466
ZRANB2	0.143873	0.81757	0.431666
SNX4	0.030755	0.360007	0.211475
ZWINT	0.023553	0.963751	0.185053
LUC7L3	0.007932	0.482639	0.107292
KIF20A	0.200849	0.660416	0.494233
APOL3	0.908098	-0.51168	0.9734
KIF4A	0.215185	0.757593	0.511491
MBD4	0.75311	0.197024	0.894106
SBF1	0.023065	0.411944	0.182986
GOSR1	0.38573	0.217186	0.664306
KAT7	0.311297	0.475502	0.609894
ABCC6	0.001344	-1.26747	0.036363
SLC25A14	0.006623	1.103358	0.097053
ATE1	0.166751	0.83965	0.457139
CCNDBP1	0.528487	0.37473	0.767576
LYPD3	0.104977	0.89267	0.369266
EPM2A	0.716156	-0.17293	0.873229
KCNK5	0.051597	1.140765	0.266309
VAPB	0.72417	0.093592	0.876654
RASAL1	0.818809	-0.25623	0.92687
SNAPIN	0.354055	0.144854	0.638094
MPZL1	0.018368	0.849416	0.163766
NDUFC2;NDUFC2-KCTD14	0.632492	0.010962	0.830965
NDUFA10	0.179497	0.221422	0.471072
FKBP9	5.18E-08	0.921739	1.47E-05
CELF2	0.117214	-0.53373	0.389929
PGLS	0.840557	-0.00473	0.939212
PAPSS2	0.840557	-0.00702	0.939212
ABCB11	0.27825	0.439113	0.580198
SMC2	0.041403	1.332182	0.240826
ATG7	0.689846	0.174393	0.859429
TACC2	0.552736	-0.10049	0.783393
TRIM16	0.076118	1.313801	0.321815
FARS2	0.616076	0.664541	0.821456
ZBTB7A	0.507781	0.316144	0.75191
LYPLA2	0.474191	-0.12412	0.727018

IPO7	0.524435	0.037248	0.764023
ARIH2	0.156972	0.913694	0.447022
TNFAIP8	0.675324	0.108441	0.853175
MAP3K6	0.013369	1.04791	0.141488
WISP1	0.080614	0.726583	0.327865
WISP3	1	-0.00319	1
GDF11	0.993518	0.019232	1
SLU7	0.005486	1.757406	0.087298
PGM3	0.003027	0.680629	0.062294
GCNT3	0.816233	0.058975	0.926122
MOCS3	0.057896	0.883835	0.280499
RAPGEF3	0.045425	0.584794	0.250872
CD2BP2	0.037428	0.475374	0.231646
MED26	0.771591	0.114057	0.903336
ZFYVE9	0.65654	-0.06601	0.842398
CNIH1	0.23975	1.323011	0.542335
TNFRSF6B	0.000137	2.766521	0.007999
BRI3	0.087124	-1.24618	0.3381
SVIL	5.09E-07	0.805304	8.68E-05
PIGN	0.145945	0.930384	0.434427
PAPLN	0.027806	-0.9128	0.202072
BAG4	0.028605	1.621727	0.204681
AHSA1	0.192667	0.285775	0.486094
SLC34A2	0.077094	-0.93381	0.324464
APOM	0.85408	0.883591	0.946993
ADAMTS2	0.048372	0.845487	0.258191
PARN	0.305035	0.484105	0.606083
PSMG1	0.088785	0.721914	0.34076
FMNL1	0.05451	0.294055	0.273313
SGPL1	0.09982	0.271037	0.359216
CLDN7	0.757531	0.359893	0.896847
CTDNEP1	0.915959	-0.16537	0.977664
ABCA1	0.350349	0.447954	0.635657
NSA2	0.663952	0.306572	0.847289
H6PD	0.586319	-0.12704	0.801141
SEC24A	0.000423	0.684046	0.018305
SEC24B	0.013753	0.284696	0.143228
LPHN2	0.411659	-0.60717	0.683152
VNN1	0.927729	0.038811	0.983115
VNN2	0.319599	0.643412	0.613139
CBX6	0.944302	0.005006	0.988547
SLC2A10	0.003581	1.857567	0.068752
NADK	0.944728	-0.0402	0.988547
TDP2	0.415634	0.527058	0.686287
SFT2D2	0.396926	1.064962	0.674166
MPC2	0.913333	0.282355	0.975706
METTL18	0.118773	0.770465	0.392409
ETHE1	0.026447	0.276723	0.195939
ACSL3	0.61848	0.131594	0.822194
POLR1A	0.0799	0.468095	0.325919
PCNT	0.32407	0.330874	0.614309
YEATS4	0.397243	0.620123	0.674601
ZBTB11	0.442234	0.254551	0.703704
CNOT4	0.344304	0.54423	0.630561
STAMBP	0.486499	0.142406	0.73612
NTN1	0.312494	0.232755	0.610732
FSTL3	0.102296	1.004561	0.364372
CPSF4	0.213721	0.375182	0.509228

NFATC1	0.009592	1.083611	0.118893
ASMTL	0.939534	0.117289	0.986498
CDS2	0.876815	-0.12542	0.958859
KRT75	0.772113	0.160532	0.903705
FGFR1OP	0.091853	1.232761	0.345626
PPP1R3D	0.938518	-0.06054	0.986498
BRD1	0.777261	0.246072	0.906372
POP4	0.480247	0.486199	0.732554
LY86	0.319672	0.331787	0.613139
HERC2	0.016333	0.90237	0.155231
CXCL14	0.037731	1.558694	0.233134
RAB3D	0.103963	-0.3682	0.366863
SNAP29	0.378546	0.03484	0.659304
OXSR1	0.006511	0.319261	0.096023
GGPS1	0.699526	-0.02596	0.864146
RAB36	0.861609	-0.16752	0.950384
HSPA4L	0.315327	-0.50872	0.613139
PTBP3	0.003744	0.40631	0.070138
TBC1D8	0.771016	-0.19266	0.903336
IL33	0.649527	-0.35208	0.839222
STARD3NL	0.484646	-0.82119	0.735121
LSM8	0.794443	0.054643	0.913708
AP2A1	7.16E-07	0.448667	0.000115
WIZ	0.035045	1.012446	0.223337
DDX58	0.046732	0.587516	0.254194
STAU1	0.060574	0.383488	0.287134
TTC4	0.760437	0.430247	0.89779
TMEM50A	0.835153	-0.44735	0.935999
SDPR	0.025563	-0.87121	0.193268
BAG2	0.392997	0.192327	0.671013
BAG3	0.028771	0.492974	0.204946
MAP4K4	0.000305	0.990277	0.014375
MLYCD	0.819984	-0.03844	0.92687
CRYZL1	0.724169	0.19545	0.876654
AIFM1	0.272095	-0.16264	0.575039
CLDN1	0.163587	0.674966	0.453012
CLIC3	0.050086	-0.94069	0.26298
EML2	0.206534	-0.33851	0.500256
LATS1	0.62289	0.147595	0.825978
GNA14	0.294675	0.81296	0.597922
ANGPTL1	0.40269	-0.36419	0.678038
NUDT14	0.925424	0.150799	0.981917
TSPAN13	0.749023	-0.55105	0.890902
TSPAN15	0.814832	0.236541	0.924847
BPNT1	0.554955	0.140382	0.783439
FADS2	0.026262	1.479022	0.195353
DDAH2	0.918567	0.041765	0.978657
G6B	0.57907	-0.15175	0.796675
ABHD16A	0.986843	0.007234	1
GPANK1	0.706688	0.248142	0.869179
C6orf47	0.959816	0.219916	0.994122
TXNDC12	0.43056	-0.12259	0.695651
OLFM2	0.571349	0.068217	0.793891
TRUB2	0.430496	0.343267	0.695651
ECD	0.856848	0.057848	0.947771
SYF2	0.635785	0.110897	0.833511
CBX7	0.146545	-0.83458	0.435629
TBX6		0	

ITGBL1	9.26E-08	3.767958	2.25E-05
EFEMP2	1.21E-05	1.079171	0.001115
IGSF6	0.309054	0.767153	0.609864
RECK	0.307997	0.61022	0.609277
MBD3	0.020417	1.070912	0.170514
TOP3B	0.079547	1.018688	0.325919
NUDT3	0.522249	-0.16746	0.762942
FAM107A		0	
AGR2	0.280828	-0.32994	0.583434
GAS8	0.756763	-0.14417	0.896333
BCL10	0.011376	1.625739	0.128971
NDUFB10	0.779221	-0.0094	0.906856
CLPTM1	0.897655	0.152639	0.969793
ZBED1	0.597759	0.305949	0.808505
MOCS2	0.805268	0.346532	0.919418
TOMM40	0.101186	0.291285	0.361901
NAPSA	0.177364	-0.56559	0.468602
PEX11B	0.54177	0.119481	0.775521
PAK4	0.699548	-0.04936	0.864146
WNT11	0.7956	-0.16183	0.914405
DNAL4	0.295394	-0.53474	0.598344
CHEK2	0.462316	0.48667	0.719051
APBA3	0.004056	1.425641	0.072359
ACTL6A	0.035653	0.261003	0.225452
WHSC1	0.075662	0.701693	0.320537
MOCS2	0.960048	-0.24153	0.994122
MT-CYB	0.758553	0.168253	0.896847
CYB5A	0.005322	-0.64285	0.086174
ADH1B	0.016204	-1.36075	0.154611
ADH1C	0.4308	-0.71689	0.69569
LDHA	0.151386	0.335298	0.44125
ALDH1A1	0.28378	-0.30159	0.586874
GLUD1	0.623137	0.060066	0.825978
DHFR	0.298209	0.6737	0.601676
CYB5R3	0.221112	0.175172	0.519064
GSR	0.835403	0.070692	0.935999
MT-CO1	0.55643	0.662274	0.783865
MT-CO2	0.177368	0.230429	0.468602
MT-CO3	0.246865	1.721121	0.549922
PAH	0.637936	-0.2305	0.834063
SOD1	0.835403	0.017169	0.935999
CP	0.171096	0.294566	0.461822
F8	0.023451	-0.9251	0.184845
OTC	0.744555	0.039089	0.888419
F13A1	0.076552	0.389791	0.322705
PNP	0.067188	0.47885	0.302058
HPRT1	0.201834	0.236992	0.495032
GOT2	0.33388	0.249018	0.620902
ABL1	0.034313	1.105197	0.222148
EGFR	0.520146	0.240774	0.760272
PGK1	0.269226	0.206654	0.571371
AK1	0.061485	-0.58636	0.289565
F2	0.286754	0.265635	0.590053
C1R	1.82E-06	1.245975	0.000243
HP	0.871617	0.274425	0.956098
HPR	0.05807	1.393449	0.281
F9	0.759058	-0.27187	0.896847
F10	0.400084	0.608539	0.67622

CFD	0.121911	0.486289	0.397343
PLG	0.524435	0.209763	0.764023
F12	0.020417	1.227894	0.170514
PLAU	0.000693	2.619645	0.024558
PLAT	0.635765	0.845237	0.833511
CFB	0.057044	0.448647	0.278658
ADA	0.247021	0.439134	0.549922
MT-ATP6	0.955288	-0.57826	0.993074
CA1	0.161013	-0.67604	0.453012
CA2	0.025563	-0.81679	0.193268
ASS1	0.067188	-0.89403	0.302058
OAS1	0.312831	0.895879	0.610954
SPINK1	0.573862	-0.42979	0.795688
SERPINC1	0.929044	-0.00489	0.983115
SERPINA1	0.637197	0.072132	0.833511
SERPINA3	0.269226	0.347386	0.571371
AGT	0.255193	0.364412	0.557593
A2M	0.45805	0.091694	0.715224
C3	0.511623	0.080173	0.754376
C5	0.059675	0.45234	0.285693
TIMP1	0.018697	0.615849	0.165633
CST3	0.049723	-0.48526	0.261364
CST4	0.331588	-0.24259	0.619341
CST1	0.062136	1.536956	0.291647
CSTA	0.817472	-0.07868	0.926863
KNG1	0.354055	0.204069	0.638094
FOS	0.331579	-0.47225	0.619341
NRAS	0.61158	0.560969	0.818417
HRAS	0.266378	0.131931	0.568807
KRAS	0.613839	-0.21364	0.819264
PDGFB	0.290313	0.349859	0.594164
LDLR	0.92264	0.01914	0.980751
TGFA	0.540968	0.180229	0.775521
TGFB1	0.02023	1.131861	0.170367
POMC	1	-0.0803	1
CALCA	0.992139	-0.12922	1
IGF2	0.168335	1.022577	0.459086
IGJ	0.590866	-0.22639	0.804875
IGKV1-33	0.543955	0.22689	0.777449
IGKV1-17	0.934009	0.176327	0.984157
IGKV1D-16	0.125947	1.118433	0.403148
IGKV1-5	0.901893	-0.10369	0.971583
IGKV3-20	0.632496	0.036059	0.830965
IGKV3-15	0.115676	0.272945	0.387731
IGLV1-44	0.488557	0.478855	0.738428
IGLV1-47	0.009626	1.481622	0.118893
IGLV1-51	0.870175	0.639276	0.956098
IGLV1-40	0.670819	0.356633	0.850681
IGLV2-11	0.881065	0.11255	0.961515
IGLV3-19	0.660208	-0.77511	0.844596
IGLV3-1	0.281823	0.923702	0.58479
IGLV3-25	0.773231	0.45184	0.903913
IGLV3-27	0.009764	-2.12732	0.119724
IGLV6-57	0.750655	-0.4242	0.892487
CD4	0.944782	-0.04219	0.988547
CD8A	0.294358	0.421878	0.597496
IGHV1-69	0.029203	0.777522	0.20681
IGHV1-46	0.661349	0.488609	0.844736

IGHV3-11	0.322009	0.189721	0.613139
IGHV3-48	0.681268	0.211195	0.856391
IGHV3-23	0.008016	1.226438	0.107782
IGHV3-13	0.228562	1.058352	0.528484
IGHV3-66	0.992139	-0.05717	1
IGHV3-7	0.221112	0.246675	0.519064
IGHV3-9	0.420966	-0.04509	0.691066
IGHV2-5	0.745933	0.371774	0.889299
IGHV4-61	0.505268	-0.1695	0.749689
PIGR	0.098465	-0.94618	0.357719
IGKC	0.098436	0.244617	0.357719
TRAC	0.410427	0.368759	0.683152
TRBC1;TRBC2	0.058573	1.408721	0.282818
IGHE	0.415253	-0.47692	0.686028
IGHG1	0.177368	0.197511	0.468602
IGHG2	0.239034	0.330862	0.541047
IGHG3	0.357491	0.20615	0.641472
IGHG4	0.623137	0.169974	0.825978
IGHM	0.277896	0.24431	0.579677
IGHA1	0.986843	-0.08891	1
IGHA2	0.799534	0.088763	0.916173
IGHD	0.1542	-1.30718	0.444958
HLA-B	0.068637	-1.34172	0.305604
HLA-A	0.645144	-0.32264	0.837353
HLA-A	0.913333	0.277606	0.975706
HLA-DRA	0.382127	-0.14715	0.661698
HLA-DQA2	0.770826	0.377185	0.903336
HLA-DQA1	0.634421	-0.95808	0.832706
HLA-DRB1	0.737243	-0.03519	0.885602
HLA-DRB1	0.980946	-0.01339	1
HLA-DQB1	0.520146	-0.05905	0.760272
HBZ	0.293533	0.44246	0.596247
HBD	0.051278	-0.69189	0.265139
MB	0.566553	-0.59	0.791838
COL1A1	0.00146	0.828169	0.038912
COL2A1	0.182465	0.442215	0.473679
COL3A1	2.00E-05	1.643662	0.00171
COL4A1	0.918567	-0.03444	0.978657
CRYAB	0.190424	0.383502	0.483515
KRT14	0.064281	0.634586	0.296208
KRT6A	0.016645	1.539671	0.156319
LMNA	0.76912	0.058524	0.901915
SPTA1	0.208913	-0.71264	0.503054
TNNC2	0.331588	-0.11846	0.619341
APOA1	0.67051	0.133239	0.850681
APOE	0.077655	0.333803	0.324641
APOA2	0.656149	-0.09219	0.84209
APOC1	0.627809	0.32517	0.828932
APOC2	0.664556	1.379766	0.847351
APOC3	0.12513	0.444599	0.401334
FGA	0.442223	-0.11848	0.703704
FGB	0.960544	-0.03204	0.994122
FGG	0.744041	-0.08707	0.888303
MBP	0.690892	-0.33672	0.860106
GYPA	0.900251	-0.1052	0.971583
SLC4A1	0.194929	-0.46742	0.488911
CRP	0.497979	0.429448	0.744803
APCS	0.44615	0.100734	0.706795

C1QA	0.175259	0.409619	0.466796
C1QB	0.084549	0.434866	0.335025
C1QC	0.1068	0.359574	0.371671
C9	0.020421	0.536174	0.170514
APOH	0.038652	0.400515	0.235412
LRG1	0.083337	0.678059	0.332962
FN1	4.59E-07	0.838513	7.95E-05
RBP4	0.609209	0.349672	0.816529
AMBP	0.825116	0.048574	0.930395
ORM1	0.308157	0.275825	0.609277
AHSG	0.38573	0.166351	0.664306
TTR	0.374984	0.23697	0.656862
ALB	0.450071	0.048997	0.709598
GC	0.292764	0.1831	0.595788
PPBP	0.553503	-0.21967	0.783439
PF4	0.0856	-1.97705	0.335537
CXCL10	0.779828	0.165763	0.907277
TFRC	0.024286	0.508771	0.188496
TF	0.389353	0.131187	0.667643
LTF	0.600007	-0.34577	0.810262
HPX	0.097132	0.270931	0.354489
FTL	0.507389	0.152666	0.75153
FTH1	0.105374	0.350511	0.369374
MT2A;MT1M	0.448077	-0.39182	0.708126
PRH1	0.686212	-0.42739	0.857929
PRB2;PRB1		0	
MT-ND1	0.070708	0.831386	0.310953
MT-ND3	0.364478	0.855656	0.646996
MT-ND4	0.194876	0.948783	0.488911
MT-ND5	0.026002	0.446758	0.193808
MT-ND6	0.05812	1.788047	0.281122
MT-ATP8	0.084549	0.760032	0.335025
ANG	0.17076	0.576196	0.461822
F11	0.332666	0.526964	0.620413
KLKB1	0.769117	0.329715	0.901915
MMP1	0.001487	2.986878	0.039474
SLPI	0.376749	-0.77707	0.658264
HLA-B		0	
C4BPA	0.450097	0.311373	0.709598
VTN	0.354055	0.221663	0.638094
HMGCR	0.44782	0.331547	0.708118
CAT	0.018368	-0.40019	0.163766
RAF1	0.674507	0.515243	0.853175
PLA2G1B	0.988991	0.035036	1
GBA	0.190424	0.222058	0.483515
FUCA1	0.277896	-0.21856	0.579677
PROC	0.047038	1.30572	0.255529
ALDOA	0.490639	0.079158	0.738656
CSTB	0.017726	-0.39389	0.161012
ANXA1	0.2958	0.127069	0.598344
PDGFA	0.139822	0.561111	0.426504
APOB	0.734083	-0.02173	0.882858
NR3C1	0.01301	0.851953	0.139171
TFF1	0.310183	0.387163	0.609864
PRNP	0.210006	0.643762	0.505464
SOD2	0.774166	0.081023	0.903913
LCAT	0.19894	-0.52434	0.49217
OAT	0.105374	0.279524	0.369374

TK1	0.185197	1.16223	0.477569
HRG	0.466082	0.19358	0.721065
THY1	1.80E-08	1.290475	6.25E-06
A1BG	0.371444	0.18774	0.65423
HLA-C	0.933998	-0.05554	0.984157
HLA-DRB1	0.007122	-1.41357	0.101139
CD74	0.181644	-0.33804	0.472575
CD3D	0.979679	0.142347	1
KRT6B	0.074776	0.740516	0.318513
KRT1	0.09982	0.652131	0.359216
S100B	0.45704	0.444017	0.715224
VWF	0.61848	-0.24968	0.822194
SHBG	0.996448	-0.05281	1
SEMG1	0.161733	0.547854	0.453012
TUBB4A	0.057633	1.269459	0.280499
GAPDH	0.734082	0.031173	0.882858
ASL	0.009264	-0.61661	0.116462
IGKV1-16	0.664648	0.123419	0.847351
IGKV1D-39	0.85486	-0.01489	0.946993
IGKV3D-11	0.218354	0.822282	0.515816
HLA-A	0.259649	-1.15871	0.563057
HLA-DPB1	0.056614	-0.59584	0.278081
ERBB2	0.324076	0.284002	0.614309
CAPNS1	0.554955	0.066373	0.783439
TP53	0.019673	1.645471	0.1684
MT1E	0.246385	-1.18185	0.549922
AMY1A	0.885923	-0.16908	0.96416
AMY2A	0.329349	-0.20819	0.619341
HSPB1	0.094502	0.235106	0.349959
CYP1A1	0.594656	-0.08891	0.806337
TYMS	0.414362	0.407497	0.685923
CYBB	0.135171	0.271727	0.417833
RPN1	0.032856	0.229982	0.217297
RPN2	0.008743	0.361186	0.112752
GNAI2	0.197211	0.130056	0.490309
SLC4A2	0.848297	0.062505	0.944084
GYPC	0.04163	-1.92707	0.241862
IGF1	0.319672	-0.30524	0.613139
ATP1A1	0.684992	-0.0239	0.85709
ATP1B1	0.102567	-0.32224	0.364372
CHGB	0.438587	0.339001	0.701933
ALDOB	0.36341	-0.55351	0.646996
APP	0.014008	0.654903	0.144006
ARG1	0.809682	-0.23329	0.921586
APOD	0.241676	0.203835	0.544139
ALDH2	0.422885	-0.1127	0.691467
CYP17A1	0.235307	0.280785	0.537436
ITGB3	0.038324	1.019482	0.234656
ITGB2	0.097132	0.222833	0.354489
S100A8	0.163989	0.581159	0.453453
HMGN1	0.780711	-0.09683	0.908113
SERPINB2	0.411887	-0.30997	0.683152
SERPINE1	0.267754	0.870727	0.570195
SLC25A5	0.897655	-0.02327	0.969793
SERPINA5	0.387089	0.488962	0.666026
SERPING1	0.206534	0.225761	0.500256
CFI	0.153276	0.237627	0.443165
F13B	0.742473	-0.26167	0.888303

ISG15	0.764084	0.182143	0.899242
MPO	0.524435	0.340234	0.764023
PCCA	0.632496	-0.06983	0.830965
PCCB	0.739057	-0.02413	0.886253
ALPL	0.009768	-1.9656	0.119724
ALPP	0.313018	-0.49669	0.611104
EIF2S1	0.694713	0.049142	0.861478
HMGN2	0.345511	0.347233	0.632227
FGF1	0.01027	1.010374	0.122478
ICAM1	0.218632	0.169157	0.515816
RPLP1	0.120332	0.306089	0.39497
RPLP2	0.340521	0.144683	0.627258
RPLP0;RPLP0P6	0.016204	0.174618	0.154611
JUN	0.042311	1.432622	0.243142
FABP3	0.637196	0.460205	0.833511
POLR3D	0.013294	0.867849	0.141215
REG1A	0.594656	-0.17852	0.806337
CLEC3B	0.000815	0.839789	0.027022
SSB	0.060574	0.292951	0.287134
HLA-A	0.845717	-0.05554	0.942239
HLA-DQB2	0.501431	0.522997	0.746793
SERPINA7	0.430639	0.680971	0.695651
SERPIND1	0.305036	0.300389	0.606083
TFAP2A	0.157982	0.726033	0.448701
ITGB1	0.053686	0.225567	0.271145
PRKCB	0.453859	-0.57892	0.712914
KRT18	0.887225	0.144904	0.96416
KRT8	0.913333	0.122322	0.975706
CALB1	0.492913	0.462718	0.741084
MYL1	0.733732	-0.09318	0.882858
HPN	0.292894	0.628279	0.595821
COL5A2	0.000159	1.07991	0.009008
CD1A	0.453758	0.550703	0.712914
CD5	0.494535	-0.73184	0.741997
UROD	0.575023	-0.23659	0.796599
UGT2B4	0.163438	-0.2328	0.453012
INSR	0.001749	0.921112	0.043787
LCK	0.83252	-0.20196	0.93551
FYN	0.688429	0.328465	0.859429
BCHE	0.745437	0.106029	0.888898
GLA	0.007779	0.563244	0.105988
IGKV2-30	0.152843	1.29845	0.443165
IGKV4-1	0.96579	-0.29403	0.99616
IGHV4-34	0.142807	1.297493	0.430424
HLA-DOA	0.103355	-1.17775	0.366609
GSN	0.04253	0.155487	0.243142
RB1	0.303914	0.595255	0.606083
PTMA	0.422885	-0.01779	0.691467
CDK1	0.065669	1.312257	0.299507
ATP5B	0.266378	0.159241	0.568807
C2	0.35064	0.445115	0.635657
S100A9	0.67051	0.124701	0.850681
S100A6	0.918567	-0.06614	0.978657
APOA4	0.38573	0.119355	0.664306
CD2	0.570773	0.406542	0.793891
EIF4E	0.581788	-0.01697	0.797998
CEACAM5	0.424752	0.303304	0.693599
CKM	0.22852	0.685344	0.528484

ENO1	0.830256	0.07853	0.933615
PYGL	0.442223	0.208182	0.703704
GPI	0.72417	0.187574	0.876654
POLB	0.106075	0.709054	0.370631
NPM1	0.18381	0.174026	0.474829
TPM3	0.142204	0.168473	0.428816
ITGAV	2.08E-10	0.967364	3.84E-07
LPL	0.623324	0.470003	0.826059
HEXA	0.934288	-0.03689	0.984157
SERPINE2	0.000502	2.390785	0.020488
EPHX1	0.084549	-0.30938	0.335025
DBI	0.498977	-0.12122	0.744839
LDHB	0.085745	0.331887	0.335537
NEFL	0.976387	0.003926	0.998921
NEFM	0.214668	0.778863	0.510809
CENPB	0.410055	0.768348	0.682809
GPX1	0.882017	-0.06363	0.961515
THBD	0.353207	0.54188	0.638094
PGK2	0.495315	0.326654	0.74257
PROS1	0.374984	0.116885	0.656862
P4HB	0.33719	0.177829	0.624371
H1F0	0.371444	-0.39974	0.65423
ACYP1	0.377803	0.641177	0.659304
FES	0.247021	0.05203	0.549922
CSF1R	0.010931	1.514067	0.127049
CTSD	0.902877	0.036625	0.971583
ANXA2	0.030249	0.256941	0.209418
C8A	0.09193	0.410248	0.345626
C8B	0.153276	0.609406	0.443165
GP1BA	0.954108	0.067562	0.993074
C8G	0.102567	0.757763	0.364372
CAPN1	0.61848	-0.03587	0.822194
TUBB	0.001332	0.440847	0.036119
CA3	0.256769	-1.12298	0.560316
IVL	0.287148	-0.91422	0.590535
PRSS2	0.981503	0.290202	1
ADRB2	0.594656	-0.06511	0.806337
DCN	0.072266	0.360681	0.313701
PSAP	0.923804	-0.01208	0.980751
HEXB	0.272095	0.135633	0.575039
CTSL	0.546147	-0.18248	0.778738
PFN1	0.462056	0.10174	0.718747
BPGM	0.266373	-0.54221	0.568807
APRT	0.078771	-0.26662	0.32578
CD3E	0.748004	-0.24963	0.890902
EPRS	0.651389	0.018128	0.839222
CTSB	0.044589	0.286078	0.24851
HSP90AA1	0.06524	0.250545	0.298136
GALT	0.085603	-0.74617	0.335537
HNRNPC	0.882017	0.01618	0.961515
UQCRH	0.887225	-0.07095	0.96416
LAMB1	0.044589	0.272773	0.24851
YES1	0.694713	0.013232	0.861478
LYN	0.809743	0.023497	0.921586
TPM2	0.000224	0.607529	0.011599
FH	0.981581	-0.03621	1
SFTPB	0.0042	-1.3022	0.073945
ERCC1	0.002199	1.59629	0.05007

THBS1	2.87E-06	2.035878	0.000346
RNASE1	0.613292	-0.17599	0.819264
GJB1	0.502839	-0.4346	0.747596
SP1	0.451722	-0.25479	0.711655
IGF1R	0.702166	0.263588	0.866168
COL1A2	0.244338	0.371899	0.547425
ANXA6	0.088177	0.180327	0.339301
RHOC	0.044577	0.07656	0.24851
NGFR	0.334656	-0.74846	0.62172
CD55	0.357491	0.197592	0.641472
ABCB1	0.769745	-0.17127	0.902552
SERPINA6	0.748889	0.343015	0.890902
SLC3A2	0.017412	0.312718	0.159467
GUSB	0.486499	0.114347	0.73612
PFKM	0.255193	0.144401	0.557593
HSP90AB1	0.090665	0.19197	0.343901
SRPR	0.192667	0.20625	0.486094
ASNS	0.08816	0.932258	0.339301
ELANE	0.33388	-0.71138	0.620902
SYP	0.396507	-0.7574	0.67382
MMP2	2.44E-06	1.368358	0.000298
GSTA1	0.76023	0.142822	0.897659
SOD3	0.613839	-0.3858	0.819264
CTSG	0.112657	-0.57529	0.382523
HMBS	0.524424	-0.17936	0.764023
MME	0.393263	0.964489	0.671013
INHBA	1	-0.10025	1
MGP	0.422605	-0.15552	0.691467
ITGA2B	0.574284	0.532327	0.795974
LPA	0.422846	-0.34926	0.691467
PDHA1	0.072266	-0.21084	0.313701
PLEK	0.490639	-0.18292	0.738656
CD14	0.051278	0.555609	0.265139
COL4A2	0.357491	-0.27056	0.641472
CYC1	0.784286	-0.06749	0.909506
PTPRC	0.699592	0.000597	0.864146
SNRPB2	0.277896	0.191508	0.579677
MET	0.214743	-0.80713	0.510878
MFI2	0.009886	1.737735	0.120352
MYL3	0.944783	-0.26541	0.988547
CFH	0.021148	0.478381	0.173882
SNRNP70	0.604602	0.082374	0.813002
HCK	0.192667	0.455965	0.486094
FCGR3A	0.117214	0.610394	0.389929
ITGA5	0.000172	0.884366	0.009509
NFIC	0.590866	0.011357	0.804875
VIM	0.006009	0.289009	0.090802
SERPINF2	0.595428	-0.00893	0.806337
RPS17	0.173168	0.112252	0.464476
F7	0.696813	-0.19983	0.863213
KRT19	0.882017	0.075804	0.961515
KRT7	0.71923	-0.30967	0.874478
GNAI3	0.201834	0.158569	0.495032
ANXA5	0.181644	0.150326	0.472575
KRT16	0.037785	2.183419	0.233205
STS	0.124902	-1.15928	0.401334
RPSA	0.02267	0.233804	0.180886
ABHD2	0.957231	0.021605	0.994122

ADRA2A	0.763077	-0.15773	0.899242
ROS1	0.401979	-0.42026	0.678038
CD63	0.577274	0.488861	0.796634
MRPL3	0.96843	-0.29481	0.997592
SNRPA	0.651389	0.013816	0.839222
FGF2	0.115504	-1.12554	0.387731
ENO2	0.211314	0.450094	0.506632
HBQ1	0.112919	-0.81451	0.382992
ACAA1	0.415291	-0.14221	0.686028
SRP19	0.694709	-0.06975	0.861478
GSTP1	0.61848	-0.09671	0.822194
CST2	0.657906	-0.3318	0.842898
SNRPC	0.063339	0.237058	0.294055
MMP7	0.571946	0.54557	0.794481
MMP10	0.100431	1.098011	0.360829
CD48	0.997368	-0.00807	1
VIL1	0.113096	1.219477	0.383358
CXCL1	0.563678	-0.19338	0.789479
LGALS1	4.64E-05	0.470036	0.003385
QDPR	0.389353	-0.19902	0.667643
HMGB1;HMGB1P1	0.78936	0.00392	0.911483
RBP1	0.000641	1.262574	0.023555
PAEP	0.481732	-0.35728	0.733586
FBP1	0.360948	-0.26604	0.644411
GNAO1	0.778738	-0.43232	0.906856
SPARC	4.99E-09	1.216937	2.81E-06
GSTM1	0.639403	0.567802	0.834603
TPM1	0.000611	0.472086	0.023051
CLTA	0.986843	-0.26378	1
CLTB	0.637197	0.044325	0.833511
ANXA4	0.426712	-0.07771	0.693599
INHBB	0.547842	0.34685	0.779889
CNP	0.442223	0.112295	0.703704
WNT2	0.420295	0.354994	0.690386
CD7	0.419888	0.551571	0.690026
HMOX1	0.112657	0.373643	0.382523
CSF1	0.30483	0.321041	0.606083
PDGFRB	0.009812	0.644336	0.119724
DLD	0.206534	-0.22546	0.500256
HNRNPA1	0.311298	0.143483	0.609894
SNRPA1	0.45805	0.100551	0.715224
CTSH	0.049723	-0.73253	0.261364
COX6C	0.600007	0.263197	0.810262
CD3G	0.280303	-0.69136	0.583434
TACSTD2	0.236413	-0.21259	0.537858
FGR	0.819807	0.093058	0.92687
C1S	2.46E-05	0.990247	0.001979
PARP1	0.032856	0.25829	0.217297
POLA1	0.471168	0.156631	0.725694
IFI6	1	-0.00758	1
IFIT2	0.9238	-0.05476	0.980751
IFIT1	0.537409	0.212376	0.773175
ALOX5	0.153276	0.245707	0.443165
UCHL1	0.001215	1.296928	0.034268
FURIN	0.823669	0.157159	0.930182
LTA4H	0.26355	-0.22706	0.565873
ALDOC	0.231232	0.267819	0.531962
C4A	0.595428	0.139785	0.806337

C4B	0.149515	0.27977	0.438811
TFPT	0.966947	-0.07206	0.996989
FBXO45	0.465993	0.464181	0.721065
H2AFB2;H2AFB1	0.322009	0.22017	0.613139
CISD3	0.468067	-0.45329	0.722799
ATXN1L	0.394605	0.271402	0.672349
JMJD7	0.048522	-0.60157	0.258731
MYZAP	0.030982	-1.35852	0.212639
POLR2M	0.902584	-0.10897	0.971583
GPR89B;GPR89A	0.404052	0.188297	0.678271
CHTF8	0.877866	-0.11138	0.95982
GSTT2B;GSTT2	0.938609	-0.31474	0.986498
TMSB15B;TMSB15A	0.733732	-0.14258	0.882858
POTEI	0.882917	-0.24216	0.962401
ZNF865	0.207345	0.444987	0.501941
ZNF888	0.750672	-0.14064	0.892487
SLC35E2B	0.215007	0.630437	0.511286
LIMS3L;LIMS3	0.473185	-0.52751	0.727018
TRAPPC2B;TRAPPC2	0.210533	1.292618	0.506604
RAB34	0.115283	0.705246	0.387731
PET100	0.228985	-0.88657	0.529099
SMIM13	0.086478	0.69529	0.337307
RGPD1;RGPD2	0.965711	-0.05628	0.99616
RBMY1A1;RBMY1C;RBMY1F	0.393617	0.205625	0.671013
PGA5;PGA3;PGA4	0.57907	-0.24156	0.796675
SAA1	0.516723	0.590005	0.758769
SAA2	0.421207	-0.31784	0.691066
SULT1A4;SULT1A3	0.613784	-0.48873	0.819264
HSPA1B;HSPA1A	0.627809	-0.05025	0.828932
SMIM10L2B;SMIM10L2A	0.040567	0.996074	0.240216
U2AF1	0.006131	0.337664	0.092018
CBS	0.074811	0.735322	0.318513
IGLC2	0.537408	0.161216	0.773175
IGLC3	0.011148	-1.14822	0.128261
IGHV1-8	0.534225	0.354547	0.771496
IGHV3-30-3	0.322009	0.158767	0.613139
IGHV3-30-5;IGHV3-30	0.151386	0.162087	0.44125
IGHV4-38-2	0.044351	0.850568	0.24851
IGKV1D-13	0.008027	2.072612	0.107782
CALM3;CALM2;CALM1	0.162993	-0.12163	0.453012
SLURP2	0.041397	0.762854	0.240826
POLR1D	0.656074	0.135126	0.84209
EEF1AKMT4	0.85486	-0.13518	0.946993
GATD3A;GATD3B	0.235109	-0.10533	0.537205
N/A	0.556057	0.226453	0.783439
NOTCH2NL	0.496019	0.279475	0.743024
GLI3	1	-0.02479	1
FDX1	0.340521	-0.30835	0.627258
RAP2A	0.473924	0.255589	0.727018
SRGN	0.351988	0.672494	0.637267
GZMB	0.664325	0.083369	0.847289
CXCL8	0.22225	1.229512	0.520592
RNASE2	0.679345	-1.01135	0.85545
TROVE2	0.266378	0.159129	0.568807
COX8A	0.647153	-0.31815	0.837719
GAA	0.211314	0.2826	0.506632
RARA	0.901067	0.102632	0.971583
RRAS	0.97106	0.026348	0.997592

HLA-C	0.466082	-0.14607	0.721065
ARAF	0.162993	0.282426	0.453012
HIST1H1E	0.03257	-1.09944	0.217083
BCL2	0.585715	0.238195	0.801141
SPP1	0.02431	2.098929	0.188548
DLAT	0.149515	-0.14445	0.438811
PTPRF	0.444182	-0.33683	0.705893
NR2F6	0.787276	0.205561	0.911483
NR2F1	0.04183	1.036214	0.242066
TXN	0.784286	0.069569	0.909506
TGFB3	0.003764	1.566715	0.07038
COX5B	0.343873	-0.02731	0.630156
CTSA	0.077655	0.257619	0.324641
MGST1	0.272095	-0.22761	0.575039
MAPT	0.860773	-0.02649	0.950165
C7	0.507389	0.069662	0.75153
PRKAR1A	0.550543	0.070043	0.781327
CHGA	0.331588	-0.12323	0.619341
TFPI	0.212513	-0.31794	0.508737
ALPPL2	0.188366	-1.1253	0.48038
PF4V1	0.331588	-0.14777	0.619341
KIT	0.106848	-1.08416	0.371691
UROS	0.058851	1.869168	0.28342
ESD	0.277896	-0.21552	0.579677
HSPD1	0.118765	-0.16356	0.392409
CLU	0.819984	0.004633	0.92687
HAPLN1	0.058284	1.485665	0.281667
MYL2	0.638802	0.181579	0.834211
HSPA5	0.266378	0.121503	0.568807
LAMC1	0.550543	-0.18002	0.781327
CD37	0.365931	0.86832	0.648976
ACP2	0.026002	0.291353	0.193808
MAP2	0.248149	0.792973	0.551436
HSPA8	0.013012	0.18609	0.139171
SLC2A1	0.11117	0.664859	0.380668
SLC2A3	0.007751	2.183189	0.105988
EPB41	0.026898	-0.44071	0.198225
UMPS	0.043893	0.408009	0.247494
PDHB	0.902877	0.002283	0.971583
DBT	0.415291	-0.16526	0.686028
ITGAM	0.071226	0.283322	0.311134
PYGB	0.010002	-0.44104	0.120967
PYGM	0.013385	-1.61005	0.141515
MBL2	0.683128	0.144297	0.85709
RALA	0.213733	0.243525	0.509228
RALB	0.023065	0.270287	0.182986
BCR	0.037126	0.381694	0.23107
SPTB	0.094502	-0.67008	0.349959
LAMP1	0.675324	0.07712	0.853175
ERG	0.860676	0.089534	0.950165
ACADM	0.327323	0.0814	0.617719
FGFR1	0.124128	0.507149	0.400153
TOP1	0.450097	-0.02096	0.709598
TOP2A	0.008618	1.530505	0.111658
G6PD	0.744041	0.073781	0.888303
UBL4A	0.482377	-0.07648	0.733586
VDR	0.180494	0.716414	0.472575
ESRRA	0.072467	0.852949	0.314326

PC	0.26355	0.176407	0.565873
DMD	0.789359	0.296334	0.911483
MTHFD1	0.490639	0.163975	0.738656
CETP	0.40269	-0.23359	0.678038
EPX	0.655653	-0.16736	0.84209
SCGB1A1	0.924899	-0.03796	0.981632
SFTPC	0.007461	-2.92431	0.10395
IGF2R	0.11568	0.417831	0.387731
ADH5	0.78936	-0.07288	0.911483
CDK4	0.407773	0.375829	0.680612
SRF	0.196915	0.434973	0.490309
MS4A1	0.976777	-0.02496	0.999117
PRPS2	0.902877	0.009807	0.971583
CD79A	0.996024	-0.0601	1
ODC1	1	-0.12759	1
PABPC1	0.026898	0.159198	0.198225
PCNA	0.35064	0.239811	0.635657
KRT3	0.74145	0.037488	0.887483
NEFH	0.105925	-0.38926	0.370605
COX6A1	0.819957	0.540619	0.92687
HARS	0.058787	0.242434	0.283235
COL11A1	0.000131	2.668886	0.007794
COL6A1	0.887225	-0.02567	0.96416
COL6A2	0.992106	-0.05297	1
COL6A3	0.407778	0.091854	0.680612
SLC25A4	0.108241	-0.31697	0.374055
SLC25A6	0.554955	0.080504	0.783439
F5	0.925711	-0.23323	0.982024
IMPDH2	0.06524	0.21932	0.298136
TPR	0.145824	0.230699	0.434185
PTHLH	0.163438	-0.33213	0.453012
PIP	0.904949	-0.1822	0.972966
CKB	0.108241	0.603666	0.374055
FCGR1A	0.038035	0.70487	0.233324
FCGR2A	0.277869	0.54662	0.579677
ANXA3	0.001057	-1.1016	0.031507
MYCLP1	0.163438	-0.23414	0.453012
CKMT1A	0.47351	-0.41204	0.727018
GZMA	0.150297	-1.18719	0.440175
BMP2	1	-0.11578	1
BMP3	0.801334	-0.24102	0.91752
BCKDHA	0.252448	-0.20213	0.555643
RNASE3	0.252339	0.944299	0.555643
SKI	0.670768	-0.03205	0.850681
ACTN1	1.43E-08	0.712722	6.25E-06
ACE	0.515876	-0.28734	0.757825
MYL4	0.64451	0.253955	0.837353
CDH1	0.581788	-0.2646	0.797998
DEFA4	0.599931	-0.38579	0.810262
MYH1	0.751942	-0.02973	0.893287
MYH7	0.60099	0.28725	0.811292
SRC	0.660922	0.150213	0.844596
PEPD	0.015914	0.413064	0.153751
XRCC6	0.155183	0.103999	0.444958
XRCC5	0.043893	0.202204	0.247494
UNG	0.104302	1.278001	0.367825
COX4I1	0.515876	-0.09855	0.757825
ALAS1	0.622941	0.400853	0.825978

GP1BB	0.954108	0.042621	0.993074
IFI30	0.656149	-0.14057	0.84209
LAMP2	0.378546	0.229601	0.659304
RNH1	0.929044	-0.03242	0.983115
BMP1	1.00E-05	2.514567	0.000968
CYBA	0.981581	-0.19644	1
CCL2	0.169846	-0.62285	0.461029
CCL5	0.731297	0.14741	0.881419
SCG2	0.145176	0.486013	0.433769
CFTR	0.488913	-0.40837	0.738656
CYP4B1	0.112205	-1.33894	0.382523
NCAM1	0.285971	-0.70196	0.590053
ICAM2	0.975991	-0.02845	0.998921
VCAN	4.26E-09	1.359462	2.62E-06
ITGA4	0.208913	0.316914	0.503054
ATP1A3	0.986749	-0.05211	1
EEF2	0.013502	0.281281	0.141812
KRT10	0.130075	0.749868	0.410899
KRT13	0.445494	0.532401	0.706795
KRT5	0.814855	0.328447	0.924847
PDIA4	0.102567	0.207354	0.364372
C6	0.155183	0.376027	0.444958
P4HA1	1.73E-05	0.850958	0.001521
ZNF35	0.813008	-0.15928	0.924544
ACP5	0.736568	0.037918	0.884985
CEACAM1	0.145421	0.818418	0.434185
TPT1	0.462056	0.051256	0.718747
ALAD	0.825116	-0.07714	0.930395
F3	0.695601	0.299619	0.862317
PRG2	0.804484	-0.27522	0.918848
HLA-A	0.71452	-0.33401	0.871808
HLA-E	0.830256	-0.12455	0.933615
HLA-DRB1	0.258457	-1.27379	0.560954
HLA-DRB1	0.334021	-1.19771	0.620956
HLA-DRB4	0.304151	-1.21289	0.606083
HLA-DOB	0.195154	-0.80407	0.489252
LCP1	0.609213	0.126842	0.816529
PLS3	0.003027	0.490489	0.062294
APEH	0.407778	-0.09152	0.680612
ETFA	0.835403	0.020804	0.935999
GYS1	0.2958	0.108495	0.598344
PRKAR2A	0.396661	-0.12056	0.67382
SLC5A1	0.163707	0.721693	0.453012
ANXA8	0.222954	0.828217	0.521792
ENO3	0.990163	-0.05667	1
GTF2F2	0.136904	0.496556	0.421544
CD59	0.590866	-0.07264	0.804875
CCDC130	0.188151	0.377308	0.480067
MTHFD2	0.009994	1.468539	0.120967
CTSE	0.324546	-0.91656	0.615095
SELL	0.959835	-0.05003	0.994122
MIF	0.934288	0.028631	0.984157
FOLR2	0.699592	-0.31996	0.864146
CD99	0.016797	1.393602	0.156698
HGF	0.158441	0.713485	0.449659
PRF1	0.475075	-0.56803	0.728131
PRKCSH	0.162993	0.213912	0.453012
IRF2	0.959194	0.020453	0.994122

HCLS1	0.055344	0.404008	0.274516
FDPS	0.09982	0.203767	0.359216
TRIM27	0.955268	0.077381	0.993074
CPM	0.704483	-0.10357	0.867291
COX7A2	0.72417	0.026985	0.876654
ATP1B2	0.019466	-1.18268	0.16752
NID1	0.169043	-0.38093	0.459676
AKR1A1	0.960544	0.026172	0.994122
PLA2G2A	0.197664	1.141301	0.491215
NCF1	0.073319	0.592534	0.31592
PKM	0.244338	0.225781	0.547425
ACYP2	0.026493	-0.9366	0.196153
HSP90B1	0.067188	0.19248	0.302058
CCNB1	0.861345	-0.11723	0.950187
MYL6B	0.175259	-0.23762	0.466796
SLC2A4	0.016508	-0.99571	0.155558
IDE	0.164991	0.234196	0.454525
GP9	0.310183	-0.36864	0.609864
IL1R1	0.34232	0.529702	0.629735
MMP9	0.03339	1.100072	0.219585
COX6B1	0.76912	0.150248	0.901915
POU2F1	0.779188	0.15415	0.906856
HNRNPL	0.343873	0.134178	0.630156
DARS	0.764084	0.027673	0.899242
IDO1	0.940597	-0.05904	0.987239
ETS1	0.008494	1.593388	0.110825
JUP	0.2958	0.128477	0.598344
UQCRB	0.013012	-0.35386	0.139171
LIF		0	
ETS2	0.322009	0.14657	0.613139
BRAF	0.36861	-0.52244	0.651515
CPB1	0.57907	-0.14266	0.796675
CPA3	0.064735	-1.19299	0.297421
FABP4	0.185033	-0.98156	0.477255
GLUL	0.651389	-0.01894	0.839222
AKR1B1	0.055344	0.262865	0.274516
ANPEP	0.004905	0.888604	0.082133
PVR	0.257801	0.886354	0.560462
RAC2	0.494799	0.004054	0.741997
CPN1	0.11703	0.966398	0.389929
GSPT1	0.586319	-0.09618	0.801141
PGAM2	1	-0.09023	1
IFNGR1	0.991882	-0.13499	1
ARSA	0.400346	-0.24408	0.67622
B4GALT1	0.020774	1.142851	0.172048
ACPP	0.976246	0.157397	0.998921
EZR	0.084549	-0.28782	0.335025
ATP6V1B1	0.632486	-0.2853	0.830965
FOLR1	0.484237	-0.39939	0.735105
ATF2	0.431857	-0.26413	0.69689
UCHL3	0.675324	0.124685	0.853175
CD19	0.219226	0.524014	0.516997
FOSL1	0.601315	0.333056	0.811336
FOSL2	0.038135	1.319422	0.233804
HPGD	0.068934	-1.68316	0.306571
VAV1	0.090665	0.267128	0.343901
ELN	0.370966	0.781927	0.65423
CSF2RA	0.405645	0.525102	0.679973

AREG	0.632486	-0.2543	0.830965
CD46	0.511623	0.028171	0.754376
NME1	0.027821	0.387965	0.202072
NQO1	0.72202	-0.35673	0.876654
GNS	0.689846	-0.12072	0.859429
VEGFA	0.282684	0.880682	0.585849
PHKG2	0.784254	0.471362	0.909506
IGLL1	0.677737	-0.23904	0.854568
ARSB	0.000487	1.534509	0.020212
RPS2	0.637197	-0.05364	0.833511
CHN1	0.259792	0.536071	0.563204
ST6GAL1	0.263545	0.30569	0.565873
DSP	0.709387	0.301606	0.869179
RPA2	0.33388	0.09392	0.620902
MUC1	0.704483	-0.07628	0.867291
COX7C	0.205836	-0.77667	0.500256
TIMP2	0.000177	1.785017	0.009596
ALOX15	0.961061	-0.10859	0.99427
NPR1	0.771215	0.150087	0.903336
CD44	0.26355	0.116306	0.565873
NQO2	0.230765	-0.72047	0.531771
H2AFX	0.53908	-0.81833	0.774975
SELP	0.792554	0.176552	0.913596
ACAN	0.174607	1.036503	0.466796
ITGB4	0.103963	-0.53866	0.366863
SPN	0.699289	-0.39407	0.864146
CBR1	0.419078	-0.16216	0.688796
ANK1	0.112657	-0.80709	0.382523
HLA-A	0.708594	0.282262	0.869179
HLA-A	0.004571	2.59254	0.078202
HLA-A	0.322009	0.139647	0.613139
ACADS	0.280828	-0.26336	0.583434
CREB1	0.827685	0.107023	0.932533
PDGFRA	0.000946	2.067907	0.029388
SLC25A16	0.970732	0.374443	0.997592
GLB1	0.017412	0.339609	0.159467
PECAM1	0.646644	-0.19405	0.837353
PPP3CB	0.117194	0.656615	0.389929
NCK1	0.010791	0.408547	0.126291
GCFC2	0.123336	1.001472	0.399387
KCNA2	0.52703	-0.27243	0.766596
HIST1H1B	0.486499	0.106859	0.73612
HIST1H1D	0.311297	-0.20183	0.609894
HIST1H1C	0.194929	-0.5497	0.488911
EPCAM	0.426712	-0.28867	0.693599
POR	0.613839	-0.1073	0.819264
ABO	0.963521	0.012178	0.995937
DPEP1	0.55076	0.751923	0.781335
EPB42	0.197108	-0.64105	0.490309
MGMT	0.047465	0.298615	0.255923
FER	0.003319	1.58745	0.065611
ATP2A2	0.028771	0.25057	0.204946
CD36	0.177302	-0.80897	0.468602
CPE	0.749559	0.37794	0.891418
IL7R	0.651513	0.0679	0.839222
PLCG2	0.537409	0.250935	0.773175
FAH	0.199513	-0.30125	0.49217
STMN1	0.060574	0.410438	0.287134

YBX3	0.006382	0.716563	0.094501
ZFX	0.33939	0.444368	0.626528
ZNF22	0.142182	-0.89784	0.428816
ZNF24	0.723762	0.17468	0.876654
ZKSCAN1	0.018918	1.508742	0.166597
ZNF3	0.96804	0.020592	0.997541
NAGA	0.04896	0.602666	0.259694
HSPA6	0.188132	-1.07536	0.480067
RHOQ	0.116614	0.813312	0.38945
HMGA1	0.077655	0.545561	0.324641
ZNF8	0.614841	-0.04307	0.820298
TMEM11	0.646635	-0.07481	0.837353
GOT1	0.709387	0.219362	0.869179
IFNAR1	0.05424	0.692982	0.273313
BPI	0.322509	0.68752	0.613498
PRKCA	0.646644	0.101285	0.837353
JUNB	0.086532	1.221404	0.337307
ITGA2	0.076552	0.372301	0.322705
GJA1	3.76E-07	3.214824	6.96E-05
NPR3	0.005445	2.092178	0.086993
SMPD1	0.9866	-0.07689	1
UBTF	0.44615	0.095332	0.706795
AKR1C4	1	-0.05265	1
JUND	0.462026	-0.1248	0.718747
CKMT2	0.520746	-0.62586	0.761048
ATF7	0.345818	0.542878	0.632498
NDUFB7	0.354042	-0.31123	0.638094
PRKACA	0.546147	-0.08531	0.778738
CAPN2	0.784286	0.01363	0.909506
DES	0.199508	-0.65564	0.49217
CEBPB	0.192599	0.721324	0.486094
GAP43	0.043559	1.448157	0.247494
HLA-G	0.576975	0.20052	0.796634
PTPN2	0.724167	-0.01827	0.876654
CTPS1	0.934288	0.080678	0.984157
ENG	0.374984	-0.10831	0.656862
DDX5	0.032856	0.239663	0.217297
PFKL	0.164991	0.173744	0.454525
GM2A	0.434427	-0.01555	0.698194
CR1	0.795534	0.11792	0.914405
LGALS3	0.012773	-0.40499	0.137944
IGFBP3	0.000586	2.224356	0.022558
SPI1	0.808416	0.160378	0.921586
FLT1	0.408836	0.404781	0.681762
PSMC3	0.069183	0.203942	0.306571
TCP1	0.76912	0.038305	0.901915
PTPN1	0.301936	0.183293	0.603275
ALOX12	0.312494	0.267334	0.610732
IGFBP2	0.627465	0.505677	0.828932
ERCC2	0.011635	0.632928	0.129699
RPL35A	0.684992	-0.23277	0.85709
ITGB5	4.93E-06	0.750068	0.000552
ARF4	0.450097	0.124294	0.709598
RPL7	0.913333	-0.10291	0.975706
EGR1	0.992139	0.052148	1
VCL	0.039276	0.233313	0.236232
GPX2	0.73657	-0.42443	0.884985
LBP	0.04253	0.674358	0.243142

PTPRA	0.830256	-0.0966	0.933615
NAT1	0.00094	0.799635	0.02929
HLA-B	0.967597	-0.12443	0.997177
IL1RN	0.069387	1.764335	0.307215
ITGB6	0.12513	0.417237	0.401334
RHCE	0.347401	-0.70815	0.632785
SON	0.069183	0.357454	0.306571
NELFE	0.396553	-0.16915	0.67382
RPL17	0.422885	0.061778	0.691467
LAG3	0.711083	-0.11022	0.870136
PGAM1	0.247021	0.195055	0.549922
RCC1	0.434427	-0.22458	0.698194
SDC1	0.118764	0.666991	0.392409
ATF1	0.393617	0.294674	0.671013
ATF6	0.017606	0.713668	0.160979
LIG1	0.177273	0.556118	0.468602
ATP5J	0.646644	-0.07859	0.837353
XRCC1	0.276436	0.224442	0.579677
KRT15	0.511583	0.362187	0.754376
KRT4	0.822286	-0.1158	0.929187
PAM	0.042724	0.793844	0.244126
CDH2	0.198062	0.606765	0.491653
TSPAN8	0.036027	-1.68115	0.227287
GNAZ	0.657906	-0.24876	0.842898
MYL12A	0.331588	-0.14424	0.619341
PLCG1	0.003297	0.844846	0.065524
UGT1A6	0.58062	0.176933	0.797998
CD58	0.941876	0.437058	0.988208
VCAM1	5.07E-09	2.456223	2.81E-06
NCL	0.825116	0.023298	0.930395
HK1	0.422885	0.142263	0.691467
POLR2C	0.09193	0.269216	0.345626
POLR2E	0.627809	-0.09843	0.828932
NDUFV2	0.572777	-0.11041	0.794481
TNFRSF1A	0.513135	0.290592	0.75581
GGT1	0.563704	-0.64489	0.789479
ERCC3	0.554917	0.054914	0.783439
TRIM21	0.892438	0.030339	0.966699
TFEB	0.210743	0.863825	0.506632
EIF2AK2	0.027356	0.309685	0.199874
TFE3	0.53548	0.352295	0.772704
SRM	0.063339	0.79289	0.294055
SLC9A1	0.131753	0.571564	0.413476
ORM2	0.011419	0.814964	0.128971
CSNK2A2	0.006009	0.340811	0.090802
RXRA	0.739804	-0.09427	0.886374
AOC1	0.909519	0.098093	0.974541
ITIH2	0.192667	0.261798	0.486094
ITIH1	0.055344	0.55751	0.274516
CEL	0.711083	-0.11522	0.870136
NFKB1	0.825116	-0.00171	0.930395
CXCL2	0.163438	-0.34129	0.453012
NCF2	0.171096	0.336729	0.461822
FST	0.09909	0.441736	0.358568
PI3	0.571349	0.16493	0.793891
TYMP	0.367925	0.126575	0.650718
ATP2B1	0.835403	0.076659	0.935999
CR2	0.349041	-0.33481	0.635145

HLA-DPA1	0.490639	-0.2838	0.738656
HLA-DRB1	0.354055	-0.45748	0.638094
EIF2S2	0.257958	0.173831	0.560462
TCN1	0.953347	-0.24935	0.993074
TCN2	0.027053	1.304905	0.198967
ANXA7	0.478274	0.049843	0.730754
SDS	0.072924	1.454421	0.315444
CD33	0.152875	0.993597	0.443165
PGC	0.066898	-1.90728	0.302058
SPINK2	0.988991	0.010489	1
AZU1	0.952602	0.245634	0.993074
TBP	0.889539	-0.75493	0.966106
TPSB2;TPSAB1	0.364426	-0.38494	0.646996
CCNA2	0.556057	0.083468	0.783439
POU3F2	0.384917	0.407802	0.664306
CD22	0.336183	0.358271	0.623617
BTF3	0.252448	0.250376	0.555643
ALOX5AP	0.364413	0.162854	0.646996
TNFRSF1B	0.370989	-0.3019	0.65423
RAB3A	0.380367	-0.61306	0.661698
RAB3B	0.001647	1.814393	0.041898
RAB4A	0.135171	0.283078	0.417833
RAB5A	0.604602	0.069895	0.813002
RAB6A	0.094502	0.227692	0.349959
PVALB	0.99433	-0.08193	1
MSH3	0.944775	0.063171	0.988547
MX1	0.71923	0.150959	0.874478
MX2	0.378546	0.38341	0.659304
NPR2	0.019784	0.738	0.1684
PSMB1	0.454064	0.164581	0.712914
M6PR	0.613839	0.118411	0.819264
COX5A	0.498977	0.063875	0.744839
LMNB1	0.09581	0.214086	0.352331
ITGAL	0.563832	-0.25332	0.789479
ITGAX	0.171096	0.378373	0.461822
DDC	0.012197	1.766374	0.134208
GZMH	0.48945	-0.42329	0.738656
HOXA5	0.2683	0.445027	0.570935
PZP	0.661409	0.14708	0.844736
BCL3	0.429721	0.436609	0.695651
OGN	0.997369	0.089868	1
CAST	0.204174	0.319898	0.497963
CYP3A5	0.167504	-0.75577	0.457358
HNF1A	0.651513	0.134929	0.839222
EFNA1	0.15375	-1.06585	0.444304
IMPDH1	0.286754	0.165608	0.590053
COL9A1	0.571349	0.020508	0.793891
C4BPB	0.311006	1.090194	0.609894
COL5A1	3.59E-06	1.403138	0.000424
FLG	0.190786	0.286389	0.484103
AGA	0.92904	0.18345	0.983115
RASA1	0.046368	0.456039	0.253584
PTMS	0.317644	0.683042	0.613139
CD247	0.131823	1.031056	0.413476
FUT3	0.090007	1.390862	0.343044
PTN	0.810266	-0.12914	0.921889
GSTM3	0.819984	0.301387	0.92687
ATP6V1B2	0.71923	0.061524	0.874478

ATP6V1C1	0.027821	0.267143	0.202072
CSRP1	0.023466	0.39102	0.184845
FLNA	2.32E-05	0.493855	0.001908
NF1	0.003228	0.711668	0.065092
MAOA	0.000412	-1.17614	0.018066
ACO1	0.651389	0.124695	0.839222
S1PR1	0.40269	0.197368	0.678038
FPR1	0.062548	1.237302	0.292586
SYT1	0.632486	-0.16139	0.830965
TNFAIP3	0.708254	-0.22112	0.869179
KITLG	0.781668	-0.16166	0.908746
NT5E	0.478274	0.183618	0.730754
TAF1	0.427818	0.356993	0.69482
GPD1	0.153234	-1.25158	0.443165
EPHA1	0.289583	0.454201	0.593134
C5AR1	0.035269	1.790367	0.224243
MDK	0.482372	0.369701	0.733586
MSR1	0.00069	1.054059	0.024536
VDAC1	0.0334	0.298787	0.219585
BGN	0.185996	0.2532	0.478129
CD72	0.771591	0.123036	0.903336
ERBB3	0.723742	-0.10996	0.876654
SDHB	0.918567	0.013022	0.978657
CD9	0.637197	0.150716	0.833511
MATN1	0.40269	0.221891	0.678038
BCKDHB	0.093209	-0.32547	0.347256
COMT	0.627809	0.054726	0.828932
TGM2	0.835403	0.064029	0.935999
BMP5	0.853939	0.087918	0.946993
MUT	0.944783	-7.38E-05	0.988547
OSBP	0.665709	0.058115	0.847351
PCMT1	0.856059	-0.02824	0.946993
FUT4	0.995277	-0.09121	1
FBL	0.72417	0.009496	0.876654
RPS4Y1	0.826337	-0.14639	0.93133
GART	0.12841	0.100011	0.407846
TNXB	0.011419	-1.16529	0.128971
CDH3	0.028235	1.453956	0.203235
PAICS	0.266378	0.226004	0.568807
ACHE	0.517148	0.268151	0.75923
IDS	0.94367	0.165646	0.988547
SCP2	0.33388	-0.17789	0.620902
UBA1	0.804634	0.077682	0.918848
GPX3	0.046732	0.442441	0.254194
NME2	0.049723	0.261783	0.261364
ENPP1	3.95E-07	3.984021	7.07E-05
USF1	0.804204	0.396911	0.918848
SPRR1B	0.339213	-0.33889	0.626409
FDXR	0.404052	-0.2441	0.678271
FGFR3	0.756615	-0.05785	0.896333
PRKACG	0.54223	0.437142	0.775607
HNRNPA2B1	0.939534	0.005344	0.986498
RFX1	0.402151	0.382587	0.678038
CALB2	0.196229	1.065215	0.490309
CBL	0.201809	0.547168	0.495032
IGFBP4	0.118709	0.823648	0.392409
PRKACB	0.404026	0.129985	0.678271
UQCRC2	0.856059	0.034127	0.946993

SLC2A5	0.046434	1.391696	0.253695
TGM1	0.041397	0.373714	0.240826
NR4A1	0.329349	-0.1921	0.619341
CA4	0.023824	-2.17873	0.186463
GNLY	0.901067	-0.3554	0.971583
AADAC	0.104774	-0.85808	0.368787
CPN2	0.194194	1.016689	0.488835
FECH	0.330591	-0.11699	0.619341
PROZ	0.444016	0.363933	0.705729
MMP8	0.283267	0.848096	0.586688
MRC1	0.411524	0.100094	0.683152
XPA	0.445334	0.433104	0.706795
IGHV1-2	0.22215	0.448483	0.520592
CES1	0.684992	0.24245	0.85709
FBLN1	6.25E-07	0.996387	0.000103
TCEA1	0.950035	0.070179	0.990863
PTGS1	0.022281	0.498281	0.179329
ITGA6	0.211314	-0.3505	0.506632
SFPQ	0.241676	0.149212	0.544139
TUBG1	0.006255	0.318791	0.093377
KEL	0.329349	-0.16402	0.619341
PPIB	0.06524	0.223923	0.298136
S100A1	0.283495	-0.72464	0.586874
HRC	0.338364	-0.28838	0.62557
KAL1	0.383013	-0.47204	0.662548
ME2	0.208914	0.164535	0.503054
GLDC	0.664073	0.307719	0.847289
WARS	0.641914	-0.02355	0.835764
RPS3	0.252448	0.094178	0.555643
GCSH	0.078768	0.232956	0.32578
RPS6KB1	0.018342	1.057091	0.163766
JAK1	0.103963	0.345608	0.366863
PTPRB	0.131823	0.522942	0.413476
PTPRD	0.547409	0.341218	0.779573
PTPRE	0.389353	-0.22979	0.667643
PTPRG	0.558151	-0.00903	0.785989
PTPRZ1	0.676056	0.260589	0.853516
SP100	0.415291	-0.22257	0.686028
MCC	0.05225	1.092362	0.26792
TNFSF4	0.730466	0.155594	0.880704
NFYA	0.386701	0.562044	0.665564
AHCY	0.794443	0.04201	0.913708
HIST1H2BO	0.272021	0.930051	0.575039
CFL1	0.292763	0.065558	0.595788
EIF4B	0.26355	0.148655	0.565873
F8A1	0.03232	0.491353	0.215938
ATP2B4	0.609213	0.078877	0.816529
ITPKA	0.118152	0.626571	0.392409
DGKA	0.000207	0.724562	0.010881
CPT2	0.171096	-0.20907	0.461822
DTYMK	0.604602	-0.11164	0.813002
RRM1	0.026397	1.121848	0.195939
CMA1	0.093822	-0.91727	0.349072
LAMA2	0.430555	-0.56309	0.695651
FCAR	0.531576	0.306262	0.770378
PRTN3	0.637197	0.039137	0.833511
COX7A1	0.089309	0.92355	0.341477
MMP11	1.13E-07	4.470569	2.62E-05

CCND1	0.53317	-0.24728	0.7709
CHM	0.036734	1.319767	0.229759
KDELR1	0.228659	0.56756	0.528484
IL4R	0.167109	0.24516	0.457139
CYP3A7	0.331588	-0.13949	0.619341
NR2F2	0.165495	0.918695	0.455554
EDNRB	0.08528	-0.45972	0.335537
EEF1B2	0.09193	0.203558	0.345626
ATP5F1	0.876815	0.041311	0.958859
TBXAS1	0.030249	0.45259	0.209418
AKAP5	0.659827	0.22841	0.844596
IGFBP6	0.011985	1.617729	0.13253
IGFBP5	0.001043	2.063795	0.031434
ACP1	0.97106	-0.10637	0.997592
PRKCH	0.562382	-0.05242	0.789479
ACAT1	0.000101	-0.62654	0.006359
TNC	0.00094	0.998948	0.02929
MYL9	0.001243	0.607276	0.034693
DNASE1	0.571349	0.002332	0.793891
CCNC	0.039166	1.52871	0.236232
POLR2A	0.025131	0.360841	0.191949
CDK2	0.05702	0.856538	0.278658
CXCR2	0.49063	-0.26441	0.738656
APC	0.19838	0.736623	0.49217
COL8A2	0.000508	2.81996	0.02064
FPR3	0.34386	0.67542	0.630156
FPR2	0.976387	-0.05924	0.998921
ADRBK1	0.2958	0.071034	0.598344
EDNRA	0.281331	0.441188	0.583877
PTAFR	0.671419	0.254778	0.851247
ACKR3	0.594656	-0.02896	0.806337
F2R	0.04021	1.328323	0.239722
MPZ	0.447034	0.290183	0.707261
MCM3	0.002838	1.017412	0.060074
NFYB	0.905076	-0.64923	0.972966
AZGP1	0.646644	-0.16581	0.837353
MPST	0.913333	0.106	0.975706
LAMA1	0.72009	-0.23988	0.875395
RPS12	0.145824	0.131965	0.434185
BRD2	0.190156	0.506942	0.483515
FAS	0.992106	-0.42972	1
YY1	0.149515	0.308942	0.438811
DNAJB1	0.249724	0.141781	0.552833
DNAJB2	0.43249	-0.14819	0.697404
ATP5A1	0.923804	0.036027	0.980751
CTSS	0.76912	0.06574	0.901915
PSMA1	0.581788	0.009852	0.797998
PSMA2	0.33719	0.103224	0.624371
PSMA3	0.050495	0.246079	0.263221
PSMA4	0.908103	0.063245	0.9734
S100P	0.884477	-0.0243	0.963599
COL5A3	0.05583	1.192014	0.276127
CD40	0.166967	0.660244	0.457139
NFKBIA	0.628642	0.322481	0.829728
ITGA3	0.071226	-0.50778	0.311134
ITGB7	0.915881	0.067232	0.977664
ITGB8	0.055226	1.009259	0.274516
PTX3	0.138795	-0.78257	0.424305

MSN	0.213733	-0.12973	0.509228
PTPN3	0.624796	0.337147	0.827713
DDX6	0.058787	0.236667	0.283235
CTNNA2	0.744977	-0.31476	0.888646
DNMT1	0.112657	0.723532	0.382523
U2AF2	0.04253	0.246626	0.243142
RPL13	0.923804	-0.02047	0.980751
CHML	0.414056	0.251333	0.685923
IVD	0.00344	-0.52351	0.067417
S100A4	0.031269	0.312408	0.212999
MGAT1	0.017726	0.420118	0.161012
HMGB2	0.819984	0.074538	0.92687
PTBP1	0.036237	0.195716	0.227448
TARS	0.044589	0.389402	0.24851
VARS	0.840557	-0.02974	0.939212
EEF1G	0.382127	0.083293	0.661698
ZFP36	0.312494	0.359202	0.610732
PLN	0.137489	0.790296	0.422524
CD27	0.349723	0.6115	0.635657
FKBP2	0.26355	-0.1741	0.565873
MST1	0.041032	1.425374	0.240826
IL3RA	0.568515	-0.28734	0.791838
CNTFR	0.549913	-0.29698	0.781327
STOM	0.78936	0.044602	0.911483
AK4	0.067173	1.341694	0.302058
PON1	0.646644	0.24999	0.837353
ANXA13	0.045488	-0.68288	0.250872
MAOB	0.031791	-0.42896	0.214212
YWHAQ	0.075462	0.254496	0.320055
MAPK3	0.094502	0.257872	0.349959
MARK3	0.221006	0.585939	0.519064
ATP6V0C	0.35106	0.829754	0.636211
CALML3	0.019188	1.467002	0.166914
DPP4	0.263524	0.635182	0.565873
ARNT	0.123729	0.672395	0.399616
CERS1	0.992139	0.015636	1
RPL10	0.637197	-0.17524	0.833511
COL8A1	0.002545	1.601614	0.055387
RPA1	0.023873	0.260321	0.186463
APEX1	0.301936	0.217392	0.603275
CD82	0.279358	-0.30439	0.58196
DCK	0.096798	0.70532	0.354489
CAD	0.001913	0.465164	0.046318
CALR	0.604602	0.080906	0.813002
PDE4A	0.22359	0.240298	0.521792
MAP4	0.000985	0.523328	0.030181
CANX	0.1882	0.168379	0.480067
CFP	0.039579	0.957874	0.237431
PIK3R1	0.32085	0.360079	0.613139
ITPKB	0.498494	0.261892	0.744839
AOAH	0.026897	1.009502	0.198225
PSMB8	0.595428	-0.02374	0.806337
PSMB9	0.76912	-0.13339	0.901915
PSMA5	0.537409	0.143367	0.773175
HLA-DMA	0.010388	0.813683	0.123374
HLA-DMB	0.908102	0.084036	0.9734
PSMB4	0.179497	0.307754	0.471072
PSMB6	0.478274	0.092055	0.730754

PSMB5	0.036828	0.577624	0.229759
GSTM2	0.908103	0.216615	0.9734
ABCD3	0.774166	-0.04982	0.903913
TMOD1	0.192624	-0.25451	0.486094
SSFA2	0.094493	-0.66138	0.349959
LOX	1.07E-06	3.682012	0.000157
CST5	0.967597	-0.26671	0.997177
PEX2	0.851845	0.139082	0.945727
CHAT	0.189699	-0.70025	0.483002
ACADL	0.036802	-1.77644	0.229759
NDUFS1	0.327323	0.116121	0.617719
ADH6	0.638802	0.146214	0.834211
POLD1	0.063332	0.505022	0.294055
TEAD1	0.06969	1.147892	0.308062
SMARCA1	0.148574	0.539427	0.438051
MAPK1	0.511623	0.044544	0.754376
GCA	1	-0.16134	1
RXRB	0.609213	-0.13926	0.816529
ERCC5	0.003305	1.112809	0.065524
GRN	0.918567	-0.04557	0.978657
PTPRM	0.549997	-0.15894	0.781327
LAP3	0.804634	0.101276	0.918848
HSD11B1	0.016869	-1.36393	0.156962
CD34	0.001572	-2.15775	0.040825
CD38	0.955289	-0.30524	0.993074
CD1C	0.071188	1.39937	0.311134
S100A2	0.890235	0.223737	0.966389
GTF2E1	0.000935	1.449596	0.02929
GTF2E2	0.019219	1.04985	0.167051
PCSK1	0.77733	-0.01026	0.906372
PCSK6	0.410792	0.270959	0.683152
TPP2	0.67051	0.026133	0.850681
IMPA1	0.646644	-0.12803	0.837353
CTGF	0.059989	0.887847	0.286703
EPHA2	0.198504	0.614714	0.49217
EPHA3	0.937694	0.114403	0.986498
EPHB2	0.177818	0.722865	0.469455
PTPN6	0.799534	0.024635	0.916173
SHC1	0.074384	0.300835	0.317894
MPG	0.204173	-0.08063	0.497963
CRABP2	0.394814	0.775466	0.672349
ARID4A	0.132726	0.853358	0.415416
KDM5A	0.235662	0.490728	0.537803
COL4A5	0.023538	-1.54935	0.185053
TKT	0.228671	-0.23375	0.528484
CASP1	0.511623	-0.15426	0.754376
NOS3	0.839554	0.112011	0.939212
NOS1	0.897342	-0.03102	0.969793
SERPINB3	0.013461	2.262838	0.141812
LMOD1	0.003297	1.005166	0.065524
RBMS1	0.077978	1.154387	0.325593
PML	0.040551	0.255152	0.240216
TYK2	0.081727	0.768994	0.330094
SERPINA4	0.02974	0.790461	0.208169
EEF1D	0.364426	0.120043	0.646996
OAS2	0.201834	0.238714	0.495032
CRABP1	0.006202	2.191024	0.092951
MARCKS	0.000268	0.783501	0.01304

AQP1	0.462032	0.028049	0.718747
GNA11	0.72417	0.05522	0.876654
ALDH4A1	0.070198	-0.38455	0.309184
PBLD	0.045853	-1.67307	0.252361
ERP29	0.918567	-0.03525	0.978657
PRDX6	0.524435	-0.07991	0.764023
BLVRB	0.190424	-0.20303	0.483515
PRDX5	0.040551	-0.36902	0.240216
DDT;DDTL	0.185996	-0.26929	0.478129
GCHFR	0.074384	-0.44158	0.317894
PRDX3	0.641914	-0.00116	0.835764
ATP5D	0.714303	0.237167	0.87164
RPL12	0.76912	-0.07875	0.901915
ECHS1	0.503174	-0.13575	0.747596
CMPK1	0.923804	0.004862	0.980751
PEBP1	0.404052	-0.10211	0.678271
PDIA3	0.43056	0.104785	0.695651
PPP2R1A	0.216173	0.087944	0.512741
PPP2R1B	0.264068	0.832399	0.566604
CD6	0.065684	-1.48936	0.299507
CDC27	0.013739	0.949513	0.143228
FCER1G	0.023872	0.771901	0.186463
WEE1	0.291387	0.476371	0.595183
PPIF	0.918566	-0.10361	0.978657
TM4SF1	0.30098	1.156196	0.602884
BDKRB2	1	-0.07744	1
NKTR	0.119403	0.952295	0.393785
NMT1	0.004424	0.419955	0.076312
HLA-A	0.642217	0.400035	0.835764
HLA-A	0.04253	0.717129	0.243142
HLA-A	0.711134	0.162648	0.870136
HLA-B	0.331588	-0.23272	0.619341
HLA-B	0.400928	0.826501	0.676817
HLA-B	0.726825	0.289528	0.878607
HLA-B	0.163438	-0.24081	0.453012
HLA-B	0.393617	0.341033	0.671013
HLA-B	0.247021	-0.26286	0.549922
HLA-B	0.57907	-0.18353	0.796675
HLA-B	0.508688	-0.23215	0.752448
HLA-B	0.237842	1.376865	0.540554
HLA-B		0	
HLA-C	0.286651	0.849987	0.590053
HLA-C	0.171935	-0.38222	0.463186
HLA-C	0.220126	-0.60601	0.518019
HLA-C	0.982991	0.005168	1
HLA-C	0.331741	-1.09143	0.619465
HLA-F	0.879415	-0.07293	0.96104
HMOX2	0.130075	0.18847	0.410899
ADSS	0.754042	0.118782	0.894351
AXL	0.01624	1.348569	0.154611
LRPAP1	0.923804	0.078239	0.980751
TSPO	0.126762	-0.48366	0.403998
OXTR	0.745146	-0.03149	0.888646
ADSL	0.084549	0.363213	0.335025
PKLR	0.051649	-0.81725	0.266309
CLIP1	0.241676	0.16653	0.544139
SRI	0.419078	-0.16505	0.688796
GNA15	0.776543	-0.16686	0.905985

GSTT1	0.177798	-1.45743	0.469455
SERPINB1	0.825116	-0.10489	0.930395
GCH1	0.992139	-0.05394	1
SLC7A1	0.468673	0.458868	0.723459
ALDH1B1	1.21E-05	1.164009	0.001115
ALDH3A1	0.040425	-2.07659	0.240216
POLR2B	0.024706	0.263869	0.190022
NTS	0.571349	0.156074	0.793891
LCN1	0.167109	0.418844	0.457139
SDHA	0.739057	0.016611	0.886253
CORO1A	0.241676	-0.16448	0.544139
GDI1	0.088177	0.285447	0.339301
S100A7	0.277593	0.566467	0.579677
MAT2A	0.26355	0.184816	0.565873
SRD5A2	0.175422	-0.51881	0.466796
PRKAR1B	0.996262	-0.07609	1
PRKAR2B	0.537398	0.159292	0.773175
CPS1	0.407707	0.354884	0.680612
RRM2	0.187759	0.722318	0.480067
CASQ1	0.234101	0.607982	0.535784
SDC4	0.054497	-0.69034	0.273313
TIA1	0.000985	0.939651	0.030181
FMO4	0.848323	-0.09152	0.944084
FMO3	0.085728	1.049972	0.335537
HIVEP2	0.568504	-0.22422	0.791838
SLC6A6	0.6553	-0.30319	0.84209
SLC6A4	0.007122	-1.02424	0.101139
DNAJA1	0.00719	0.353496	0.101457
AKT1	0.144006	0.256255	0.431666
AKT2	0.546125	0.061336	0.778738
IL2RG	0.980555	-0.04677	1
UQCRC1	0.374984	-0.08681	0.656862
HIBADH	0.754042	0.009718	0.894351
ATIC	0.252448	0.193776	0.555643
HNRNPH3	0.159052	0.236282	0.449897
HNRNPH1	0.136904	0.193296	0.421544
CASP14	0.079667	1.093452	0.325919
YWHAB	0.142204	0.215701	0.428816
SFN	0.164991	-0.34725	0.454525
STIP1	0.76912	0.006161	0.901915
S100A11	0.918567	0.081215	0.978657
FCGR2B	0.022402	1.874959	0.179917
FCGR2C	0.109581	0.878597	0.377423
CEACAM8	0.170642	-1.04961	0.461822
L1CAM	0.49392	0.423928	0.741997
INPP5B	0.00763	1.617845	0.10525
PRDX2	0.024706	-0.32537	0.190022
ARRB2	0.97106	-0.0873	0.997592
GK;GK3P	0.902877	-0.07915	0.971583
VIPR1	0.014974	-1.06829	0.150049
CCR1	0.607012	0.363095	0.81555
CCR7	0.322009	0.130597	0.613139
GPR183	0.01123	1.166916	0.128539
CDA	0.156729	0.975261	0.447022
DCTD	0.835399	0.002922	0.935999
PYCR1	0.292764	0.446706	0.595788
SLC8A1	0.930143	0.08663	0.983996
GBP1	0.241676	0.273315	0.544139

GBP2	0.09982	0.525042	0.359216
ELF1	0.000759	2.350011	0.025904
HPD	0.310183	-0.43122	0.609864
GTF2H1	0.000386	1.872205	0.017122
STX2	0.000624	2.153972	0.023434
DSG3	0.380237	0.42721	0.661698
CTH	0.111922	-0.94421	0.382523
ICAM3	0.82506	-0.03949	0.930395
RPL9	0.02152	0.200812	0.175496
CD70	0.980555	0.118959	1
ACSL1	0.063339	-0.25182	0.294055
CDH5	0.094471	-0.6416	0.349959
KIF5B	0.185996	0.120683	0.478129
CSTF2	0.112657	0.308189	0.382523
LSP1	0.041861	0.370405	0.242066
DUT	0.709387	-0.02324	0.869179
GUCY1A2	0.180545	-0.75622	0.472575
ABCC1	0.030755	0.539749	0.211475
CKS2	0.291769	0.379417	0.595183
S100A3	0.563678	-0.15748	0.789479
ABCD1	0.015071	0.480841	0.150049
MAN1A1	0.034511	0.679685	0.222148
KDELR2	0.188151	0.607842	0.480067
TTK	0.072914	0.510886	0.315444
MCM4	0.057907	1.226531	0.280499
MCM5	0.074381	0.947213	0.317894
MCM7	0.266365	0.854599	0.568807
GALNS	0.004611	0.589073	0.078316
RNASE4	0.259673	0.559053	0.563057
SDC2	0.641109	0.426322	0.835764
CD68	0.154996	0.637212	0.444958
SHMT1	0.149515	-0.4024	0.438811
SHMT2	0.000706	0.475627	0.024871
EVI2B	0.677598	0.049719	0.854568
EPHX2	0.071055	-0.93928	0.311134
HSPA4	0.153276	0.125823	0.443165
GRK5	0.512056	-0.28932	0.754913
MPI	0.646644	0.05346	0.837353
PRSS3	0.322009	0.128716	0.613139
GPC1	4.19E-09	3.473083	2.62E-06
BTC	0.26324	-0.38642	0.565873
PFN2	0.003627	0.702178	0.069037
CA8	0.08745	-0.82134	0.338891
CTNNA1	0.218632	-0.21143	0.515816
CTNNB1	0.466082	-0.10073	0.721065
BMI1	0.960376	-0.36876	0.994122
PHB	0.426712	0.083676	0.693599
PTPN7	0.46178	-0.56052	0.718747
SERPINB6	0.247021	0.361012	0.549922
NF2	0.367923	0.055989	0.650718
RDX	0.317645	-0.11798	0.613139
RPA3	0.208914	0.120644	0.503054
SFTPD	0.019106	-1.37445	0.166854
RFC4	0.069183	0.426371	0.306571
RFC2	0.131757	0.502516	0.413476
RFC1	0.528742	0.262142	0.767576
RPL22	0.085745	0.086989	0.335537
GTF2F1	0.091287	0.645242	0.345435

SPR	0.003744	-0.5578	0.070138
ADM	1	-0.01065	1
SPRR1A	0.508688	-0.21618	0.752448
PTGS2	0.001304	2.491704	0.035705
APLNR		0	
THBS2	1.05E-08	3.178459	5.30E-06
THBS4	0.017358	1.191441	0.159467
IDUA	0.409636	-0.33066	0.682657
CLCN1	0.262189	0.377536	0.565636
KRT9	0.162993	0.690885	0.453012
SAA4	0.786058	0.195857	0.910989
FBN1	0.008089	0.485631	0.107782
FBN2	0.404659	0.563169	0.67885
PCK1	0.319672	-0.24174	0.613139
IRS1	0.034088	1.011716	0.221621
AGL	0.28378	-0.23614	0.586874
MYH9	2.54E-07	0.446998	5.31E-05
MYH10	0.144006	0.360163	0.431666
TIE1	0.447034	-0.2226	0.707261
COPB2	0.064284	0.1836	0.296208
ACTN2	0.00537	-1.31534	0.086604
SOAT1	0.462056	0.330033	0.718747
ADD1	0.918567	0.01126	0.978657
ADD2	0.414243	-0.29196	0.685923
BSG	0.015348	0.365698	0.151713
TIMP3	0.014135	0.80425	0.144889
ADRBK2	0.794794	-0.27383	0.913708
FUS	0.075462	0.203979	0.320055
NUP214	0.036237	0.279317	0.227448
DEK	0.226131	0.161025	0.525131
ATP7B	0.019188	0.746918	0.166914
HNF1B	0.308073	-0.61155	0.609277
MYH11	0.442223	0.204993	0.703704
GLRX	0.407778	-0.21793	0.680612
CHKA	0.88688	-0.02272	0.96416
PPM1A	0.330591	-0.15711	0.619341
IGFALS	0.8483	-0.04186	0.944084
AHR	0.058787	0.428639	0.283235
KRT20	0.392489	0.178016	0.671013
KRT2	0.090665	0.620206	0.343901
HMGCL	0.167008	-0.26172	0.457139
FLT4	0.907445	-0.24002	0.9734
KDR	0.428964	0.48266	0.695651
PSMC2	0.272095	0.108261	0.575039
SLC16A2	0.121151	1.146121	0.396836
CHI3L1	0.067835	1.352393	0.303736
GGT5	0.155183	0.322983	0.444958
GJA5	0.673801	-0.33974	0.853
ARL2	0.856059	0.078262	0.946993
ARL3	0.070198	0.326762	0.309184
TRIM23	0.568296	-0.08785	0.791838
MAP2K2	0.255193	0.168773	0.557593
ZNF76	0.44489	0.251976	0.706713
ATP5C1	0.660922	0.022269	0.844596
ATP6V1E1	0.360948	0.077989	0.644411
CPOX	0.179497	0.311516	0.471072
RPL4	0.257958	-0.01061	0.560462
NUDT1	0.115525	1.024188	0.387731

LONP1	0.206534	0.14434	0.500256
PGM1	0.689846	0.081037	0.859429
PPP1CC	0.000268	0.802272	0.01304
ACVR1B	0.716722	0.065402	0.873394
TGFBR1	0.000451	2.17113	0.019184
GNL1	0.073319	0.297361	0.31592
LTBR	0.447034	0.298203	0.707261
SERPINB5	0.048183	1.698635	0.257798
POLR2I	0.247021	0.345307	0.549922
SERPINF1	2.26E-05	1.078357	0.001867
SREBF1	0.056315	0.469501	0.277104
DLST	0.923804	0.046317	0.980751
GMPR	0.086783	-1.42357	0.337854
GPX4	0.151386	-0.20823	0.44125
CFHR2	0.053107	2.041748	0.270063
ACVRL1	1	-0.20581	1
HSD17B2	0.713801	-0.45094	0.87164
SCNN1A	0.69426	0.079807	0.861478
SRP14	0.213733	-0.40726	0.509228
TGFBR2	0.577365	-0.20654	0.79666
NUP62	0.015348	0.23795	0.151713
HPCAL1	0.97632	-0.05814	0.998921
FDFT1	0.522193	0.447599	0.762942
ZEB1	0.688686	-0.14517	0.859429
PIGA	0.247982	0.984191	0.551286
TAGLN2	0.257958	0.121752	0.560462
TALDO1	0.181644	-0.23622	0.472575
SNCA	0.471956	0.127273	0.726403
ETFB	0.164991	-0.19711	0.454525
RBMX	0.637197	0.105403	0.833511
BRCA1	0.02519	0.465663	0.192018
COIL	0.033619	0.891052	0.220876
GGCX	0.646641	0.132158	0.837353
ITGAE	0.001003	2.357001	0.030576
LIPA	0.462056	0.046394	0.718747
ATP6V1A	0.407778	0.092915	0.680612
HSPA9	0.960544	0.024737	0.994122
EIF4A3	0.027356	0.19573	0.199874
IGHMBP2	0.986528	0.020392	1
RPS19	0.228671	0.07652	0.528484
RPL3	0.581788	-0.01356	0.797998
COL15A1	0.577274	0.048259	0.796634
COL18A1	0.960544	-0.09903	0.994122
MPV17	0.704475	0.855811	0.867291
DDOST	0.075462	0.254984	0.320055
ANP32A	0.97632	-0.03369	0.998921
FEN1	0.007333	0.709858	0.102825
PLA2G5	0.107007	0.813878	0.371691
CUX1	0.118765	0.212635	0.392409
MMP12	0.00525	3.011296	0.085373
CAPG	0.330591	0.168826	0.619341
CAP2	0.005771	1.302326	0.089283
IL6ST	0.168028	-0.91671	0.45845
GP5	0.421207	0.132165	0.691066
CEACAM6	0.819807	-0.299	0.92687
TXLNA	0.0799	0.415375	0.325919
CCT6A	0.546147	-0.05102	0.778738
CD79B	0.85486	-0.05286	0.946993

NNMT	0.00049	1.059691	0.020212
IFI27	0.404384	-0.68034	0.678624
PSMB10	0.374984	-0.13298	0.656862
VHL	0.603077	0.335713	0.812922
ADH7	0.328863	0.706546	0.619341
PBX1	0.018614	1.575352	0.165633
PBX2	0.346993	0.741653	0.632606
PBX3	0.013261	1.115393	0.141015
RPL13A	0.944783	-0.01748	0.988547
ARL1	0.177368	0.201185	0.468602
MLH1	0.141154	0.783918	0.427981
STAT3	0.407778	0.100431	0.680612
USP8	0.048948	0.776614	0.259694
PEX19	0.103963	0.447247	0.366863
MDH1	0.72417	-0.06032	0.876654
MDH2	0.546147	0.116264	0.778738
RFC5	0.082195	0.468056	0.330671
RFC3	0.494799	0.203178	0.741997
HADHA	0.466082	-0.05395	0.721065
EIF2S3;EIF2S3L	0.533067	0.055323	0.7709
LEP	0.683728	-0.11242	0.85709
ETV3	0.041395	0.855514	0.240826
CASR	0.447034	-0.32014	0.707261
CETN2	0.632496	0.002694	0.830965
ETV6	0.109616	0.948505	0.377423
EIF2D	0.073842	0.643157	0.316943
CD200	0.005341	1.004088	0.086344
MNDA	0.520146	0.044499	0.760272
PRPH	0.115488	-0.84009	0.387731
WNT5A	0.001125	2.570401	0.032731
PTGDS	0.664722	-0.4419	0.847351
BUD31	0.192667	0.307712	0.486094
UBA7	0.274985	0.223918	0.577625
NAA10	0.581788	0.11839	0.797998
KDM5C	0.024218	1.1203	0.18849
PPP1R2;PPP1R2P3	0.255193	0.456221	0.557593
CSK	0.442223	0.113926	0.703704
PNPLA4	0.446222	-0.33259	0.706805
GARS	0.09581	0.32151	0.352331
IARS	0.09982	0.191317	0.359216
MAP3K8	0.920812	-0.00018	0.98039
FOLR3	0.540968	0.185739	0.775521
SLC19A1	0.149142	0.406684	0.438811
EIF1	0.794443	-0.02485	0.913708
CCR2	0.089503	0.572776	0.341477
TSPAN7	0.066178	-1.53971	0.300455
PRKCI	0.918567	-0.01695	0.978657
ELK3	0.391415	0.592589	0.670039
ACTR1B	0.866426	0.029768	0.953042
ECI1	0.167008	-0.12648	0.457139
TMPO	0.965801	0.060404	0.99616
TMPO	0.014008	0.488663	0.144006
STAT1	0.084549	0.399406	0.335025
STAT6	0.498977	0.129283	0.744839
STAT5A	0.010997	0.422351	0.127049
GRIA1	0.40269	0.170559	0.678038
SKIV2L2	0.563832	0.084454	0.789479
AKR1C3	0.528679	-0.45307	0.767576

ARHGAP25	0.646644	-0.31426	0.837353
PIK3CA	0.014853	0.859933	0.14976
PIK3CB	0.478714	0.207658	0.731324
MTOR	0.181644	0.175343	0.472575
PI4KA	0.466082	0.128394	0.721065
HAL	0.149142	-0.70388	0.438811
EPS15	0.012538	0.291056	0.136468
MLLT3	0.826366	0.098489	0.93133
CASP3	0.314461	0.120628	0.612305
CASP2	0.009146	1.475118	0.116274
DPP6	0.960709	0.151802	0.994122
RPS27	0.779221	-0.21146	0.906856
ABL2	0.064893	0.856634	0.298022
FRK	0.54908	0.574137	0.781251
HELZ	0.02859	1.040527	0.204681
NCAPD3	0.25134	0.639096	0.555301
RBM34	0.095273	1.285026	0.351755
LIFR	0.79579	-0.00738	0.914433
LRPPRC	0.272095	0.130977	0.575039
ACAA2	0.478274	-0.16094	0.730754
RPL35	0.478274	0.253274	0.730754
WAS	0.861239	-0.09994	0.950165
CDKN2A	0.980924	-0.19776	1
CDKN2C	0.050503	1.480192	0.263221
PRCP	0.438315	0.075855	0.701933
CXCL5	0.837269	-0.46552	0.937805
HTT	0.226131	0.24075	0.525131
ECE1	0.034511	0.781221	0.222148
MTHFR	0.855917	-0.0438	0.946993
SLC1A3	0.002999	1.839368	0.062294
SLC1A2	0.889918	0.135763	0.96614
SLC1A1	0.246076	-0.73458	0.549922
SLC1A4	0.034501	0.583901	0.222148
PAFAH1B1	0.020421	0.280596	0.170514
PTGIR		0	
MCAM	0.009087	0.460666	0.115713
CRAT	0.231232	-0.21145	0.531962
CTSO	0.307761	-0.66821	0.609277
CTSK	0.000193	2.653924	0.01029
MATR3	0.026002	0.316359	0.193808
MSH2	0.208914	0.440975	0.503054
GRK6	0.781486	-0.02076	0.90873
BTD	0.196763	0.298432	0.490309
GPD2	0.301936	0.171121	0.603275
SSR1	0.000151	0.568173	0.008631
ALDH3B1	0.018696	-0.63625	0.165633
MAGEA1	0.69426	0.128157	0.861478
MAGEA4	0.041841	1.205056	0.242066
MAGEA9	0.319672	0.284478	0.613139
MAGEA10	0.6137	0.288417	0.819264
PTPN9	0.003514	0.897046	0.067901
ZAP70	0.151534	0.661318	0.441443
SYK	0.003744	0.504758	0.070138
RANBP1	0.627808	-0.36712	0.828932
NAMPT	0.14042	0.344445	0.426568
AFM	0.641914	-0.01085	0.835764
LPAR6		0	
PSMC4	0.197211	0.162906	0.490309

GATA4	0.079293	0.455094	0.32578
NKX2-1;NKX2-4	0.397911	-0.3963	0.675323
TSFM	0.856059	0.033476	0.946993
ASPA	0.116711	-0.52717	0.389656
MMP13	0.050063	0.960718	0.26298
ABCG1	0.119052	1.038497	0.392861
PPIC	1.19E-06	1.158566	0.000169
VDAC2	0.434427	0.157934	0.698194
ACADSB	0.013753	-0.59254	0.143228
CBX5	0.066208	0.335809	0.300455
USP5	0.02152	0.332783	0.175496
MAPK8	0.185012	0.705214	0.477255
MAPK9	0.374979	0.086548	0.656862
MAP2K4	0.002089	0.664815	0.048476
MKI67	0.044877	1.165053	0.249738
PHKA2	0.774145	0.277673	0.903913
PHKA1	0.617543	-0.13331	0.822194
SLC15A1	0.993518	-0.05813	1
RANGAP1	0.197211	0.161598	0.490309
RECQL	0.000855	0.484179	0.027892
NOP2	0.05451	0.270275	0.273313
ATRX	0.233812	0.440692	0.535403
CRK	0.226131	0.250723	0.525131
CRKL	0.181644	0.184709	0.472575
MTIF2	0.850885	0.087182	0.944945
BAG6	0.422885	0.10184	0.691467
GSTM5	0.170093	-0.5605	0.461474
NSF	0.09193	0.240265	0.345626
CDKN1B	0.773891	-0.25599	0.903913
NOTCH1	0.104629	0.702828	0.368512
MAP2K3	0.528742	0.269154	0.767576
BRCC3	0.804634	0.101294	0.918848
RPL27A	0.934288	0.013838	0.984157
RPL5	0.426712	0.093625	0.693599
RPL21	0.887225	-0.27764	0.96416
RPL28	0.470127	-0.14044	0.72456
RPS9	0.929044	0.012937	0.983115
RPS5	0.604602	0.040448	0.813002
RPS10	0.520146	-0.003	0.760272
MAP1B	0.007479	1.063321	0.10395
GNPDA1	0.866426	0.076716	0.953042
NEDD4	0.025998	0.571416	0.193808
YAP1	0.78936	0.112358	0.911483
UTRN	0.955289	0.070963	0.993074
IQGAP1	0.586319	0.033821	0.801141
HAAO	0.894851	0.135667	0.968678
GYG1	0.192667	-0.2177	0.486094
STT3A	0.061485	0.273976	0.289565
RABIF	0.041079	1.260573	0.240826
PLA2G4A	0.754042	0.095316	0.894351
RAP1GAP	0.12661	-0.55706	0.403998
CAPZA2	0.046008	0.166655	0.252361
CAPZB	0.944783	0.003451	0.988547
EIF1AX	0.411523	-0.46983	0.683152
ALDH1A3	0.007769	1.043751	0.105988
QARS	0.581788	0.006223	0.797998
RPL29	0.347246	-0.37174	0.632606
ID4	0.045488	-0.69984	0.250872

LGALS7	0.277976	1.053304	0.579736
ZFP36L2	0.020153	1.692157	0.169843
UQCRFS1	0.632496	-0.08447	0.830965
XDH	0.907931	-0.14335	0.9734
ATP5O	0.897655	0.002778	0.969793
LIMS1	0.0334	0.41173	0.219585
GLIPR1	0.039482	1.738016	0.23721
CXCL12	0.100111	1.709398	0.359821
SLC6A11	0.167805	0.751459	0.458066
SLC6A9	0.224923	0.424464	0.52386
PREP	0.595428	0.112802	0.806337
ME1	0.498972	-0.06804	0.744839
IREB2	0.478129	-0.34549	0.730754
TM4SF4	0.15526	0.210846	0.444958
TFPI2	0.054366	1.315329	0.273313
LEPR	0.375606	-0.42696	0.657384
RFX2	0.135051	0.611677	0.417833
RFX3	0.592958	0.292313	0.806337
RFX5	0.539494	0.051246	0.775168
PIP4K2A	0.018368	0.352298	0.163766
SOX2	0.964156	0.101145	0.99616
SOX9	0.937694	-0.15679	0.986498
ARCN1	0.364426	0.119202	0.646996
LSS	0.76912	0.047337	0.901915
PPP3CC	0.015605	1.227369	0.152856
GCLC	0.347246	0.60056	0.632606
GCLM	0.559385	0.171389	0.78633
CD151	0.759058	-0.11754	0.896847
PCP4	0.189621	0.732372	0.482914
KCNJ5	0.83432	-0.03479	0.935999
IFNAR2	0.923284	0.071147	0.980751
TRAPPC10	0.744041	0.160387	0.888303
PSMD8	0.929044	-0.01779	0.983115
SERPINB4	0.055868	1.11559	0.276127
SERPINB10	0.263574	0.627343	0.565873
PRRC2A	0.000484	1.172833	0.020165
GSS	0.609213	0.058578	0.816529
CCT5	0.944783	0.006384	0.988547
PTDSS1	0.327323	0.316157	0.617719
KRT6C	0.013533	1.316783	0.141885
NES	0.856059	0.094885	0.946993
HSPA13	0.006775	0.727959	0.098484
AMT	0.025917	-1.53254	0.193808
CSNK1A1	0.577274	0.065054	0.796634
CSNK1D	0.272095	0.36286	0.575039
IDH2	0.734083	-0.00169	0.882858
PIK3CG	0.48451	0.280095	0.735121
PITPNB	0.011206	0.388023	0.128397
MASP1	0.677979	-0.08137	0.854776
NOV	0.300882	0.227641	0.602797
CD97	0.292764	0.205388	0.595788
POLD2	0.013493	1.314377	0.141812
MARCKSL1	0.164991	0.230229	0.454525
PXN	0.511623	0.216692	0.754376
CAMLG	0.010949	0.980889	0.127049
NR2C2	0.014602	1.405299	0.147768
MAPKAPK2	0.404039	0.207131	0.678271
DNASE1L1	0.037224	1.396069	0.231548

ALDH9A1	0.199513	-0.16254	0.49217
RPL34	0.280828	0.230739	0.583434
RPIA	0.112656	-0.59275	0.382523
LMAN1	0.014008	0.326895	0.144006
SLC11A1	0.205366	0.853799	0.49957
SLC11A2	0.054603	1.279093	0.273507
NASP	0.44615	0.275597	0.706795
FMO5	0.229576	-0.92531	0.529537
FASN	0.704483	-0.16737	0.867291
FNTA	0.003666	0.485439	0.06938
FNTB	0.155808	1.15262	0.446067
DHPS	0.426753	0.670099	0.693599
CCT3	0.744041	0.000683	0.888303
MRPL19	0.554955	0.100856	0.783439
ARRB1	0.052873	-0.31317	0.269122
TUFM	0.308157	-0.11226	0.609277
AMPH	0.622203	0.122944	0.825978
ALDH7A1	0.520146	-0.09228	0.760272
CDC34	0.00094	2.300321	0.02929
INPP1	0.822544	-0.10482	0.92929
CYB561	0.40574	-0.76711	0.679973
GLUD2	0.61986	-0.33033	0.823535
CENPF	0.000906	1.889948	0.028881
SRP9	0.97106	-0.15447	0.997592
UBE2A	0.077557	1.349495	0.324641
PCYT1A	0.003823	0.398037	0.070612
AARS	0.058787	0.326863	0.283235
CARS	0.03232	0.354966	0.215938
HARS2	0.892438	-0.05985	0.966699
SARS	0.133456	0.178535	0.416228
PPM1F	0.35064	0.169288	0.635657
DGKG	0.556057	0.150574	0.783439
TTPA	0.632486	-0.23533	0.830965
MAN2A2	0.15309	0.193351	0.443165
PRIM1	0.757912	0.096226	0.896847
PRIM2	0.86596	0.038614	0.953042
CASP4	0.382127	0.474516	0.661698
CSNK1E	0.712043	0.136525	0.870223
CTCF	0.939534	-0.04695	0.986498
CEBPD	0.751226	0.266942	0.892921
PSMB3	0.269226	0.194941	0.571371
PSMB2	0.396661	0.155227	0.67382
MCM2	0.010996	1.447352	0.127049
THBS3	8.52E-08	3.887793	2.20E-05
COMP	0.000106	2.617048	0.006612
ACADVL	0.600007	0.076294	0.810262
YLPM1	0.396661	0.204661	0.67382
ACOT2	0.147392	0.999039	0.436386
VPS41	0.039909	0.56643	0.23845
TMED10	0.228671	0.263865	0.528484
RBM25	0.407778	0.200747	0.680612
NUMB	0.835403	-0.09607	0.935999
CLK1	0.120471	0.631937	0.39531
CLK2	0.192782	0.720244	0.486274
CLK3	0.296535	-0.62252	0.599275
PGF	0.079293	0.396626	0.32578
VEGFB	0.805837	0.056982	0.919879
VEGFC	0.375847	0.245529	0.657392

PSEN1	0.648867	0.163281	0.839222
EIF2B2	0.153276	0.257471	0.443165
HINT1	0.474191	-0.11635	0.727018
RARRES1	0.499616	-0.17077	0.745391
FHIT	0.597706	0.064807	0.808505
NUP153	0.38573	0.23576	0.664306
RANBP2	0.069183	0.235818	0.306571
RGS19	0.003769	1.36776	0.07038
RGS3	0.280957	0.43781	0.583538
PSEN2	0.138998	0.792701	0.4248
TSC2	0.003904	0.684189	0.071121
NDUFV1	0.944783	-0.00933	0.988547
GSK3A	0.079897	0.788792	0.325919
GSK3B	0.00841	0.579474	0.110127
STK19	0.962053	0.057702	0.99507
TAF6	0.164991	0.590584	0.454525
KLK7	0.988991	0.011587	1
GZMK	0.249041	-1.05923	0.552533
SULT1E1	0.068539	0.880817	0.305289
NT5C2	0.000207	0.647497	0.010881
SEPHS1	0.709387	0.149271	0.869179
SEPP1	0.631631	-0.25364	0.830965
CAMP	0.683724	0.465326	0.85709
MTHFS	0.400274	0.224847	0.67622
GMPS	0.164991	0.265169	0.454525
LIG3	0.997369	0.091068	1
LIG4	0.063602	0.829842	0.294903
MRE11A	0.033952	0.323306	0.221141
ENTPD1	0.002233	0.703413	0.050657
HNMT	0.665709	-0.22552	0.847351
GNAQ	0.494799	0.048654	0.741997
GNG4	0.067799	1.26767	0.303698
GNG10	0.118765	0.55296	0.392409
IDH3A	0.45805	-0.10624	0.715224
MEOX1	0.881175	-0.19899	0.961515
SULT1A1	0.016204	-0.59778	0.154611
SULT1A2	0.070956	-1.09899	0.311134
CRIP1	0.819984	-0.02264	0.92687
MMP14	2.53E-09	1.996101	2.00E-06
PPOX	0.784286	0.031492	0.909506
GDI2	0.197211	0.216515	0.490309
EMD	0.913333	-0.02365	0.975706
CPT1A	0.068179	0.370396	0.303928
GATM	0.301936	0.566528	0.603275
SLC26A2	0.005049	1.574635	0.083219
SERPINB8	0.578778	0.325077	0.796675
SERPINB9	0.206534	0.265312	0.500256
SERPINH1	3.73E-07	0.787721	6.96E-05
CSRP3	0.129735	1.145118	0.410641
PDLIM4	1.55E-09	2.548929	1.33E-06
ST13	0.586319	0.143737	0.801141
PEX5	0.032854	0.487169	0.217297
ERF	0.057217	1.068183	0.279188
VASP	0.052873	0.177467	0.269122
DNM2	0.72417	0.009738	0.876654
METAP2	0.779221	0.019414	0.906856
NUDT2	0.204149	0.491313	0.497963
TNFSF10	0.602579	0.130978	0.8126

CDK7	0.129313	0.952875	0.40989
HLCS	0.411372	0.503619	0.683152
KNTC1	0.264682	0.26835	0.567373
RASSF2	0.001054	1.329857	0.031507
CDK9	0.023466	0.402641	0.184845
LRBA	0.009264	-0.54192	0.116462
BCAM	0.007932	-0.69141	0.107292
PPT1	0.007779	0.401804	0.105988
RPL14	0.794443	-0.10906	0.913708
CCT8	0.568296	-0.05793	0.791838
CCT4	0.892438	-0.02119	0.966699
ATP1A2	0.13996	-0.52452	0.426568
ANXA11	0.934288	-0.00361	0.984157
PAPOLA	0.040551	0.625046	0.240216
FXR1	0.000122	0.537149	0.007389
FXR2	0.020065	0.410744	0.169555
GZMM	0.606932	-0.25656	0.815541
RAB5C	0.002045	0.305167	0.047942
RAB7A	0.422885	0.108364	0.691467
RAB9A	0.997369	0.008294	1
RAB13	0.147661	0.336682	0.436386
RAB28	0.153854	0.708248	0.444477
RAB27A	0.012538	-0.49032	0.136468
FABP6	0.547409	-0.54398	0.779573
SCNN1B	0.799868	0.030332	0.916176
SCNN1G	1	-0.09521	1
PLCD1	0.223612	0.397478	0.521792
DAP	0.796968	0.191968	0.915123
DAP3	0.010194	0.393324	0.121876
DUSP3	0.056188	0.371253	0.276683
MMP15	0.4088	0.536602	0.681762
NOVA1	0.300882	0.230851	0.602797
SMARCA2	0.175259	0.246303	0.466796
SMARCA4	0.343873	0.216977	0.630156
IDH3G	0.32085	-0.15927	0.613139
GALK1	0.123514	0.182611	0.399387
SSR4	0.001562	0.482377	0.040678
BCAP31	0.656149	-0.07531	0.84209
P2RX1	0.423285	0.367693	0.691917
TPMT	0.563831	-0.03884	0.789479
CYP2J2	0.346723	0.340237	0.632606
RENBP	0.515871	0.066344	0.757825
MECP2	0.537409	-0.03656	0.773175
HCFC1	0.197211	0.153132	0.490309
IRAK1	0.063192	1.417752	0.294055
CAV2	0.30811	-0.80567	0.609277
ALDH3A2	0.656149	-0.1084	0.84209
ALDH5A1	0.010997	-0.53631	0.127049
GPC3	0.88748	0.22426	0.964342
HSD17B4	0.442223	-0.18523	0.703704
PSMD7	0.031791	0.275763	0.214212
UBE2D1	0.18937	-1.76062	0.482385
GPM6A	0.211643	-0.72324	0.507094
CCR5	0.171491	0.524581	0.462438
CCR6	0.845382	0.046815	0.942239
SUOX	0.398393	-0.43032	0.675933
SGSH	0.526725	-0.82342	0.766252
ARSD	0.882017	0.112751	0.961515

ARSE	0.709651	0.724309	0.869407
STAT5B	0.026884	0.773385	0.198225
USP11	0.000186	1.380745	0.010031
KCNQ1	0.787673	0.013325	0.911483
CLCN3	0.136595	0.320669	0.421413
CLCN4	0.571349	0.036687	0.793891
CLCN5	0.720143	0.219683	0.875395
CLCN6	0.504624	-0.38204	0.749235
CLCN7	0.204174	0.076542	0.497963
PLXNA3	0.23546	0.261248	0.537676
DYNLT3	0.12513	0.078043	0.401334
VAMP7	0.537409	0.22773	0.773175
XK	0.319672	0.196536	0.613139
RPS6KA3	0.559385	-0.04852	0.78633
BMX	0.971656	-0.17752	0.997592
ZNF75D	0.291769	0.34106	0.595183
AFF1	0.015658	1.147413	0.153007
ADCY7	0.36297	0.409592	0.646802
NR0B1	0.22359	0.442012	0.521792
TKTL1	0.163587	0.714867	0.453012
AKR1D1	0.759464	-0.04037	0.896997
HDGF	0.581788	0.138489	0.797998
LUM	0.009264	0.464995	0.116462
PRELP	0.269226	0.188143	0.571371
CNN1	0.001187	0.898634	0.03367
CCNH	0.261927	0.21138	0.565402
MNAT1	0.076212	1.044724	0.322007
NEK3	0.266479	0.083584	0.568807
NEK4	0.976387	-0.07491	0.998921
UBE2E1	0.286448	0.727607	0.590053
NDUFA8	0.929044	0.018123	0.983115
HNRNPA3	0.739057	-0.03011	0.886253
PGD	0.609213	-0.10215	0.816529
HNRNPM	0.021897	0.337762	0.176756
KPNA2	0.001597	1.482757	0.041198
KPNA1	0.554954	-0.15454	0.783439
NCBP2	0.274505	0.279305	0.577625
RAP1GDS1	0.094502	0.430201	0.349959
JAK3	0.00503	1.068096	0.083138
DGKE	0.005865	-1.26694	0.08985
POLR2H	0.520146	0.314567	0.760272
POLR2J;POLR2J3;POLR2J2	0.577253	-0.03866	0.796634
MAP2K6	0.040321	1.404842	0.240173
ARHGDIA	0.133456	0.305502	0.416228
ARHGDIB	0.67051	-0.15487	0.850681
SLC7A2	0.173783	0.530927	0.46573
AGFG1	0.434427	0.121799	0.698194
HNRNPF	0.378546	0.068573	0.659304
STAT2	0.000444	0.64498	0.019015
GTF2A1	0.289266	0.451862	0.593134
GTF2A2	0.100012	-1.30026	0.359674
MSH6	0.028293	0.722109	0.203235
KIF11	0.292977	0.589889	0.595821
VAV2	0.689846	-0.083	0.859429
ZNF131	0.19172	0.574551	0.485915
ZNF143	0.008427	1.154025	0.11022
RBM5	0.500879	0.134353	0.746473
CHN2	0.274206	0.50964	0.577625

HRSP12	0.902819	0.34051	0.971583
SMS	0.515876	-0.09212	0.757825
HK2	0.684992	-0.05785	0.85709
HK3	0.371444	0.180295	0.65423
EFNB2	0.707926	0.222237	0.869179
EFNA5	0.310183	0.240818	0.609864
MRPL12	0.404052	0.231798	0.678271
STC1	0.702511	0.166148	0.866226
DGKQ	0.144874	0.76017	0.43345
NDST1	0.642886	0.275731	0.836333
NDST2	0.542046	0.078049	0.775521
THOP1	0.049723	0.472851	0.261364
AKR1C2	0.348549	1.064576	0.634355
CAPZA1	0.02267	0.266103	0.180886
HMGA2	0.009668	2.780277	0.119174
CRIP2	0.54177	0.191227	0.775521
NUP98	0.360948	0.141867	0.644411
BLVRA	0.159052	-0.26305	0.449897
SLC25A1	0.559385	0.106359	0.78633
PPP5C	0.052873	0.137263	0.269122
PLK1	0.842222	0.060791	0.940547
DAPK1	0.353314	0.645233	0.638094
ARFIP2	0.552736	-0.35959	0.783393
ARFIP1	0.794443	-0.06314	0.913708
NUDT6	0.485692	-0.38116	0.736106
NUBP1	0.627809	-0.07852	0.828932
ACLY	0.061485	0.231027	0.289565
COL4A4	0.4794	-0.53408	0.731833
FOSB	0.192496	0.472286	0.486094
CEBPG	0.476091	0.466736	0.728924
METAP1	0.537409	-0.14446	0.773175
SUCLG1	0.231232	-0.17045	0.531962
MVD	0.861238	0.528584	0.950165
PGGT1B	0.483618	0.695725	0.734969
RABGGTB	0.913328	-0.01063	0.975706
COPB1	0.067188	0.203111	0.302058
COPA	0.008089	0.235868	0.107782
CTSC	0.76912	-0.07224	0.901915
LIMK1	0.083234	0.776221	0.332962
LIMK2	0.048642	1.358749	0.259247
CLTCL1	0.914896	0.00966	0.976905
AP3M2	0.000688	1.624435	0.024536
AP2S1	0.05791	0.360553	0.280499
HCCS	0.76912	0.277759	0.901915
ITGA8	0.236483	-0.74827	0.537906
MAPK12	0.107487	0.758371	0.372977
SLC5A3	0.322817	0.617549	0.613707
PTTG1IP	0.114105	0.608561	0.385201
POLR2K	0.969947	0.818079	0.997592
SMTN	0.038017	1.280121	0.233324
PLA2G16	0.166175	0.869496	0.457139
SLC16A1	0.015582	1.433906	0.152856
IST1	0.162993	0.249071	0.453012
SEC24C	0.00719	0.256061	0.101457
SUB1	0.887225	-0.00907	0.96416
POLG	0.627465	-0.03397	0.828932
CLNS1A	0.295317	0.757852	0.598344
CRISP3	0.953103	0.024966	0.993074

BLM	0.339777	0.604084	0.627033
RARS	0.939534	0.003351	0.986498
HIRA	0.418668	0.118841	0.688796
ATXN3	0.238822	0.549602	0.541047
ATXN1	4.32E-06	3.141828	0.000494
ATN1	0.00881	1.096612	0.11325
TERF1	0.96026	-0.19251	0.994122
PMS1	0.147023	0.347329	0.436347
PMS2	0.550674	0.143737	0.781335
CACNA2D1	0.241676	0.399298	0.544139
YARS	0.965801	-0.03077	0.99616
USP14	0.32085	0.15726	0.613139
PRKAG1	0.26355	0.239881	0.565873
PRKAA2	0.772447	0.234062	0.903913
HSPA2	0.301936	0.420993	0.603275
BCAT1	0.043861	0.731838	0.247494
ATP12A	0.222633	-1.15181	0.521263
ATP1B3	0.03232	0.419489	0.215938
RAD23A	0.54177	-0.14483	0.775521
RAD23B	0.208914	0.12677	0.503054
EPHB3	0.005771	1.197264	0.089283
EPHB4	0.796831	-0.06889	0.915123
EPHA4	0.481971	0.409218	0.733586
NAGLU	0.38573	0.156472	0.664306
GALC	0.781746	-0.41067	0.908746
AK2	0.2958	-0.22175	0.598344
PRRX1	2.68E-08	3.91522	8.36E-06
GAS1	1	-0.02722	1
GYS2	0.411887	0.206559	0.683152
EMP3	0.005372	1.394139	0.086604
UGT2B15	0.736368	-0.20936	0.884985
HMGCS2	0.11387	-0.86399	0.385201
ALDH18A1	0.213733	0.270164	0.509228
NAPA	0.689846	0.012621	0.859429
ADPRH	0.015909	0.670595	0.153751
SLURP1	0.331588	-0.19317	0.619341
MFAP2	0.039276	0.487243	0.236232
AIF1	0.520146	0.094119	0.760272
EIF5	0.037428	0.289312	0.231646
SLC12A2	0.35064	0.521113	0.635657
PSMD4	0.595428	0.111869	0.806337
DRG2	0.850885	-0.15164	0.944945
GEM	0.872446	-0.16385	0.956723
RRAD	0.560457	-0.22556	0.787589
NR1H2	0.064027	1.117956	0.296208
APOC4	0.091968	1.255598	0.345653
PLTP	0.083358	0.984334	0.332962
CSE1L	0.064284	0.283058	0.296208
TMBIM6	0.002419	1.570053	0.053552
VCP	0.274985	0.079764	0.577625
MFAP1	0.786816	0.369762	0.911452
MFAP3	0.65015	0.138641	0.839222
MFAP4	0.419073	0.208333	0.688796
HADHB	0.149515	-0.16862	0.438811
AQP4	0.009012	-2.77505	0.115288
INHBC	0.868337	-0.0335	0.954766
GDF10	0.446588	-0.67815	0.707261
MANF	0.563832	0.079259	0.789479

MTTP	0.932488	-0.18174	0.984157
NCKAP1L	0.32085	0.137136	0.613139
MLLT4	0.236413	-0.23212	0.537858
MLLT6	0.647413	-0.30026	0.83786
ELL	0.172232	0.699444	0.463535
BRPF1	0.626168	-0.24825	0.828579
NAP1L1	0.033952	0.290487	0.221141
CASP7	0.689843	0.024164	0.859429
CASP9	0.041387	1.54744	0.240826
CASP6	0.997369	-0.07153	1
GP2	0.664325	0.011648	0.847289
ADK	0.960544	0.033827	0.994122
ADAR	0.048208	0.311852	0.257798
LAMB2	0.050495	-0.65886	0.263221
CDKN2D	0.960709	0.175128	0.994122
CDH6	0.163211	-0.78212	0.453012
CDH11	6.33E-06	1.06514	0.000675
CDH13	0.12513	0.353113	0.401334
FOXA1	0.151388	1.388897	0.44125
TPD52	0.533067	-0.1489	0.7709
PRMT2	0.996791	0.025345	1
PKNOX1	0.008095	1.879309	0.107782
SEC13	0.199513	0.146587	0.49217
NHP2L1	0.61848	0.040578	0.822194
PAX9	0.405729	0.5123	0.679973
CCL18	0.475884	0.458329	0.728908
NPEPPS	0.343873	0.131921	0.630156
GFER	0.716758	0.181659	0.873394
HNRNPH2	0.374984	0.065697	0.656862
OXCT1	0.06524	0.492796	0.298136
SUMO3	0.985711	0.134142	1
EIF3B	0.047465	0.206069	0.255923
FCGRT	0.286754	0.209524	0.590053
BID	0.027122	0.90373	0.199082
ATP5J2	0.43056	0.166977	0.695651
GFRA1	0.210617	-0.43628	0.506604
NDUFV3	0.802262	0.392558	0.91835
RRP1	0.354009	-0.48435	0.638094
MARS	0.292764	0.163665	0.595788
ITGA1	0.799534	-0.12243	0.916173
CTSW	0.954108	0.083927	0.993074
ARPP19	0.997359	0.029904	1
MAZ	0.640497	-0.23305	0.835736
CMC4	0.481393	-0.59888	0.733586
MTCP1	0.163438	-0.20557	0.453012
TCL1A	0.753221	0.142809	0.894143
POLE2	0.675707	0.217181	0.85327
AP1S2	0.513557	0.201031	0.75602
ATP5I	0.699592	0.218026	0.864146
LGALS4	0.384279	-1.04035	0.66413
HDAC4	0.188175	1.157233	0.480067
EIF6	0.055344	0.251176	0.274516
CAV3	0.024484	-0.68695	0.189451
CTBP2	0.055344	0.302861	0.274516
NDUFA6	0.581788	-0.07796	0.797998
TMEM50B	0.97632	0.054563	0.998921
ARL4C	0.001796	1.709253	0.044352
PEX3	0.20772	0.35336	0.502361

NME5	0.688686	-0.17831	0.859429
WNT3	0.188151	-0.49543	0.480067
WNT4	0.739224	-0.26758	0.886253
WNT7B	0.37661	-0.35532	0.658208
PRSS12	0.993518	0.017115	1
CLDN6	0.132741	0.916693	0.415416
CLDN12	0.857343	-0.23036	0.948129
CLDN18	0.253801	-1.01045	0.557407
HSD17B7	0.960544	0.194972	0.994122
BCAR1	0.243003	0.434017	0.546608
STX17	0.342181	0.01162	0.629606
SLC37A1	0.17188	0.590868	0.463149
RWDD2B	0.367073	-0.29014	0.649965
UBASH3A	0.235307	0.270365	0.537436
C21orf59	0.079693	1.002375	0.325919
RIPK4	0.852223	0.201945	0.945958
WDR4	0.039072	1.29632	0.236232
TBX4	0.269746	-0.22313	0.572146
JAM2	0.358069	-0.39921	0.642301
TMEM33	0.861239	0.01083	0.950165
SYNJ2BP	0.759058	-0.04272	0.896847
GEMIN4	0.130825	0.615279	0.412798
PCBP3	0.934273	-0.6929	0.984157
PCBP4	0.986393	-0.07302	1
RAB38	0.287701	-0.63404	0.591234
RAB25	0.382122	-0.62502	0.661698
CORO7	0.004905	0.44577	0.082133
TCTA	0.331588	-0.17501	0.619341
CLDN2	0.207643	0.792725	0.502361
NUP107	0.05791	0.274277	0.280499
GSDMD	0.190424	0.243159	0.483515
SNX16	0.126059	-1.12457	0.403148
EEFSEC	0.694713	0.109571	0.861478
FBXW4	0.105925	-0.37871	0.370605
SESN2	0.91984	0.086203	0.97992
SESN3	0.543052	0.376001	0.776459
EPPK1	0.830256	-0.15329	0.933615
INHBE	0.596678	0.318019	0.807535
LOXL3	0.213674	0.542405	0.509228
ZNF121	0.013334	1.07165	0.141381
ANTXR2	0.117158	0.761668	0.389929
MLF1	0.202197	0.525115	0.495704
ADAMTS12	2.71E-08	2.904706	8.36E-06
FAM3B	0.946549	-0.02625	0.989647
SMIM11	0.397548	0.483767	0.675017
MTPN	0.871617	-0.18559	0.956098
YBEY	0.083995	1.214214	0.334503
EVA1C	0.346041	-0.42728	0.632606
TIRAP	0.15526	0.223378	0.444958
HIST1H2BD	0.375606	-0.55686	0.657384
RAB15	0.030181	1.516949	0.209418
DEFA3	0.280828	-0.72707	0.583434
GNG2	0.887225	-0.27307	0.96416
AP3S2	0.276129	-0.69381	0.579478
DNAJB13	0.518766	0.295883	0.760272
ARPC4	0.142204	0.133091	0.428816
ELOF1	0.188642	0.81576	0.480971
DEFB1	0.563678	-0.20924	0.789479

CD81	0.76912	0.108411	0.901915
SEC61G	0.009444	0.446452	0.117552
TPI1	0.272095	0.171689	0.575039
PLP1	0.57907	-0.10245	0.796675
EIF3E	0.419078	0.027508	0.688796
SEC61B	0.45805	0.21704	0.715224
PTEN	0.512959	0.390273	0.755743
PPP4C	0.133456	0.238704	0.416228
GABARAPL2	0.132239	0.827623	0.414428
ROMO1	0.138652	0.759868	0.424105
UBE2G2	0.066208	0.495793	0.300455
MYL6	8.33E-05	0.44706	0.005438
ACTB	0.001634	0.21248	0.041715
RAC3	0.964227	0.041281	0.99616
EIF4A1	0.030755	0.319383	0.211475
RPS20	0.660922	-0.09263	0.844596
SNAP25	0.300882	0.230812	0.602797
PRPS1	0.581788	-0.11955	0.797998
PSMA6	0.784286	0.081503	0.909506
S100A10	0.641914	-0.15327	0.835764
CDC42	0.835403	0.002637	0.935999
DSTN	0.784286	-0.02172	0.909506
GMFB	0.825116	0.008033	0.930395
RAB8A	0.61848	0.132245	0.822194
SPCS3	0.090665	0.320379	0.343901
SRP54	0.871617	-0.01278	0.956098
RAB4B	0.389353	-0.09898	0.667643
RAB2A	0.819984	0.002444	0.92687
RAB5B	0.503174	0.010466	0.747596
CKS1B	0.325226	0.63783	0.615753
RAB10	0.114161	0.340789	0.385201
CXCR4	0.827576	-0.08202	0.932505
UBE2D3	0.675324	0.266039	0.853175
UBE2M	0.064284	0.292154	0.296208
UBE2K	0.167008	0.421287	0.457139
UBE2N	0.411524	0.081586	0.683152
RAB14	0.094502	0.127742	0.349959
ZC3H6	0.598967	0.500091	0.809942
ACTR3	0.010002	0.233053	0.120967
ACTR2	0.005656	0.290973	0.088608
ACTR1A	0.223612	0.133571	0.521792
TMEM258	0.327323	0.22122	0.617719
COPS2	0.997369	0.04541	1
ABCE1	9.56E-05	0.467009	0.006126
RAP1B	0.694713	0.054116	0.861478
RAP2B	0.039909	0.278515	0.23845
MAX	0.789154	-0.29046	0.911483
RPS3A	0.739057	-0.07936	0.886253
RPL26	0.169043	-0.47131	0.459676
STX1B	0.300882	0.283482	0.602797
PSME3	0.015629	0.418586	0.152856
RPL15	0.367925	0.121253	0.650718
MAGOH	0.074384	0.257715	0.317894
RPL27	0.646644	0.058686	0.837353
ATP6V0D1	0.799534	-0.03601	0.916173
PCBD1	0.382127	-0.07357	0.661698
RPL37A	0.280828	0.047864	0.583434
RHOA	0.102567	0.155982	0.364372

RND3	0.140925	0.719851	0.42775
NAA20	0.033706	1.191417	0.221141
NCALD	0.915947	-0.30518	0.977664
HSPE1	0.010791	-0.4397	0.126291
SEC61A1	0.027821	0.311952	0.202072
LYZ	0.490639	-0.24411	0.738656
VBP1	0.051278	0.283187	0.265139
STXBP1	0.809743	0.079057	0.921586
B2M	0.185996	-0.27207	0.478129
DAD1	0.00841	0.41394	0.110127
TGFB2	0.1647	0.92767	0.454525
NPC2	0.454064	-0.06854	0.712914
COPZ1	0.075462	0.249236	0.320055
GNG11	0.150898	-1.43259	0.44125
SUMO2	0.997368	-0.31987	1
UFM1	0.05451	0.330172	0.273313
DCAF7	0.161013	0.146637	0.453012
WDR5	0.28378	0.136147	0.586874
AP1S1	0.641914	-0.13238	0.835764
NUTF2	0.709387	-0.07423	0.869179
HNRNPK	0.533067	0.080207	0.7709
YWHAG	0.298858	0.15307	0.601676
USP46	0.294284	0.46593	0.597457
RRAS2	0.251082	-0.29706	0.554953
TIMM10	0.97632	0.067562	0.998921
TSPAN5	0.410792	0.423641	0.683152
RPS7	0.071226	0.125384	0.311134
PPP1CA	0.102567	0.11087	0.364372
PPP1CB	0.078771	0.165294	0.32578
NCS1	0.044372	1.511983	0.24851
PSMC1	0.084549	0.184891	0.335025
PSMC5	0.034511	0.168998	0.222148
RPS8	0.26355	0.027415	0.565873
RPS15A	0.438315	0.025073	0.701933
RPS16	0.675323	-0.26879	0.853175
UBE2G1	0.050419	0.880726	0.263221
UBE2H	0.060535	0.715892	0.287134
YWHAE	0.037428	0.214851	0.231646
RPS14	0.819984	-0.1005	0.92687
RPS23	0.524435	0.029119	0.764023
RPS18	0.577274	-0.06757	0.796634
RPS29	0.882011	0.331613	0.961515
RPS13	0.272095	0.084614	0.575039
RPS11	0.699592	-0.16018	0.864146
SNRPE	0.830256	-0.05534	0.933615
SNRPF	0.702009	-0.69056	0.866168
SNRPG;SNRPGP15	0.929044	-0.02082	0.983115
LSM3	0.646644	-0.10726	0.837353
LSM6	0.507385	-0.79047	0.75153
SNRPD1	0.680152	-0.03928	0.85545
SNRPD2	0.32085	0.137117	0.613139
SNRPD3	0.255193	0.187413	0.557593
TMSB4X	0.049723	-0.87369	0.261364
ARF6	0.434427	0.111687	0.698194
PSMC6	0.175259	0.112501	0.466796
SELT	0.902877	0.01905	0.971583
TBPL1	0.948734	-0.17755	0.990863
RPL7A	0.014531	0.113855	0.147184

POLR2G	0.330585	0.72401	0.619341
RAB11A	0.623137	0.055545	0.825978
ETF1	0.175259	0.230991	0.466796
ESRRG	0.322009	0.17379	0.613139
CNBP	0.939534	-0.11011	0.986498
YPEL5	0.310008	-0.43506	0.609864
RPS4X	0.779221	-0.13662	0.906856
PPP2CB	0.636994	-0.56647	0.833511
ACTA2	0.546146	0.533769	0.778738
RHOB	0.934287	0.175252	0.984157
RPL23A	0.289748	0.234887	0.593134
RPS6	0.554955	-0.18414	0.783439
VSNL1	6.45E-07	3.963015	0.000105
HIST1H4A	0.931332	-0.00333	0.984157
HIST1H2BC;HIST1H2BK;H2BFS	0.127815	-1.67494	0.407004
RAB1A	0.324076	0.207625	0.614309
RAN	0.407778	0.076856	0.680612
RPL23	0.093209	-0.06143	0.347256
RAP1A	0.981581	0.0411	1
UBE2D2	0.422274	0.53999	0.691467
RPS15	0.098469	0.081408	0.357719
RPS24	0.117214	0.161513	0.389929
RPS25	0.216173	0.098303	0.512741
RPS26;RPS26P11	0.067188	0.590806	0.302058
RPS28	0.228671	0.228416	0.528484
FAU	0.091605	0.084548	0.345626
GNB1	0.396661	0.111976	0.67382
POLR2L	0.350639	0.652809	0.635657
RBX1	0.568292	0.307566	0.791838
GNB2	0.799534	-0.07225	0.916173
RPL30	0.171096	-0.01617	0.461822
RPL31	0.744041	0.063564	0.888303
RPL10A	0.825116	-0.02035	0.930395
RPL32	0.595428	-0.25263	0.806337
RPL11	0.590866	0.041041	0.804875
RPL8	0.528742	-0.32848	0.767576
PPIA	0.426712	-0.08244	0.693599
FKBP1A	0.850885	-0.04063	0.944945
RPS27A;UBB;UBC	0.81486	0.041084	0.924847
UBA52	0.845785	-0.014	0.942239
GRB2	0.759058	0.043767	0.896847
TRA2B	0.035653	0.335513	0.225452
RAC1	0.81486	-0.06305	0.924847
AP2B1	0.00023	0.257306	0.011713
VAMP2	0.876813	0.241486	0.958859
GNAS	0.289748	0.146952	0.593134
GNAI1	0.12841	-0.20255	0.407846
PPP3R1	0.204174	0.189695	0.497963
YWHAZ	0.093209	0.225931	0.347256
UBE2B	0.406996	0.734585	0.680612
PPP2R2A	0.133456	0.241674	0.416228
SNRPN	0.396661	0.108711	0.67382
SUMO1	0.729121	0.229575	0.879656
DYNLL1	0.438315	0.065246	0.701933
DYNLT1	0.929044	0.114484	0.983115
RPL38	0.33388	0.065277	0.620902
SKP1	0.699592	0.01148	0.864146
GNG5	0.244338	0.303019	0.547425

RPS21	0.646644	-0.04785	0.837353
EIF5A;EIF5AL1	0.1068	0.171205	0.371671
GNB2L1	0.004424	0.244661	0.076312
ACTG1	0.474191	0.152709	0.727018
ACTG2	0.044471	1.165008	0.24851
SUPT4H1	0.955125	-0.14468	0.993074
UBE2I	0.586319	0.047724	0.801141
SEPW1	0.1787	1.19301	0.47055
TMSB10	0.382127	0.107308	0.661698
TNNC1		0	
PPP2CA	0.1882	0.15694	0.480067
YBX1	0.061485	0.347003	0.289565
SEC11A	0.126762	0.214041	0.403998
CSNK2B	0.04253	0.300213	0.243142
TPM4	0.000191	0.576472	0.010256
ACTC1	0.039276	0.324951	0.236232
UBE2L3	0.923804	0.056126	0.980751
EEF1A1;EEF1A1P5	0.1068	0.150787	0.371671
ACTA1	0.058974	0.743072	0.283643
TUBA1B	0.020421	0.268681	0.170514
TUBA4A	0.586319	0.070199	0.801141
TUBB4B	0.024706	0.265153	0.190022
CSNK2A1	0.434427	-0.01582	0.698194
PAFAH1B2	0.794443	0.058089	0.913708
HIST1H3A;HIST3H3;H3F3C	0.623127	0.70148	0.825978
UBXN2A	0.375606	-0.48683	0.657384
HBB	0.462013	-0.09712	0.718747
HBG1	0.16297	-1.14114	0.453012
HBG2	0.267796	-0.93585	0.570195
HBA1	0.658897	-0.1105	0.843859
CXADR	0.100661	-0.87416	0.361539
FAM193A	0.093082	0.551047	0.347256
SH3BP2	0.09103	0.803001	0.344815
NOP14	0.199309	0.549476	0.49217
IGBP1	0.012537	0.723014	0.136468
SIRPA	0.520146	0.193169	0.760272
ADAM8	0.059989	1.37548	0.286703
CYP4F2	0.079293	0.318702	0.32578
PSPH	1	-0.16112	1
RBM6	0.003951	1.418433	0.071362
EIF4G2	0.015629	0.305533	0.152856
RPP38	0.688054	-0.21801	0.85937
RPP30	0.005656	0.881343	0.088608
GTF2I	0.632496	-0.0282	0.830965
PIP4K2B	0.008168	0.601944	0.108493
CNTNAP1	0.001516	1.671301	0.03994
SRPK2	0.029993	0.146193	0.209097
PHC1	0.27536	0.299033	0.578109
CSNK1G2	0.542931	-0.36361	0.776386
CLDN10	0.873013	0.160267	0.956872
CCT2	0.371444	-0.08064	0.65423
OLR1	0.312494	0.360376	0.610732
SLC35A2	0.665637	0.048833	0.847351
SLC35A1	0.370809	-1.0247	0.65423
SLC35B1	0.015013	2.132438	0.150049
KRT85	0.996791	-0.15656	1
RAE1	0.14042	0.233058	0.426568
BTN3A2	0.155704	-0.84776	0.445884

GSTO1	0.892438	0.042516	0.966699
CX3CL1	0.075543	0.816902	0.320278
JAG1	0.224923	0.341065	0.52386
ST5	3.42E-05	1.70433	0.002581
PRKDC	0.016204	0.2484	0.154611
ADAM17	0.019715	0.582544	0.1684
BLOC1S1	0.106766	0.637118	0.371671
SRPX	0.017671	1.645582	0.161012
ARG2	0.123603	0.72749	0.399439
ELF3	0.894777	-0.24909	0.968678
NTHL1	0.438526	-0.49106	0.701933
IL13RA1	0.449841	0.206967	0.709598
CCL20	0.200937	1.120316	0.494254
MAP1A	8.70E-07	2.020629	0.000136
CRADD	0.000816	2.334479	0.027022
PHEX	0.140267	-0.72101	0.426568
ADARB1	0.070106	0.948935	0.309184
HLA-DRB3	0.86966	0.650067	0.955842
GPLD1	0.841655	-0.08338	0.940155
LCN2	0.575013	-0.69871	0.796599
IFI35	0.327323	0.188689	0.617719
MT1X	0.011262	2.419287	0.12877
NUCB2	0.199513	0.196558	0.49217
HSD11B2	0.527568	0.32722	0.767277
DLK1	0.319672	0.151316	0.613139
ABAT	0.484434	0.202045	0.735121
S100A12	0.762223	0.695592	0.899242
BASP1	0.002	0.697048	0.047089
IGLV3-21	0.876747	-0.24903	0.958859
HAMP	0.433996	0.445878	0.698194
DCD	0.405567	-0.86668	0.679973
SSBP2	0.528824	0.34954	0.767592
TMF1	0.11568	0.357861	0.387731
MRPS22	0.169043	0.225397	0.459676
MRPS25	0.013753	0.607124	0.143228
MRPS10	0.007931	1.136344	0.107292
MRPS35	0.164991	0.068208	0.454525
MRPS5	0.902862	-0.1217	0.971583
MRPS36	0.06524	-0.12867	0.298136
MRPS11	0.366062	0.461011	0.648976
MRPS15	0.366062	-0.62823	0.648976
MRPS21	0.789328	0.131714	0.911483
MRPS34	0.871617	-0.03405	0.956098
MRPS6	0.923803	-0.24584	0.980751
MRPS9	0.024286	0.325317	0.188496
HMGN5	0.678898	-0.0463	0.855378
SARNP	0.809743	0.07959	0.921586
RBP5	0.322796	-0.6702	0.613707
ADAMTSL3	0.956936	-0.1293	0.994122
HTRA4	0.995826	-0.04281	1
HTRA3	7.06E-05	1.603373	0.004747
LACTB	0.05791	0.336076	0.280499
COG7	0.204174	0.349166	0.497963
RPL24	0.507389	-0.09112	0.75153
TXNL4A	0.520106	0.455367	0.760272
RPL36A	0.850121	0.169233	0.944945
CBX1	0.008743	0.392463	0.112752
SMAD3	0.269225	0.580366	0.571371

ARF1	0.830256	0.078109	0.933615
ARF5	0.179497	-0.29434	0.471072
ERH	0.684992	0.082767	0.85709
RHOG	0.1068	0.14851	0.371671
RPL19	0.819984	-0.15078	0.92687
SERF2	0.741942	0.380414	0.887847
SRSF3	0.660922	0.140313	0.844596
MXRA7	0.249719	0.727093	0.552833
H3F3A	0.749036	-0.22569	0.890902
FOXK1	0.015911	0.770297	0.153751
ARHGAP8	0.642252	0.191817	0.835764
PRR5	0.141292	0.520032	0.427981
RPP14	0.243159	0.870272	0.546811
ZBED6	0.045973	0.36574	0.252361
CCZ1;CCZ1B	0.002334	0.397249	0.052291
TNFAIP6	0.229479	0.399101	0.529537
DAB2	0.05207	0.494144	0.267367
MUC5AC	0.482512	-0.8305	0.73359
FBLN2	6.74E-06	0.888564	0.000698
DGCR2	0.207717	0.754492	0.502361
VLDLR	0.116461	0.596015	0.389223
HSPG2	0.554955	0.01139	0.783439
LRP2	0.318749	-0.53223	0.613139
XIAP	0.006131	0.568411	0.092018
ARHGAP4	0.247021	0.220062	0.549922
EFNB1	0.881497	-0.10941	0.961515
FAM3A	0.710351	0.567646	0.870073
RBM10	0.171096	0.348982	0.461822
RBM3	0.138653	0.404365	0.424105
ATP2C1	0.739057	0.070342	0.886253
ATP11A	0.101185	-0.76564	0.361901
ATP8B2	0.095134	0.494954	0.35136
CYCS	0.177368	0.334491	0.468602
MPP1	0.392997	0.041237	0.671013
TFAM	0.533062	-0.0824	0.7709
PITPNA	0.057044	0.450237	0.278658
MAT1A	0.686212	-0.40621	0.857929
SLC25A3	0.840557	-0.02946	0.939212
HDLBP	2.69E-05	0.509386	0.002129
GTF2B	0.380288	-0.24276	0.661698
CDK6	0.317161	0.3412	0.613139
CDK5	0.577274	0.260241	0.796634
CDK16	0.436369	0.303189	0.700907
CDK17	0.000528	1.393135	0.02098
PURA	0.078771	-0.1883	0.32578
CDC42EP1	0.000672	1.629799	0.024319
CLTC	0.062406	0.179354	0.292127
HSF1	0.003025	1.070534	0.062294
NFKB2	0.018697	0.436373	0.165633
FKBP3	0.520146	0.093558	0.760272
PLCB2	0.088785	0.275861	0.34076
REEP5	0.794443	-0.04295	0.913708
SORD	0.330591	-0.28716	0.619341
HNRNPU	0.804634	0.114292	0.918848
IRF9	0.013253	1.162558	0.141015
SPTBN1	0.324076	-0.1143	0.614309
TIAL1	0.005322	0.36522	0.086174
E2F1	0.092612	0.566786	0.347013

SET	0.066208	0.396426	0.300455
SCN7A	0.069187	-1.22228	0.306571
SRSF2	0.714303	-0.02164	0.87164
FOXK2	0.059733	1.124566	0.285807
RUNX1	6.97E-05	0.834763	0.00473
RELB	0.059395	0.885309	0.285461
MS4A2	0.540968	0.277723	0.775521
GALK2	0.280717	0.24004	0.583434
AMPD3	0.204174	0.243281	0.497963
AMPD2	0.00719	0.404937	0.101457
CTBS	0.101865	-0.75761	0.363392
FABP5	0.486499	-0.08452	0.73612
ANK2	0.845668	-0.13403	0.942239
CAP1	0.426712	0.122977	0.693599
DEFA5	0.784838	0.227759	0.909861
FLI1	0.138535	0.996665	0.424105
HMGCS1	0.723762	0.835513	0.876654
IFITM3	0.000261	1.627079	0.012879
IL1RL1	0.331588	-0.08594	0.619341
SLC7A5	0.086339	0.288327	0.33706
DR1	0.001363	0.583282	0.036688
TFAP4	0.273877	0.689728	0.577625
FMO1	0.014952	1.856881	0.150049
EXOSC10	0.185996	0.240829	0.478129
OTUD4	0.147477	0.53409	0.436386
PFKP	0.09581	0.401936	0.352331
XPC	0.183786	-0.52408	0.474829
EWSR1	0.120332	0.268975	0.39497
CDR2	0.701336	0.241921	0.865627
COL4A3	0.534725	-0.27778	0.772118
OCRL	0.030249	0.609972	0.209418
PLCB3	0.466081	0.08455	0.721065
ROR1	0.086969	0.921794	0.337854
ROR2	3.02E-07	2.928069	6.08E-05
TAGLN	4.64E-05	0.836462	0.003385
AKAP17A	0.354029	0.568188	0.638094
MYL5	0.322009	0.13512	0.613139
MEF2A	0.57907	-0.10245	0.796675
NAAA	0.773946	-0.23244	0.903913
SP2	0.238305	0.492646	0.540624
RHAG	0.203986	0.760343	0.497963
GUCY1A3	0.134935	0.525546	0.417833
DHODH	0.779219	-0.08391	0.906856
GUCY1B3	0.590866	0.110792	0.804875
PRKCE	0.233658	-0.40975	0.535403
OGDH	0.14042	-0.18438	0.426568
TNFRSF17	0.655601	-0.27999	0.84209
KIF23	0.096067	1.012272	0.352925
ALDH6A1	0.0334	-0.38383	0.219585
NRG1	0.079293	0.392277	0.32578
CYP27A1	1	0.03059	1
BDH1	0.038652	-0.63317	0.235412
SEMG2	0.09909	-0.63591	0.358568
COL7A1	0.211261	0.829001	0.506632
APBA1	0.483801	0.430055	0.735071
DSG1	0.186803	0.726749	0.479162
SP4	0.711754	0.149531	0.870161
SP3	0.063323	1.546328	0.294055

DSC2	0.604318	0.153527	0.813002
MUC3A	0.310183	-0.23894	0.609864
HIST1H1A	0.046096	1.280553	0.252472
RPL18A	0.257958	-0.24037	0.560462
IRF8	0.042978	1.112263	0.245328
GCNT1	0.175825	0.858815	0.46742
MAP2K1	0.048208	-0.56526	0.257798
TEK	0.972173	-0.12571	0.997906
FKBP4	0.759058	-0.05906	0.896847
PLOD1	1.80E-08	1.182293	6.25E-06
MUC2	0.488557	0.369091	0.738428
NUCB1	0.32085	0.14899	0.613139
RASSF7	0.661575	0.282453	0.84482
RPL6	0.908103	-0.17311	0.9734
TOP2B	0.167008	0.177415	0.457139
CREB5	0.992139	-0.03236	1
AKAP12	0.018368	0.505706	0.163766
SLC25A11	0.660922	0.043393	0.844596
CFHR3	0.119848	-1.15225	0.394783
DST	0.000172	1.077355	0.009509
GSTM4	0.315992	0.834672	0.613139
CREM	0.657906	-0.14997	0.842898
MLLT1	0.024774	1.357552	0.190411
MECOM	0.294609	-0.54036	0.597898
GNA12	0.090519	1.07935	0.343901
CAV1	0.537409	-0.24869	0.773175
ACY1	0.108187	-0.09016	0.374055
KMT2A	0.902845	0.043847	0.971583
TGFBR3	0.86384	0.010168	0.951898
TNFAIP2	0.121915	0.520025	0.397343
CENPC	0.025634	1.350279	0.193538
LMNB2	0.950035	0.059078	0.990863
PTS	0.20074	0.506599	0.494233
PLAUR	0.000796	1.717703	0.026879
MVK	0.950034	0.33715	0.990863
ERCC6	0.803788	-0.21688	0.918848
TAP1	0.247021	0.253574	0.549922
TAP2	0.305036	0.251528	0.606083
CFHR1	0.044589	0.637076	0.24851
COL10A1	0.000557	2.829539	0.02176
CEBPZ	0.274985	0.167574	0.577625
RELA	0.407778	0.12075	0.680612
UBXN1	0.044589	0.47953	0.24851
GBE1	0.646644	0.037904	0.837353
FOLH1	0.000327	2.128385	0.015317
EIF4G1	0.016499	0.293838	0.155558
ATP7A	0.902874	-0.03192	0.971583
KRT17	0.656149	0.082396	0.84209
NOTCH2	0.110397	0.556508	0.378842
TLE1	0.426641	0.381782	0.693599
TLE2	0.416636	-0.21103	0.687635
TLE3	0.286748	0.554392	0.590053
HGFAC	0.912712	0.023793	0.975706
PRKCQ	0.475114	-0.38083	0.728131
GLO1	0.81486	0.045062	0.924847
ACVR1	0.674864	0.178564	0.853175
HLA-B	0.479044	-0.42856	0.731627
AKR1C1	0.206491	-0.96644	0.500256

SSBP1	0.559385	-0.19228	0.78633
REL	0.87161	0.100935	0.956098
MST1R	0.389976	0.528474	0.668401
YWHAH	0.050495	0.242934	0.263221
PLP2	0.374911	0.65564	0.656862
CSTF1	0.05451	0.336195	0.273313
ICA1	0.354007	-0.20055	0.638094
UBE3A	0.213733	0.399027	0.509228
DNM1	1.20E-07	2.142944	2.71E-05
PTPN12	0.015348	0.577973	0.151713
CLC	0.41184	0.920301	0.683152
PTK2	0.511623	0.139302	0.754376
LIPE	0.013628	0.996127	0.14262
ZNF91	0.683128	0.146126	0.85709
PRKCZ	0.064541	-1.04812	0.296805
ZBTB16	0.988991	0.063427	1
SRSF11	0.015629	0.399297	0.152856
EEF1A2	0.083056	1.596682	0.332962
PRKCD	0.311298	-0.23669	0.609894
CALD1	3.49E-09	1.109535	2.42E-06
COL14A1	0.426712	0.157151	0.693599
RNASEL	0.191531	0.61063	0.485658
FPGS	0.475812	0.59547	0.728899
SLC18A2	0.31899	0.280966	0.613139
MBTD1	0.306086	0.158809	0.607626
CTDSPL2	0.564362	0.586099	0.790121
FAM160A1	0.267466	-0.48829	0.570195
ITIH3	0.442197	0.636828	0.703704
PTPN11	0.053686	0.216552	0.271145
KDSR	0.411524	-0.00986	0.683152
REG3A	0.988567	0.009614	1
BTK	0.804632	-0.10119	0.918848
PPP2R3A	0.062595	0.689434	0.29268
PPAT	0.059672	0.356565	0.285693
GFPT1	0.699592	0.066639	0.864146
EXOSC9	0.078755	0.966146	0.32578
AOX1	0.296535	-0.73319	0.599275
PSME1	0.646644	-0.07622	0.837353
RBPJ	0.371444	0.171945	0.65423
MEF2C	0.157368	1.147325	0.447293
TYRO3	0.178261	0.223037	0.469506
APLP2	0.960544	-0.21753	0.994122
GABPA	0.897655	-0.04233	0.969793
GABPB1	0.36297	0.610486	0.646802
RING1	0.675324	0.06696	0.853175
FMR1	0.159052	0.330921	0.449897
FMOD	3.16E-06	2.104476	0.000377
PRDX1	0.360948	-0.13723	0.644411
HLA-C	0.482377	0.088373	0.733586
CDK18	0.200159	0.860935	0.493326
RPL18	0.255191	0.14765	0.557593
C1QBP	0.076552	0.252813	0.322705
CKAP4	4.26E-05	0.608203	0.00315
ENPEP	0.067173	0.951042	0.302058
COL16A1	0.023298	1.153484	0.18431
CD69	0.260391	-0.87361	0.56373
TJP1	0.709387	0.051291	0.869179
CXCL9	1	-0.04159	1

PDE4B	0.733732	-0.0786	0.882858
ZFP36L1	0.002793	2.075751	0.059562
DPT	0.734083	-0.05286	0.882858
SPAG1	0.603247	0.257238	0.813002
TFF3	0.293581	-0.87927	0.596247
KHDRBS1	0.072266	0.302539	0.313701
BAX	0.2958	0.174522	0.598344
BCL2L1	0.583348	0.362757	0.799544
MCL1	0.143484	1.221458	0.43162
SLC3A1	0.181133	-0.33622	0.472575
POLE	0.031311	1.239025	0.212999
KLC1	0.019032	0.324248	0.166597
SOS1	0.012403	1.102039	0.135929
SOS2	0.212175	0.475769	0.508038
TNK2	0.488022	0.229237	0.738022
LRP1	0.002	0.546076	0.047089
SRSF1	0.623137	0.119769	0.825978
ARHGAP1	0.000528	0.418202	0.02098
CYP24A1	0.068539	0.973369	0.305289
AES	0.063557	1.417965	0.29482
SRSF4	0.81486	-0.14306	0.924847
PCDH1	0.660672	-0.08698	0.844596
TGM3	0.571349	0.062786	0.793891
PPP3CA	0.454064	0.131361	0.712914
DHX9	0.049723	0.201402	0.261364
CRYZ	0.109698	-0.28151	0.377423
IL10RB	0.171491	0.499208	0.462438
DDR1	0.182991	-0.47305	0.474175
SLC20A2	0.566553	-0.50916	0.791838
GOLGA3	0.043893	0.256994	0.247494
GOLGA2	0.486499	0.095197	0.73612
LGALS3BP	0.118765	0.342353	0.392409
LOXL1	3.45E-07	3.233771	6.59E-05
EHHADH	0.72417	-0.114	0.876654
MFGE8	0.00511	0.91294	0.083708
CYP4F3	0.497809	0.406927	0.744799
DMTN	0.186908	-0.90959	0.479162
PDE4D	0.514015	-0.53433	0.756193
DSC1	0.221132	0.602219	0.519064
HDHD1	0.206699	-0.99434	0.500547
CD47	0.247021	-0.22509	0.549922
PPID	0.244338	0.285931	0.547425
FGL1	0.631585	-0.10554	0.830965
SSRP1	0.004517	0.438731	0.077667
RBL2	0.303767	0.350757	0.605949
CAMSAP2	0.007817	1.267534	0.106243
SPIRE1	0.491645	0.240325	0.739769
SLFN5	0.050495	0.194976	0.263221
MZT1	0.259673	0.842576	0.563057
C2orf54	0.357796	-0.63746	0.641914
VAC14	0.02152	0.379588	0.175496
SIGLEC14	0.331588	-0.14096	0.619341
NSUN2	0.438315	0.164234	0.701933
RBBP4	0.05207	0.35676	0.267367
NCBP1	0.047465	0.277825	0.255923
MGAT3	0.464202	-0.2857	0.721065
MGAT5	0.000451	2.03127	0.019184
EP300	0.02152	0.545528	0.175496

AHNAK	0.120332	0.300202	0.39497
NIPAL4	0.300882	0.40929	0.602797
SLC10A7	0.026184	1.0305	0.194901
TBC1D8B	0.061618	1.079362	0.290071
FCHO2	0.595428	0.184113	0.806337
TMEM62	0.219293	0.820114	0.517045
ELP6	0.129468	0.8925	0.410218
APOBR	0.020414	0.857719	0.170514
HSPA14	0.053686	0.629551	0.271145
SCRN3	0.114883	0.710085	0.387168
C5orf28	0.927689	0.041418	0.983115
CGNL1	0.197206	-0.45206	0.490309
LAMTOR4	0.494732	0.39305	0.741997
NEXN	0.000168	1.117706	0.009401
MGAT2	0.086954	0.593057	0.337854
GALNT2	0.006131	0.581129	0.092018
GALNT1	0.001082	0.718422	0.031645
AP1B1	0.179497	0.154374	0.471072
CPSF1	0.161013	0.273891	0.453012
TEF	0.393617	0.252571	0.671013
BST1	0.274985	0.281648	0.577625
BST2	0.086954	-0.60329	0.337854
PMPCA	0.944783	-0.00337	0.988547
FUT2	0.500258	0.231266	0.745947
ST3GAL1	0.128525	1.128835	0.408017
ST3GAL4	0.002302	1.815009	0.051684
KIF1A	0.69435	0.090939	0.861478
SCRN1	0.520146	0.023936	0.760272
KIAA0195	0.791917	-0.09227	0.913596
KIAA0196	0.001272	0.319314	0.034924
NUP160	0.011856	0.345511	0.13136
SCAP	0.201224	0.591007	0.494631
SREBF2	0.576266	0.201218	0.796634
ARHGEF5	0.403328	-0.44263	0.678271
FOXO1	0.015821	0.840277	0.153751
TBL3	0.382127	0.162489	0.661698
GTF3C1	0.007479	0.518476	0.10395
KCNMA1	0.084236	0.834977	0.335025
TWF1	0.008089	0.416082	0.107782
HYAL1	0.988991	0.063968	1
ASPH	0.272095	0.240949	0.575039
TFCP2	0.038652	0.537221	0.235412
AKAP13	0.181644	-0.40845	0.472575
EFEMP1	0.000657	0.716306	0.023973
TRO	0.701126	0.17634	0.865518
SMARCB1	0.360948	0.130541	0.644411
BPTF	0.470024	0.141312	0.72456
CDC20	0.552775	0.369933	0.783393
FSTL1	0.001081	1.399461	0.031645
STX4	0.774166	0.134271	0.903913
GRSF1	0.595428	0.10852	0.806337
MAP4K2	0.027888	0.871393	0.202292
NFIA	0.56708	0.563657	0.791838
CNTN1	0.620263	-0.21668	0.823971
CDH17	0.340315	0.386829	0.627258
MERTK	0.568314	0.391842	0.791838
SFSWAP	0.002687	1.75797	0.057778
CHD3	0.001055	1.448861	0.031507

SF3A3	0.103963	0.229478	0.366863
DPYD	0.507389	0.311736	0.75153
FAP	1.49E-09	1.800866	1.33E-06
TP53BP1	0.000155	0.703092	0.008818
HYAL2	0.485484	0.444561	0.736044
TMEM115	0.460493	0.863822	0.71823
IFRD2	0.503183	-0.363	0.747596
TRIM26	0.213733	0.14943	0.509228
AIMP1	0.764084	-0.10119	0.899242
ILF2	0.002283	0.371082	0.051363
ILF3	0.045294	0.243862	0.250872
LMAN2	0.103963	0.184276	0.366863
LRMP	0.091269	1.014589	0.345435
PTPRJ	0.298858	0.027171	0.601676
PTPN13	0.443823	0.525546	0.705524
EPS8	0.462056	0.124101	0.718747
TRAP1	0.997369	0.060787	1
TRAF2	0.819974	0.083056	0.92687
FOXF1	0.309279	-0.48454	0.609864
ANK3	0.003368	-0.90518	0.066236
DLG1	0.572777	0.124493	0.794481
TAF10	0.350482	-0.37593	0.635657
MYO1E	0.000139	0.630914	0.008094
NFATC3	0.062711	1.34503	0.292924
PPP1R8	0.075462	0.351519	0.320055
PTP4A2	0.114161	-0.19321	0.385201
ABR	9.82E-05	0.665763	0.006224
NPRL3	0.529222	0.425787	0.767971
BNIP1	0.774166	-0.1094	0.903913
BNIP2	0.453061	-0.28403	0.712914
BNIP3	0.991673	-0.07014	1
NFX1	0.212057	0.606181	0.507864
CSTF3	0.051278	0.306967	0.265139
TSPAN31	0.1865	1.055787	0.479026
TIAM1	0.107511	-1.19276	0.372977
ECH1	0.286754	-0.03177	0.590053
MLLT11	0.15526	0.31804	0.444958
ARHGAP5	0.494757	0.047673	0.741997
PLA2R1	0.06058	0.434916	0.287134
MALL		0	
PRDM2	0.334741	0.390165	0.621773
STRN3	0.005543	0.466348	0.087699
CDC16	0.002185	0.680794	0.049962
STK4	0.041202	0.441823	0.240826
FLII	0.97632	-0.01834	0.998921
TRIM32	0.279358	0.162853	0.58196
COASY	0.434427	0.037756	0.698194
NAIP	0.059403	0.94617	0.285461
TRAF1	0.10601	0.766625	0.370631
MRPL28	0.360948	0.224819	0.644411
ACACA	0.623137	-0.21302	0.825978
PDIA2	0.464202	0.186337	0.721065
PLA2G7	0.983209	0.046427	1
LCP2	0.003514	0.651418	0.067901
GPS1	0.419078	0.101663	0.688796
IFT88	0.711652	-0.24603	0.870161
SPP2	0.909075	0.315727	0.97416
ZBTB17	0.799868	0.077258	0.916176

USP4	0.266378	0.319692	0.568807
CHAF1A	0.005005	1.19167	0.082994
CHAF1B	0.581366	0.335896	0.797998
PDZK1IP1	0.304249	0.65417	0.606083
TRAF3	0.002505	1.274747	0.055018
IK	0.466082	0.175921	0.721065
MTAP	0.660922	-0.01477	0.844596
RLF	0.302901	0.46843	0.604766
PRKAA1	0.218632	0.203387	0.515816
PPFIA1	0.030755	0.447011	0.211475
CALCOCO2	0.35405	-0.26232	0.638094
EIF2B5	0.71923	0.070938	0.874478
TARDBP	0.211314	0.21601	0.506632
HNRNPA0	0.017103	0.473379	0.157941
PAK1	0.61848	-0.08704	0.822194
AIMP2	0.845718	-0.03306	0.942239
FADD	0.249723	-0.59386	0.552833
PRDX4	0.277896	0.176772	0.579677
MAP2K5	0.684849	0.282396	0.85709
MAPK7	0.005132	1.950666	0.083955
PAK2	0.020781	0.262017	0.172048
SLC13A2	0.538865	-0.42031	0.774868
CBX3	0.680152	-0.08035	0.85545
STK3	0.086954	0.144827	0.337854
STX5	0.003027	0.572137	0.062294
CBLB	0.298466	0.469679	0.601676
PSMD2	0.171096	0.169085	0.461822
MMRN1	0.892045	-0.04698	0.966699
DDX10	0.636441	0.419981	0.833511
TBX2	0.685434	0.12092	0.857546
SEMA3B	0.820989	-0.19142	0.927911
DNAJC3	0.317645	0.126457	0.613139
PAPPA	0.167136	0.652808	0.457139
GPS2	0.38638	0.979834	0.665219
SELENBP1	0.014008	-0.86169	0.144006
CHIT1	0.121951	1.274683	0.397343
NME3	0.944783	-0.03178	0.988547
MAP3K1	0.739224	-0.27627	0.886253
PRKG2	0.464202	-0.24963	0.721065
SRSF9	0.197211	0.252013	0.490309
SRSF5	0.327323	0.448585	0.617719
SRSF6	0.876815	0.014309	0.958859
NOG	0.810389	-0.05112	0.921889
MAD2L1	0.088792	1.299956	0.34076
TRIM28	0.000125	0.503637	0.007549
DHRS2	0.48063	0.290183	0.73294
SEMA3F	0.845899	-0.0064	0.942239
STX3	0.460039	-0.56628	0.717621
G3BP1	0.000641	0.515312	0.023555
CLN3	0.693088	-0.89556	0.861478
NMI	0.277896	0.218444	0.579677
SLAMF1	0.36378	0.517764	0.646996
KCNAB2	0.064284	0.403434	0.296208
PTK7	0.000373	0.712506	0.01681
SKP2	0.731297	0.063822	0.881419
PABPC4	0.016204	0.302221	0.154611
ATM	0.089414	0.344305	0.341477
GRB10	0.032157	1.321803	0.215938

IFIT5	0.357491	0.115073	0.641472
SGCG	0.46384	-0.56867	0.721065
MTA1	0.071226	0.589015	0.311134
PTPRS	0.123028	0.719603	0.399387
SLC14A1	0.329349	-0.27813	0.619341
SP140	0.357155	0.466774	0.641472
EIF3I	0.112657	0.148532	0.382523
PPIL2	0.026439	0.936771	0.195939
MFAP5	0.611514	-0.13453	0.818417
PPP2R5C	0.03228	0.474828	0.215938
CTBP1	0.400346	0.130948	0.67622
AP3B2	0.455572	0.137884	0.71417
PDE3B	0.132292	0.486408	0.414476
PDCL	0.274443	0.81156	0.577625
PLD1	0.322989	0.491151	0.613929
TARBP1	0.162467	0.710942	0.453012
MYO7A	0.429641	-0.40888	0.695651
UBE2V1	0.631426	0.073808	0.830965
MRPL49	0.641914	0.2848	0.835764
DYNC1I2	0.794443	-0.0569	0.913708
ORC1	0.063318	0.676511	0.294055
ORC2	0.072566	0.73961	0.31451
ILK	0.017726	0.259712	0.161012
MSLN	0.896422	-0.05959	0.969793
IKZF1	0.264136	0.741669	0.566641
NNT	0.882017	0.037603	0.961515
SNTA1	0.702734	-0.28937	0.866291
SNTB2	0.986843	0.0785	1
XRCC4	0.007031	1.608402	0.100623
PPIG	0.340521	0.141848	0.627258
TCOF1	0.343873	0.256926	0.630156
UNC119	0.049522	1.109291	0.261364
SLC39A6	0.311853	0.647182	0.610732
SF3B2	0.112657	0.29308	0.382523
OS9	0.012079	0.471424	0.133041
GOLGA4	0.586319	0.221208	0.801141
PDAP1	0.360948	0.336343	0.644411
ADAM9	0.020065	0.558084	0.169555
ADAM15	0.96052	-0.23945	0.994122
TMED1	0.145824	0.144639	0.434185
LSAMP	0.426641	-0.20861	0.693599
FKBP5	0.308157	0.314971	0.609277
TUSC3	0.199247	0.872716	0.49217
MYO9B	0.001243	0.508299	0.034693
ROCK1	0.244338	0.116865	0.547425
FZD5	0.556057	0.087645	0.783439
NFATC2	0.734076	0.085386	0.882858
TNK1	0.660792	-0.29045	0.844596
TOP3A	0.086582	0.890109	0.337307
IL18R1	0.21693	0.943026	0.513767
GAB1	0.020787	1.317794	0.172048
SMAD4	0.044583	0.335796	0.24851
TCIRG1	0.511623	-0.07651	0.754376
BIRC2	0.248978	0.659499	0.552533
GPM6B	0.092612	-0.66851	0.347013
PICALM	0.002718	0.384745	0.058088
MTM1	0.233812	-0.18554	0.535403
SQSTM1	0.749036	0.023972	0.890902

MED21	0.333172	0.253024	0.620731
MTX1	0.892438	-0.00619	0.966699
NAB1	0.012976	0.9666	0.139171
TUBB3	0.017103	1.190778	0.157941
ASAH1	0.411524	-0.06917	0.683152
PRPF4B	0.157109	0.281746	0.447022
PIN1	0.177368	0.194695	0.468602
SERINC3	0.001587	2.070642	0.041122
ATR	0.797297	0.157031	0.915298
EIF4EBP1	0.316406	1.208571	0.613139
EIF4EBP2	0.612103	0.428259	0.81882
RIPK1	0.000596	0.557265	0.022651
HDAC1	0.114161	0.262908	0.385201
CAMK2B	0.713329	-0.03889	0.87141
CAMK2G	0.064284	-0.24274	0.296208
CAMK2D	0.098469	-0.24687	0.357719
DCTN2	0.426712	0.085924	0.693599
PKD2	0.000471	2.211844	0.019767
NAE1	0.103963	0.321535	0.366863
IRF5	0.502215	0.75275	0.747559
TDG	1	-0.04544	1
LAPTM5	0.172995	0.336174	0.464476
ITPK1	0.933906	0.029482	0.984157
SNW1	0.327323	0.202895	0.617719
DGKZ	0.170417	0.704255	0.461822
IQGAP2	0.632496	-0.21011	0.830965
STIM1	0.490639	0.12115	0.738656
GRAP	0.312248	-0.62596	0.610732
SEMA5A	0.768139	0.071717	0.901915
TRA2A	0.934288	-0.06941	0.984157
SNX1	0.2958	0.116471	0.598344
KRR1	0.543955	0.244972	0.777449
PEX6	0.773827	-0.07593	0.903913
DNASE1L3	0.702525	0.10622	0.866226
PWP1	0.024286	0.730093	0.188496
MTMR1	0.850885	0.044831	0.944945
MTMR2	0.005891	1.432601	0.089865
MTMR3	0.265899	0.798372	0.568807
CUL1	0.407778	0.279149	0.680612
CUL2	0.729121	0.072188	0.879656
CUL3	0.840557	0.081045	0.939212
CUL4A	0.053686	0.538128	0.271145
CUL4B	0.09193	0.313069	0.345626
TP53BP2	0.290359	0.613126	0.594164
DYRK1A	0.618268	-0.00415	0.822194
TSTA3	0.44615	-0.25592	0.706795
RAB31	3.02E-05	0.779728	0.002309
RAB32	0.151386	0.203101	0.44125
TPBG	0.000815	0.8	0.027022
FHL1	0.572777	-0.26763	0.794481
FHL3	5.81E-05	1.041088	0.004106
PMS2P11	0.881708	0.122487	0.961515
RIN1	0.019168	1.21811	0.166914
ITGA7	0.828732	0.112391	0.933615
AAMP	0.188098	0.359275	0.480067
ALKBH1	0.077382	0.714544	0.324641
MOGS	0.038035	0.256021	0.233324
ALCAM	0.142204	-0.37983	0.428816

LAMB3	0.634844	0.232606	0.833063
LAMC2	0.466057	0.221092	0.721065
RUNX3	0.005739	1.359911	0.089283
THOC5	0.600007	0.086447	0.810262
NCOA4	0.028425	1.031573	0.20366
APOF	0.437322	0.404751	0.70193
ARFRP1	0.030755	0.433657	0.211475
SHROOM2	0.860544	-0.05014	0.950165
ITGA9	0.599325	-0.23022	0.810262
SPTAN1	0.311298	-0.10956	0.609894
ENPP2	0.045989	1.010224	0.252361
GNL2	0.025994	0.892522	0.193808
AUH	0.466082	-0.25649	0.721065
PKP1	0.129517	0.825428	0.410218
DDX39B	0.056188	0.249083	0.276683
BLMH	0.009812	0.50124	0.119724
EXOSC2	0.779221	0.083218	0.906856
BMPR2	0.450332	0.111052	0.709868
PTK6	0.084914	-0.86417	0.335537
SNTB1	0.236413	-0.25364	0.537858
TUBB2A	0.003297	0.498474	0.065524
KLF5	0.912516	0.11652	0.975706
GTF2H2	0.092612	0.437235	0.347013
GTF2H3	0.07686	1.254881	0.323634
BYSL	0.913333	-0.16675	0.975706
RAPGEF1	0.030941	0.991903	0.212485
IDI1	0.887214	0.126449	0.96416
CAPS	0.665395	0.360835	0.847351
CUX1	0.171095	0.494976	0.461822
RUNX2	0.000358	2.44163	0.016336
CBFB	0.004611	0.448976	0.078316
NFYC	0.533062	0.424186	0.7709
PRKG1	0.02267	0.681714	0.180886
CEACAM7	0.144841	-0.59801	0.43345
CDK13	0.332231	0.185777	0.620025
IL16	0.474191	0.176023	0.727018
CKAP5	0.018368	0.319835	0.163766
CIRBP	0.019371	0.43916	0.167192
CAMK1	0.036226	0.61482	0.227448
COTL1	0.244338	-0.30354	0.547425
COL4A6	0.043407	1.165996	0.247142
COL9A3	0.594656	-0.11651	0.806337
COL9A2	0.052298	0.904968	0.26804
COX17	0.114157	-0.20883	0.385201
HNRNPD	0.804634	0.015207	0.918848
TOB2	0.739737	0.084106	0.886374
SCARB2	0.094502	0.329398	0.349959
NID2	0.105374	0.230242	0.369374
LRP8	0.22416	0.72244	0.522852
IL18	0.430547	-0.72357	0.695651
DPYS	0.683728	-0.06197	0.85709
DAG1	0.61848	-0.07482	0.822194
VEZF1	0.263262	-0.55172	0.565873
PDE1C	0.3604	0.374728	0.644411
DSG2	0.71923	0.291796	0.874478
TRIM29	0.889918	0.212182	0.96614
VGLL4	0.74294	0.138129	0.888303
BOP1	0.216173	0.261897	0.512741

UBE4A	0.126762	0.220019	0.403998
SEPTIN6	0.426712	0.129495	0.693599
TRIM14	0.743984	0.091416	0.888303
KEAP1	0.46508	0.113926	0.721065
URB2	0.009372	1.201893	0.117212
DHX34	0.002203	1.167713	0.05007
MORC3	0.039909	0.660505	0.23845
SAFB2	0.38573	0.196726	0.664306
EIF3A	0.595428	0.045506	0.806337
ARHGEF7	0.754042	0.179942	0.894351
EFR3A	0.001749	0.603145	0.043787
UBAP2L	0.000289	0.659714	0.013838
SCRIB	0.070198	-0.37155	0.309184
GIT2	0.000897	0.476385	0.028766
SCARF1	0.979027	0.004767	1
IKBKE	0.074021	0.921927	0.31759
MLEC	0.123514	0.243947	0.399387
TTLL12	0.311298	0.262846	0.609894
MPP2	0.679383	0.033579	0.85545
POLA2	0.065603	0.898808	0.299507
DOCK1	0.001133	0.929878	0.032731
TFDP1	0.164306	0.867494	0.454094
TFDP2	0.78809	0.04812	0.911483
WRN	0.400167	0.393902	0.67622
FHL2	2.13E-07	1.305512	4.54E-05
CRMP1	0.26988	0.679973	0.572322
DPYSL3	9.32E-08	1.004953	2.25E-05
ICT1	0.78936	-0.09038	0.911483
ZMYM3	0.378388	0.431258	0.659304
DCTN1	0.913333	0.000829	0.975706
DYNC1H1	0.157109	0.11868	0.447022
LY6D	0.99433	-0.08418	1
EBI3	0.101663	-1.2787	0.363022
EIF2B1	0.360948	0.19417	0.644411
EIF4A2	0.052873	0.221492	0.269122
TCEB3	0.247715	-0.55551	0.550914
SELPLG	0.992523	0.042222	1
MAP7	0.808226	-0.17688	0.921586
CTTN	0.167008	0.326389	0.457139
ENDOG	0.481654	0.468139	0.733586
FLOT2	0.286754	-0.12866	0.590053
RCN2	0.049723	0.467724	0.261364
TRIM25	0.003823	0.284091	0.070612
ERV3-1	0.421971	-0.37447	0.691467
PTK2B	0.78936	-0.06395	0.911483
FASTK	0.226845	0.561726	0.526348
FGL2	0.474167	-0.2906	0.727018
FLNC	0.001527	1.082296	0.040048
FKBP8	0.955289	-0.03004	0.993074
FAM50A	0.01453	0.388414	0.147184
FRG1	0.866382	0.109647	0.953042
GNA13	0.101186	0.205266	0.361901
GAMT	0.241242	0.576101	0.544139
GALE	0.123514	-0.37137	0.399387
LRRC32	0.019364	1.504829	0.167192
GAS6	0.004819	1.497433	0.08122
GCKR	0.08528	-0.38071	0.335537
GK2	0.48463	0.450909	0.735121

PDE3A	0.075935	0.889702	0.321214
GALNT3	0.208909	0.629464	0.503054
PIGH	0.925522	-0.08655	0.981917
CAPRIN1	0.001956	0.471783	0.04685
GRB14	0.74806	-0.25682	0.890902
GRB7	0.753596	-0.15507	0.894351
BECN1	0.382122	0.020303	0.661698
HES1	0.382833	0.483788	0.662478
SLBP	0.438086	0.343153	0.701933
RBM39	0.007049	0.39215	0.100623
WFDC2	0.696924	0.843189	0.863255
NEDD9	0.450001	-0.33044	0.709598
FGFBP1	0.067371	0.856714	0.302148
SPARCL1	0.910717	0.003087	0.975636
FAT1	5.76E-06	2.012722	0.000627
HABP2	0.554951	-0.10702	0.783439
KRT33B	0.81914	-0.10141	0.92687
HIC1	0.204074	0.522335	0.497963
HLTF	0.170955	0.384814	0.461822
KRT81	0.556057	0.080983	0.783439
SQLE	0.377949	0.461558	0.659304
HNF4G	0.300882	0.305125	0.602797
SLC29A2	0.992139	0.006772	1
PDIA5	0.004424	0.408579	0.076312
PRPSAP1	0.360948	-0.14047	0.644411
DHX8	0.09193	0.366361	0.345626
SEMA3A	0.366436	0.224042	0.649119
MCM6	0.013241	1.668503	0.141015
ITPR2	0.902877	-0.09231	0.971583
ITPR3	0.032856	-0.37781	0.217297
DSC3	0.565824	0.236663	0.79107
ZNF460	0.182896	0.370657	0.474175
NBR1	0.636492	0.142992	0.833511
ITIH4	0.656149	0.323753	0.84209
INPP5A	0.846775	0.131279	0.943024
ITPR1	0.613839	-0.12472	0.819264
RASA3	0.001149	1.660449	0.033016
PLS1	0.67051	-0.04447	0.850681
IRF3	0.5116	0.335539	0.754376
KCNJ11	0.181133	-0.34622	0.472575
LAGE3	0.689778	0.474758	0.859429
KIAA0100	0.364315	-0.29553	0.646996
TRIP12	0.019032	0.394276	0.166597
PUM1	0.072266	0.475397	0.313701
MDC1	0.047046	0.607636	0.255529
CLINT1	0.774166	-0.06452	0.903913
KANK1	0.113653	0.810257	0.384893
MELK	0.048504	0.732157	0.258731
KCTD2	0.954108	0.047223	0.993074
SMC1A	0.554955	0.089633	0.783439
RRP1B	0.000547	1.279013	0.02168
NCOA6	0.484375	0.322022	0.735121
GSE1	0.529416	0.331762	0.768066
DIP2A	0.456042	0.347221	0.714622
PDCD11	0.897655	-0.02993	0.969793
GINS1	0.229479	0.491444	0.529537
BMS1	0.081042	0.601835	0.328405
LPIN1	0.239679	0.391661	0.542335

USP10	0.005772	0.525741	0.089283
MESDC2	0.09193	0.240325	0.345626
GANAB	0.34052	0.094822	0.627258
RFTN1	0.840557	0.017806	0.939212
MBTPS1	0.176252	0.598067	0.468218
SSPN	0.084371	1.296515	0.335025
KCNAB1	0.106965	-0.76726	0.371691
MFSD10	0.699592	-0.00553	0.864146
PPP2R5D	0.011419	0.395017	0.128971
LBR	0.108241	0.343628	0.374055
COG2	0.392997	0.290951	0.671013
GNMT	0.186982	-0.43521	0.479162
PTPRCAP	0.530115	-0.27635	0.768664
MVP	0.613839	0.00813	0.819264
STAT4	0.423391	-0.33404	0.691988
LTBP1	4.78E-06	1.130035	0.000541
LTBP2	0.314461	0.344332	0.612305
ICAM4	0.163438	-0.24877	0.453012
CBX2	0.25797	0.439255	0.560462
GOLGB1	0.085745	0.221502	0.335537
CASP8	0.309724	0.322305	0.609864
NAA30	0.082821	1.234932	0.332815
FXYD3	0.107566	-1.2877	0.373049
KIF22	0.106695	0.869894	0.371671
MEF2D	0.158724	0.322907	0.449897
CHD4	0.023466	0.318108	0.184845
LASP1	0.04896	0.322368	0.259694
STARD3	0.298114	0.312733	0.601676
ARID5B	0.163894	0.564655	0.453304
CRYM	0.11931	-0.86843	0.393595
PTGR1	0.575023	0.176912	0.796599
DRAP1	0.030249	0.528949	0.209418
NFATC4	0.003295	1.482332	0.065524
NFIX	0.709323	0.557543	0.869179
GPNMB	0.173168	0.409312	0.464476
RAB39A	0.933998	0.106826	0.984157
ZNF638	0.181644	0.407333	0.472575
KPNB1	0.013012	0.255152	0.139171
NOLC1	0.779219	0.331923	0.906856
NUMA1	0.490639	0.121389	0.738656
PSME4	0.22065	0.680137	0.519031
CUL7	0.003732	1.103574	0.070138
SLMAP	0.082198	0.282296	0.330671
GAPVD1	0.239034	0.129856	0.541047
CLCA4	0.045488	-0.45753	0.250872
KRT72	0.625883	-0.38038	0.828579
UBXN2B	0.775018	0.140659	0.904436
NAA25	0.00511	0.808356	0.083708
FASTKD3	0.177218	0.685628	0.468602
VEPH1	0.079685	-0.31525	0.325919
NCAPH	0.220081	0.699006	0.518019
KIAA0101	0.516723	0.269251	0.758769
SPCS2	0.123514	0.194593	0.399387
EMC2	0.407778	0.15422	0.680612
WTAP	0.241676	0.227814	0.544139
PSMD6	0.422885	0.14051	0.691467
HERPUD1	0.965781	0.056664	0.99616
LAPTM4A	0.35943	-0.69584	0.64422

MAD2L1BP	0.234209	0.490946	0.535922
MORF4L2	0.328142	0.624682	0.618843
FAM175B	0.520135	-0.22672	0.760272
SEPTIN2	0.003589	0.304241	0.068752
SART3	0.131757	0.163493	0.413476
NCAPD2	0.134471	0.941096	0.417765
SUZ12	0.185921	0.384681	0.478129
EXOSC7	0.35064	0.171813	0.635657
TNIP1	0.134781	0.786786	0.417833
ACAP1	0.604602	-0.31574	0.813002
EFTUD2	0.012773	0.264037	0.137944
LARS2	0.563832	0.185516	0.789479
R3HDM1	0.807504	-0.12572	0.921159
TRAM2	0.000786	2.290194	0.026652
SNX17	0.010997	0.476696	0.127049
DAZAP2	0.310183	0.38194	0.609864
ARL6IP1	0.724167	-0.32294	0.876654
RAB3GAP1	0.016204	0.302929	0.154611
SLC39A14	0.000132	1.637828	0.007794
KARS	0.272095	0.108312	0.575039
SETDB1	0.066575	1.071912	0.30175
LRRC14	0.707192	-0.15985	0.869179
RRS1	0.014934	0.550603	0.150049
IQCB1	0.066923	0.74044	0.302058
ARHGEF6	0.009087	0.399334	0.115713
KIAA0040	0.568504	0.384925	0.791838
POLD3	0.031117	1.227482	0.212999
EIF4H	0.008248	0.436649	0.109165
ACAP2	0.064284	0.540231	0.296208
KIF14	0.599931	0.226432	0.810262
BRD3	0.00891	1.271762	0.114289
WDR43	0.764084	0.146101	0.899242
POSTN	8.62E-06	1.064864	0.00087
ACOX1	0.035078	0.425354	0.223337
OXA1L	0.144006	0.273744	0.431666
ZNF146	0.332529	0.419609	0.620367
EEA1	0.017412	0.263443	0.159467
NCF4	0.036828	0.463579	0.229759
PDIA6	0.147661	0.185913	0.436386
PAFAH1B3	0.689846	0.058927	0.859429
AGER	0.215142	-1.16049	0.511491
PLCL1	0.22538	0.751536	0.52468
PCOLCE	2.27E-08	1.917835	7.40E-06
PDK1	0.269225	0.190284	0.571371
PDK2	0.986843	0.045658	1
PDK3	0.568296	0.118045	0.791838
PEA15	0.018368	0.4162	0.163766
PGM5	0.97632	0.091874	0.998921
EBP	0.008089	0.688482	0.107782
PMVK	0.764084	-0.13339	0.899242
PRKD1	0.003104	1.588517	0.063161
PLCB4	0.43012	-0.25504	0.695651
PLEC	0.003297	0.331121	0.065524
PCM1	0.292764	0.649393	0.595788
NOMO1	0.180562	0.740895	0.472575
PON2	0.308157	0.076745	0.609277
PON3	0.085642	-0.92302	0.335537
TCEAL1	0.643739	0.293514	0.837208

PPP2R5A	0.462056	-0.16701	0.718747
PPP2R5B	0.028929	1.164148	0.205808
PPA1	0.327323	-0.16919	0.617719
PTGES3	0.609213	0.007008	0.816529
PDGFRL	1.93E-06	3.250979	0.000255
STK38	0.016799	0.441075	0.156698
PVRL1	0.080348	1.078004	0.327024
NONO	0.09982	0.227571	0.359216
PPP2R4	0.892438	0.080873	0.966699
PTPRK	0.965786	0.262038	0.99616
PWP2	0.097132	0.284286	0.354489
QPRT	0.87156	0.09473	0.956098
RABEP1	0.016499	0.36371	0.155558
RASA2	0.006253	1.225973	0.093377
RAB35	0.149515	0.213358	0.438811
RNPS1	0.289748	0.231301	0.593134
RBBP5	0.14042	0.188887	0.426568
RCN1	0.000412	0.550469	0.018066
IRF4	0.492983	0.521657	0.741084
RALBP1	0.66395	-0.23842	0.847289
KRT31	0.331588	-0.1188	0.619341
ZMYND11	0.410055	-0.18909	0.682809
LLGL1	0.010783	0.910064	0.126291
LRRC41	0.354009	0.713765	0.638094
TMED2	0.197211	0.129261	0.490309
PCBP1	0.419078	0.119843	0.688796
PCBP2	0.019715	0.222126	0.1684
TCEB1	0.729121	0.049963	0.879656
TCEB2	0.882017	0.005806	0.961515
RHEB	0.632496	0.169608	0.830965
UBE3C	0.105374	0.381851	0.369374
TOMM20	0.684533	0.499788	0.85709
ANGPT1	0.08528	-0.37833	0.335537
MTFR1	0.142952	1.070188	0.430602
DHCR24	0.651386	-0.19887	0.839222
SF3B3	0.038035	0.25582	0.233324
KIAA0020	0.665709	0.10244	0.847351
DLGAP5	0.391202	0.318794	0.669777
TLR1	0.053552	0.877149	0.271145
RSU1	0.011206	0.341155	0.128397
CNN3	6.23E-08	1.212325	1.73E-05
RPS6KA1	0.185996	-0.21122	0.478129
SAFB	0.0799	0.51558	0.325919
SF3B4	0.097132	0.38389	0.354489
SF3A2	0.317645	0.195021	0.613139
RBMS2	0.306214	0.933179	0.607661
PPP1R7	0.374984	0.140725	0.656862
SEC23A	2.92E-07	0.73322	6.00E-05
SEC23B	0.586319	0.056053	0.801141
CYTH1	0.049522	0.826983	0.261364
SF3A1	0.147661	0.236427	0.436386
SHB	0.6534	-0.44754	0.841387
SIX1	0.233926	0.633332	0.535496
SKIV2L	0.126762	0.175321	0.403998
FCN2	0.323227	0.462846	0.614276
RGN	0.103362	-1.23907	0.366609
SPA17	0.638802	0.107785	0.834211
CDSN	0.15526	0.310916	0.444958

SURF1	0.694709	0.198806	0.861478
SURF2	0.770646	0.09831	0.903323
MED22	0.393093	1.273527	0.671013
SS18	0.618129	-0.30644	0.822194
TAF5	0.58156	0.059238	0.797998
TAF13	0.756203	0.163561	0.896333
TAF7	0.001028	1.302847	0.031079
TERF2	0.680143	0.058203	0.85545
MAPRE2	0.001956	0.720243	0.04685
TGFBI	0.014008	0.421477	0.144006
TGIF1	0.832496	0.058202	0.93551
NCOA2	0.169335	0.795705	0.460317
SLC9A3R2	0.012079	-0.44066	0.133041
TRADD	0.211314	0.283797	0.506632
TRAM1	0.013502	0.545707	0.141812
TSN	0.171096	0.281323	0.461822
TARBP2	0.374429	0.50821	0.656862
SF1	0.031269	0.327814	0.212999
TRIP10	0.025131	0.416047	0.191949
TRIP11	0.057044	0.349934	0.278658
TRIP13	0.04378	1.891232	0.247494
OASL	0.412816	0.570792	0.684488
MED1	0.121792	0.570249	0.397343
TRIP4	0.03024	0.677008	0.209418
HMGN3	0.024484	-0.97706	0.189451
JMJD1C	0.134779	0.36517	0.417833
NFKBIB	0.80434	0.461326	0.918848
TRIP6	0.208914	0.359107	0.503054
RHOH	0.730466	0.14022	0.880704
PTPN14	0.000346	1.789628	0.015914
MAPRE1	0.011856	0.317335	0.13136
DLG2	0.012927	0.990599	0.139171
TSC22D1	0.201062	-1.37637	0.494343
ELAVL1	0.199513	0.204051	0.49217
LTB4R	0.467682	-0.48874	0.722799
ELF2	0.074697	1.054326	0.318513
NSDHL	0.093209	0.313166	0.347256
NAB2	0.010828	1.747462	0.126436
MYLK	0.008575	0.589331	0.111496
TAB1	0.0412	0.776239	0.240826
HERC1	0.589244	0.107032	0.804544
SLC1A5	0.028293	0.539549	0.203235
MAPK11	0.322009	0.126439	0.613139
CD226	0.825553	-0.06947	0.930793
RAB30	0.335138	0.543053	0.622407
MLF2	0.863663	0.312125	0.951892
ZKSCAN8	0.80943	0.144796	0.921586
CHI3L2	0.705459	0.151112	0.868107
TOMM34	0.286748	0.571261	0.590053
NCOA1	0.789238	0.229506	0.911483
SMAD2	0.173102	0.543531	0.464476
SMAD1	0.338275	0.618355	0.625538
MSMO1	0.253708	0.585345	0.557407
ITSN1	0.00116	0.647054	0.033154
TBCE	0.28378	0.412964	0.586874
TBCC	0.6657	-0.1289	0.847351
NPTX1	0.672064	0.162932	0.851286
UBE2V2	0.709387	-0.02039	0.869179

CST6	0.078408	1.183537	0.32578
STK11	0.079988	0.819418	0.326155
STXBP2	0.764084	-0.02193	0.899242
CCDC85B	0.473815	0.300942	0.727018
VAMP3	0.289748	-0.05368	0.593134
KCNJ8	0.250555	-0.42331	0.554113
NEDD8	0.992106	-0.52349	1
ADIRF	0.195965	-0.85781	0.490309
ADIPOQ	0.487515	0.025984	0.737456
SLC14A2	0.988991	0.181759	1
USF2	0.596298	0.604521	0.807219
ATP6AP1	0.613839	-0.06531	0.819264
VPS72	0.696683	0.534435	0.863213
RAB11B	0.280828	0.33726	0.583434
EZH2	0.029414	1.279252	0.207259
ZFHX3	0.029315	0.622854	0.207066
ZYX	0.000582	0.506432	0.022491
HSPB2	0.594656	-0.08537	0.806337
ETFDH	0.077655	-0.30828	0.324641
SEPTIN7	0.135171	0.158792	0.417833
ADRM1	0.78936	-0.07493	0.911483
CCDC6	0.123514	0.285037	0.399387
UAP1	0.27785	0.573639	0.579677
E2F4	0.035301	1.206815	0.224243
IGFBP7	0.004905	0.521918	0.082133
NTRK3	0.594656	0.118003	0.806337
PDCD2	0.571251	0.288276	0.793891
SLC15A2	0.734512	-0.17456	0.883278
INA	0.996024	-0.16121	1
LAMA4	0.051278	0.258661	0.265139
PRR4	0.331588	-0.21479	0.619341
EXT1	0.020432	1.227558	0.170514
PSMD5	0.426712	0.123786	0.693599
TNXA		0	
PKN1	0.292764	0.091693	0.595788
PKN2	0.1068	0.344559	0.371671
CSRP2	1.00E-06	1.109621	0.00015
DDB1	0.169043	0.161675	0.459676
SNAPC1	0.49063	-0.23197	0.738656
PPP2R5E	0.749036	0.005552	0.890902
MAPK14	0.002718	0.437064	0.058088
MRPL23	0.892354	0.071116	0.966699
CDC37	0.086954	0.226497	0.337854
LY6E	0.322009	0.161878	0.613139
DPYSL2	0.347246	-0.08195	0.632606
SNRNP35	0.650206	-0.27212	0.839222
SYPL1	0.689846	-0.12526	0.859429
CAMK4	0.594588	0.157381	0.806337
ACKR1	0.983606	-0.04223	1
RBBP7	0.068179	0.276621	0.303928
C3AR1	0.217791	0.643224	0.515367
MAP3K11	0.067267	0.937989	0.302148
SGCB	0.055152	1.564505	0.274516
SGCA	0.988991	0.006372	1
TAF9	0.840203	0.000337	0.939212
FXN	0.327296	0.222025	0.617719
CALCRL	0.312942	-0.65488	0.611063
ECM1	6.74E-06	1.727822	0.000698

BAK1	0.177356	0.407228	0.468602
CTF1	0.573625	-0.37099	0.795459
NTRK2	0.960709	0.085145	0.994122
OCLN	0.246948	-0.17609	0.549922
MEA1	0.371998	0.873497	0.654894
CCL14	0.280329	-0.59933	0.583434
SRSF7	0.305036	0.257646	0.606083
CPSF6	0.020421	0.29084	0.170514
TAZ	0.046145	1.041629	0.252614
SMN1	0.479996	0.864277	0.732402
DBN1	5.27E-07	0.824363	8.85E-05
MAPKAPK3	0.965767	0.00093	0.99616
PTGIS	0.009087	0.447311	0.115713
NFIL3	0.358581	0.200863	0.642907
PRSS8	0.500737	0.459671	0.746432
PDK4	0.559081	-0.07692	0.78633
NRF1	0.494603	-0.08096	0.741997
FSCN1	0.000236	0.737154	0.011882
HIF1A	0.576048	0.265432	0.796634
IFI16	0.466082	0.178194	0.721065
ZSCAN26	0.38676	0.303424	0.665564
CYP1B1	0.003086	1.505837	0.063034
CYP2A13	0.738907	0.017138	0.886253
DECR1	0.950035	-0.0032	0.990863
MAN2A1	0.007049	0.52303	0.100623
NDUFA5	0.45805	-0.06157	0.715224
KYNU	0.088177	0.609342	0.339301
UGCG	0.200321	0.771755	0.493506
CLPP	0.641914	-0.0696	0.835764
TST	0.298858	-0.23012	0.601676
UBE2S	0.771591	0.171687	0.903336
QPCT	0.057714	1.735578	0.280499
CCBL1	0.962592	0.118804	0.995441
GUK1	0.759058	-0.01961	0.896847
HAGH	0.14042	-0.23188	0.426568
LAMA3	0.128409	-0.96739	0.407846
CA9	0.048774	1.755719	0.259694
NDUFA9	0.084549	0.232024	0.335025
ME3	0.194925	0.40816	0.488911
RTN1	0.090037	0.509079	0.343044
PCK2	0.699592	-0.09037	0.864146
PTPN21	0.306985	0.253546	0.608756
PTPRO	0.361047	0.400661	0.644411
DUSP6	0.321952	0.35488	0.613139
UPP1	0.009087	1.116811	0.115713
DDR2	0.009681	1.157338	0.119174
HADH	0.068179	-0.37556	0.303928
ST3GAL2	0.083546	0.577277	0.333074
CYP51A1	0.524434	-0.32182	0.764023
UGP2	0.739057	0.04939	0.886253
AOC3	0.05791	-0.635	0.280499
DGUOK	0.858539	0.231459	0.949134
ATP6V1F	0.939534	0.016137	0.986498
LTC4S	0.955385	0.113774	0.993081
PFKFB3	0.891321	-0.14667	0.966699
PFKFB4	0.854285	-0.05537	0.946993
UGT8	0.040652	1.178295	0.240594
TXNRD1	0.434427	0.12295	0.698194

TPD52L1	0.164343	-0.89855	0.454094
IMMT	0.442223	-0.05009	0.703704
TATDN3	0.97519	-0.03076	0.998921
ARHGAP44	0.723639	-0.26456	0.876654
EXOC3L4	0.304735	0.788497	0.606083
FAM98C	0.418612	-0.55038	0.688796
CPEB4	0.071157	1.015102	0.311134
ECSCR	0.733732	-0.09998	0.882858
KNOP1	0.844318	0.10426	0.942239
DUOXA1	0.657906	-0.16673	0.842898
HNRNPUL2	0.02152	0.212153	0.175496
TMEM132A	0.093069	0.752873	0.347256
INF2	0.018368	0.400564	0.163766
HLA-C	0.268408	-1.18648	0.571056
HLA-B	0.387241	1.009235	0.666184
HLA-C	1	-0.16937	1
HLA-C	0.404471	0.780413	0.678667
HLA-DRB1	0.616148	-0.38842	0.821456
PDS5A	0.486499	-0.02823	0.73612
KIAA0753	0.839125	0.032384	0.939212
QSER1	0.218373	0.402825	0.515816
CLEC16A	0.016733	0.843845	0.156698
KIF26B	0.362628	0.059129	0.646401
KIAA1109	0.598512	0.23266	0.809425
ARHGAP31	0.065004	-0.87623	0.298136
MTHFSD	0.322596	0.572497	0.613498
FAM83E	0.716495	0.217466	0.873394
AAK1	0.003589	0.491967	0.068752
C17orf58	0.782496	-0.00574	0.909237
MPEG1	0.200985	0.769147	0.494264
KIAA1033	0.004905	0.320838	0.082133
EXOC3L2	0.951201	-0.14388	0.991986
SHROOM1	0.331766	0.698434	0.619465
SLC5A9	0.08528	-0.36082	0.335537
TCTN1	0.291769	0.239139	0.595183
ERCC6L	0.740249	0.175951	0.886428
TSR1	0.004564	0.628908	0.078202
VMAC	0.834608	0.28828	0.935999
RALGAPA2	0.618479	-0.36003	0.822194
DPY19L1	0.001562	0.699761	0.040678
TRIM71	0.068041	0.573789	0.303928
MPV17L	0.705459	0.130111	0.868107
TYSND1	0.26284	0.789239	0.565873
IAH1	0.520146	0.2292	0.760272
ALG11	0.005543	0.466645	0.087699
CCDC57	0.693787	0.218801	0.861478
MUM1	0.11247	0.444933	0.382523
QRICH1	0.071225	0.726187	0.311134
TUSC1	0.709925	-0.05024	0.869646
GOLGA7B	0.391124	-0.51896	0.669747
SMU1	0.138653	0.160604	0.424105
ATG2A	0.707192	-0.01096	0.869179
ZNF800	0.146234	1.122924	0.43517
HKDC1	0.048022	1.30961	0.257798
CWF19L2	0.14108	0.841165	0.427981
COL28A1	0.594994	0.826271	0.806337
AP5B1	0.177172	0.518151	0.468602
CTU2	0.123453	1.032639	0.399387

UVSSA	1	-0.02227	1
HLA-DRB1	0.073289	1.941886	0.31592
HLA-DRB5	0.625215	0.398488	0.828169
HLA-DRB1	0.57907	-0.15369	0.796675
HFE	0.467955	0.415607	0.722799
BTBD16	0.536524	0.072517	0.773175
ATHL1	0.093803	0.924975	0.349072
LRRFIP1	0.289748	-0.10845	0.593134
PGS1	0.162867	0.717308	0.453012
LEPRE1	1.37E-09	1.220731	1.33E-06
TRMT5	0.372956	0.431712	0.656059
EML3	0.646644	0.124071	0.837353
NDUFAF6	0.438589	-0.47886	0.701933
LRRK1	0.25441	0.411163	0.557593
TWISTNB	0.655061	0.267584	0.84209
GFOD2	0.676778	0.234944	0.854135
EDRF1	0.168756	0.330728	0.459676
MAP7D1	0.000515	0.966118	0.020674
UAP1L1	0.054507	0.806322	0.273313
SLC25A35	0.748956	0.172941	0.890902
GRAMD1B	0.49063	0.132082	0.738656
C2orf76	0.397868	0.573128	0.675323
CHD9	0.485693	0.108302	0.736106
DAK	0.133456	-0.32256	0.416228
LSM12	0.217619	0.502722	0.515069
TBC1D25	0.267643	-0.39959	0.570195
RGL3	0.671419	0.126275	0.851247
ITPRIPL2	0.471546	0.452199	0.726047
TMEM30B	0.609091	-0.49108	0.816529
PUS10	0.546635	0.314626	0.778971
ADCK5	0.822953	0.071226	0.929468
HSDL1	0.378519	0.488408	0.659304
ARL13B	0.09909	0.403848	0.358568
SLC25A52	0.393788	-0.32286	0.6712
SP6	0.672064	0.182503	0.851286
ALDH1L2	1.60E-06	1.387155	0.000219
BBS9	0.886026	-0.1287	0.96416
GON4L	0.097657	0.635667	0.356252
GNPTAB	0.350349	0.155173	0.635657
CCDC88A	0.025994	0.95773	0.193808
TMEM176B	0.216062	0.767914	0.512741
TBRG1	0.750874	0.003512	0.892598
RABL6	0.162993	0.073144	0.453012
VMA21	0.3169	-0.21863	0.613139
TUBB8	0.236413	0.239742	0.537858
FAM174B	0.724835	0.219283	0.877157
TIMM50	0.28378	0.202294	0.586874
LGALSL	0.016813	-1.24638	0.156698
PARP14	0.001493	0.704526	0.039523
PLEKHN1	0.651513	0.083174	0.839222
TIGIT	0.079293	0.349062	0.32578
FUT11	0.009338	1.368453	0.117105
SLFNL1	0.322009	0.122962	0.613139
GLYR1	0.632496	0.056814	0.830965
ZBED5	0.643047	0.15819	0.836406
CRY2	0.009676	-0.66312	0.119174
C5orf22	0.932434	0.07345	0.984157
COX19	0.708911	0.840901	0.869179

MAP9	0.378108	-0.79174	0.659304
C2CD3	0.204074	-0.65389	0.497963
RIC1	0.002077	1.123031	0.048395
GTDC1	0.165468	-0.7249	0.455554
FAM188B	0.163085	-0.41826	0.453012
VPS26B	0.236413	0.060676	0.537858
CCSMST1	0.601657	-0.49895	0.811699
LARP7	0.360948	-0.09194	0.644411
NADK2	0.014531	0.369064	0.147184
KCTD21	0.135945	0.587187	0.419757
CCDC40	0.786868	0.140718	0.911452
GXYLT1	0.304291	0.646792	0.606083
ACSF3	0.161013	-0.28665	0.453012
PREPL	0.131582	0.536137	0.413476
TBC1D10B	0.083367	0.339311	0.332962
TPRN	0.632433	-0.20687	0.830965
ANO6	0.083367	0.435935	0.332962
PLCH1	0.97632	-0.13983	0.998921
FILIP1L	6.32E-06	1.477925	0.000675
AASDH	0.244375	-0.30666	0.547425
SVEP1	0.238688	0.525891	0.541047
DNAL1	0.655968	-0.30269	0.84209
ARID4B	0.031337	1.154328	0.212999
MRPL51	0.351135	0.869216	0.636243
GRIPAP1	0.882017	0.132654	0.961515
TMEM119	0.41336	-0.26616	0.685053
BCL7A	0.733732	-0.05957	0.882858
CCDC58	0.981581	0.024509	1
FLYWCH1	0.23168	-0.67684	0.532772
AMOT	0.041014	0.882549	0.240826
ATP13A4	0.060401	-1.01964	0.287134
SYTL3	0.158047	-0.72165	0.448712
GREB1	0.651513	0.029875	0.839222
FNDC1	2.02E-06	3.090061	0.00026
TMEM259	0.061847	1.308002	0.290811
PAN2	0.016827	1.030704	0.156698
RFTN2	0.359814	0.415001	0.644411
FAM98B	0.714303	0.101039	0.87164
EPC2	0.056667	0.633886	0.278217
ARHGAP29	0.736544	0.194174	0.884985
PDCD4	0.236413	0.12406	0.537858
FNDC3B	9.75E-06	0.914541	0.000948
CRTC2	0.391566	0.655744	0.670194
AGPAT9	0.824577	-0.10748	0.930395
LRRC23	0.03481	-0.56578	0.223337
CEP55	0.073574	0.954084	0.316471
C17orf85	0.177172	0.557197	0.468602
MPPE1	0.282463	0.595989	0.58557
TP53I3	0.071226	0.522198	0.311134
TMEM35	0.065197	0.924733	0.298136
C11orf73	0.394655	1.357442	0.672349
ORMDL2	0.29269	0.250973	0.595788
ACSM3	0.108294	-1.065	0.374055
IFI44L	0.964835	0.126457	0.99616
PHLDA2	0.034841	1.518139	0.223337
SLC44A4	0.570217	-0.30204	0.793891
PDLIM3	0.000382	0.782243	0.017029
PLEKHO1	0.265857	0.826085	0.568807

PARP10	0.084549	0.500399	0.335025
HSD17B12	0.064284	0.195528	0.296208
GLE1	0.194928	0.191034	0.488911
USP39	0.061485	0.333005	0.289565
KLHL22	0.005769	1.558724	0.089283
AGK	0.61848	-0.075	0.822194
SETMAR	0.975958	0.164821	0.998921
PLA1A	0.472922	-0.26764	0.727018
LACTB2	0.997369	0.040162	1
PYCRL	0.955289	0.026671	0.993074
CCDC92	0.800635	-0.43862	0.916865
KLHL26	0.539401	0.278044	0.775135
TSSC1	0.986843	-0.03366	1
CDCA8	0.182122	0.703523	0.473381
SMUG1	0.628551	-0.2526	0.829715
SOWAHC	0.632688	0.041534	0.831021
LBH	0.147247	1.600366	0.436386
ARHGAP15	0.648815	0.380754	0.839222
FASTKD1	0.327618	0.480782	0.618172
FBLN7	0.943939	-0.04305	0.988547
ANKRD39	0.49213	0.238855	0.740198
KRTCAP3	0.407991	-0.86844	0.680763
RAB6C	0.007479	0.467036	0.10395
BOLA3	0.629138	-0.56678	0.830094
TMEM177	0.555173	0.570826	0.783439
COBLL1	0.606822	-0.34548	0.815541
HS1BP3	0.009626	0.694119	0.118893
CYBRD1	0.874215	0.091609	0.957716
WDR81	0.223612	0.240281	0.521792
ACTBL2	0.687409	-0.34196	0.858864
CCDC93	0.981581	-0.00927	1
MPV17L2	0.907445	0.086254	0.9734
ZNF385B	0.644428	-0.51156	0.837353
EAPP	0.309615	0.58236	0.609864
OCIAD2	0.524435	0.095166	0.764023
SFT2D3	0.902728	0.628846	0.971583
PAN3	0.087714	0.860849	0.339301
NAALADL2	0.174719	0.74894	0.466796
SDK2	0.983224	-0.15928	1
PLEKHG4	0.794727	0.027054	0.913708
HSP90AB4P	0.102977	1.200421	0.365649
HSP90AB2P	0.166674	0.429097	0.457139
HSP90AA4P	0.407416	0.640046	0.680612
DCAF6	0.976387	-0.08212	0.998921
RPL39P5;RPL39	0.107619	1.936649	0.373118
TMEM41B	0.099811	-0.58735	0.359216
TMEM97	0.426727	-0.90138	0.693599
ODF2	0.263505	0.501984	0.565873
TMEM128	0.975991	-0.12229	0.998921
YIF1B	0.079892	0.315204	0.325919
ALG10	0.233837	0.898987	0.535403
OXLD1	0.296597	0.825278	0.599275
C19orf54	0.967597	-0.0983	0.997177
SLC45A4	0.476091	0.248812	0.728924
FAM133B	0.08117	0.771966	0.328802
ZNF326	0.11117	0.365031	0.380668
TTMP	0.761889	0.097073	0.899044
NOM1	0.509349	0.361588	0.753225

DALRD3	0.272407	-0.8572	0.57559
ZC3H12A	0.003449	1.323776	0.067458
FLG2	0.043784	0.930924	0.247494
MEST	0.057713	1.193435	0.280499
RILPL1	0.000689	1.344826	0.024536
PDZD11	0.143873	0.018639	0.431666
CMPK2	0.963173	0.034409	0.995704
CARD16	0.170198	-1.22456	0.461644
DNAJC21	0.16689	0.327307	0.457139
ARSI	0.712043	0.164055	0.870223
VWA2	0.401292	-0.56828	0.67733
TNFAIP8L3	0.853281	0.076458	0.946657
HERC4	0.330589	0.431284	0.619341
PIGG	0.554864	0.116609	0.783439
FREM1	0.36378	-0.18972	0.646996
CT83	0.447034	0.351201	0.707261
TFDP3	0.594656	-0.07867	0.806337
PPP6R3	0.024706	0.491921	0.190022
DDX60L	0.409138	0.539239	0.682163
TMEM67	0.442398	-0.48364	0.703763
MTX3	0.856037	0.700653	0.946993
RABL3	0.162993	-0.43054	0.453012
FAM76B	0.354985	0.517116	0.639146
COQ5	0.426712	-0.13978	0.693599
SH3D19	0.719142	0.280049	0.874478
NHSL2	0.123417	0.923537	0.399387
EMC4	0.590866	0.054839	0.804875
NOMO2	0.002283	0.385714	0.051363
EARS2	0.965801	0.000115	0.99616
C3orf38	0.001082	0.711337	0.031645
METTL10	0.957511	0.013846	0.994122
TCTEX1D4	0.57907	-0.13013	0.796675
MIA3	0.340521	0.278835	0.627258
PAGE2B;PAGE2	0.992139	0.023255	1
PITRM1	0.145824	-0.25756	0.434185
NHLRC3	0.005654	1.19932	0.088608
PSMG4	0.193918	1.116101	0.488275
WDR44	0.046732	0.261201	0.254194
DDX26B	0.319571	-0.55778	0.613139
DOCK11	0.023065	0.429218	0.182986
FGD3	0.422095	0.307761	0.691467
EFHC2	0.993518	0.072392	1
PRRC2B	0.252497	0.512672	0.555643
PLAC9	0.445128	-0.16358	0.706795
AMER1	0.730466	0.071018	0.880704
TJAP1	0.334628	0.379271	0.62172
RRP12	0.76912	0.10694	0.901915
COA6	0.651389	-0.1851	0.839222
TOR1AIP1	0.759058	0.035904	0.896847
CEP78	0.583581	0.228966	0.799765
AARS2	0.929044	0.055431	0.983115
TOR2A	0.520009	0.318299	0.760272
IQSEC2	0.00023	1.845949	0.011713
TEX30	0.168941	0.947426	0.459676
PCID2	0.194928	0.476058	0.488911
EFHC1	0.335614	-0.24308	0.623082
HABP4	0.012012	1.414993	0.132693
DOPEY1	1	-0.06145	1

SLC27A3	0.415291	-0.07275	0.686028
SAMD9	0.181643	0.129766	0.472575
COLEC12	0.364352	0.681556	0.646996
SPECC1	2.13E-07	1.546339	4.54E-05
RNFT1	0.066645	0.989281	0.301803
SMEK2	0.177071	0.741077	0.468602
WDR45B	0.120275	0.913019	0.39497
WIPI1	0.205395	0.905296	0.49957
EOGT	0.002718	0.65483	0.058088
SAMD4B	0.006502	0.582661	0.096023
BRMS1L	0.575791	0.355974	0.796634
CLEC12A	0.542041	0.140068	0.775521
TBCEL	0.016793	0.677183	0.156698
DNTTIP2	0.038957	0.836405	0.236232
DCAF10	0.902044	0.092952	0.971583
RABGAP1L	0.001996	0.896523	0.047089
TTC38	0.159047	-0.08126	0.449897
FAM26F	0.190221	0.938225	0.483515
ERICH3	0.752244	-0.33016	0.893365
COX20	0.101186	0.646332	0.361901
EXOSC6	0.81486	0.018355	0.924847
LRRK2	0.003056	-1.72145	0.06265
PPM1L	0.541018	0.343494	0.775521
TMEM201	0.364426	0.295439	0.646996
C6orf136	0.484591	0.417245	0.735121
ATAT1	0.212636	-0.63515	0.508813
SNAP47	0.023267	1.349021	0.18431
HIATL1	0.144938	0.550026	0.433523
NUP188	0.011419	0.372179	0.128971
PHYHD1	0.98683	-0.21124	1
TTC39A	0.095326	-0.8116	0.351834
MANEA	0.943162	0.077555	0.988547
MUC21	0.316309	-0.5957	0.613139
HP1BP3	0.774166	-0.06292	0.903913
SAPCD1		0	
VARS2	0.929044	0.031229	0.983115
SLC25A30	0.185786	-0.79377	0.478129
ZMYM1	0.187689	0.65548	0.480067
CEP170	0.001146	1.049224	0.033016
LDLRAP1	0.172631	0.567979	0.464157
TMEM69	0.079293	0.437863	0.32578
C10orf55	0.331588	-0.23751	0.619341
ODR4	0.233812	-0.20914	0.535403
PLEKHS1	0.666764	0.348347	0.848351
DNLZ	0.583888	0.741507	0.800019
NOL9	0.030755	0.622399	0.211475
NHSL1	0.359796	0.528221	0.644411
FREM2	0.839022	0.296434	0.939212
SZT2	0.182435	0.600905	0.473679
HYI	0.117009	-0.50868	0.389929
ZNF362	0.021286	1.695242	0.174748
TPRG1L	0.378257	0.13452	0.659304
CC2D1B	0.324076	-0.0722	0.614309
FNBP1L	0.660922	-0.0174	0.844596
FAM83B	0.673559	0.22694	0.852854
C6orf132	0.467659	0.44827	0.722799
UBXN11	0.992139	-0.00159	1
RARS2	0.066695	0.588574	0.301803

THEM4	0.981491	0.539024	1
FKBP15	0.105374	0.16809	0.369374
DDX59	0.897228	0.010241	0.969793
ZC3H13	0.054622	1.001235	0.273507
C9orf114	0.141764	1.190255	0.428816
OTUD3	0.021806	0.552865	0.176756
C10orf76	0.699592	-0.16036	0.864146
PDSS1	0.201482	0.536079	0.49489
MPP7	0.0084	-1.02769	0.110127
TMEM63B	0.709387	0.258526	0.869179
GPATCH4	0.010542	1.554292	0.124785
LRIF1	0.032247	1.078167	0.215938
ABCC10	0.400391	0.341106	0.67622
IBA57	0.324076	-0.20802	0.614309
HECTD3	0.35064	0.191441	0.635657
CERCAM	9.65E-07	3.129375	0.000149
TMTC4	0.322566	0.369548	0.613498
ZFYVE27	0.394968	0.37874	0.672485
UBR4	0.779221	0.057369	0.906856
C1orf194	0.571349	0.100268	0.793891
STXBP5	0.694668	0.53248	0.861478
KIAA1217	3.80E-05	1.142675	0.002829
ARHGAP21	0.056787	0.939237	0.278658
BEND3	0.188978	0.617356	0.481719
CAMSAP1	0.181684	0.6255	0.472575
GPR110	0.015982	0.850367	0.154257
MRPL2	0.817404	0.452779	0.926863
CFAP58	0.269746	-0.24721	0.572146
DCAF12	0.100118	0.760161	0.359821
UBAP2	0.148922	0.98556	0.438811
IDNK	0.741581	-0.22663	0.887545
C9orf64	0.311298	0.161249	0.609894
FAM162B	0.251878	-0.76407	0.555643
LCE1F	0.322009	0.167766	0.613139
FAM69A	0.559237	0.540772	0.78633
KANK4	0.648707	0.128756	0.839222
GORAB	0.042193	1.24248	0.243142
ZNF618	0.011758	1.088081	0.130798
TSTD2	0.57907	-0.14652	0.796675
ACBD5	0.382127	0.143388	0.661698
FAM102B	0.131585	0.783551	0.413476
RBM26	0.347246	0.375682	0.632606
ATAD3B	0.177172	0.631616	0.468602
FAM102A	0.123186	0.746025	0.399387
PIP5KL1	0.163438	-0.19929	0.453012
WLS	0.001597	1.496579	0.041198
CPSF3L	0.550481	0.125427	0.781327
CPTP	0.833456	0.127327	0.935999
TTC22	0.82732	-0.05579	0.932311
DCAF8	0.955289	0.122127	0.993074
ZCCHC11	0.596675	0.228466	0.807535
FRY	0.399203	-0.21089	0.67622
RNASEH2B	0.855454	0.15049	0.946993
BCL2L15	0.260848	-0.83041	0.56373
N4BP2L1	0.224995	-0.36232	0.523918
ATPAF1	0.18138	0.404906	0.472575
GRTP1	0.019085	0.889025	0.166801
RC3H1	0.078369	0.655411	0.32578

OGFRL1		0.016661	1.469881	0.156336
MAGI3		0.134693	-0.8914	0.417833
SH3PXD2A		0.013305	1.476133	0.141215
RSPH4A		0.499616	0.103494	0.745391
FHL5		0.437689	-0.46931	0.701933
DDI2		0.343873	-0.22378	0.630156
THEMIS2		0.192667	0.328646	0.486094
NDUFAF5		0.88069	-0.03731	0.961515
SOGA3		0.481732	-0.18787	0.733586
NT5DC1		0.835403	-0.09139	0.935999
SIRPB1		0.677236	0.072937	0.854325
PXDC1		0.454615	0.405362	0.713678
TMCO4		0.767502	0.399663	0.901915
AHDC1		0.009272	1.176308	0.116462
MINOS1		0.33259	-0.82906	0.620376
ARFGEF3		0.028414	-1.05421	0.20366
VPS13D		0.97632	-0.10349	0.998921
LIN9		0.049077	0.829075	0.260189
ZNF658;ZNF658B		0.855035	0.338498	0.946993
CROCC		0.61848	-0.05115	0.822194
TMEM164		0.234531	-1.11207	0.536436
ASPHD1		0.300882	0.251448	0.602797
MEX3C		0.792859	0.012043	0.913708
LYRM7		0.753963	0.046183	0.894351
RASIP1		0.428621	-0.10534	0.695651
EMC10		0.613835	0.168748	0.819264
PIK3R6		0.411887	0.247603	0.683152
RIF1		0.192599	0.579622	0.486094
VPS53		0.709387	-0.05334	0.869179
MANEAL		0.64003	-0.10391	0.835224
STRIP1		0.037428	0.179276	0.231646
ABHD17B		0.036978	1.394976	0.230405
CEP350		0.05761	0.701576	0.280499
CDC42BPA		0.078771	0.534691	0.32578
RPRD2		0.029257	0.521023	0.20681
	1-Mar	0.149732	-0.83076	0.4391
ANXA8L2		0.74145	0.098129	0.887483
RNF220		0.941333	0.098404	0.987918
ANGEL2		0.146679	0.905442	0.435709
KLHDC7A		0.028644	-1.10986	0.204827
PRPF38B		0.541768	0.433361	0.775521
TTC39B		0.260456	0.71822	0.56373
RNF20		0.041861	0.593608	0.242066
HHAT		0.289626	-0.58747	0.593134
PDE4DIP		0.003987	0.774339	0.071551
CACHD1		0.387622	0.571487	0.666378
ZNF318		0.020604	1.057309	0.171558
FAM171A1		0.137489	-0.42808	0.422524
C1orf53		0.342416	-0.35073	0.629771
LRCH2		0.979131	-0.10295	1
SDHAF4		0.634822	-0.19117	0.833063
OTUD1		0.291769	0.207955	0.595183
ARHGEF16		0.196056	-0.50943	0.490309
CDKAL1		0.001925	1.198744	0.046514
ZNF691		0.306947	0.545461	0.608756
IRAK1BP1		0.080193	-1.15776	0.326714
YOD1		0.302328	0.487377	0.603839
BROX		0.208914	0.150024	0.503054

FOCAD	0.040224	0.635335	0.239722
GPR107	0.183545	0.647026	0.474829
TAF3	0.138736	0.432151	0.424242
SNX30	0.22354	-0.59744	0.521792
FAM208B	0.549467	0.409337	0.781327
FAM46C	0.01547	1.759736	0.152523
RSBN1	0.034778	1.013645	0.223337
DAB2IP	0.273538	0.406215	0.577625
C1orf168	0.651568	0.322371	0.839222
C1QL3	1	-0.10078	1
KHDRBS2	0.381798	-0.46327	0.661698
LYPLAL1	0.830256	-0.01048	0.933615
PEAR1	0.803503	-0.14031	0.918848
ECM29	0.088177	0.218279	0.339301
ZCCHC6	0.834713	-0.06185	0.935999
SLX4IP	0.683128	0.108416	0.85709
ANKRD22	0.47419	0.077633	0.727018
SHE	0.645144	-0.14468	0.837353
DENND4C	0.32085	0.207801	0.613139
NAA35	0.02891	1.35288	0.205806
MBNL2	0.069467	1.300482	0.307321
LRRC16A	0.126762	-0.36463	0.403998
ZMYM4	0.243966	0.752049	0.547425
BSPRY	0.133518	-0.9108	0.416228
FAM160B1	0.277891	0.351757	0.579677
ZDHHC20	0.666998	0.389146	0.848454
SPRYD7	0.799529	-0.12549	0.916173
PAPD7	0.141292	0.397733	0.427981
KRT79	0.298717	0.333685	0.601676
FAM219B	0.49063	0.121194	0.738656
QIL1	0.759058	0.083453	0.896847
RNF123	0.388697	-0.48486	0.667643
FBXW9	0.836576	-0.04383	0.937124
ANO1	0.162979	0.730657	0.453012
HLA-DRB1	0.992139	0.006433	1
CD276	6.46E-08	1.442741	1.75E-05
ALS2CL	0.576048	0.173906	0.796634
AFMID	0.029398	-1.4497	0.207259
PLCXD3	0.716722	-0.01345	0.873394
RNF213	0.069183	0.363649	0.306571
EGFLAM	0.281331	0.269195	0.583877
TNS2	0.882016	0.261402	0.961515
KANK2	0.317645	0.320833	0.613139
FAM21A	0.031101	0.882205	0.212999
METRNL	0.713547	0.246682	0.871581
LPCAT4	0.541985	0.815934	0.775521
FAM154B	0.238303	-0.45704	0.540624
STEAP3	0.871617	-0.26099	0.956098
FAM91A1	0.157109	0.176315	0.447022
ICE2	0.954108	-0.02983	0.993074
LARP1B	0.556747	0.272378	0.784211
CEP135	0.675185	-0.30692	0.853175
TBC1D9B	0.032856	0.346451	0.217297
MAP1S	0.2958	0.062962	0.598344
CPZ	1.97E-05	2.689657	0.001698
E4F1	0.060469	0.622092	0.287134
PPP2R2D	0.994671	0.275345	1
ARL6IP4	0.002362	1.190468	0.052824

ATG16L1	0.731594	-0.21925	0.881584
RAP1GAP2	0.792343	0.105439	0.913596
STEAP4	0.148574	-0.72515	0.438051
OLFML2A	0.481155	0.331524	0.733586
OLFML2B	0.043608	1.032213	0.247494
HGSNAT	0.013913	1.799967	0.144006
ARID2	0.008219	1.210973	0.10891
DIEXF	0.176827	0.435489	0.468602
GLT8D1	0.005486	0.941654	0.087298
SEL1L3	0.850881	0.050491	0.944945
RPGRIP1L	0.298171	0.406895	0.601676
TNS3	0.000275	0.597406	0.013262
HAUS3	0.008795	1.484014	0.11325
SLFN13	0.030839	0.949496	0.211921
SPTY2D1	0.636013	0.055825	0.833511
MBLAC2	0.38928	-0.17739	0.667643
FMN1	0.079555	-0.92291	0.325919
ANKS6	0.962445	0.038602	0.995382
PLA2G4F	0.792046	-0.38147	0.913596
LMBRD2	0.482833	0.624033	0.733977
ZFYVE26	0.135998	0.394984	0.419803
MSL1	0.025871	1.320401	0.193808
C2orf16	0.831174	-0.06758	0.934471
CRYBG3	0.412682	0.373955	0.684368
INTS3	0.000741	0.689024	0.025686
ARHGAP17	0.014008	0.368108	0.144006
SLC6A19	0.15526	0.274704	0.444958
NCBP2-AS2	0.226242	-1.22827	0.525276
CWF19L1	0.097128	0.673496	0.354489
KIAA1429	0.223612	0.427726	0.521792
SPECC1L	0.025563	0.566846	0.193268
C12orf73	0.616936	-0.47879	0.822194
C1orf95	1	-0.05284	1
HEATR6	0.027183	1.155229	0.199264
ANKRD40	0.78305	-0.18551	0.909506
GLTSCR1L	0.071488	0.746268	0.312032
WWC2	0.081553	1.265655	0.329871
SCARA3	0.058364	0.850663	0.281933
TRIM68	0.939739	0.080357	0.986525
HBM	0.971656	0.095128	0.997592
CYB5R2	0.068946	1.401973	0.306571
REP15	0.484075	-0.26303	0.735071
UHRF1BP1	0.1793	0.603903	0.471072
ZNF787	0.925827	0.040479	0.982053
ATL3	0.000815	0.357234	0.027022
RPL7L1	0.400346	0.241027	0.67622
LGALS9C;LGALS9B	0.657906	-0.20377	0.842898
NXN	0.004955	0.789753	0.082765
TTC19	0.57882	-0.44604	0.796675
MCTP2	0.547806	0.199777	0.779889
IQSEC1	0.068176	0.586691	0.303928
CDC42BPG	0.907988	-0.19635	0.9734
DMKN	0.69426	0.065642	0.861478
POLR2M	0.321952	0.479652	0.613139
VASN	0.003027	1.185005	0.062294
RLTPR	0.132629	0.70252	0.415298
SFRP4	2.08E-06	3.139875	0.000262
HIST2H2AA3;HIST2H2AC	0.161012	-0.33826	0.453012

CIAPIN1	0.646644	-0.00461	0.837353
ZFAND6	0.654698	0.332586	0.84209
FAAH2	0.003522	1.419218	0.067937
SMYD5	0.957906	0.232783	0.994122
PTRHD1	0.070198	-0.32239	0.309184
XAF1	0.101663	1.000745	0.363022
OTUD7B	0.203799	0.672277	0.497963
LAIR1	0.075781	1.37461	0.320798
RALGAPA1	0.022597	1.092279	0.180886
ABLIM2	0.078579	-1.00587	0.32578
THOC7	0.866426	-0.09266	0.953042
SIGIRR	0.056503	-0.75963	0.277829
NADSYN1	0.016199	1.04244	0.154611
ELP2	0.02152	0.459689	0.175496
LAMTOR1	0.840557	0.014946	0.939212
DHRS7B	0.131757	-0.34357	0.413476
TWF2	0.023065	0.304121	0.182986
NCAPH2	0.26189	0.678909	0.565402
GRAMD4	0.389353	0.29627	0.667643
DESI1	0.117167	0.773464	0.389929
FAM109B	0.740193	0.109324	0.886428
KIAA0930	0.360975	0.276672	0.644411
TANGO2	0.131811	-0.60821	0.413476
SNRNP48	0.614399	-0.17057	0.819914
MRM1	0.093509	1.031304	0.348258
SMEK1	0.466082	0.226883	0.721065
LYRM5	0.819598	-0.17578	0.92687
TYW3	0.546526	0.258596	0.778915
CCSAP	0.775021	-0.10382	0.904436
NAPEPLD	0.739498	0.136712	0.886295
RAB12	0.147661	0.269159	0.436386
PLEKHA7	0.07517	-0.7175	0.31955
DENND5A	0.250158	0.563886	0.553572
SDE2	0.973875	0.07075	0.998921
WBSCR17	0.568515	0.235358	0.791838
GRHL2	0.702654	-0.27904	0.866289
RCSD1	0.349127	-0.29798	0.635199
ACAD10	0.572756	0.3016	0.794481
PYHIN1	0.372503	-0.44339	0.655575
KRT80	0.072317	1.364936	0.313796
NIPBL	0.568296	0.375403	0.791838
SLC25A25	0.053456	1.227257	0.271092
PDE12	0.76912	0.05266	0.901915
B4GALNT3	0.198875	0.655581	0.49217
HEPHL1	0.725845	-0.19598	0.877996
LIN54	0.257971	0.396274	0.560462
PRR5L	0.46818	0.24257	0.722799
FSTL4	0.65015	0.270801	0.839222
CAPN13	0.969476	0.101966	0.997592
TENM4	4.32E-07	2.106692	7.61E-05
ZNF280D	0.779121	-0.04387	0.906856
OGFOD2	0.388612	0.421807	0.667643
NAA16	0.196206	0.629903	0.490309
MFSD5	0.000911	0.971099	0.028963
KIAA1161	0.054252	1.189107	0.273313
LRRC8E	0.788605	-0.07707	0.911483
SLC18B1	0.396169	0.519141	0.67382
HMBOX1	0.667193	-0.34624	0.848605

RHBDD2	0.617691	0.222078	0.822194
SLC25A24	0.12513	0.276104	0.401334
REEP3	4.98E-05	2.122125	0.003588
RETSAT	0.257957	0.041048	0.560462
ACSM5	0.029137	-0.70967	0.206759
RINT1	0.876815	0.148965	0.958859
TMEM214	0.018045	0.414303	0.162744
TCTN3	0.001159	1.092194	0.033154
UGT3A1	0.15526	0.283195	0.444958
MFSD12	0.009698	1.339508	0.119248
KIAA1211L	0.456727	-0.48235	0.715224
HIBCH	0.179497	-0.22916	0.471072
RWDD4	0.817274	-0.56183	0.926828
C3orf17	0.087816	0.861686	0.339301
PRPF40B	0.083185	0.956409	0.332962
ARMC6	0.051843	1.280186	0.266818
ESRP1	0.699591	-0.42984	0.864146
TTI2	0.130663	0.796253	0.412519
ANKRD54	0.948334	0.097788	0.990766
SLC30A6	0.004803	0.80969	0.081112
TAPT1	0.474179	-0.63142	0.727018
KANK3	0.101235	-0.95177	0.361959
JMJD6	0.047243	0.549585	0.255923
PPP1R18	0.000554	0.954674	0.021722
MZT2B	0.201482	0.751103	0.49489
PTRF	0.546147	-0.06009	0.778738
ZCCHC8	0.016499	0.600367	0.155558
CDC42EP5	0.161533	0.411888	0.453012
RPUSD3	0.678199	0.245608	0.854957
FAM180B	0.556057	0.128542	0.783439
DHX57	0.003514	0.819487	0.067901
MRPL54	0.484149	-0.53323	0.735071
ERAP2	0.200294	-1.098	0.493506
LPCAT3	0.524435	-0.32044	0.764023
CDC73	0.81486	-0.10527	0.924847
PMF1	0.055039	1.201065	0.274516
GTF2H2C	0.015301	0.488959	0.151713
FAM117B	0.299797	-0.68355	0.602797
MRPL14	0.581778	-0.53622	0.797998
SLC27A4	0.077655	0.353851	0.324641
LLGL2	0.005432	-0.89859	0.086993
CC2D1A	0.627809	0.02692	0.828932
TATDN1	0.277891	0.639224	0.579677
LETMD1	0.860982	-0.02049	0.950165
METTL2B;METTL2A	0.010055	1.631162	0.121214
MSANTD2	0.110316	0.7542	0.378798
DUS1L	0.274494	-0.62993	0.577625
C3orf33	0.723033	-0.21947	0.876654
TAF2	0.137223	0.54531	0.422409
C8orf82	0.343868	-0.37571	0.630156
MED27	0.008473	0.990397	0.110694
EDC4	0.000392	0.509109	0.017322
CEP85	0.744459	0.237485	0.888419
TMEM53	0.339343	-0.5721	0.626528
FAHD2B	0.912516	-0.10756	0.975706
PNCK	0.322009	0.130066	0.613139
PRMT9	0.220201	0.543697	0.518085
PRPF8	0.039276	0.259247	0.236232

DENND1B	0.194685	0.374434	0.488911
FBXO42	0.542247	0.196221	0.775607
SCYL2	0.177368	0.244919	0.468602
TTC27	0.008373	1.317309	0.110127
NIPAL3	0.048375	-1.31644	0.258191
SFXN4	0.387864	0.507153	0.666532
PLBD1	0.986843	-0.00329	1
CASC4	0.037428	0.38115	0.231646
FDX1L	0.206464	0.936681	0.500256
WDR73	0.353546	0.435395	0.638094
CNNM4	0.840557	-0.09078	0.939212
NFRKB	0.3791	0.320085	0.660167
GGT6	0.369092	-0.33918	0.652262
MZT2A	0.082817	1.867967	0.332815
FAHD1	0.330591	0.264964	0.619341
TNFAIP8L2	0.915947	-0.39943	0.977664
KLC3	0.784435	-0.1125	0.909583
RPL22L1	0.185268	1.108826	0.477641
LEG1	0.102462	-0.62896	0.364372
SLC39A4	0.08092	-0.90701	0.328405
C22orf39	0.08528	-0.37233	0.335537
PKN3	0.701126	0.094179	0.865518
C15orf26	0.503943	-0.28377	0.748324
ERICH5	0.133624	-0.64398	0.416228
ANKRD16	0.344752	-0.54852	0.631068
ALKBH5	0.011508	1.293195	0.129242
FAM171B	0.08528	-0.31114	0.335537
PDXDC1	0.590866	0.169042	0.804875
GALNT18	0.173297	0.901035	0.464598
TLDC1	0.228671	0.229569	0.528484
INTS5	0.010095	0.909858	0.121571
LRRC8B	0.496019	0.305998	0.743024
TMEM154	0.153189	0.454519	0.443165
GIMAP6	0.497979	0.333217	0.744803
VWA1	0.311298	-0.20606	0.609894
RSBN1L	0.195656	0.739065	0.490289
ABHD17C	0.522696	-0.42255	0.763395
SLC27A1	0.474191	-0.16721	0.727018
EMB	0.818371	0.137497	0.92687
RFWD3	0.09362	0.387971	0.348554
PGM2L1	0.082197	0.864461	0.330671
CTR9	0.308157	0.208913	0.609277
AAGAB	0.083702	1.435295	0.333456
TMEM88	0.163438	-0.30699	0.453012
PODNL1	0.119941	0.859075	0.39497
PPIP5K1	0.330021	0.312506	0.619341
TTC37	0.38573	0.193451	0.664306
ULK3	0.697991	-0.22683	0.864146
IYD	0.654248	-0.33178	0.84199
SHQ1	0.785681	-0.23075	0.910648
KCTD18	0.247289	0.718381	0.550407
DARS2	0.939534	0.081664	0.986498
LILRA6	0.101707	0.952191	0.363061
TMEM65	0.073842	0.88951	0.316943
INO80C	0.513901	0.445882	0.756193
TTC33	0.264588	0.740128	0.567281
KLHDC10	0.466537	0.362567	0.721669
MYO7B	0.529463	0.099192	0.768066

CCDC174	0.023962	0.892734	0.187023
FBXO38	0.684434	-0.23665	0.85709
NCEH1	0.016204	0.411862	0.154611
FIGNL1	0.536414	0.059884	0.773175
PAPD4	0.844559	0.030843	0.942239
TRAT1	0.293533	0.433064	0.596247
SPSB3	0.452579	0.429457	0.712397
FBXO46	1	-0.06525	1
TRIM65	0.012278	1.321428	0.134963
RHBDF2	0.041276	0.943216	0.240826
ELMSAN1	0.255167	0.434995	0.557593
BRAT1	0.059651	0.709003	0.285693
WDR59	0.68257	-0.03611	0.85709
ZC3H14	0.181644	0.400845	0.472575
CNST	0.073159	0.910772	0.31592
CCDC137	0.380889	0.481994	0.661698
OGFOD3	0.088169	0.436378	0.339301
ZNF773	0.639304	0.178561	0.834571
TXNDC11	0.651282	0.213136	0.839222
LARP1	0.002547	0.539372	0.055387
ATAD2	0.021605	1.332059	0.175931
TMED8	0.869474	-0.49594	0.955732
SLC30A9	0.280828	-0.206	0.583434
CPLX2	0.300882	0.380423	0.602797
TRAF7	0.003609	1.359564	0.069011
APOA5	0.171298	0.79737	0.462219
DEGS2	0.690553	-0.1171	0.859876
BLOC1S3	0.74404	0.161505	0.888303
BLOC1S2	0.151386	-0.16303	0.44125
RICTOR	0.155183	0.424795	0.444958
WDR74	0.134222	0.575459	0.417223
AGTRAP	0.774166	-0.12807	0.903913
FAM111B	0.550116	-0.42789	0.781327
SAMD1	0.228283	0.654284	0.528484
SMARCD3	0.203689	0.811743	0.497963
SARM1	0.000215	1.596884	0.011194
NHS	0.060233	0.708472	0.287134
METAP1D	0.339149	-0.39517	0.626409
MTHFD1L	0.028293	0.464268	0.203235
ANKRD12	0.668688	-0.22554	0.849728
ANKRD11	0.868475	0.07264	0.954823
AFTPH	0.387429	-0.37749	0.666378
FIP1L1	0.066208	0.402618	0.300455
CRTC3	0.035051	1.230975	0.223337
CRTC1	0.354342	0.762277	0.63835
SASS6	0.832496	-0.08511	0.93551
CSPG4	0.169043	0.455045	0.459676
MOXD1	0.322459	0.338734	0.613498
CYP20A1	0.466082	0.128186	0.721065
SFTA2	0.890867	-0.10187	0.966699
KDELC1	4.29E-11	3.807787	1.58E-07
TMEM205	0.794443	0.136355	0.913708
UQCC3	0.115826	-1.09141	0.388045
IL27RA	0.281331	0.3165	0.583877
C16orf54	0.796376	0.104473	0.914822
LRSAM1	0.005432	0.745559	0.086993
FAM180A	0.846287	-0.12681	0.942576
FAM198B	2.77E-05	2.45602	0.002167

PARM1	0.005845	-1.86216	0.089662
SHISA2	0.154635	0.611851	0.444958
TMCO3	0.087242	1.191885	0.338322
DHRS11	0.143106	-0.51001	0.430717
LCLAT1	0.089414	0.379991	0.341477
SBSN	0.655845	0.061411	0.84209
IGFL2	0.163438	-0.29742	0.453012
ENPP6	0.992139	-0.0798	1
PET117	0.923284	0.074975	0.980751
C6orf89	0.256839	0.718433	0.560316
CES3	0.701171	-0.18699	0.865518
HHIPL2	0.411887	0.269763	0.683152
PRSS57	0.455572	-0.42521	0.71417
OLFML1	0.630149	-0.07043	0.830965
FAM175A	0.647413	0.146186	0.83786
CWC27	0.161013	0.222007	0.453012
OLFM4	0.907566	-0.29498	0.9734
DHRS13	0.834084	-0.29021	0.935999
LAYN	0.310183	0.242633	0.609864
VSTM1	1	-0.02213	1
METTL7B	0.913061	0.124032	0.975706
DRAM2	0.838522	0.318991	0.93883
PLXDC2	0.000261	0.857378	0.012879
B3GNT9	0.000146	1.775159	0.008402
C16orf89	0.020123	-1.49696	0.169778
ZDHHC24	0.960779	-0.04086	0.994122
CXCL17	0.117742	-1.28862	0.39145
PI16	0.046776	-0.98701	0.254312
SEZ6L2	0.001994	2.072583	0.047089
MFSD7	0.045871	-0.66882	0.252361
TMEM108		0	
CLEC18B;CLEC18C;CLEC18A	0.829742	0.073775	0.933615
KIAA1324	0.702166	-0.36695	0.866168
CD300LG	0.331588	-0.11319	0.619341
BTNL9	0.014023	-1.13097	0.144006
CRELD2	0.221112	0.299579	0.519064
CCBE1	0.163438	-0.27906	0.453012
PAMR1	0.52523	0.269723	0.764479
NPNT	0.887225	-0.03697	0.96416
LRRN1	0.287388	0.451028	0.590811
LRIG3	0.913274	-0.0612	0.975706
WDR82	0.101186	0.261402	0.361901
APOOL	0.267798	-0.19133	0.570195
BAIAP2L2	0.169846	-0.42564	0.461029
TMC5	0.801929	0.169674	0.918063
LRRC31	0.328	0.277417	0.618682
ADAMTSL4	0.047361	1.225115	0.255923
FAT4	0.274588	0.444146	0.577625
DPP8	0.011362	1.223622	0.128971
WASH2P;WASH3P	0.007628	0.460895	0.10525
ATF7IP	0.002156	1.693407	0.049824
RANBP10	0.284062	0.508203	0.587347
NHLRC1	0.626197	0.132701	0.828579
CYP2R1	0.045488	-0.44714	0.250872
PACS1	0.022281	0.405175	0.179329
BCOR	0.062137	0.567293	0.291647
MUC6	0.899924	0.233065	0.971581
MPRIP	0.009264	0.449622	0.116462

RAB11FIP1	0.032856	0.53525	0.217297
CHRDL2	0.024484	-0.77673	0.189451
IGSF10	0.044667	-0.9495	0.248727
SOSTDC1	0.324967	-0.65061	0.615473
RBMS3	0.418065	0.458419	0.688796
NAPRT	0.44615	-0.31163	0.706795
DNMBP	0.727951	0.241143	0.879393
HACD2	0.563811	-0.03047	0.789479
B3GALTL	0.227354	0.641918	0.527418
GIGYF2	0.010194	0.612308	0.121876
CD109	0.000985	1.279117	0.030181
THADA	0.12482	0.662424	0.401334
HSDL2	0.000444	-0.71258	0.019015
CCBL2	0.376761	0.244518	0.658264
B3GNT6	0.576048	0.271394	0.796634
TMEM184A	0.178045	-0.79942	0.469506
CERS6	0.382795	-0.34893	0.662478
PPP1R21	0.043207	0.411913	0.246126
SCARA5	0.296011	-0.51605	0.598661
IL34	0.291769	0.313884	0.595183
CYHR1	0.034334	1.431718	0.222148
ADAMTSL5	0.469172	-0.3326	0.723926
THSD4	0.090662	0.56299	0.343901
KDM7A	0.501076	-0.27721	0.74652
DTHD1	0.339213	-0.07764	0.626409
TRIM72	0.312494	0.4662	0.610732
SYNE3	0.002161	0.914388	0.049824
LIN28B	0.474521	0.444796	0.727424
AEBP2	0.508455	0.152804	0.752448
MACC1	0.034562	1.480893	0.222216
DEF8	0.702511	-0.10761	0.866226
ZNF574	0.727828	0.183569	0.87934
GBP6	0.340337	0.695529	0.627258
FRRS1	0.398985	0.314769	0.67622
NFXL1	0.960544	-0.04884	0.994122
MBOAT1	0.014002	1.738969	0.144006
ATG14	0.0881	0.741479	0.339301
NBEAL2	0.604598	-0.06842	0.813002
FGD5	0.026869	1.090607	0.198225
GDPGP1	0.90759	0.029432	0.9734
CCDC141	0.219797	0.71505	0.517683
DPY19L3	0.649663	0.556767	0.839222
SLCO4C1	0.341849	-0.50159	0.629182
C9orf171	1	-0.02105	1
C15orf39	0.844739	0.203471	0.942239
QSOX2	0.047462	0.714652	0.255923
MMS22L	0.172222	0.358858	0.463535
SRCAP	0.232412	0.64013	0.534123
CCDC129	0.711489	-0.26387	0.870161
FAM83H	0.249724	-0.54689	0.552833
RBPMS2	0.331588	-0.12378	0.619341
RINL	0.346277	-0.31613	0.632606
FAM65A	0.015909	0.812776	0.153751
NBEAL1	0.256527	-0.37941	0.560028
C19orf35	0.472922	-0.26232	0.727018
WDFY4	0.426707	0.128399	0.693599
GK5	0.440294	-0.21354	0.70314
C1orf122	0.264379	1.208829	0.566943

N/A	0.038025	0.700358	0.233324
MFSD6	0.370566	-0.41417	0.654137
ARHGEF18	0.077647	0.669135	0.324641
TBC1D9	0.028037	0.736914	0.203111
UBR3	0.032777	0.843651	0.217297
TMPPE	0.38573	0.266997	0.664306
BARGIN	0.120332	0.268838	0.39497
ANKRD13D	0.770646	0.067547	0.903323
CDHR3	0.594656	-0.09143	0.806337
FAM92B	0.590114	0.409132	0.804875
KIAA1211	0.001636	1.711414	0.041715
KIAA0408	0.275346	-0.65913	0.578109
UBN2	0.646384	-0.0267	0.837353
CEP128	0.109354	0.942734	0.377246
PIK3AP1	0.293376	0.791021	0.596247
TMEM26	0.601315	0.24086	0.811336
ARHGAP27	0.367851	0.136579	0.650718
CCDC149	0.88648	0.148216	0.96416
C11orf57	0.442234	0.401957	0.703704
C15orf52	0.458401	0.574692	0.715672
DENND5B	0.074369	0.350352	0.317894
LHFPL2	0.0008	2.397999	0.026879
PNPLA7	0.628829	-0.1493	0.829785
LANCL3	0.161733	0.463597	0.453012
FGD6	0.040525	0.870451	0.240216
GPRIN3	0.975752	0.094841	0.998921
NUDT18	0.235662	0.81399	0.537803
TOM1L2	0.052871	-0.61864	0.269122
IL17REL	0.992139	0.0309	1
NCCRP1	0.711266	0.224243	0.870136
SYDE1	0.00023	1.85617	0.011713
PAXIP1	0.931766	0.014059	0.984157
STXBP4	0.348036	0.611602	0.633525
C1orf186	1	-0.0313	1
CYP4V2	0.295394	-0.31571	0.598344
MBOAT2	0.254598	0.565054	0.557593
TMTC3	7.41E-07	0.993436	0.000117
GTF2H5	0.439987	0.578598	0.70314
XIRP1	0.496663	0.438298	0.74366
VPS13C	0.892438	-0.05647	0.966699
ACAD11	0.067408	-1.15037	0.30217
USP34	0.075769	0.847424	0.320798
USP30	0.18384	0.719062	0.474829
RAPH1	0.7143	0.058081	0.87164
USP53	0.85486	0.040164	0.946993
USP54	0.544493	0.191246	0.778118
USP43	0.965711	0.115361	0.99616
SLC25A26	0.071983	0.958656	0.313575
MOB2	0.639209	0.263899	0.834546
MOB3C	0.430232	0.248426	0.695651
UNC13D	0.389353	0.031046	0.667643
CHSY3	0.238305	0.36016	0.540624
IKBIP	1.44E-05	0.883939	0.001292
PREX2	1	-0.02889	1
FRA10AC1	0.131062	0.601283	0.413427
UBE2R2	0.96271	0.369456	0.99547
CYS1	0.014974	1.196389	0.150049
KCTD1	0.021635	1.108176	0.176046

HIST2H3A	0.724835	0.349482	0.877157
MED13L	0.792343	0.046495	0.913596
LARP4	0.22653	1.199774	0.525726
SMIM5	0.310183	0.303171	0.609864
TMUB2	0.102454	1.081026	0.364372
MED25	0.1263	0.845414	0.403572
TUBA1A	0.000455	0.827279	0.019269
H2AFV;H2AFZ	0.819984	-0.06266	0.92687
RPS27L	0.572777	-0.55076	0.794481
CBLL1	0.090516	1.245114	0.343901
FBN3	0.0018	1.323348	0.044357
INTS8	0.08872	0.664994	0.34076
PGAP1	0.851507	-0.08295	0.945447
MTSS1L	0.923804	-0.12771	0.980751
NOL8	0.216052	0.654585	0.512741
SSH2	0.074758	0.647639	0.318513
CCDC80	3.93E-06	3.312843	0.000458
ERN2	0.234387	-0.79928	0.536219
CEP68	0.724189	0.190023	0.876654
BTN2A1	0.954782	-0.14323	0.993074
SUPT6H	0.007049	0.422447	0.100623
SND1	0.354055	0.116709	0.638094
MARK2	0.28378	0.203948	0.586874
COX15	0.286462	0.656914	0.590053
DDX46	0.09193	0.285797	0.345626
RUFY3	0.528742	0.028448	0.767576
SV2A	0.095807	0.378851	0.352331
TRIL	0.981644	-0.06204	1
TRMT10C	0.515876	0.225599	0.757825
BZW1	0.025346	0.38632	0.192941
CHST9	0.684725	0.280515	0.85709
CYB5R4	0.286383	0.5654	0.590053
MON1B	0.021105	0.679155	0.173882
LRRC8D	0.267723	0.579086	0.570195
ABHD13	0.781397	0.092788	0.90873
ASRGL1	0.631426	-0.03048	0.830965
KCTD9	0.072537	0.65511	0.314507
DHX30	0.005889	0.513212	0.089865
EIF3M	0.201834	0.161075	0.495032
MEPCE	0.266304	0.572	0.568807
CENPQ	0.161533	0.368738	0.453012
ARMCX2	0.289145	0.460158	0.593134
CDC37L1	0.319751	0.947931	0.613139
PARS2	0.269226	-0.33573	0.571371
FAM73B	0.762934	0.121261	0.899242
RSRC2	0.081478	1.239648	0.329697
ARMCX6	0.289035	0.668107	0.593134
RRAGA	0.651389	-0.09037	0.839222
CYFIP1	0.03232	0.134986	0.215938
DOK3	0.296961	0.870412	0.599817
NDUFAF7	0.171072	0.694833	0.461822
FA2H	0.187439	-0.81839	0.480067
GET4	0.923804	-0.08058	0.980751
GDPD3	0.947482	0.222412	0.990063
COPS6	0.871617	0.108796	0.956098
LPCAT2	0.175259	0.223606	0.466796
ENOSF1	0.809323	-0.18829	0.921586
MAEA	0.221087	0.599844	0.519064

EPM2AIP1	0.424795	0.209966	0.693599
DHX32	0.796942	0.13199	0.915123
TAOK1	0.118765	0.33858	0.392409
RAB11FIP2	0.924807	-0.12664	0.981628
VASH1	0.000863	1.033909	0.027972
SH3BP5L	0.032654	1.143381	0.217297
FASTKD5	0.244183	0.338443	0.547425
DPH6	0.944971	0.128732	0.988556
EEPD1	0.19622	0.692595	0.490309
KDM3B	0.157109	0.397933	0.447022
CHMP1B	0.74653	-0.27236	0.889644
RASGRP2	0.277875	-0.76928	0.579677
CHST15	0.000131	1.339641	0.007794
RRM2B	0.085741	-0.39862	0.335537
HS2ST1	0.025131	0.497599	0.191949
CHST3	0.007678	1.434777	0.105778
STRADA	0.019008	1.24624	0.166597
NIPA1	0.019965	2.008416	0.169553
MICAL3	0.121792	0.811449	0.397343
BHLHA15	0.944971	0.100162	0.988556
OTOP3	0.724261	-0.05186	0.876654
DYM	0.129607	0.96273	0.410353
SLC29A4	0.57907	-0.13724	0.796675
PHF5A	0.317645	0.111109	0.613139
AAED1	0.044207	0.869974	0.24851
APTX	0.849731	0.28623	0.944945
SLC36A1	0.078	1.090084	0.325593
ERMP1	0.426712	0.179407	0.693599
TRMT1L	0.347246	0.21675	0.632606
ZC3HAV1	0.392997	0.161705	0.671013
MRPL21	0.050493	0.32091	0.263221
PID1	0.418838	-0.51807	0.688796
EFTUD1	0.689846	0.124645	0.859429
MAMDC2	0.613722	0.038251	0.819264
FAM122B	0.351404	-0.48404	0.636424
SETX	0.31965	-0.63498	0.613139
DPY19L4	0.250228	0.769531	0.553616
TRAPPC11	0.38573	0.293148	0.664306
NEGR1	0.654513	0.550366	0.84209
KANSL1	0.065626	0.755778	0.299507
NUP54	0.112657	0.201007	0.382523
ATG9A	0.032856	0.690446	0.217297
LYSMD3	0.77488	-0.22671	0.904436
C14orf159	0.78936	-0.03921	0.911483
CCDC186	0.409636	0.545629	0.682657
ARMC9	0.000579	2.106255	0.022454
GPR155	0.473815	0.337979	0.727018
PRICKLE2	0.258196	0.278087	0.560621
C16orf62	0.004157	0.360393	0.073531
POGZ	0.038652	0.521139	0.235412
SLC46A3	0.493121	0.437924	0.741084
ZFYVE16	0.986843	0.024175	1
MON2	0.025781	0.479649	0.193808
UBE3B	0.13981	0.782822	0.426504
DENND4A	0.465979	-0.32747	0.721065
TMC7	0.329349	-0.18647	0.619341
TMC6	0.245026	0.637717	0.548564
TMC4	0.016364	-1.48316	0.155393

MYH14	0.138653	-0.16197	0.424105
PEX26	0.034484	1.301501	0.222148
NUFIP2	0.011679	1.374136	0.130055
SZRD1	0.86047	0.24578	0.950165
MAVS	0.704483	0.227038	0.867291
CYP2U1	0.333277	0.499482	0.620823
CLASP1	0.019371	0.457566	0.167192
DHX29	0.357491	0.243827	0.641472
LRP10	0.213664	0.5156	0.509228
COMMD6	0.16099	0.754316	0.453012
TRMT11	0.463508	0.485954	0.720701
HDDC2	0.185991	-0.26105	0.478129
HAUS6	0.018279	1.270116	0.163766
KDELC2	0.036828	0.398578	0.229759
LIMS2	0.713141	0.382706	0.871276
TTC21B	0.016163	1.009677	0.154611
P4HA3	0.006955	1.781168	0.100054
HEATR3	0.70439	0.061603	0.867291
C6orf120	0.325316	0.571374	0.615819
KIF21A	0.04454	0.978784	0.24851
HDGFRP2	0.2823	0.334378	0.58545
DCXR	0.136904	-0.23087	0.421544
BRAP	0.005438	1.502086	0.086993
EMSY	0.043761	1.099665	0.247494
AKNA	0.714038	-0.22196	0.87164
GOLGA7	0.177172	-1.18367	0.468602
RAI1	0.0736	0.810716	0.316471
WAPAL	0.003023	1.279107	0.062294
VMO1	0.657906	-0.16514	0.842898
R3HCC1L	0.862929	0.134903	0.951367
PODN	0.291252	0.393034	0.595183
IRF2BP2	0.835403	0.4295	0.935999
SDK1	0.711814	0.319041	0.870161
HSD17B13	0.016508	-1.10294	0.155558
CPEB2	0.157621	0.531425	0.44779
APBB1IP	0.305031	0.664053	0.606083
WDR53	0.43065	0.080499	0.695651
PPP1R32		0	
BMP8A;BMP8B	0.205086	0.731151	0.49957
SNX20	0.120228	0.506079	0.39497
SPRED2	0.013213	-0.58347	0.140906
SPRED1	0.49063	-0.22042	0.738656
CCDC91	0.863251	0.232825	0.951627
SRGAP1	0.073507	0.903443	0.316363
RBBP6	0.015817	1.383275	0.153751
ARHGAP30	0.09687	1.073008	0.354489
C5orf24	0.11545	1.358132	0.387731
SH3RF1	0.945492	0.075193	0.988914
GRASP	0.563678	-0.16992	0.789479
FGD2	0.040071	1.089107	0.239196
FRMD5	0.411887	0.19441	0.683152
TSEN54	0.716156	-0.17794	0.873229
PTAR1	0.459397	0.560697	0.716923
ARPIN	0.049288	0.247173	0.260808
TECPR1	0.577044	0.106454	0.796634
RABEPK	0.581483	0.495243	0.797998
MILR1	0.626197	0.123854	0.828579
MTERF4	0.782211	-0.1465	0.909001

RAB44	0.482107	0.161564	0.733586
ADAT2	0.319672	-0.20821	0.613139
HUWE1	0.098469	0.244592	0.357719
PLEKHH3	0.798665	-0.11796	0.916173
YTHDF3	0.292764	0.31426	0.595788
KRT77	0.822008	0.247702	0.928967
CNTRL	0.031578	1.393985	0.214075
CTU1	0.718812	0.246042	0.874478
PXK	0.003691	1.180295	0.069741
FILIP1	0.653609	0.358708	0.841555
PIGW	0.269641	0.472196	0.572141
STRA8	0.988991	0.010357	1
TAF8	0.881649	-0.24966	0.961515
VTCN1	0.178261	0.261331	0.469506
UBE2Q1	0.027235	0.538685	0.199515
KIAA0907	0.808002	-0.16586	0.921586
MRPL55	0.971059	0.183055	0.997592
ABI3BP	0.163799	-1.0051	0.453154
VPS13B	0.975703	0.10531	0.998921
TMED4	0.934288	0.136738	0.984157
MRPL10	0.317598	-0.07459	0.613139
CMC1	0.016059	-0.61718	0.154611
CENPV	0.819877	0.151796	0.92687
SLFN11	0.001995	1.189941	0.047089
ZER1	0.759508	0.206795	0.896997
C11orf96	0.469069	-0.63212	0.723869
MEGF8	0.262189	0.27708	0.565636
B3GNT8	0.81505	0.225104	0.924969
GALNT5	0.025597	1.113052	0.19339
TMEM179B	0.670438	-0.65942	0.850681
PCGF5	0.241498	0.54231	0.544139
GALNT7	0.913333	-0.07674	0.975706
S100A7A	0.322009	0.153845	0.613139
AMIGO2	0.158829	0.72663	0.449897
SCD5	0.338161	0.647523	0.62553
PHLDB2	8.87E-06	2.210532	0.000881
GPR126	0.449354	0.387859	0.709598
SDCCAG8	0.28809	0.631281	0.591924
DHDDS	0.012726	0.83149	0.137944
GALNT10	0.156972	0.839216	0.447022
GLRX5	0.825116	-0.00903	0.930395
TRAPPC6B	0.260739	0.096341	0.56373
TMEM55B	0.476218	-0.02668	0.729018
CLEC14A	0.099554	-1.1091	0.359216
ZBTB33	0.892123	0.105651	0.966699
DAAM2	0.152721	0.640707	0.443165
KIAA1328	0.167109	0.226174	0.457139
PATL1	0.086275	0.63143	0.336929
PEG10	0.338173	-0.37006	0.62553
ADAMTSL2	0.858679	0.200144	0.949134
TBC1D1	0.357329	0.388431	0.641472
DPP9	0.000835	0.753386	0.027481
TADA2B	0.815888	0.200766	0.925825
SYVN1	0.11117	0.546521	0.380668
TRPT1	0.191702	0.876081	0.485915
PRUNE	0.228666	0.585406	0.528484
MRPL52	0.095704	1.569156	0.352331
SETD3	0.019715	0.668278	0.1684

TTC7B	0.038216	1.14964	0.234176
ADCK1	0.589874	0.658372	0.804875
ACOT1	0.950035	0.034917	0.990863
RBM23	0.346648	0.464318	0.632606
ISCA2	0.277883	-0.20795	0.579677
NOP9	0.101186	0.388968	0.361901
PABPN1	0.057044	0.271565	0.278658
METTL3	0.426711	0.4553	0.693599
LDB1	0.631183	0.441283	0.830965
PBRM1	0.044589	0.669748	0.24851
YRDC	0.086582	1.22295	0.337307
PRPF39	0.252375	0.796075	0.555643
RPAIN	0.043729	1.310784	0.247494
TMEM135	0.865777	-0.12196	0.953042
RSPH3	0.994878	-0.03853	1
SAPCD2	0.120228	0.684087	0.39497
OAF	0.245751	1.141316	0.549743
SLC9B2	0.079293	0.394712	0.32578
ZNF546	0.554398	-0.23651	0.783439
MTDH	0.419078	0.153504	0.688796
TLK2	0.016595	1.177302	0.156247
TSPAN33	0.115315	0.744048	0.387731
ABCA12	0.072645	0.763792	0.314729
ZNF598	0.006253	0.992402	0.093377
AGPAT6	0.42656	0.517155	0.693599
MAGI2	0.755194	0.150909	0.895527
RTN4RL2	0.964156	0.033899	0.99616
KTN1	0.939534	0.059423	0.986498
CUZD1	0.163438	-0.33568	0.453012
SMG6	0.077758	0.8694	0.324826
NLRX1	0.520146	0.010713	0.760272
BCL9L	0.262302	0.557099	0.56577
PHLDB1	3.36E-07	1.218608	6.54E-05
USP48	0.774166	0.129374	0.903913
CADPS2	0.193928	1.068127	0.488275
DTX2	0.536428	0.353489	0.773175
ITIH5	0.113528	-0.98991	0.384589
STK32C	0.508455	-0.25906	0.752448
FERMT3	0.151386	0.247898	0.44125
FAM83A	0.797368	0.321463	0.915298
NAA40	0.530392	0.566329	0.768764
NT5DC3	0.066332	1.317966	0.300895
CASZ1	0.227857	-0.43345	0.528473
AACS	0.420398	0.328952	0.690453
LUZP1	1.24E-05	1.204266	0.001139
ALYREF	0.036828	0.308051	0.229759
GPR180	0.143106	0.544367	0.430717
FAM160B2	0.010519	1.206518	0.124644
MDP1	0.390129	0.802543	0.66856
KBTBD6	0.315731	-0.27228	0.613139
CD163	0.81486	0.142693	0.924847
SLC25A42	0.545521	0.427901	0.778738
MYPOP	0.086478	0.453831	0.337307
SERINC5	0.074776	-0.7223	0.318513
LRRTM4		0	
IQGAP3	0.000294	1.775432	0.013997
LAPTM4B	0.661071	0.137034	0.844596
ZC3H18	0.876796	0.243547	0.958859

VPS36	0.171096	0.175325	0.461822
TAX1BP1	0.809455	0.110907	0.921586
PACS2	0.610857	0.489936	0.818041
CAND1	0.006642	0.339263	0.097053
GLCCI1	0.759911	-0.20328	0.897378
TXNRD3	0.468054	0.25943	0.722799
FAM134C	0.374984	-0.01862	0.656862
HOOK3	0.000119	0.595883	0.007233
COMTD1	0.383925	-0.24946	0.66367
RTTN	0.282016	-0.76071	0.58508
SESTD1	0.013535	0.870933	0.141885
COMMD7	0.674751	0.513478	0.853175
TSPYL5	0.642073	-0.0486	0.835764
TMEM200A	0.390858	-0.65933	0.669566
P2RY8	0.088468	0.661113	0.339985
LRP11	0.945312	0.171569	0.98882
SGMS1	0.059684	1.014559	0.285693
TPRA1	0.411887	0.24489	0.683152
AMZ2	0.973875	0.034093	0.998921
THOC6	0.12841	0.365176	0.407846
METTL16	0.5677	0.573527	0.791838
PARG	0.311598	0.508636	0.610372
ANKRD46	0.006353	1.620736	0.094329
PPFIBP1	1.61E-08	1.163936	6.25E-06
BPHL	0.709387	-0.17218	0.869179
LONP2	0.312497	0.189854	0.610732
SLC26A11	0.398622	0.511997	0.676088
ZC3HC1	0.155183	0.212015	0.444958
OSTM1	0.052539	1.350575	0.268973
SBF2	0.093946	0.769533	0.349414
RASSF3	0.99433	-0.00043	1
CHD1L	0.135673	1.031652	0.419268
PAQR7	0.161533	0.412403	0.453012
FCHSD1	0.004126	1.459432	0.07321
NR2C2AP	0.399621	1.146396	0.67622
CCDC25	0.289457	0.687084	0.593134
PROSER2	0.681268	0.207028	0.856391
TTC30A	0.311973	0.472003	0.610732
LDHD	0.043865	-1.27679	0.247494
SKAP1	0.735989	-0.24884	0.884862
TMEM173	0.72417	-0.01588	0.876654
COA5	0.738974	0.178719	0.886253
RPS19BP1	0.884512	0.014263	0.963599
CDR2L	0.08214	1.044748	0.330671
RALGAPB	0.739057	-0.10141	0.886253
RALGPS2	0.589376	0.221794	0.804624
LSR	0.511622	-0.4511	0.754376
CHSY1	2.77E-05	2.063734	0.002167
ERICH1	0.40774	0.416666	0.680612
CARM1	0.017103	0.452019	0.157941
NUDT13	0.07862	1.103605	0.32578
NIT1	0.61848	-0.10965	0.822194
COMMD2	0.590845	-0.42876	0.804875
CIR1	0.071808	0.538913	0.313061
HEATR5A	0.289743	0.215702	0.593134
MICU3	0.862929	0.213472	0.951367
HOGA1	0.163438	-0.19783	0.453012
NCAPG2	0.611382	0.164829	0.818417

PPP1R3B	0.319672	-0.30801	0.613139
C19orf68	0.540202	0.507522	0.775521
FBXO11	0.10343	0.56594	0.366733
SFR1	0.993518	0.011764	1
VSIG1	0.315978	0.603882	0.613139
ANKLE2	7.60E-05	1.756407	0.005016
PROSER1	0.126827	0.523164	0.404091
MEX3D	0.021445	2.097605	0.175496
DGKH	0.072865	0.382056	0.315444
DDX42	0.004071	0.389036	0.072359
GKN2	0.22648	-0.73151	0.525719
TICAM2	0.459322	0.427684	0.716923
CEP57	0.683396	0.235166	0.85709
RNF130	0.00067	1.576327	0.024319
TMEM219	0.312248	-0.63354	0.610732
NME9	0.563678	-0.1695	0.789479
FRAS1	0.736094	0.26793	0.884893
SPATS2	0.000135	2.038981	0.007901
VRK2	0.021765	0.90763	0.176756
GPR97	0.783792	-0.16985	0.909506
CACUL1	0.362527	0.507261	0.646325
XYLT1	0.006905	1.118619	0.099675
NDUFA11	0.145824	0.058208	0.434185
KRT73	0.082403	1.349186	0.331377
DNAAF5	0.206534	0.553864	0.500256
PTRH1	0.082151	-0.78856	0.330671
STX12	0.194929	-0.14842	0.488911
KIF18B	0.151349	0.34868	0.44125
ECHDC2	0.005988	-2.2258	0.090802
ERO1LB	0.125174	0.570912	0.40136
PDSS2	0.617939	0.416789	0.822194
THNSL2	0.380889	0.354653	0.661698
TDRP	0.464421	-0.57027	0.721065
HOMER1	0.781457	-0.14135	0.90873
DOLPP1	0.288469	0.913718	0.592593
GATAD2A	0.155183	0.432644	0.444958
CPNE8	0.053387	1.104644	0.270869
GPSM1	0.119568	0.7959	0.394212
RAB11FIP4	0.114023	1.112022	0.385201
RAB43	0.520146	0.215484	0.760272
C2CD5	0.809743	-0.16774	0.921586
MIB1	5.97E-05	1.293798	0.004192
AMICA1	0.974393	-0.01223	0.998921
RASAL3	0.709372	-0.37748	0.869179
SGK223	0.004235	1.114094	0.074174
HPS6	0.016217	1.151881	0.154611
HRNR	0.096702	0.682359	0.354489
KLB	0.024142	-1.22445	0.188163
DCP2	0.067342	0.754252	0.302148
TMC8	1	-0.05151	1
IRF2BP1	0.145692	0.619171	0.434185
CAMK1D	0.677728	0.060264	0.854568
ABCA9	0.335614	-0.17114	0.623082
RHPN2	0.362148	0.545123	0.645858
ERC1	3.59E-05	0.792302	0.00269
RNF135	0.019732	1.360943	0.1684
HIST2H2AB	0.876815	-0.29356	0.958859
CBWD2	0.015262	1.254544	0.15168

MINA	0.610857	-0.30592	0.818041
RBM45	0.850819	-0.26582	0.944945
ZDHHC13	0.121792	1.113456	0.397343
ZDHHC17	0.218025	0.642228	0.515589
CRLF3	0.12112	0.400652	0.396836
PLXDC1	0.015995	1.26675	0.154257
CILP2	4.73E-08	3.79704	1.38E-05
CLEC10A	0.169057	-1.17179	0.459676
SIAH1	0.657906	-0.14513	0.842898
TRAPPC5	0.188199	-0.12143	0.480067
ARMC8	0.078771	0.408864	0.32578
EPHX4	0.093078	0.783499	0.347256
SPATA2L	0.886026	0.054531	0.96416
RELL1	0.565049	0.530997	0.790883
TMEM126B	0.649663	0.342465	0.839222
APOBEC3F	0.694709	0.047024	0.861478
AEBP1	2.14E-06	1.060966	0.000267
EGFL6	0.366436	0.324536	0.649119
PHYKPL	0.185008	-0.7427	0.477255
LURAP1L	0.629505	0.258007	0.830381
TBC1D10C	0.501359	-0.65799	0.746786
PLD3	0.343873	0.252494	0.630156
LACC1	0.03691	1.139499	0.230114
CCDC71	0.799868	0.113096	0.916176
HID1	0.660905	0.54915	0.844596
ANKMY2	0.36616	-0.08515	0.649045
ERI1	0.066692	1.271036	0.301803
LYSMD2	0.482512	0.685051	0.73359
DENND1C	0.025862	1.063448	0.193808
PRR15	0.392686	0.604603	0.671013
RASGRP3	0.94171	0.16714	0.988127
VRK3	0.676581	0.322705	0.854081
SLC9A9	0.692357	0.294908	0.861478
LIX1L	0.067267	0.999769	0.302148
NUDCD3	0.146647	0.702199	0.435709
PLEKHH2	0.311118	0.216436	0.609894
AHNAK2	0.003368	1.399035	0.066236
FMNL3	0.000741	1.019855	0.025686
SAMD9L	0.595424	-0.0227	0.806337
FOXP4	0.032589	1.323876	0.217083
MMAA	0.641914	-0.09754	0.835764
MAP4K3	0.374999	0.400324	0.656862
NOSTRIN	0.897609	-0.10322	0.969793
SLC41A1	0.581659	-0.39832	0.797998
NAV2	0.160242	0.530446	0.452801
LEPREL1	0.375011	0.590123	0.656862
LEPREL2	3.77E-11	2.817939	1.58E-07
CCDC50	0.124233	0.636757	0.400311
MUSTN1	0.064416	-1.67858	0.296449
SBSPON	0.266046	-0.70811	0.568807
FUNDC1	0.493028	0.496587	0.741084
ZDHHC21	0.421385	0.628243	0.691255
MCAT	0.804632	-0.40234	0.918848
GLYCTK	0.651513	0.120931	0.839222
MISP	0.576893	0.367114	0.796634
KSR1	0.081967	0.610952	0.330671
HERC6	0.644739	-0.42113	0.837353
SPNS2	0.40269	-0.25638	0.678038

C1orf210	0.706892	-0.31306	0.869179
VSTM4	0.246809	0.462594	0.549922
CEP97	0.182954	0.642388	0.474175
CARKD	0.32085	-0.18624	0.613139
FAM219A	0.083546	0.595781	0.333074
GLB1L2	0.724261	0.077517	0.876654
ARHGEF19	0.006855	0.836559	0.099518
WDR75	0.208914	0.352988	0.503054
MFN1	0.00049	0.764425	0.020212
SLC44A2	0.623133	-0.43721	0.825978
ITPRIP	0.213374	0.654768	0.509228
WDFY1	0.055344	0.281084	0.274516
TEX2	0.337949	0.287014	0.625324
CCDC117	0.72732	0.139999	0.878927
FAM114A1	0.008743	0.482248	0.112752
DCUN1D3	0.854217	0.010194	0.946993
PLEKHM2	0.003214	1.223382	0.065092
FOXRED2	0.026997	-1.02234	0.198793
DENND6A	0.181261	0.666241	0.472575
MGA	0.371886	0.205778	0.654801
GCC2	0.208914	0.456685	0.503054
SFTPA2	0.035078	-1.27222	0.223337
SFTPA1	1	-0.05852	1
HSCB	0.894964	-0.24066	0.968678
CCDC28A	0.218407	-0.65747	0.515816
ZC3H7A	0.016638	0.919954	0.156319
TRIM59	0.000516	2.244831	0.020674
PHF6	0.939533	0.055017	0.986498
ZBTB8OS	0.240421	1.512368	0.543631
SCN4B	0.001084	-1.47806	0.031645
CUL9	0.035446	1.105423	0.224904
LRRC8A	0.314435	0.503728	0.612305
LMTK2	0.965759	-0.33358	0.99616
SULF2	0.047538	0.812612	0.256066
SULF1	7.83E-06	3.018488	0.000797
LAX1	0.513544	0.506619	0.75602
CNTN4	0.589979	-0.34594	0.804875
UBR1	0.112657	0.439643	0.382523
UBR2	0.011631	0.725692	0.129699
ARHGAP12	0.502215	-0.44148	0.747559
ADHFE1	0.012466	-1.3914	0.136352
SGPP2	0.116516	-1.20376	0.389242
UNC45B	0.46786	-0.22006	0.722799
CHERP	0.055344	0.283335	0.274516
SCUBE1	0.556057	0.128712	0.783439
CDAN1	0.372948	0.535069	0.656059
ANKHD1	0.438315	0.129906	0.701933
BBS7	0.303188	0.430253	0.605162
SUGP1	0.044673	0.906227	0.248727
SUGP2	0.007779	0.775689	0.105988
WWC1	0.034998	-0.70732	0.223337
UEVLD	0.352332	0.370019	0.637424
CD302	0.133434	-0.21705	0.416228
CCAR1	0.550543	0.147888	0.781327
HOMEZ	0.756615	-0.04246	0.896333
DHX40	0.850492	-0.21953	0.944945
MCEMP1	0.689485	0.290452	0.859429
SCUBE3	0.00391	1.98142	0.071121

SKA3	0.464202	0.147032	0.721065
DNAJC10	0.204174	0.209253	0.497963
NELFCD	0.992106	0.23629	1
RHOT2	0.550543	0.081482	0.781327
RHOT1	0.434427	0.027993	0.698194
SIRT2	0.866426	0.092141	0.953042
PHC2	0.213663	0.789858	0.509228
GALNT12	0.334489	-0.76533	0.621619
FAM20C	0.872865	-0.00867	0.956872
MSRB3	0.003826	1.702313	0.070612
BAP18	0.190424	0.152427	0.483515
MRPL41	0.255193	0.354468	0.557593
NRM	0.303482	0.297199	0.605491
C9orf40	0.333111	-0.53013	0.620722
GPALPP1	0.638904	0.470304	0.834246
KLHL7	0.033871	1.335097	0.221141
PARP9	0.012079	0.540267	0.133041
PALM2	0.569813	0.717434	0.793446
FAM126B	0.525055	-0.28876	0.764479
RBM12B	0.43056	0.321698	0.695651
SLC35F2	0.343007	0.629774	0.630156
KLHDC8B	0.119988	-0.53033	0.39497
RPAP2	0.197201	0.591792	0.490309
PNPLA6	0.360948	0.136298	0.644411
SMC5	0.913281	0.196012	0.975706
DDX60	0.866426	-0.1179	0.953042
CMIP	0.863982	-0.03551	0.95196
PPAPDC2	0.050264	1.344863	0.263221
IFT20	0.971038	0.530728	0.997592
MICALL2	0.003987	0.555968	0.071551
SLC15A3	0.090029	0.269539	0.343044
DHX37	0.047291	0.841949	0.255923
C4orf19	0.331588	-0.12436	0.619341
AMN1	0.745146	-0.09173	0.888646
KBTBD2	0.019469	1.171431	0.16752
YAF2	0.64451	0.442957	0.837353
AMOTL1	0.056157	1.021248	0.276683
RAVER1	0.038652	0.486373	0.235412
FTSJ3	0.018368	0.373921	0.163766
SLX4	0.762934	-0.02948	0.899242
TMEM192	0.320845	0.329899	0.613139
MB21D2	0.182462	0.692483	0.473679
SRRM1	0.442223	0.163222	0.703704
SMAP1	0.350614	0.694963	0.635657
DIS3L2	0.76398	0.62443	0.899242
SUPV3L1	0.759058	0.020389	0.896847
GSPT2	0.955148	0.048793	0.993074
TCEB3B	0.678393	-0.2052	0.855103
ZZZ3	0.089856	0.771114	0.342706
EXOC8	0.840557	0.031632	0.939212
SYTL1	0.192599	-0.3016	0.486094
REM2	0.756763	-0.2939	0.896333
C1orf174	0.158993	0.570892	0.449897
TRIM22	0.116444	0.406849	0.389223
ZDHHC23	0.974393	0.043555	0.998921
THNSL1	0.143944	-0.52778	0.431666
SMYD4	0.160642	0.453482	0.453012
GRAMD1C	0.892148	-0.13693	0.966699

PM20D2	0.831829	-0.11781	0.935017
KIAA2013	0.007932	0.588392	0.107292
OSCAR	0.907098	-0.12238	0.9734
CMTR2	0.888901	0.11765	0.965603
CCDC170	0.540465	-0.34723	0.775521
KATNAL2	0.434422	0.170992	0.698194
HACE1	0.011869	-1.71165	0.131376
MICU2	0.266378	0.257207	0.568807
RNF168	0.007803	1.064749	0.106183
ANKRD13A	0.071226	0.427407	0.311134
ZBED8	0.605305	-0.08106	0.813848
PHACTR4	0.864926	-0.18682	0.952905
RASEF	0.592958	-0.19315	0.806337
CHPF	0.076507	0.782931	0.322705
TRMT2A	0.179234	0.342334	0.471072
RPUSD2	0.592074	-0.31439	0.806224
ELMOD2	0.249724	0.158748	0.552833
ALDH16A1	0.613839	-0.05368	0.819264
KIAA0319L	0.832824	0.3525	0.935756
RTKN2	0.967597	-0.00281	0.997177
DCP1B	0.175535	0.814555	0.466986
SLC22A15	0.988991	0.023003	1
SCYL3	0.30707	0.484546	0.608816
GPR116	0.205247	-0.77536	0.49957
XRN1	0.003823	0.429234	0.070612
UNC5B	8.04E-05	2.078046	0.005274
MAML2	0.508688	0.061278	0.752448
PELP1	0.013502	0.486328	0.141812
ZDHHC14	0.45638	0.260554	0.714931
ABI1	0.317645	0.071005	0.613139
GPR64	0.559766	0.405271	0.786765
WDFY3	0.830201	-0.05581	0.933615
SELH	0.490639	-0.38144	0.738656
CACNA2D3	0.651513	0.098968	0.839222
ASPM	0.205178	0.401016	0.49957
WDR17	0.252927	-0.61067	0.55648
CMTM8	0.457206	0.624914	0.715224
RDH10	0.236413	-0.52446	0.537858
TNS4	0.292995	0.363553	0.595821
ABCA7	0.609946	-0.37622	0.817215
CENPL	0.853023	0.018371	0.946466
FAM185A	0.508603	-0.4638	0.752448
C1orf87	0.173804	-0.60282	0.46573
VKORC1L1	0.724167	-0.23226	0.876654
RBFA	0.686281	-0.24929	0.857929
LGI2	0.127902	0.492148	0.407164
GCNT2	0.913093	0.081008	0.975706
FUK	0.474191	-0.21078	0.727018
CLYBL	0.604602	-0.28691	0.813002
SPG20	0.33719	0.313638	0.624371
ZNF444	0.57907	-0.12701	0.796675
SPICE1	0.233429	0.584129	0.535403
TTC5	0.02811	1.049903	0.203207
PUSL1	0.803024	-0.21207	0.918848
VSIG10	0.960709	0.139431	0.994122
MIER1	0.186896	0.295399	0.479162
CYP4X1	0.214398	-1.08771	0.510276
RPTOR	0.179497	0.152504	0.471072

CADM3	0.256843	-0.31373	0.560316
FAM177A1	0.850885	0.219183	0.944945
CNPY4	0.005543	0.699272	0.087699
LGI4	0.693819	0.048667	0.861478
CNTROB	0.297805	0.508295	0.601315
ORMDL3	0.840548	0.103307	0.939212
ABCA6	0.937537	-0.06928	0.986498
ADSSL1	0.426	0.739184	0.693599
LGI3	1	-0.03397	1
LILRA2	0.09909	-0.43614	0.358568
CCAR2	0.004071	0.368078	0.072359
NDUFAF2	0.779221	0.322078	0.906856
SIX5	0.65015	0.11694	0.839222
C4orf33	0.041971	-1.47032	0.242324
VPS52	0.871617	0.048839	0.956098
NUP93	0.016499	0.288286	0.155558
STK11IP	0.478274	0.189976	0.730754
ZNF687	0.380632	0.382926	0.661698
CMTR1	0.218632	0.38255	0.515816
LRRC47	0.861239	-0.03453	0.950165
DOCK4	0.45805	-0.07318	0.715224
THEMIS	0.108283	0.694312	0.374055
KRT78	0.229765	0.723942	0.529796
AIM1L	0.740193	0.083436	0.886428
CA13	0.220102	-0.66125	0.518019
SLC39A11	0.199513	0.418983	0.49217
CFAP52	0.993518	-0.01458	1
ARHGEF28	0.899734	0.016746	0.971471
INTS1	0.017103	0.688021	0.157941
ARHGAP24	0.725603	0.426473	0.877894
PROM2	0.392858	0.490509	0.671013
ANKRD35	0.414362	0.302057	0.685923
GAPT	0.756615	-0.02021	0.896333
VWDE	0.567751	-0.21266	0.791838
ARMC10	0.794443	-0.00159	0.913708
ZCCHC24	0.005831	1.452317	0.089662
GHDC	0.14042	-0.32299	0.426568
PYROXD2	0.361303	-0.53671	0.644764
SYS1	0.539833	0.020145	0.775521
ABHD12	0.266378	0.239063	0.568807
UBE2J2	0.273877	0.685546	0.577625
CLASRP	0.164137	0.846024	0.453749
LTBP4	0.322459	-0.20792	0.613498
TMEM256	0.249013	1.841757	0.552533
PQLC1	0.055609	1.472719	0.275585
PIAS4	0.003308	1.121926	0.065524
APITD1	0.6137	0.196607	0.819264
CCDC23	0.109838	1.089648	0.377625
AGGF1	0.00396	1.51971	0.071415
MUC20	0.503542	0.238061	0.747828
GPD1L	0.038652	-0.46407	0.235412
SLC35F6	0.694581	-0.44925	0.861478
SLC43A2	0.078605	1.32122	0.32578
KDM8	0.246813	0.50818	0.549922
LRRC25	0.44221	0.196381	0.703704
ARHGAP18	0.062406	0.211332	0.292127
TMTC2	0.771215	0.101761	0.903336
VWA5B2	1	-0.08208	1

ASCC3	0.019032	0.416745	0.166597
CLIP4	0.806555	-0.14079	0.920528
EHBP1L1	0.027821	0.366307	0.202072
PLCD3	0.001758	1.59092	0.043911
MTURN	0.593545	-0.37029	0.806337
MICALL1	0.362307	0.450803	0.646037
TMEM130	0.683728	-0.09743	0.85709
BBS5	0.587009	0.213157	0.801986
PPM1K	0.070837	-0.9735	0.311134
CADM2	0.319672	0.257701	0.613139
VPS8	0.071226	0.441245	0.311134
MPP5	0.734069	0.476318	0.882858
STAG2	0.511623	0.085213	0.754376
SYNPO	0.181644	0.197057	0.472575
FNBP4	0.248661	0.163944	0.552243
FBXW8	0.009374	1.375207	0.117212
SDR16C5	0.910551	-0.3239	0.975553
GTPBP8	0.488515	0.437948	0.738428
ZCCHC7	0.003912	1.447223	0.071121
LILRB2	0.104956	1.071259	0.369266
GALNT16	0.196915	0.365872	0.490309
CPXM2	1.06E-05	2.978799	0.001006
GUF1	0.463508	0.30076	0.720701
LNX2	0.054852	0.301501	0.274162
D2HGDH	0.84156	0.218946	0.940143
MFSD4	0.184889	-0.9931	0.477255
SFRP1	0.756763	-0.10178	0.896333
FSTL5	0.141545	0.634066	0.428577
RYBP	0.040119	1.015885	0.239354
PNKD	0.33388	-0.11138	0.620902
GALNT4	0.960531	0.166683	0.994122
FAM109A	0.299972	0.52287	0.602797
NIN	0.712787	0.136717	0.871036
MINK1	0.257225	0.513148	0.560462
BPIFB2	0.775021	-0.18813	0.904436
TOMM5	0.660922	-0.01358	0.844596
TMEM55A	0.80715	-0.39751	0.920997
SLC44A3	0.789744	-0.21837	0.911737
KLHL36	0.64399	-0.23683	0.837353
HDDC3	0.059668	-0.59866	0.285693
ZADH2	0.376546	-0.2929	0.658199
CHCHD4	0.417181	0.227314	0.68792
CCDC82	0.622736	0.083378	0.825978
PAQR4	0.781907	-0.07147	0.908838
MARVELD2	0.129242	0.852463	0.409783
CPA6	0.818427	-0.00422	0.92687
CBR4	0.018697	-1.12236	0.165633
MMGT1	0.586283	0.499564	0.801141
AFAP1L2	0.008603	1.747847	0.111593
CRACR2B	0.113752	-0.90728	0.385112
RAB42	0.08621	1.226373	0.336911
TMEM199	0.923687	-0.34407	0.980751
FBXL6	0.369552	0.349812	0.652868
FIBCD1	0.677236	0.020382	0.854325
OGFOD1	0.126025	0.990623	0.403148
ZNF276	0.008016	0.702342	0.107782
AFAP1	0.000421	1.390255	0.018305
MREG	0.737066	-0.24799	0.885485

ZCCHC9	0.52506	0.161903	0.764479
DCLK2	0.786868	0.019494	0.911452
OXR1	0.402184	-0.46442	0.678038
TTC39C	0.159843	0.570296	0.452018
MPND	0.363607	0.346814	0.646996
ZGPAT	0.039187	1.202566	0.236232
CERS5	0.138218	0.810594	0.42406
FAM26E	7.62E-06	1.199693	0.000783
SRBD1	0.897652	-0.0574	0.969793
TAB3	0.902287	-0.00738	0.971583
WDTC1	0.387599	-0.37483	0.666378
NKAP	0.253699	1.109941	0.557407
SMIM20	0.572718	-0.46955	0.794481
TMEM57	0.850164	0.443185	0.944945
FAM89B	0.291769	0.304155	0.595183
SH2D3C	0.337209	-0.30819	0.624371
ARRDC1	0.957272	-0.21772	0.994122
KCNRG	0.078849	0.792778	0.32578
DHRSX	0.375513	0.873208	0.657384
C12orf45	0.928788	0.114433	0.983115
FAM63A	0.307361	1.011173	0.609175
CISD2	0.149515	0.157413	0.438811
RPP25L	0.535075	0.450141	0.772522
ATPAF2	0.769119	-0.21789	0.901915
TTC9C	0.378388	0.585719	0.659304
JAGN1	0.417074	0.044587	0.687847
MRPL50	0.845718	0.053968	0.942239
ZC3H8	0.484075	0.163826	0.735071
CAMKK1	0.992139	-0.09426	1
NGEF	0.011481	0.780389	0.129242
FAM101B	0.87803	0.082459	0.959905
EIF4E3	0.800044	0.545808	0.916283
MSL3	0.70842	0.296439	0.869179
PARP16	0.783712	0.282187	0.909506
AADAT	0.49213	-0.28044	0.740198
DPP10	0.430232	0.200561	0.695651
FAM160A2	0.112485	0.669705	0.382523
LZTR1	0.502425	0.275283	0.747596
COMMD1	0.089411	0.662169	0.341477
ZBTB2	0.770451	-0.22002	0.903284
CPSF7	0.289748	0.265245	0.593134
SLC5A8	0.49063	-0.24644	0.738656
SLC15A4	0.855973	-0.23671	0.946993
MYCT1	0.349744	-0.43649	0.635657
LILRA3	0.310183	-0.40023	0.609864
CSGALNACT2	0.034133	1.21715	0.221721
ADAMTSL1	8.42E-05	2.346989	0.005462
ARFGAP2	0.347246	0.294148	0.632606
N/A	0.322009	0.148845	0.613139
KRTCAP2	0.000378	3.020796	0.01699
OTUD6B	0.023559	1.246095	0.185053
FITM2	0.906087	0.021834	0.9734
C1orf52	0.030204	0.733727	0.209418
NATD1	0.666516	-0.28524	0.848231
CD177	0.399594	0.631601	0.67622
METTL25	0.496019	0.263965	0.743024
METTL13	0.003904	0.961637	0.071121
ARL6IP6	0.572756	0.010532	0.794481

ARFGAP1	0.260744	0.203412	0.56373
SIRT6	0.005999	1.417556	0.090802
USHBP1	0.440215	-0.13587	0.70314
PCDH20	0.208847	0.627737	0.503054
LRRC17	0.0006	1.92505	0.022717
ZNF655	0.019784	0.908565	0.1684
CDKN2A	0.626197	0.312189	0.828579
NPW	0.322009	0.197976	0.613139
PQLC3	0.743478	-0.38718	0.888303
EMC1	0.12841	0.239833	0.407846
PAF1	0.015629	0.643216	0.152856
CCNYL1	0.371725	0.35902	0.65462
BEND7	0.251789	-0.5403	0.555643
UBR7	0.106261	1.162678	0.370959
MB21D1	0.631426	0.611331	0.830965
ANKRD44	0.272095	-0.20914	0.575039
DDX51	0.08816	0.761662	0.339301
PDCD7	0.835151	0.075557	0.935999
CEP112	0.791168	-0.00749	0.913
SAMD12	0.966966	0.021794	0.996989
C4orf32	0.039916	-1.8706	0.23845
KIAA1958	0.384669	0.243103	0.66413
ARL10	0.153189	0.463477	0.443165
PTGR2	0.09047	-0.1484	0.343901
COX18	0.538071	0.516295	0.773967
NIPA2	0.079293	0.29646	0.32578
SLC25A29	0.774926	0.142287	0.904436
C2orf69	0.613804	-0.39658	0.819264
ARL14EP	0.051109	1.34409	0.265139
ENAH	0.019371	0.528717	0.167192
BMPER	0.134221	-0.71912	0.417223
GBP7	0.641486	0.143478	0.835764
ATP6V0D2	0.981558	0.547097	1
DCBLD1	0.482211	0.219579	0.733586
GPATCH11	0.039302	0.97595	0.236259
CEP120	0.154964	-0.67407	0.444958
ABTB2	0.052564	-0.72436	0.268973
MRPL43	0.033942	0.954923	0.221141
NECAB1	0.57907	-0.16981	0.796675
C12orf29	0.15142	1.051621	0.44125
CNEP1R1	0.036574	2.022259	0.229306
JMY	0.390386	0.380385	0.668898
GDPD1	0.403464	-0.46436	0.678271
SYT2	0.58881	0.322308	0.80405
FADS6	0.169846	-0.36605	0.461029
DTX3	0.099814	0.844745	0.359216
ACOT4	0.425854	0.476366	0.693599
C19orf47	0.005152	1.822806	0.084152
TMEM102	0.197863	0.722794	0.491413
ASCC1	0.719216	-0.31561	0.874478
BANP	0.491632	0.400543	0.739769
LRRC57	0.277896	-0.24707	0.579677
EIF1AD	0.755386	-0.22034	0.895563
SCAI	0.938984	-0.04128	0.986498
KRI1	0.327317	0.63806	0.617719
TC2N	0.010795	-1.8512	0.126291
WDSUB1	0.898276	0.006977	0.970275
CCDC71L	0.960481	-0.09287	0.994122

MFSD2A	0.192968	-0.35387	0.486632
POC5	0.047752	0.988041	0.256846
ZNF579	0.845762	-0.08245	0.942239
FAM73A	0.055662	0.658401	0.275603
ZBTB38	0.838142	0.044061	0.938499
POMGNT2	0.798506	-0.21831	0.916173
PRPF38A	0.025563	0.396498	0.193268
KDF1	0.317121	-0.53802	0.613139
USP38	0.455711	0.157142	0.714286
ZNF511	0.970786	-0.0098	0.997592
MLKL	0.333853	0.521798	0.620902
PDDC1	0.199511	-0.66263	0.49217
ANKRD52	0.258033	0.486407	0.560487
ATP11C	0.162803	0.46606	0.453012
KDM1B	0.004306	1.334547	0.075057
SPATA5	0.528742	-0.14248	0.767576
DTWD2	0.691948	0.199614	0.861227
NHLRC2	0.181644	0.286421	0.472575
AVL9	0.636678	0.571927	0.833511
SLC43A3	0.671782	-0.28185	0.851286
XXYLT1	2.60E-05	2.676883	0.002074
GOLM1	0.274985	0.328643	0.577625
COLGALT1	8.89E-06	0.714185	0.000881
SUMF2	0.151386	-0.26407	0.44125
SIDT2	0.068007	1.156048	0.303928
SUMF1	0.010038	0.956003	0.12115
POGLUT1	0.382126	0.377854	0.661698
UBAC2	0.117194	0.277127	0.389929
PCYOX1L	0.157086	0.79207	0.447022
TMEM87A	0.059675	0.316123	0.285693
RDH13	0.892438	0.055841	0.966699
TTC13	0.01925	1.341709	0.167058
MFSD9	0.147665	0.840742	0.436386
PCSK9	0.402634	0.561638	0.678038
HSD17B11	0.305036	-0.18198	0.606083
FAM63B	0.019336	1.2967	0.167192
SLC4A11	0.393617	0.187472	0.671013
TXNDC5	0.434427	0.153215	0.698194
POC1A	0.078721	0.651031	0.32578
SPC24	0.031114	1.652366	0.212999
ATAD1	0.950035	0.00023	0.990863
SLC38A9	0.509993	0.487561	0.753775
SCCPDH	0.09982	0.343915	0.359216
INO80E	0.270854	0.593124	0.574167
UXS1	0.411524	0.246698	0.683152
CLEC1A		0	
ACBD4	0.08528	-0.46961	0.335537
RNF149	0.107682	0.886911	0.373221
FAM134A	0.028284	0.921787	0.203235
SERBP1	0.048208	0.325358	0.257798
KCT2	0.006708	1.993899	0.097888
LEMD2	0.044589	0.28762	0.24851
NOA1	0.050897	1.078815	0.264901
RBBP8NL	0.780512	-0.20895	0.907977
NECAP1	0.936896	-0.37653	0.986451
FAM98A	0.000119	0.666456	0.007233
ZNF784	0.384669	-0.31678	0.66413
PLA2G15	0.025773	1.165808	0.193808

TSEN2	0.8147	-0.00462	0.924847
MTMR14	0.00517	1.222514	0.084325
NFATC2IP	0.080765	1.090695	0.32836
CHST4	0.57907	-0.16142	0.796675
DAGLB	0.759058	-0.01497	0.896847
CHST14	0.000768	1.031041	0.026134
SPRYD3	0.124316	0.91746	0.400461
GALNT6	0.158635	0.895099	0.449897
APCDD1L	0.960709	0.125827	0.994122
DYNC2H1	0.565508	0.318993	0.79107
RNF169	0.250602	0.501205	0.554113
PDPR	0.067188	0.337515	0.302058
ZBTB44	0.632486	-0.16394	0.830965
ZMYM6NB	0.92213	-0.03749	0.980751
APOA1BP	0.274985	0.251345	0.577625
GALNT11	0.766238	0.158695	0.90149
MSANTD4	0.322009	0.11186	0.613139
SMG8	0.002544	1.338411	0.055387
RNF214	0.314415	0.441179	0.612305
ZNRF1	0.015471	0.794641	0.152523
PPFIBP2	0.923803	0.114781	0.980751
LSM14A	0.002438	0.796805	0.053762
GIMAP8	0.513707	0.292125	0.756141
CCNY	0.002185	0.824983	0.049962
ZNF280C	0.565716	0.433047	0.79107
PNMA1	0.001614	1.541378	0.041532
LRRN4CL	0.09909	0.537193	0.358568
HMCN2	0.448385	-0.48777	0.708512
MROH1	0.266378	-0.39111	0.568807
BANK1	0.553176	0.231845	0.783439
MAPK1IP1L	0.346163	0.926758	0.632606
PAPD5	0.23975	0.594507	0.542335
NPEPL1	0.97632	-0.03702	0.998921
EHBP1	0.037014	1.100482	0.230501
RCBTB1	0.229605	0.670854	0.529537
RBM15B	0.246548	0.607867	0.549922
SMC1B	0.936875	-0.09344	0.986451
PSD4	0.407931	0.770275	0.680763
PHC3	0.274695	0.455689	0.577625
ZNF740	0.504878	-0.3815	0.749411
C3orf58	0.154934	0.79462	0.444958
TMEM161B	0.708208	-0.22791	0.869179
TMEM104	0.472079	0.437527	0.726492
CNNM3	0.784284	-0.27194	0.909506
SETD9	0.533465	-0.22907	0.7709
FAM13C	0.571349	0.068554	0.793891
CPEB3	0.041397	0.424428	0.240826
CHDH	0.263505	-0.70312	0.565873
ABCF1	0.392997	0.099885	0.671013
MCU	0.317645	0.247302	0.613139
PIK3C3	0.226131	0.273244	0.525131
GSTCD	0.784922	-0.14505	0.909863
SRFBP1	0.336548	0.436896	0.624085
C7orf57	0.331588	-0.05914	0.619341
FAM83F	0.971656	0.004119	0.997592
NGDN	0.134442	0.876128	0.417765
DDHD1	0.233808	0.557008	0.535403
SHCBP1	0.686102	-0.02496	0.857929

SUPT20H	0.00102	1.438153	0.03092
PDZD8	0.350038	0.619941	0.635657
C1orf162		0	
LFNG	0.274366	0.883124	0.577625
NFAM1	0.137489	0.50075	0.422524
CHST13	0.540968	0.161238	0.775521
APPL2	0.005006	0.584326	0.082994
EXPH5	0.365071	-0.46573	0.64784
SLC30A7	0.434427	0.204509	0.698194
NAV1	0.019074	1.254643	0.166801
ATP6V1C2	0.232298	-0.71269	0.53408
PPHLN1	0.387654	0.923931	0.666378
VPS37A	0.079871	0.603898	0.325919
WDR19	0.752017	-0.19104	0.893287
KMT2C	0.70592	-0.42639	0.868525
FBXO22	0.312831	0.470345	0.610954
LPCAT1	0.244338	-0.36999	0.547425
DOCK8	0.407778	0.047193	0.680612
ZMIZ2	0.879142	0.070797	0.961025
SYNE1	0.426712	0.175024	0.693599
ZNF397	0.741994	-0.24614	0.887847
USP32	0.045446	0.972577	0.250872
BOD1L1	0.277869	0.729023	0.579677
ARID1B	0.173167	0.432041	0.464476
FLAD1	0.003904	0.56248	0.071121
FLCN	0.287054	0.665412	0.590535
NUP43	0.175259	0.229348	0.466796
NUP37	0.126762	0.188502	0.403998
NUP35	0.233812	0.211089	0.535403
REPS2	0.757084	-0.16818	0.896521
ENGASE	0.87149	-0.20246	0.956098
GPRC5A	0.136774	-1.14026	0.421544
BBS1	0.332803	0.46687	0.62046
B3GNT7	0.368126	0.515297	0.650971
ADCY4	0.833005	0.02975	0.935865
NBEA	0.969774	-0.01424	0.997592
TOR1AIP2	0.197211	0.241972	0.490309
STEAP2	0.005723	1.77324	0.089283
DNER	0.331588	-0.12904	0.619341
TSTD1	0.016203	-0.85609	0.154611
ABHD11	0.147661	-0.43515	0.436386
COL22A1	0.278824	0.245454	0.581068
CMAS	0.144006	0.239187	0.431666
MYRIP	0.49063	0.140972	0.738656
STXBP6	0.542041	-0.34008	0.775521
FBXO18	0.321966	0.347047	0.613139
NLGN2	0.041852	0.650321	0.242066
TNIP2	0.470605	0.242591	0.725029
CADM4	0.440265	0.481727	0.70314
HELB	0.366436	0.291499	0.649119
TSPAN14	0.035651	-0.79068	0.225452
TTL	0.546205	0.198673	0.778738
OR4A5	0.291769	0.307408	0.595183
SVIP	0.324609	0.93884	0.61511
ZNRF2	0.787688	0.188594	0.911483
ATL2	0.779221	-0.07575	0.906856
LILRB4	0.375995	0.687871	0.657546
LILRB1	0.483269	0.479561	0.73454

KDM2B	0.364616	-0.45807	0.647136
MOSPD2	0.015071	0.754379	0.150049
PLBD2	0.181644	0.337898	0.472575
RASSF8	0.229479	-0.52364	0.529537
DDX55	0.475812	0.312709	0.728899
MFSD8	0.353607	0.573242	0.638094
CT45A3;CT45A1;CT45A2;CT45A9;CT45A8;CT45/	0.156489	0.684424	0.44698
SGMS2	0.055868	0.820977	0.276127
TDRD7	0.010209	1.273091	0.121876
GIMAP7	0.206057	0.276887	0.500256
NEDD1	0.246532	0.445418	0.549922
RHOXF1	0.988991	0.05158	1
TPCN2	0.911233	-0.08854	0.975706
RFWD2	0.17207	0.643145	0.463435
GAS2L2	0.244375	-0.37817	0.547425
CDC26	0.4976	-0.14948	0.744587
NCOA7	0.015851	0.842233	0.153751
MCFD2	0.003823	0.782444	0.070612
THOC2	0.072266	0.285799	0.313701
INADL	0.733279	0.38166	0.882858
WDR36	0.537409	0.109755	0.773175
PPTC7	0.957804	0.449602	0.994122
ADCK3	0.081043	-0.50805	0.328405
ANGPTL6	0.31899	0.240638	0.613139
RP9	0.25363	0.654885	0.557407
GRPEL2	0.014387	1.226614	0.14653
VANGL1	0.203001	0.500617	0.497346
IL17D	0.68021	-0.42647	0.85545
SLC30A5	0.32407	0.044412	0.614309
OCC1	0.677236	0.059153	0.854325
SNIP1	0.074916	0.654545	0.318717
PPP1R14C	0.500641	0.486011	0.746418
GADD45GIP1	0.554864	-0.20626	0.783439
WDR48	0.913333	0.218873	0.975706
EXOC6	1	0.0175	1
FIBIN	0.000141	2.499232	0.008147
TTC8	0.553754	0.304706	0.783439
CEP76	0.393617	0.229511	0.671013
MPLKIP	0.300882	0.288812	0.602797
SMARCC2	0.18381	0.160021	0.474829
ZNF420	0.655601	-0.61076	0.84209
NPLOC4	0.003228	0.408551	0.065092
FAM76A	0.464202	0.2609	0.721065
GSDMB	0.584957	-0.36452	0.801141
CXorf38	0.133743	0.804267	0.416228
UBALD1	0.079293	0.435895	0.32578
SPATA20	0.577274	-0.1359	0.796634
RIN3	0.233092	0.372367	0.535403
GDAP1	0.440222	0.759986	0.70314
NUBPL	0.029203	-1.10476	0.20681
ABHD4	0.029665	1.042973	0.208169
DEPTOR	0.153028	0.956929	0.443165
FBXO30	0.71164	0.157561	0.870161
SLC35B2	0.442223	0.172452	0.703704
PUM2	0.02974	0.38363	0.208169
NDNF	0.277353	-0.70038	0.579677
ITFG1	0.306581	0.006426	0.608171
GOLGA5	0.422885	0.304739	0.691467

KLHDC4	0.848301	0.031373	0.944084
SHKBP1	0.261242	0.570605	0.564363
UBA3	0.151386	0.172981	0.44125
ZSCAN18	0.277055	0.661729	0.579677
BAHD1	0.992139	0.00371	1
CNIH3	0.041397	0.65098	0.240826
NANP	0.111092	1.233133	0.380668
FAM213B	0.81485	0.515842	0.924847
ZCRB1	0.211425	0.911695	0.50679
PIGX	0.042516	1.440475	0.243142
ZCCHC10	0.29846	0.713695	0.601676
TMEM254	0.60794	0.283987	0.816529
DNAJB14	0.043652	1.569698	0.247494
RAB3IL1	6.43E-06	2.944566	0.000679
FAM174A	0.442014	-0.83292	0.703704
SLC25A40	0.000363	1.740615	0.016492
TMEM167A	0.183806	0.384397	0.474829
FAM57A	0.066541	1.384587	0.301718
PIP4K2C	0.101186	0.287592	0.361901
WDR20	0.034562	1.18543	0.222216
TRMT10A	0.977916	-0.00066	1
MDM1	0.804685	0.036757	0.918848
TBC1D15	0.077655	0.3399	0.324641
RDH11	0.112657	0.162626	0.382523
TMEM163	0.021073	-1.96146	0.173882
POC1B	0.925299	0.191028	0.981917
SPATA18	0.132454	-1.04585	0.414867
FAM110B	0.163438	-0.21512	0.453012
LRRC20	0.424179	-0.41665	0.693173
IFI44	0.597324	0.663202	0.808212
MRPL30	0.169494	1.478042	0.460637
C18orf32	0.894301	-0.38127	0.968527
NT5C	1	-0.01223	1
FAM45A	0.314461	0.155541	0.612305
ZFAND1	0.216679	0.798826	0.513611
KIAA1524	0.40433	-0.60027	0.678624
PI4K2B	0.146904	0.950885	0.436111
CPT1C	0.319672	0.203723	0.613139
STT3B	0.043207	0.258542	0.246126
PNPT1	0.71923	0.08842	0.874478
SPPL3	0.039604	0.856092	0.237431
SPPL2B	0.034876	1.210057	0.223337
SPPL2A	0.687417	0.150352	0.858864
HM13	0.01039	0.415756	0.123374
PREX1	0.249711	0.321578	0.552833
DYNC2LI1	0.911648	-0.12162	0.975706
URGCP	0.137857	0.69046	0.423394
CD99L2	0.001675	2.125239	0.042312
AGR3	0.043891	-1.33629	0.247494
MIPOL1	0.834322	0.048189	0.935999
BICD2	0.064284	0.316976	0.296208
NEK9	0.286754	0.233707	0.590053
SFXN5	0.565508	0.406598	0.79107
CHD6	0.690161	-0.02356	0.859679
GPT2	0.727595	0.354316	0.879154
TRPM4	0.220749	0.797351	0.519064
CD200R1	0.553889	-0.26632	0.783439
PLEKHO2	0.627809	0.064081	0.828932

MGARP	0.404702	0.422392	0.67885
DTX3L	0.004424	0.573636	0.076312
DDX54	0.711836	0.373754	0.870161
RASGRP4	0.183878	0.248744	0.474829
BLOC1S5	0.931664	-0.42814	0.984157
DMXL2	0.033947	0.564592	0.221141
BPIFB1	0.822496	0.212996	0.92929
DLG5	0.788765	-0.26995	0.911483
CABLES1	0.077529	-0.87998	0.324641
BRIX1	0.81486	0.066616	0.924847
RNASEH2C	0.486487	0.360223	0.73612
HAVCR2	0.448456	0.322587	0.708523
CD300LF	0.17518	-0.89049	0.466796
GNPDA2	0.056188	0.42002	0.276683
TRAF3IP1	0.766633	-0.01726	0.901859
STK35	0.472922	-0.23	0.727018
HCAR2;HCAR3	0.594656	-0.08301	0.806337
LRRC8C	0.144006	0.297163	0.431666
SYTL5	0.435027	0.698968	0.699055
CSGALNACT1	0.005734	0.71284	0.089283
NEK7	0.327323	0.210523	0.617719
RB1CC1	0.952651	0.438486	0.993074
ASAP3	0.044061	1.227394	0.248311
MICAL1	0.109698	0.31018	0.377423
ELP5	0.162483	0.774079	0.453012
ADAMTS16	0.011417	1.590108	0.128971
EPS8L3	0.69426	0.074147	0.861478
EPS8L1	0.58819	0.424429	0.803499
DNAH5	0.578778	-0.3738	0.796675
SSH3	0.54177	-0.18041	0.775521
SH3TC1	0.509154	0.224105	0.753038
PXYLP1	0.025791	0.83667	0.193808
NSUN6	0.431183	0.308573	0.696172
TBCK	0.012485	0.849035	0.136424
DTD1	0.80459	0.099492	0.918848
DCAF11	0.928935	0.055546	0.983115
RHBDD1	0.336105	0.384335	0.623617
UTP15	0.028293	0.503596	0.203235
GPX8	2.95E-08	2.143237	8.86E-06
SLC37A2	0.304818	0.615583	0.606083
AFAP1L1	0.076764	1.04837	0.323352
DENND1A	0.098827	0.791106	0.358568
SH3RF3	0.251789	0.322865	0.555643
TRPC4AP	0.961101	0.018148	0.99427
NUP210	0.121915	0.241722	0.397343
CEP192	0.502425	0.057072	0.747596
SNX29	0.232345	0.502877	0.53408
GEMIN5	0.714303	0.130283	0.87164
PIGO	0.314461	-0.47703	0.612305
SNED1	0.080271	1.03599	0.326832
ARHGEF40	0.001183	1.077918	0.03367
FBF1	0.140267	-0.53331	0.426568
GANC	0.525098	-0.35332	0.764479
RAPGEF6	0.499503	0.469714	0.745391
SMCR8	0.110282	0.742452	0.378797
PARD3	0.120246	0.696078	0.39497
PARD3B	0.905154	0.019717	0.972966
IPO4	0.330591	0.233988	0.619341

CREB3L4	0.632486	-0.21085	0.830965
USP33	0.132233	0.740385	0.414428
PNISR	0.275372	0.809679	0.578109
PPP4R1	0.185984	0.268808	0.478129
DYNLRB2	0.08528	-0.38065	0.335537
WHAMM	0.122914	0.849853	0.399191
UBASH3B	0.983649	-0.05293	1
DIS3L	0.971687	0.047298	0.997592
ZFP90	0.041397	0.271403	0.240826
ATP8B4	0.932592	-0.13175	0.984157
GIPC3	0.565156	0.269862	0.790933
GIPC2	0.972044	-0.02259	0.997865
LRRC15	1.29E-09	5.314223	1.33E-06
ZNF384	0.568169	0.563709	0.791838
SLC16A10	0.293533	0.384259	0.596247
SHROOM3	0.256545	0.983133	0.560028
WIPF2	0.892331	-0.28255	0.966699
ABHD5	0.088104	0.961383	0.339301
SETD7	0.088174	0.257267	0.339301
NOC3L	0.680143	0.129293	0.85545
SCARB1	0.282463	0.752338	0.58557
COG1	0.002438	0.69611	0.053762
NPRL2	0.361802	0.334383	0.645551
GATSL3	0.374495	0.605496	0.656862
GLYATL2	0.467577	0.384194	0.722799
STK32A	0.133765	0.582173	0.416228
PYROXD1	0.123482	0.779568	0.399387
RNF139	0.0174	1.171128	0.159467
FRS2	0.346277	0.197113	0.632606
MZB1	0.550543	0.013218	0.781327
ABHD3	0.090401	1.295138	0.343901
SCFD2	0.171096	0.326347	0.461822
SMAP2	0.856059	-0.049	0.946993
ZC3H15	0.260744	0.266152	0.56373
PPIL4	0.006642	0.828294	0.097053
GTF3C2	0.041202	0.665938	0.240826
TBC1D22A	0.585477	0.46059	0.801141
TSKU	0.526694	0.459899	0.766252
PHF10	0.000302	2.256194	0.014322
RAB2B	0.094502	0.549538	0.349959
CCDC12	0.345556	-0.26736	0.632227
CHPT1	0.831352	-0.07122	0.934576
PPP1R13L	6.86E-05	1.334061	0.004699
FAM172A	0.632495	0.341449	0.830965
SLC22A17	0.178261	0.348808	0.469506
CHURC1	0.418622	0.462837	0.688796
TGFBRAP1	0.082197	0.092763	0.330671
TMEM263	0.249719	0.396347	0.552833
HDAC7	0.001217	1.836133	0.034268
STYX	0.721234	0.211109	0.876242
CYB5D2	1	-0.05683	1
CEMIP	0.000349	1.973954	0.016013
PTPMT1	0.133452	0.771965	0.416228
NUP133	0.118765	0.257859	0.392409
PDCD6IP	0.794443	0.021401	0.913708
SLC20A1	0.121075	0.508893	0.396833
UBTD2	0.070152	1.515516	0.309184
FBLIM1	2.39E-05	1.145498	0.001947

CACTIN	0.090871	0.964914	0.344564
LRRN4	0.197046	0.879743	0.490309
GATAD1	0.304902	0.797982	0.606083
BRK1	0.38573	-0.1796	0.664306
CHAC2	0.446152	0.453685	0.706795
CHMP7	0.055419	1.261559	0.274767
THEM6	0.861239	0.0207	0.950165
PRUNE2	0.871162	-0.19719	0.956098
NAT14	0.014642	1.402082	0.148033
BCL7C	0.484092	-0.95858	0.735071
ORAOV1	0.768139	-0.03363	0.901915
SFT2D1	0.902991	-0.08417	0.971583
NSMCE1	0.455535	0.318952	0.71417
PHLDA1	0.452011	0.438985	0.712009
BLNK	0.367089	0.429452	0.649965
SNX33	0.183798	0.833844	0.474829
TRIM41	0.298086	-0.35778	0.601676
PTCD2	0.068041	0.651092	0.303928
NUDT8	0.523943	0.400491	0.764023
SLC35F5	0.632804	0.244101	0.831075
MITD1	0.163509	0.82515	0.453012
LACE1	0.958791	-0.07584	0.994122
ZFAND2B	0.021542	1.345088	0.175548
HEXDC	0.455572	0.210088	0.71417
CHTF18	0.197193	0.667208	0.490309
LEO1	0.881999	-0.14738	0.961515
DCAKD	0.559385	0.211448	0.78633
RNF141	0.565774	0.385976	0.79107
N6AMT2	0.167109	0.311107	0.457139
TMEM170A	0.102462	-0.56378	0.364372
OSCP1	0.496712	-0.40646	0.74366
SMIM4	0.72732	0.21495	0.878927
NUDCD2	0.586319	-0.1615	0.801141
SNRNP27	0.133789	1.397709	0.416228
SKA2	0.540968	0.145602	0.775521
ANKRD49	0.453445	0.402051	0.712914
TFB1M	0.539269	-0.03036	0.775046
STAG1	0.006511	0.511924	0.096023
SCFD1	0.021148	0.248014	0.173882
SECTM1	0.401678	-0.43524	0.677774
UBE2Q2	0.699852	-0.26642	0.864371
TNFAIP8L1	0.436915	0.162798	0.701684
CANT1	0.032856	0.6307	0.217297
C7orf43	0.009851	1.334779	0.120067
WDR60	0.481993	-0.10253	0.733586
TRAPPC12	0.2958	0.213767	0.598344
POF1B	0.029211	2.480555	0.20681
HNRNPLL	0.002185	0.409071	0.049962
C4orf3	0.139034	1.548381	0.4248
FAR1	0.029444	0.929112	0.207345
UBLCP1	0.364426	0.25684	0.646996
TSEN15	0.186259	1.278639	0.478636
PCNP	0.498977	0.300328	0.744839
DNAJA4	0.763569	0.381481	0.899242
TEKT4	0.157319	-0.38791	0.447277
GTSF1	0.57907	-0.12373	0.796675
APH1B	0.683896	0.25629	0.85709
SPRYD4	0.383925	-0.15819	0.66367

TMED6	0.563678	-0.23617	0.789479
ITLN1	0.969086	-0.13602	0.997592
TMEM40	0.321952	0.479689	0.613139
GLMP	0.091899	0.69827	0.345626
C2orf47	0.794443	0.119071	0.913708
CAPSL	0.5199	0.355407	0.760272
TRUB1	0.260147	0.586847	0.56373
LMO7	0.586319	-0.0269	0.801141
SLC44A1	0.023065	0.547445	0.182986
CKAP2	0.133624	0.571377	0.416228
ATXN2L	0.014268	0.496672	0.145444
CYGB	0.638143	0.376759	0.834211
ARAP3	0.050169	1.074959	0.263211
GIMAP1	0.704483	-0.09565	0.867291
PHIP	0.934288	0.059259	0.984157
HPSE2	0.192496	0.40791	0.486094
STAB2	0.479442	-0.48214	0.731833
RTN4IP1	0.892102	-0.31219	0.966699
RASSF5	0.875701	-0.25864	0.958859
SELM	0.003514	0.653056	0.067901
PRPF31	0.003987	0.435964	0.071551
LIPH	0.324878	-0.90311	0.615409
EDARADD	0.310183	0.255382	0.609864
IGFBPL1	0.683728	-0.07085	0.85709
NELFB	0.466082	0.261176	0.721065
PALLD	8.99E-08	1.188256	2.25E-05
RUFY2	0.059998	1.005439	0.286703
SREK1	0.524435	0.364978	0.764023
MYEOV2	0.34755	1.136353	0.632849
GEMIN6	0.212622	1.315738	0.508813
CASKIN2	0.096063	0.972068	0.352925
ATRIP	0.014132	1.862024	0.144889
STON2	0.104134	1.009666	0.367349
PSPC1	0.442223	0.195695	0.703704
ATL1	0.026447	0.296967	0.195939
RSAD2	0.251789	-0.65551	0.555643
MADD	0.116249	0.640109	0.389168
SYNE2	0.981581	-0.00262	1
ACOT11	0.359911	-0.68293	0.644411
MUC16	0.313197	0.530743	0.611346
GATAD2B	0.006317	0.966421	0.094168
MYO3B	0.166815	0.634245	0.457139
PIBF1	0.115261	0.727967	0.387731
DNAJC9	0.00094	0.606669	0.02929
AUTS2	0.421207	0.083758	0.691066
BRI3BP	0.033942	0.481244	0.221141
BBX	0.113096	0.803085	0.383358
THAP4	0.565604	0.180396	0.79107
TMEM234	0.322009	0.124649	0.613139
IFT81	0.789191	0.112154	0.911483
CTNNBL1	0.074384	0.216351	0.317894
ING5	0.101434	1.017143	0.362438
SEPTIN1	0.611442	-0.60351	0.818417
SSH1	0.003733	1.422115	0.070138
ATG4A	0.883716	-0.05718	0.963083
RIN2	2.40E-05	2.226782	0.001947
AHCTF1	0.081043	0.417236	0.328405
CHCHD10	0.141292	0.720884	0.427981

DGCR8	0.14785	0.646815	0.436815
ZCCHC14	0.57907	-0.12295	0.796675
PIK3R5	0.15526	0.20049	0.444958
RSPH1	0.688686	-0.18126	0.859429
TMEM190	0.08528	-0.4205	0.335537
KLHL6	0.046096	1.481217	0.252472
ARAP2	0.233429	0.899239	0.535403
RFFL	0.302856	0.260681	0.604766
ROBO4	0.08528	-0.18539	0.335537
OVCA2	0.263505	0.480167	0.565873
LZIC	0.609213	0.12653	0.816529
POMGNT1	0.582211	0.051669	0.798306
IRGQ	0.54177	0.046201	0.775521
ST8SIA4	0.193164	0.738652	0.487015
DDB2	0.255036	0.417621	0.557593
CLEC2B	0.105526	1.371026	0.369791
SMPDL3A	0.988097	-0.03356	1
SMPDL3B	0.274985	0.020839	0.577625
DDX1	0.030249	0.155761	0.209418
STARD8	0.310183	-0.28961	0.609864
SEC14L1	0.029828	1.061881	0.208196
SLC39A7	0.667966	-0.26196	0.849281
HSD17B8	1.32E-05	-1.32224	0.00119
PIEZO1	0.02302	0.842539	0.182986
FAM3C	0.162993	0.430748	0.453012
PIGB	0.122914	0.677219	0.399191
H1FX	0.794443	0.063417	0.913708
CPT1B	0.423133	0.16618	0.69177
PSMF1	0.221112	0.279839	0.519064
SLC7A6	0.358301	0.617393	0.642612
GBF1	0.00511	0.364319	0.083708
LPIN2	0.728634	-0.20832	0.879656
SMG7	0.876779	-0.11822	0.958859
RTF1	0.840548	0.371726	0.939212
NCSTN	0.11568	0.239759	0.387731
SNX19	0.176103	0.860308	0.468045
TM9SF4	0.415291	0.149136	0.686028
TMEM131	0.843384	-0.11943	0.941517
RGP1	0.002532	2.108608	0.055387
TOPBP1	0.263695	-0.51639	0.565913
IP6K1	0.337822	0.32458	0.625298
MRPS27	0.233812	0.255269	0.535403
ELMO1	0.32085	0.091426	0.613139
WASF1	0.670768	-0.08621	0.850681
BAP1	0.00394	1.167315	0.071362
FIG4	0.1197	0.700549	0.394412
SPOCK2	0.852558	-0.02254	0.946235
NR4A3	0.556057	0.146599	0.783439
AP3S1	0.008743	0.738252	0.112752
TSC1	0.257912	0.527311	0.560462
UBXN4	0.595428	0.171362	0.806337
PHF3	0.033844	1.036008	0.221141
SLC9A6	0.041883	0.949148	0.242067
MAML1	0.366436	0.230145	0.649119
NDRG1	0.298858	0.265577	0.601676
HSPH1	0.305036	0.179989	0.606083
SEPTIN8	9.45E-06	0.705303	0.000928
RQCD1	0.005006	0.426536	0.082994

LPGAT1	0.049723	0.615254	0.261364
DOCK2	0.289748	0.130069	0.593134
TBC1D5	0.255193	0.49065	0.557593
ZNF592	0.091287	0.846188	0.345435
EDEM1	0.149664	0.520603	0.439016
JADE3	0.601315	0.209035	0.811336
MYO18A	0.76912	0.038944	0.901915
LARP4B	0.458049	-0.11417	0.715224
GCN1L1	0.072266	0.230063	0.313701
ZNF516	0.249679	0.499383	0.552833
HMHA1	0.689846	-0.16582	0.859429
DHX38	0.000337	0.874848	0.015578
NUP205	0.794443	-0.11178	0.913708
KIAA0226	0.333105	0.465184	0.620722
TTC9	0.676745	-0.27444	0.854135
APPBP2	0.932488	-0.13038	0.984157
ANKS1A	0.623137	-0.29062	0.825978
PXDN	0.011419	0.57671	0.128971
SGCD	0.009443	0.826658	0.117552
LPAR1	0.771591	0.096404	0.903336
NSMAF	0.117602	0.793236	0.391103
PIGK	0.085745	0.358111	0.335537
MRPS31	0.233812	0.277933	0.535403
AKAP1	0.561596	-0.11224	0.788865
SORL1	0.913333	0.08494	0.975706
ALG3	0.052176	0.848115	0.267704
ANP32B	0.438315	-0.16158	0.701933
PVRL2	0.031529	0.363208	0.214006
RABGGTA	0.934288	-0.18091	0.984157
PTPRU	0.930143	-0.0246	0.983996
PRCC	0.262943	0.584145	0.565873
TFG	0.029749	0.319313	0.208169
USP6NL	0.503122	-0.35717	0.747596
HTRA1	0.002838	0.7086	0.060074
ARPC1A	0.041202	0.269545	0.240826
TNR	0.382354	-0.18427	0.661883
TFAP2C	0.671985	0.331996	0.851286
GTF2H4	0.014209	1.132196	0.145268
KRT35	0.05372	-1.26825	0.271194
FRZB	0.221202	0.954788	0.519118
RREB1	0.778432	0.231028	0.906856
HDAC2	0.001829	0.620432	0.044883
STAM	0.00841	0.761604	0.110127
DPF3	0.145032	0.611687	0.433688
DPF2	0.866426	0.041041	0.953042
PROX1	0.976419	0.030626	0.998921
LEPREL4	2.23E-18	3.704144	2.47E-14
CREBBP	0.124973	0.775116	0.401334
KAT6A	0.988816	0.055776	1
DLG3	0.218354	-0.24809	0.515816
SYMPK	0.020781	0.364236	0.172048
EZH1	0.853692	0.088759	0.946925
TAF15	0.637197	0.150693	0.833511
GOLGA1	0.052064	0.849104	0.267367
EVPL	0.017103	-0.80971	0.157941
GGH	0.034511	0.422036	0.222148
NRCAM	0.907445	0.044756	0.9734
PCSK5	0.131365	0.275695	0.413476

CORO2A	0.537405	-0.29611	0.773175
KAT2A	0.265744	0.423438	0.568807
NELL1	0.929566	-0.1079	0.983573
INPP5D	0.43056	0.117295	0.695651
DDX17	0.063339	0.177879	0.294055
BCL2L2	0.471676	0.49729	0.726074
TANK	0.027815	1.112217	0.202072
KIFAP3	0.939399	0.161498	0.986498
CASP10	0.434426	0.457671	0.698194
SEMA4D	0.853939	-0.08557	0.946993
NEO1	0.855612	0.357971	0.946993
APBB2	0.051558	1.047011	0.266309
PMM1	0.356402	-0.29517	0.641385
KLK6	0.135713	0.80444	0.419275
RAD50	0.039276	0.254144	0.236232
CELF1	0.05451	0.384841	0.273313
OSTF1	0.039276	0.250182	0.236232
ABCC2	0.78961	0.254723	0.911676
ARHGEF1	0.450097	0.061257	0.709598
ERCC4	0.078755	0.315594	0.32578
UFD1L	0.135171	0.177358	0.417833
GLG1	0.001332	0.484961	0.036119
UPF1	0.142204	0.188461	0.428816
HPS1	0.40975	0.303155	0.68267
CDS1	0.276465	-0.80439	0.579677
DAZL	0.15526	0.394233	0.444958
COPS5	0.177368	0.161849	0.468602
GATA6	0.671735	-0.16362	0.851286
FGF13	1	-0.0007	1
GPKOW	0.1422	0.532459	0.428816
MAP4K1	0.637439	0.318083	0.833511
SMARCC1	0.058787	0.61599	0.283235
SMARCD2	0.533067	0.120843	0.7709
RAB8B	0.515876	-0.07015	0.757825
BAD	0.351365	1.112375	0.636424
KHSRP	0.641914	0.099495	0.835764
GCDH	0.324076	-0.36907	0.614309
PRG4	0.533637	0.314926	0.771048
TNFRSF14	0.183419	0.889226	0.474829
SLCO2A1	0.783813	0.012146	0.909506
RIT1	0.031822	1.265597	0.214292
PEX13	0.020289	1.341185	0.170514
TNPO1	0.520146	0.08281	0.760272
ARHGEF2	0.260744	0.198144	0.56373
EMG1	0.068179	0.266903	0.303928
NINJ1	0.274193	0.775521	0.577625
IRF7	0.138572	0.916568	0.424105
CLP1	0.04752	1.147268	0.256066
GLMN	0.187853	0.672719	0.480067
KAT5	0.418567	0.460961	0.688796
BRF1	0.628247	-0.28415	0.829411
USP13	0.822828	-0.12979	0.929468
DVL3	0.0247	1.483734	0.190022
USP9X	0.311298	0.108661	0.609894
USP7	0.280828	0.073737	0.583434
NAT6	0.013213	-0.74525	0.140906
CD101	0.861859	0.07021	0.950565
CUL5	0.357491	-0.14926	0.641472

STMN2	0.756519	-0.36011	0.896333
ATP6V0A1	0.04896	-0.3254	0.259694
LPP	0.001913	0.420156	0.046318
RBPMS	0.546146	0.431492	0.778738
EXT2	0.081727	1.077645	0.330094
ART4	0.247596	-0.58629	0.55087
SECISBP2L	0.822927	-0.13438	0.929468
MED12	0.00584	1.089644	0.089662
HIST1H2AC	0.041461	-2.13735	0.241008
HIST1H2BH;HIST2H2BF	0.381971	-0.10015	0.661698
ATP2A3	0.774166	-0.02812	0.903913
BHMT	0.686212	-0.45416	0.857929
RNASE6	0.017795	1.037451	0.161507
PTP4A1	0.629589	0.314922	0.830392
WNT2B	0.322271	-0.31544	0.613404
HGD	0.610306	0.267472	0.817501
PHKB	0.155179	-0.45428	0.444958
HLA-B	0.016955	2.16898	0.157494
MR1	0.012351	1.709197	0.135633
HLA-DRB1	0.675402	0.520834	0.853176
SCAMP4	0.550538	0.324752	0.781327
TCEAL3;TCEAL5	0.110195	-1.37042	0.378734
TSR2	0.889651	0.239531	0.966133
GTF3C6	0.874646	0.003913	0.958094
HPS3	0.002063	1.383081	0.048162
SMARCE1	0.367925	0.171206	0.650718
PRKCDBP	0.554955	0.194556	0.783439
RFK	0.243019	-0.89619	0.546608
CNKSR1	0.729777	-0.46808	0.880161
POP5	0.041014	1.144848	0.240826
MYDGF	0.286754	0.148147	0.590053
GLYATL1	0.397751	0.457307	0.675257
ZKSCAN4	0.137489	0.38002	0.422524
LOH12CR1	0.003507	-1.2934	0.067901
RNF34	0.167504	0.463892	0.457358
MAL2	0.611514	-0.50322	0.818417
TOMM40L	0.020469	1.596558	0.170697
YIPF5	0.784217	0.703498	0.909506
UBE2F	0.309006	0.520705	0.609864
PIGT	0.037428	0.352781	0.231646
IGSF8	0.26918	0.189111	0.571371
TOP1MT	0.027102	0.745184	0.199066
RPL36AL	0.866418	-0.17538	0.953042
ARL11	0.133765	0.57924	0.416228
RAB24	0.568286	-0.04699	0.791838
OSBP2	0.374482	0.359417	0.656862
L3MBTL2	0.067342	0.69374	0.302148
ITFG2	0.923804	-0.02057	0.980751
NEIL2	0.260666	0.46496	0.56373
ZNF622	0.003086	0.941997	0.063034
HDAC10	0.120749	-0.88442	0.395987
GFM2	0.507366	0.128421	0.75153
SNX21	0.447881	0.37738	0.708118
NT5C3B	0.787721	-0.07539	0.911483
WBP2	0.861156	0.81521	0.950165
FBXW5	0.373409	0.342812	0.656544
PSMG2	0.45305	1.145153	0.712914
NCLN	0.026002	0.344972	0.193808

TEKT1		0.181133	-0.33421	0.472575
MUL1		0.273387	0.03481	0.57755
MKL1		0.004167	1.277917	0.073586
RILPL2		0.726238	-0.16173	0.878089
TMBIM1		0.94461	-0.35124	0.988547
ERGIC1		0.97106	-0.05088	0.997592
CIRH1A		0.311298	0.181478	0.609894
GTPBP3		0.734024	-0.18075	0.882858
TBRG4		0.105374	0.593007	0.369374
	2-Mar	0.015071	-0.66781	0.150049
PPP1R14A		0.398866	1.018137	0.676219
HIST1H2BA		0.007217	-1.49329	0.101678
CCDC102A		0.905372	0.181998	0.973106
C11orf52		0.118954	-1.08528	0.392655
CPNE4		0.901067	0.104478	0.971583
FAM162A		0.112657	0.504294	0.382523
SLC35C1		0.299936	0.776182	0.602797
MYLPF		0.683128	0.194689	0.85709
CCDC47		0.157109	0.177113	0.447022
MRPL24		0.637197	0.12848	0.833511
RNF166		0.266479	0.216217	0.568807
SLC25A28		0.359903	0.297162	0.644411
SYAP1		0.098469	-0.17211	0.357719
TMEM230		0.329059	0.54418	0.619341
RERG		0.051749	-0.74291	0.266457
EXOC4		0.260744	0.154663	0.56373
MAGOHB		0.031688	1.100093	0.214212
KIAA1191		0.701917	0.378206	0.866168
COL26A1		0.131554	1.815922	0.413476
RFT1		0.055344	0.523449	0.274516
ISOC2		0.162993	0.157107	0.453012
NTAN1		0.151757	0.846527	0.441884
FERMT2		0.149515	0.236666	0.438811
PNPLA2		0.88873	0.087956	0.965603
FUBP1		0.43056	0.172477	0.695651
TTC17		0.323964	0.321799	0.614309
SLC25A46		0.477834	0.318684	0.730754
LRRC59		0.009444	0.308013	0.117552
RAB7B		0.01393	1.707227	0.144006
CLUAP1		0.545115	0.248133	0.778738
VTI1A		0.160074	0.904147	0.452557
APOBEC3D		0.276362	-0.30883	0.579677
MRGPRF		0.307968	0.687833	0.609277
TMEM143		0.287608	-0.59408	0.591153
ACD		0.447084	0.32386	0.707261
ESAM		0.913246	-0.13442	0.975706
TMEM125		0.057058	-1.24962	0.278658
PBXIP1		0.327323	0.274997	0.617719
MCUR1		0.426503	0.425611	0.693599
KIAA1143		0.278808	0.622637	0.581068
RPE		0.734083	-0.1767	0.882858
VPS33A		0.057044	0.255787	0.278658
RAB37		0.235524	-0.78172	0.53771
MIB2		0.001082	1.533278	0.031645
FKBP10		1.91E-10	1.769876	3.84E-07
TTC28		0.019173	1.046085	0.166914
ISG20		0.76912	-0.19803	0.901915
UBE2W		0.041397	0.633192	0.240826

C18orf25	0.087925	-1.24908	0.339301
EXOSC8	0.082198	0.298664	0.330671
CLDN23	0.440294	-0.26545	0.70314
AKT1S1	0.037422	0.97775	0.231646
C10orf32	0.641882	-0.5069	0.835764
TOMM6	0.268747	-1.24199	0.571371
ZNF428	0.447813	0.894474	0.708118
LENG9	0.379847	0.592323	0.661258
TMEM186	0.157076	0.925432	0.447022
RGMA	0.300882	0.253835	0.602797
TMEM159	0.2111	1.443073	0.506632
SH3KBP1	0.343873	0.17081	0.630156
CREB3L1	0.710688	-0.01931	0.870136
SLCO4A1	0.626197	0.120051	0.828579
PHF21A	0.927662	0.078836	0.983115
SKA1	0.00607	0.820829	0.091588
NACC2	0.319916	0.4154	0.613139
RNF25	0.498835	-0.49065	0.744839
SLC22A18	0.199364	0.560909	0.49217
APH1A	0.429904	0.511622	0.695651
AIDA	0.33719	0.371106	0.624371
ELMO3	0.096906	-0.84523	0.354489
PINX1	0.204751	0.789667	0.499097
ARL8A	1	0.018902	1
TADA1	0.118513	0.903431	0.392409
OTULIN	0.109483	0.913608	0.377423
CHCHD1	0.747938	0.266878	0.890902
PPWD1	0.02267	0.449486	0.180886
FAM3D	0.759283	-0.29024	0.896923
CCDC127	0.251071	-0.53736	0.554953
SGK3	0.944222	-0.0521	0.988547
COA7	0.670506	-0.24925	0.850681
TESC	0.711208	0.380567	0.870136
CENPT	0.384669	0.200846	0.66413
ALKBH8	0.430232	0.271061	0.695651
UPRT	0.542438	0.914563	0.775781
PTER	0.274972	-0.90121	0.577625
TAMM41	0.101842	0.799923	0.363392
MOB3A	0.541018	0.635593	0.775521
DOCK10	0.078771	0.533209	0.32578
ATG2B	0.089183	0.885541	0.341477
SARAF	0.762178	0.206232	0.899242
PLD4	0.535404	0.351716	0.772696
TBC1D20	0.025164	1.12773	0.191949
ZBTB9	0.085209	0.75106	0.335537
FAM136A	0.105374	-0.48099	0.369374
DHX58	0.280854	0.270751	0.583434
FGGY	0.803024	-0.1098	0.918848
ARMC5	0.599951	-0.61319	0.810262
EFHD2	0.138653	0.379385	0.424105
GALM	0.360948	-0.20321	0.644411
SYTL4	0.557951	0.427721	0.785807
RUNDC1	0.657918	-0.18759	0.842898
PYCR2	0.568296	0.114361	0.791838
ZNF524	0.076728	-0.72428	0.323325
C12orf43	0.122179	1.006605	0.397854
ROPN1L	0.612037	-0.11471	0.81882
DCPS	0.494799	-0.16116	0.741997

PPP1R14B	0.830217	0.097972	0.933615
SDCCAG3	0.122635	0.854744	0.39887
INTS12	0.002174	2.023848	0.049962
NSUN4	0.524424	0.175614	0.764023
RHBDF1	0.818371	0.407639	0.92687
FBXL8	0.383036	0.869774	0.662548
PPCDC	0.64158	-0.04702	0.835764
TM4SF18	0.567751	-0.2513	0.791838
CHMP4C	0.578817	0.503029	0.796675
TIFA	0.337913	0.664695	0.625324
CTHRC1	7.64E-10	3.609685	1.06E-06
RPUSD4	0.035909	0.851513	0.226677
ACSF2	0.537409	-0.00015	0.773175
EVI5L	0.142815	0.845927	0.430424
LRRC45	0.829882	0.115319	0.933615
ISOC1	0.314461	0.228808	0.612305
GCC1	0.749036	0.036938	0.890902
FLYWCH2	0.58391	0.515893	0.800019
GRAMD1A	0.044191	1.407517	0.24851
TLCD1	0.633541	-0.34374	0.831846
SLC25A36	0.84226	-0.07325	0.940547
HAUS1	0.087821	1.127095	0.339301
FAF2	0.031269	0.228836	0.212999
PLEKHB2	0.674987	0.239573	0.853175
CCDC124	0.117214	0.213571	0.389929
FOXRED1	0.030485	0.65916	0.210793
OPTN	0.005543	0.535494	0.087699
AP2M1	0.077655	0.191705	0.324641
TUBGCP3	0.000554	0.54017	0.021722
SLC7A6OS	0.518128	0.408575	0.760272
KCTD12	0.045294	0.332824	0.250872
ZNF501	0.045488	-0.4169	0.250872
LRRC58	0.189828	0.577361	0.483202
C10orf35	0.932715	-0.14509	0.984157
RCN3	1.33E-08	1.56809	6.13E-06
ORAI1	0.714734	0.196953	0.871878
NMD3	0.000596	1.009934	0.022651
ADCK4	0.460039	0.297747	0.717621
REPS1	0.675324	0.180098	0.853175
HVCN1	0.61848	0.133544	0.822194
ZG16B	0.678919	-0.42085	0.855378
RAB39B	0.095807	0.40638	0.352331
DNAJC19	0.784217	0.091897	0.909506
HDAC11	0.943701	0.080754	0.988547
RMDN1	0.779221	0.04518	0.906856
TMCO6	0.181133	-0.32744	0.472575
ECHDC3	0.018852	-1.04609	0.166474
NUDT16	0.819984	0.008298	0.92687
ANAPC16	0.530095	0.348194	0.768664
DGCR14	0.002608	1.812931	0.056494
CMBL	0.57727	-0.14649	0.796634
MSI2	0.866426	-0.09351	0.953042
SNRNP40	0.442223	0.115077	0.703704
C18orf8	0.04896	0.492483	0.259694
MTFMT	0.756861	-0.3343	0.896353
ARHGEF26	0.949005	0.115709	0.990863
LGALS12	0.163587	0.580379	0.453012
ATG4C	0.083398	0.948708	0.332962

ZBTB10	0.06058	-0.88957	0.287134
SLAMF6	0.681268	0.190486	0.856391
ITPKC	0.453217	0.441996	0.712914
MRPL38	0.632496	0.256668	0.830965
SLC25A38	0.161733	0.386177	0.453012
RSPRY1	0.000939	1.458435	0.02929
ASB9	0.054703	-1.28716	0.273787
RTP4	0.402634	0.470905	0.678038
ERLEC1	0.034511	0.320194	0.222148
CMTM5	0.696781	-0.06881	0.863213
MRRF	0.411416	-0.23316	0.683152
RMI2	0.104774	0.756663	0.368787
SMIM19	0.442884	-0.62815	0.704334
MTERF3	0.060747	1.227839	0.287678
RBMXL1	0.360947	-0.55213	0.644411
C9orf9	0.980552	0.063774	1
OMA1	0.221996	-0.64821	0.520431
ELP4	0.033956	1.034989	0.221141
SIRT1	0.203522	0.713479	0.497963
YIPF6	0.11416	-0.0151	0.385201
HOOK2	0.989472	0.111068	1
SEH1L	0.236413	0.101172	0.537858
CCDC126	0.005451	2.152625	0.086993
FBXO17	0.121356	0.550653	0.397271
MALSU1	0.918415	-0.01278	0.978657
TCEAL4	0.297811	0.990438	0.601315
THAP11	0.293316	0.833323	0.596247
KIAA1279	0.167007	0.552318	0.457139
GNPNAT1	0.350614	0.734767	0.635657
FAM120B	0.773231	-0.10746	0.903913
KTI12	0.409681	0.509998	0.682657
MRPS24	0.149452	0.450282	0.438811
MRPL53	0.641882	-0.39655	0.835764
L3HYPDH	0.000593	1.629543	0.022651
MOCOS	0.188151	-0.46802	0.480067
RNF31	0.031269	0.535985	0.212999
DAZAP1	0.819984	-0.03184	0.92687
SLC10A4	0.15526	0.34433	0.444958
SGTB	0.341196	0.715359	0.628291
SAAL1	0.238285	0.57995	0.540624
CCDC51	0.887225	0.080964	0.96416
MFSD3	0.575621	-0.34592	0.796634
CCDC101	0.29979	0.222932	0.602797
TVP23C	0.963074	-0.04282	0.995704
RRP36	0.416163	0.536705	0.68706
C1GALT1C1	0.42933	0.419179	0.695651
RBM33	0.042445	0.752326	0.243142
DTNBP1	0.028747	0.87055	0.204946
HSPBAP1	0.205597	0.580222	0.499952
SMIM12	0.441778	-0.51941	0.703704
WDR34	0.739737	0.061049	0.886374
DNAJA3	0.908103	0.031655	0.9734
TMA16	0.337143	0.559322	0.624371
MVB12A	0.218562	-0.42165	0.515816
PTCD3	0.216173	0.184138	0.512741
MMAB	0.490639	-0.34271	0.738656
MCRS1	0.069824	1.035496	0.308533
CYFIP2	0.850885	-0.26642	0.944945

SAT2	0.7507	-0.212	0.892487
GIMAP5	0.389327	-0.62478	0.667643
NRBF2	0.126025	1.049196	0.403148
ALG14	0.414218	0.874461	0.685923
TRIM11	0.224644	0.719443	0.523685
ZNF503	0.505111	0.295785	0.749557
IL17RA	0.131255	0.683523	0.413476
CCDC97	0.128872	1.107741	0.408844
DISP1	0.555312	-0.02369	0.783439
CNRIP1	0.704468	0.492603	0.867291
EDC3	0.004554	1.008536	0.078191
PELI1	0.339213	-0.12342	0.626409
PHYHIPL	0.045488	-0.59555	0.250872
DDX11	0.144556	-0.66413	0.433053
PIK3IP1	0.237941	-0.83037	0.540557
MISP3	0.004366	-2.03444	0.075776
CDCA5	0.06188	0.934901	0.290811
MEF2BNB	0.426727	-0.55974	0.693599
NEIL1	0.65654	-0.08285	0.842398
STAMBPL1	0.200884	0.835222	0.494233
DYNLL2	0.934288	0.230211	0.984157
WDR89	0.911082	-0.1095	0.975706
SLC47A1	0.559081	-0.19469	0.78633
GALNT14	0.219991	0.810539	0.518019
PGAP3	1	-0.03274	1
CPNE2	0.981581	0.065172	1
DTD2	0.622547	0.447074	0.825978
S100A16	0.173168	0.369308	0.464476
SIPA1	0.121915	0.341563	0.397343
IFT43	0.181989	0.676268	0.473145
LRRC46	0.664325	0.027815	0.847289
SCRN2	0.858648	0.031351	0.949134
THOC1	0.255193	0.292043	0.557593
OTUB1	0.533067	0.088938	0.7709
TRMT61A	0.776594	0.107863	0.905985
PERP	0.156489	0.570793	0.44698
HMCES	0.098646	1.039673	0.358247
CMTM7	0.726229	-0.32765	0.878089
CHMP6	0.520146	-0.1218	0.760272
BICD1	0.000628	1.584008	0.023434
PGM2	0.997369	-0.02161	1
IMP4	0.399288	0.742058	0.67622
CERS2	0.135171	0.018005	0.417833
MED8	0.170668	0.817349	0.461822
CFAP36	0.275784	0.75381	0.578864
KLHDC7B	0.045488	-0.52624	0.250872
DUS3L	0.071626	1.19207	0.312422
P2RY11	0.119644	0.550066	0.394344
BSCL2	0.341824	0.364381	0.629182
LTV1	0.196229	0.934487	0.490309
SDSL	0.108886	0.628025	0.375864
MRPL48	0.739057	0.186455	0.886253
VMP1	0.008089	0.640641	0.107782
PDXP	0.694266	0.364882	0.861478
SCMH1	0.438587	-0.2838	0.701933
AURKB	0.380481	0.46937	0.661698
CEP95	0.937694	-0.28815	0.986498
CALML4	0.992139	-0.08814	1

TMEM261	0.140784	1.450348	0.42744
DCUN1D1	0.389353	0.230649	0.667643
FAM89A	0.386649	0.505214	0.665564
TRMT2B	0.964759	-0.09269	0.99616
FAHD2A	0.434427	0.194483	0.698194
SMARCD1	0.169002	0.624102	0.459676
TOE1	0.894767	0.017086	0.968678
CDCA7L	0.083536	0.536138	0.333074
SCARF2	0.000238	2.395	0.011933
C16orf58	0.929044	0.027894	0.983115
DDX27	0.680152	0.183991	0.85545
ZDHHC12	0.500258	0.236427	0.745947
C17orf59	0.759058	0.228193	0.896847
ABHD17A	0.056154	0.983989	0.276683
XAGE2	1	-0.01279	1
PAGE5	0.163438	-0.35409	0.453012
MARS2	0.855881	0.151	0.946993
TCTN2	0.095807	0.330428	0.352331
ATXN7L3B	0.667409	0.346332	0.848783
MASTL	0.152726	0.505171	0.443165
APIP	0.618461	-0.31146	0.822194
ZC2HC1A	0.699506	0.253903	0.864146
LIN37	0.039777	0.756021	0.238337
SLC41A3	0.364209	-0.47483	0.646996
SNF8	0.316049	0.21141	0.613139
CENPN	0.141292	0.476972	0.427981
MYO19	0.175405	0.725585	0.466796
SLC25A44	0.813672	-0.05524	0.924847
ZC3HAV1L	0.050493	0.683775	0.263221
POM121	0.924567	0.165127	0.981468
TONSL	0.048699	0.853179	0.259424
WDYHV1	0.902849	0.037745	0.971583
PEX11G	0.561615	0.42804	0.788865
PDLIM5	1.00E-06	0.907725	0.00015
CRELD1	0.233812	0.20645	0.535403
ACY3	0.260814	-0.50625	0.56373
ERO1L	0.038035	0.391055	0.233324
SFRP2	1.30E-09	4.974984	1.33E-06
TMEM19	0.988567	-0.00251	1
GRAMD3	0.020498	1.596304	0.170803
SENP5	0.675707	0.223421	0.85327
MS4A3	0.167504	0.919623	0.457358
C7orf55	0.398866	-0.26823	0.676219
SH3YL1	0.808103	-0.10941	0.921586
AHCYL2	0.1915	-0.44144	0.485658
DOCK6	0.419078	0.241184	0.688796
OXNAD1	0.012687	1.541401	0.13791
TMEM176A	0.079293	0.40411	0.32578
CDKN2AIPNL	0.274246	0.933236	0.577625
MED30	0.928956	-0.20299	0.983115
NAF1	0.111992	-0.95506	0.382523
REEP6	0.256192	1.292653	0.559666
PGAM5	0.364426	0.199921	0.646996
SGSM3	0.354411	-0.39528	0.63835
TMEM41A	0.194887	0.542336	0.488911
INTS4	0.006911	0.673448	0.099675
DDRGK1	0.14042	0.244651	0.426568
DHTKD1	0.76912	0.168419	0.901915

SCLY	0.960543	0.381029	0.994122
PYURF	0.048094	-1.33341	0.257798
FUBP3	0.744041	0.09307	0.888303
RBM17	0.286754	0.488678	0.590053
PPP1R16A	0.642109	0.123588	0.835764
TMEM141	0.301331	-0.84857	0.603275
WBSCR16	0.853471	0.003121	0.946774
NARS2	0.581788	-0.12461	0.797998
GPATCH3	0.241325	0.378127	0.544139
SUCLG2	0.060574	-0.23954	0.287134
AJUBA	0.642079	0.378115	0.835764
FBXL20	0.400321	0.303313	0.67622
LRCH3	0.028293	0.6008	0.203235
GMPPA	0.317645	0.277052	0.613139
TMEM101	0.032252	1.532039	0.215938
BOD1	0.97705	0.001531	0.999305
GMCL1	0.322271	-0.29438	0.613404
FAM46A	0.274038	0.410922	0.577625
VSIG2	0.139571	-0.86591	0.426206
ZNF414	0.067371	0.688636	0.302148
ZNF845	0.045349	0.719188	0.250872
ABHD14B	0.083367	-0.36824	0.332962
NGLY1	0.061021	0.652098	0.28873
FAXDC2		0	
SEC22A	0.195232	0.505689	0.489337
USMG5	0.764084	0.031625	0.899242
NSL1	0.048254	1.319059	0.257919
CPB2	0.003018	1.905483	0.062294
PAWR	0.004516	0.950917	0.077667
RBM41	0.096712	0.574181	0.354489
RSRC1	0.112838	0.945739	0.382833
THOC3	0.45805	0.354133	0.715224
ITCH	0.015914	0.392841	0.153751
TXNDC15	0.110812	0.205168	0.380148
KIRREL	0.000971	1.473811	0.029997
EPSTI1	0.163707	0.734658	0.453012
LRIG1	0.126143	0.807872	0.403301
COG3	0.147661	0.283172	0.436386
CDK5RAP3	0.61848	0.069518	0.822194
LOXL4	0.468054	-0.20835	0.722799
VPS39	0.078771	0.312995	0.32578
CCDC132	0.255193	0.221974	0.557593
MAGED4	0.222683	0.713157	0.52127
VCPIP1	0.025131	0.502423	0.191949
RADIL	0.284348	-0.44175	0.587721
SPG11	0.204174	0.182861	0.497963
ELMO2	0.001332	0.467786	0.036119
TMX3	0.33388	0.16562	0.620902
DCAF5	0.406926	0.472771	0.680612
ZNF462	0.902287	0.0116	0.971583
CHAMP1	0.027821	0.742932	0.202072
L3MBTL3	0.8257	0.077897	0.930864
LCOR	0.109712	0.496193	0.377423
MYO15B	0.74145	0.074721	0.887483
ZFP91	0.552104	-0.01727	0.783041
DCHS1	0.19855	0.286709	0.49217
CLMN	0.077627	0.823735	0.324641
SERAC1	0.773543	-0.15856	0.903913

PDLIM2	0.400346	0.049565	0.67622
FAR2	0.092612	0.333618	0.347013
BTF3L4	0.026002	0.913358	0.193808
RNF170	0.965801	0.20114	0.99616
ZFYVE19	0.166342	0.896021	0.457139
SLC35E1	0.754042	0.039339	0.894351
TMEM87B	0.010997	0.965537	0.127049
ZNF668	0.683433	0.254097	0.85709
ZBTB45	0.883203	-0.00684	0.962618
USP47	0.218632	0.224348	0.515816
CLPTM1L	0.070198	0.449473	0.309184
PBK	0.299972	0.631983	0.602797
ARL5B	0.193885	0.778799	0.488275
DNAJC1	0.060574	0.552073	0.287134
SCYL1	0.367925	0.132462	0.650718
COX4I2	0.858289	0.078057	0.949081
ZNF512B	0.329491	0.562544	0.619341
FAM84B	0.340494	-0.85617	0.627258
CNDP1	0.830329	-0.22544	0.933615
PKNOX2	0.322009	0.113089	0.613139
EXOC2	0.466082	0.104984	0.721065
CNDP2	0.684992	0.092057	0.85709
PPP1R13B	0.357326	-0.46872	0.641472
EHMT2	0.010194	0.598083	0.121876
ZFR	0.04253	0.437926	0.243142
FAM210B	0.314759	0.880168	0.612778
FAM167A	0.186982	-0.36751	0.479162
B3GALT6	0.225978	0.649506	0.525131
NSD1	0.952829	-0.27132	0.993074
EP400	0.0662	0.865195	0.300455
SNX27	0.021897	0.301315	0.176756
KIF16B	0.442875	0.514921	0.704334
ALPK3	0.632969	0.247642	0.831194
FCRL2	0.161733	0.335529	0.453012
PRMT6	0.375073	0.413353	0.656867
IFT74	0.96549	0.032369	0.99616
SIGLEC10	0.867668	-0.13073	0.954126
TRIM47	0.135171	0.459342	0.417833
SENP8	0.212869	-0.80215	0.509228
CNOT6L	0.884406	-0.14155	0.963599
DHRS1	0.78936	0.125536	0.911483
UBXN10	0.26324	-0.32914	0.565873
DNAJC30	0.9027	0.045633	0.971583
UBE2E2	0.018778	1.809132	0.165951
SAMD8	0.810389	-0.14381	0.921889
C9orf72	0.313777	0.507352	0.612263
RNPC3	0.73481	0.26682	0.883541
C9orf89	0.333874	0.346873	0.620902
ZSCAN31	0.331051	-0.3846	0.619341
RMDN2	0.038957	-0.49109	0.236232
PRRC1	0.004071	0.510621	0.072359
TBATA	0.155961	0.748671	0.44616
CCDC114	0.533465	-0.16224	0.7709
CFAP53	0.722377	-0.14367	0.876654
KLHL15	0.021806	0.419941	0.176756
FGD4	0.074893	-0.79042	0.318717
PACRG	0.169846	-0.37802	0.461029
AK8	1	-0.02454	1

HIAT1	0.417598	-0.37105	0.688506
C12orf66	0.912516	0.214307	0.975706
FBXL18	0.267753	0.314994	0.570195
ZNF512	0.687409	0.451275	0.858864
COQ10A	0.175422	-0.33632	0.466796
NSMCE2	0.033781	1.467415	0.221141
JSRP1	0.41554	0.525153	0.686235
NDNL2	0.508197	0.390929	0.752425
PCMTD1	0.655653	0.446245	0.84209
HEXIM2	0.461622	0.266297	0.718747
TMEM68	0.581701	0.176476	0.797998
PPM1M	0.550476	0.161253	0.781327
FAM20A	0.063249	1.078941	0.294055
HSPA12B	0.496874	-0.62501	0.743802
HS6ST2	0.942152	0.008737	0.988404
KCTD7	0.894479	-0.01088	0.968626
CFAP57	0.213217	-0.42449	0.509228
RNF145	0.516723	-0.22864	0.758769
CEP63	0.285717	0.243536	0.590053
YTHDC1	0.960544	-0.04824	0.994122
KREMEN1	0.696781	-0.04002	0.863213
TMEM56	0.659408	0.237977	0.844416
CCDC43	0.023327	1.301004	0.184409
COG8	0.121914	0.546477	0.397343
ZNF48	0.045973	0.497996	0.252361
WDR92	0.011635	0.734291	0.129699
NOL4L	0.402321	0.492989	0.678038
VSTM2L	0.074408	1.636601	0.317894
C7orf26	0.020759	1.538718	0.172048
ENTHD2	0.771489	0.121187	0.903336
TTC14	0.044336	0.771094	0.24851
PWWP2A	0.101385	0.69963	0.362378
MBOAT7	0.923804	0.009726	0.980751
DOCK7	0.000759	0.69257	0.025904
UROC1	0.319672	-0.21319	0.613139
SPATA13	0.1865	0.640886	0.479026
RILP	0.219436	-0.75541	0.517271
TSNARE1	0.43065	0.105733	0.695651
FOPNL	0.065617	0.677581	0.299507
SFXN2	0.143691	-0.99517	0.431666
ZNF830	0.007612	2.018762	0.10525
ZMAT2	0.249041	-0.04607	0.552533
FAM210A	0.421637	0.405177	0.691467
FRMD6	1.80E-05	2.568039	0.001573
TTC25	0.79259	-0.10883	0.913596
N/A	0.331588	-0.16276	0.619341
SCLT1	0.234942	-0.20608	0.537046
C8orf37	0.739382	0.216186	0.886294
CCDC115	0.876774	0.376481	0.958859
NAP1L5	0.52506	0.319839	0.764479
SLC46A1	0.641107	0.46278	0.835764
ANKRD27	0.039553	0.837132	0.237431
CLIC6	0.382127	-0.59916	0.661698
PVRL4	0.728303	0.19999	0.879656
PRAP1	0.670768	-0.1185	0.850681
BIRC8	0.244375	-0.4689	0.547425
NSUN5	0.376165	0.603114	0.657741
RPRD1A	0.238962	0.591124	0.541047

FCRL3	0.26299	-1.46004	0.565873
COL21A1	0.485519	0.169868	0.736044
AGAP3	0.320823	0.622663	0.613139
ARAP1	0.020781	0.330555	0.172048
ACAP3	0.904949	-0.2184	0.972966
WDFY2	0.014512	1.459768	0.147184
SERPINB12	0.09909	0.517186	0.358568
IPO9	0.006775	0.644455	0.098484
CASD1	0.935162	-0.04542	0.984984
AP1S3	0.543819	0.653839	0.777449
MIA2	0.247021	0.293999	0.549922
DCBLD2	0.018531	1.164757	0.165084
PGLYRP2	0.317645	0.263479	0.613139
GPR124	0.15336	0.511787	0.443292
ARHGEF17	0.051278	0.475858	0.265139
INPP4A	0.089413	0.537568	0.341477
MCEE	0.171095	-0.80136	0.461822
RBM14	0.216173	0.222245	0.512741
SCGB3A2	0.245732	-1.25792	0.549743
ERMAP	0.845899	-0.02118	0.942239
RCHY1	0.10622	-0.98975	0.370932
ZNF385A	0.26368	0.349419	0.565913
GBP5	0.939498	0.00511	0.986498
GBP4	0.801372	-0.07107	0.91752
SORCS2	0.372466	0.22118	0.655575
SIGLEC12	0.967597	-0.16491	0.997177
KLHL5	0.004311	0.727312	0.075057
UHRF2	0.313375	-0.04715	0.611585
NEDD4L	0.651389	-0.08251	0.839222
QKI	0.019032	0.289941	0.166597
PNMA5	0.116303	0.604129	0.389223
LENG8	0.186018	0.293482	0.478129
CCDC85A	0.15526	0.254045	0.444958
PLEKHG4B	0.603077	0.266183	0.812922
FMNL2	0.330591	0.338128	0.619341
NEK1	0.138594	0.542547	0.424105
PUS7	0.78934	0.210254	0.911483
FAM111A	0.1263	0.850097	0.403572
TRAPPC9	0.623137	0.07565	0.825978
PLIN4	0.954544	-0.15249	0.993074
BTBD9	0.242992	0.674785	0.546608
TRNT1	0.051278	0.212178	0.265139
SMG1	0.103963	0.754761	0.366863
ALS2	0.161065	0.422348	0.453012
TMEM237	0.188151	0.462122	0.480067
DERL3	0.13821	1.072152	0.42406
ALKBH3	0.792231	-0.09777	0.913596
GSDMA	0.575621	0.142852	0.796634
DLC1	0.552521	0.277384	0.783393
PPP1R10	0.04253	0.632291	0.243142
SLC38A2	0.371397	0.639658	0.65423
FYTTD1	0.142921	0.388666	0.430602
SLC2A13	0.690274	-0.12337	0.859723
TEFM	0.167194	0.917725	0.457187
RAB3IP	0.468807	-0.43927	0.723565
MTMR9	0.009626	1.036489	0.118893
PRAM1	0.343863	0.352807	0.630156
VPS35	0.992106	-0.02156	1

SMIM14	0.96601	0.06778	0.996282
ALPK1	0.219547	-0.46762	0.517425
SCGB3A1	0.213895	-1.49346	0.509407
PURB	0.568296	0.225619	0.791838
TRPM7	0.671991	0.100138	0.851286
PHF12	0.225738	0.64162	0.525131
XPO6	0.261801	0.815064	0.56535
HHIP	0.541937	-0.1812	0.775521
MAGI1	0.368403	-0.33953	0.651252
RBP7	0.993518	-0.08215	1
SPAG5	0.409632	0.399076	0.682657
PANX1	0.07804	0.980762	0.325635
FCRL5	0.816718	-0.27074	0.926577
NACC1	0.470084	-0.09081	0.72456
SNX18	0.039276	0.402465	0.236232
PASK	0.5823	0.265426	0.798306
TNFRSF13C	0.563678	-0.15308	0.789479
CIC	0.003486	1.470298	0.067901
BBS4	0.369312	0.357714	0.652548
UIMC1	0.223664	0.517384	0.521803
VPS13A	0.947409	-0.01633	0.990063
MED15	0.015038	1.177769	0.150049
GAL3ST4	0.320105	1.009323	0.613139
GFM1	0.255193	0.085541	0.557593
ERGIC2	0.033952	0.331553	0.221141
MCCC1	0.208914	-0.24315	0.503054
IL4I1	0.077655	0.454418	0.324641
CAMKK2	0.661309	0.245488	0.844736
TGS1	0.198885	0.565554	0.49217
NUDCD1	0.528742	0.068581	0.767576
ERBB2IP	0.378546	-0.14681	0.659304
TUBGCP6	0.011382	1.499892	0.128971
TUBGCP5	0.085294	1.005441	0.335537
USP28	0.159044	0.924061	0.449897
FNBP1	0.213733	0.27117	0.509228
TRIB1	0.540968	0.205369	0.775521
HMCN1	0.260731	-0.34293	0.56373
IFT140	0.143106	0.596297	0.430717
LMF1	0.595383	0.321416	0.806337
WDR24	0.729504	-0.21559	0.880023
JMJD8	0.21436	0.619841	0.510276
C16orf13	0.746536	0.006192	0.889644
RAB40C	0.689054	-0.1968	0.859429
RPS6KC1	0.019823	0.875932	0.168605
TP53RK	0.194929	0.226745	0.488911
PIGS	0.007333	0.466364	0.102825
WRNIP1	0.172129	0.379543	0.463481
RANBP9	0.004805	0.541923	0.081112
CLCC1	0.442223	0.203918	0.703704
UBL7	0.001623	2.376613	0.041669
HAPLN3	0.01237	1.837253	0.135707
LYSMD1	0.310183	0.234295	0.609864
CCNL2	0.147228	0.677091	0.436386
PEBP4	0.698835	-0.25524	0.864146
MYADM	0.000236	0.858693	0.011882
PLEKHF1	0.462056	0.063484	0.718747
SERINC2	0.455572	0.268239	0.71417
PPP1R9B	0.01039	0.440237	0.123374

SRPK1	0.577274	0.131012	0.796634
SMC6	0.478207	0.003617	0.730754
KCTD15	0.361932	0.539046	0.645575
STRBP	0.934288	0.29919	0.984157
TSPAN18	0.146521	-0.8395	0.435629
TMEM209	0.378546	0.038337	0.659304
DIRC2	0.013129	1.442604	0.140278
GPX7	2.44E-06	2.182362	0.000298
CPXM1	3.28E-07	3.734028	6.50E-05
ORAI2	0.175422	-0.40699	0.466796
CDK5RAP2	0.34596	0.537003	0.632606
CYP2S1	0.835394	0.38273	0.935999
IWS1	0.440254	0.419564	0.70314
SIN3A	0.072266	0.279518	0.313701
CEP89	0.301599	0.598173	0.603275
OSBPL9	0.981581	-0.05798	1
CRBN	0.129527	0.833852	0.410218
VWA9	0.068176	0.78536	0.303928
ADO	0.595344	0.094816	0.806337
CDK5RAP1	0.155375	0.648573	0.445058
MAP7D2	0.175307	0.569646	0.466796
SECISBP2	0.346277	0.233963	0.632606
RSF1	0.442223	0.237354	0.703704
RBM15	0.101186	0.550739	0.361901
PPP1R16B	0.452579	0.236134	0.712397
RUFY1	0.450097	0.093849	0.709598
IMMP2L	0.087124	1.020718	0.3381
KCNK17	0.079293	0.306167	0.32578
SPEN	0.05285	0.697406	0.269122
PNKP	0.327323	0.102012	0.617719
NMNAT3	0.031342	-1.36407	0.212999
MMS19	0.025563	0.487379	0.193268
SLC9A7	0.68741	-0.30204	0.858864
UHRF1	0.025741	1.683369	0.193808
FAM129B	0.887225	-0.02685	0.96416
YME1L1	0.034511	0.412692	0.222148
RMDN3	0.960544	0.080774	0.994122
TCF12	0.33682	0.445559	0.624371
MUC4	1	-0.14513	1
MYCBP	0.627809	0.133831	0.828932
CYTH2	0.229894	0.687534	0.529875
ACOX2	0.591789	-0.36299	0.805934
TBCB	0.498977	0.183857	0.744839
PSMB7	0.744041	0.046355	0.888303
CNN2	5.77E-06	0.796324	0.000627
SEC62	0.277896	0.525123	0.579677
PCYT2	0.185996	-0.19047	0.478129
PHOX2B;DRGX;OTP;RAX	0.609946	-0.70251	0.817215
CDC5L	0.094502	0.254805	0.349959
PSMD1	0.197211	0.160848	0.490309
NOTCH4	0.942624	0.054032	0.988547
CD180	0.041842	0.866509	0.242066
SDF2	0.006642	0.446089	0.097053
PFDN5	0.364426	0.084497	0.646996
PAFAH2	0.004996	-1.2954	0.082994
DDO	0.369906	-0.76756	0.653181
AGAP2	0.470337	-0.39583	0.724717
RNF2	0.02184	1.258017	0.176756

PARK7	0.354055	-0.20872	0.638094
GAS2L1	0.018687	1.346325	0.165633
EYA1	1	-0.01098	1
EYA3	0.016985	1.440151	0.157641
FMO2	1	-0.06711	1
NEU1	0.015628	1.134635	0.152856
SORT1	0.897655	0.005507	0.969793
VAT1	0.563832	0.039847	0.789479
LGMN	0.257958	0.282777	0.560462
PLIN2	0.020065	0.803572	0.169555
MMP19	0.00706	2.128082	0.10064
DNAJC2	0.656099	-0.47959	0.84209
MPHOSPH6	0.983801	-0.34353	1
MPHOSPH8	0.01589	0.98753	0.153751
NUP88	0.249724	0.155638	0.552833
PKP4	0.051201	-0.74695	0.265139
PIK3R4	0.197211	0.251126	0.490309
P2RX4	0.950035	-0.00046	0.990863
P2RX7	0.60065	-0.15592	0.811031
SERPINI1	0.898515	-0.02015	0.970438
POP1	0.42284	0.096052	0.691467
TSC22D3	0.94561	0.11512	0.988945
MNT	0.029335	1.101626	0.207066
S100A13	0.181644	-0.17388	0.472575
SCAF11	0.084071	0.345974	0.334685
TBX5	0.300882	0.165167	0.602797
TEAD3	0.262528	0.735355	0.565873
TIMM17A	0.253422	1.022442	0.557407
TSNAX	0.559385	0.100786	0.78633
NDN	0.571349	0.081059	0.793891
SEPHS2	0.699584	-0.06281	0.864146
EIF3C;EIF3CL	0.252448	0.061409	0.555643
TTC1	0.442223	0.170205	0.703704
DNAJC7	0.025131	0.344004	0.191949
CDCA3	0.011513	0.803356	0.129242
C12orf57	0.892438	0.319724	0.966699
PHB2	0.426712	-0.10529	0.693599
SLC38A3	0.157319	-0.55141	0.447277
COPS8	0.211314	0.183938	0.506632
PRPF18	0.063232	1.115324	0.294055
RAD9A	0.054495	1.186529	0.273313
PKMYT1	0.496019	0.238982	0.743024
SDHC	0.12513	0.253306	0.401334
OSMR	0.097679	0.847391	0.356252
CHP1	0.830256	0.083453	0.933615
KIF2C	0.01246	1.287428	0.136352
CGREF1	0.03382	1.28267	0.221141
ZNF184	0.482162	0.122784	0.733586
MAP3K5	0.089397	0.614727	0.341477
MGLL	0.177368	-0.33762	0.468602
LYST	0.771029	-0.07153	0.903336
ATXN2	0.020062	0.84642	0.169555
DOK1	0.456995	0.302209	0.715224
MTR	0.311224	0.380314	0.609894
RBBP8	0.916225	0.091552	0.977854
KCNJ15	0.333105	-0.62568	0.620722
HSD17B10	0.324076	0.150304	0.614309
COL12A1	2.18E-08	2.119309	7.34E-06

SMAD5	0.103849	0.938437	0.366863
SEPTIN5	2.25E-05	1.397507	0.001867
SIGMAR1	0.13509	1.152391	0.417833
BARD1	0.768139	0.037632	0.901915
HNRNPAB	0.223612	0.240211	0.521792
CCL19	0.337382	-0.77816	0.624587
LITAF	0.968571	0.057783	0.997592
NAP1L4	0.142204	0.189052	0.428816
MGST2	0.796968	0.000255	0.915123
NAPG	0.590866	0.065993	0.804875
PIP5K1A	0.052831	1.180165	0.269122
TXN2	0.830256	0.111651	0.933615
ABCA3	0.027011	-2.29984	0.198793
MAP3K3	0.019431	0.743003	0.167517
ATP5S	0.929042	0.533715	0.983115
APBA2	0.011513	0.717982	0.129242
CMKLR1	0.755165	0.203546	0.895527
GPA33	0.447034	0.30694	0.707261
MIPEP	0.861239	-0.04912	0.950165
ACO2	0.986843	-0.00754	1
TM9SF2	0.023466	0.320982	0.184845
COQ7	0.00746	-0.96407	0.10395
SLC29A1	0.762625	-0.45086	0.899242
EPAS1	0.141811	0.586952	0.428816
TSG101	0.101186	0.206695	0.361901
CIB1	0.090665	-0.4219	0.343901
CPNE1	0.006131	0.413755	0.092018
CCT7	0.286754	-0.07114	0.590053
MYD88	0.411524	0.120682	0.683152
EBNA1BP2	0.559385	0.316591	0.78633
ARID3A	0.369966	0.443446	0.653183
HAUS7	0.021138	1.176123	0.173882
PRMT1	0.012773	0.354036	0.137944
BAG1	0.432447	-0.15662	0.697404
ATF6B	0.290886	0.651626	0.595133
AGPAT1	0.2958	0.409872	0.598344
EGFL8	0.331588	-0.13812	0.619341
PTPN18	0.904941	-0.06411	0.972966
DUSP9	0.556057	0.131534	0.783439
FOXC2	0.736368	-0.13359	0.884985
PKP2	0.798178	0.231436	0.916038
SH3GL1	0.021897	0.30648	0.176756
SH3GL2	0.571349	-0.05697	0.793891
RARRES2	0.008409	0.306142	0.110127
MYOC	0.163438	-0.23163	0.453012
TEP1	0.861239	-0.1338	0.950165
OMD	1.18E-06	4.466277	0.000169
SEMA3C	0.003304	1.94381	0.065524
VRK1	0.020419	0.84075	0.170514
GDF15	0.946039	-0.06879	0.989207
AKAP9	0.950035	0.07518	0.990863
DPYSL5	0.844559	0.261136	0.942239
B9D2	0.859348	0.083346	0.949684
P2RY13	0.748884	-0.17238	0.890902
NIPSNAP1	0.057044	0.298964	0.278658
DHRS9	0.829237	0.208948	0.933615
HSD17B14	0.183878	-0.63587	0.474829
NCAPG	0.881172	-0.08508	0.961515

ARPC5L	0.04896	0.326085	0.259694
MICU1	0.515876	-0.03699	0.757825
C7orf25	0.032372	1.272179	0.216159
FAM118B	0.178132	1.090584	0.469506
HOPX	0.283779	0.068686	0.586874
SPIN2B	0.481732	0.172596	0.733586
PAIP2	0.093007	1.228516	0.347256
MAPKAP1	0.384663	0.661144	0.66413
RBM4B	0.453828	0.702697	0.712914
KCTD14	0.249903	-0.95649	0.553119
NABP2	0.612202	-0.42027	0.818853
ZFYVE21	0.031483	1.644896	0.213823
DDX50	0.118765	0.31661	0.392409
MRPL34	0.971687	0.053195	0.997592
SMIM7	0.339004	0.267825	0.626409
PDCD1LG2	0.902917	0.101293	0.971583
ELAC2	0.003368	0.462484	0.066236
C19orf43	0.673609	1.108227	0.852854
GRWD1	0.024286	0.525642	0.188496
MACROD1	0.897655	-0.12641	0.969793
TCF25	0.020774	0.990766	0.172048
CMSS1	0.688865	-0.36596	0.859429
TBL1Y	0.452579	0.198326	0.712397
FAM110A	0.49063	0.127671	0.738656
ECSIT	0.183798	0.349043	0.474829
WDR77	0.164991	0.164741	0.454525
C17orf62	0.151386	0.214439	0.44125
SOST	0.393617	0.371319	0.671013
VKORC1	0.159041	1.184034	0.449897
DPH2	0.161599	0.570566	0.453012
MRPL57	0.063784	1.574818	0.295622
KXD1	0.637367	0.149499	0.833511
FAM173A	0.971656	0.022809	0.997592
TUBA1C	0.992106	0.002176	1
VIMP	0.874203	-0.45783	0.957716
APOL2	0.016499	0.652219	0.155558
BCL7B	0.593889	-0.30174	0.806337
SENP7	0.010684	1.145501	0.125661
MYBBP1A	0.049723	0.41424	0.261364
NUDT12	0.721208	-0.05931	0.876242
AIF1L	0.001255	-2.06109	0.03481
NRIP2	0.879869	-0.09029	0.961441
TMEM47	0.659619	-0.33967	0.844589
LPIN3	0.939698	0.034526	0.986525
FERMT1	0.565021	0.414986	0.790883
MGME1	0.068855	1.255397	0.30645
GORASP1	0.666962	0.328441	0.848454
SYT15	0.020356	-1.01296	0.170514
HEPH	1.63E-08	2.812589	6.25E-06
FYCO1	0.450097	-0.07138	0.709598
SLC25A21	0.062424	1.360505	0.292127
CLSTN3	0.217477	0.573207	0.514844
ZSWIM1	0.167109	0.304092	0.457139
JPH2	0.658522	0.136344	0.843575
ACBD6	0.009408	1.70483	0.11751
CORO1B	0.343873	0.047212	0.630156
CCDC77	0.095031	0.846536	0.35136
NAA38	0.45935	0.456802	0.716923

TXNDC17	0.641914	-0.12827	0.835764
PIGQ	0.513141	0.25438	0.75581
BUD13	0.475807	0.575538	0.728899
CPPED1	0.533067	0.177558	0.7709
VPS25	0.378546	0.301746	0.659304
SH2D3A	0.400605	-0.39034	0.676417
MRPL45	0.675323	-0.31338	0.853175
C7orf50	0.239021	-0.94228	0.541047
NUDT16L1	0.764084	-0.04537	0.899242
MXRA8	0.002834	1.891069	0.060074
LZTS2	0.851252	-0.16321	0.945258
SDF4	0.011636	0.556583	0.129699
SRSF8	0.224762	-0.66671	0.523815
TM2D3	0.019587	1.718705	0.1684
PDCD2L	0.425473	0.451786	0.693599
PAAF1	0.033636	1.553814	0.220876
WIBG	0.825116	0.021738	0.930395
PYGO2	0.116368	1.11753	0.389223
CHCHD6	0.367923	0.12676	0.650718
AIFM2	0.576975	1.037216	0.796634
ADPGK	0.041202	0.333901	0.240826
GPATCH1	0.002086	1.758079	0.048476
ARHGAP9	0.034149	0.554742	0.221721
RIOK1	0.439669	0.905093	0.70314
UQCC2	0.289748	-0.23275	0.593134
MIEN1	0.986841	0.144533	1
LLPH	0.151842	1.405666	0.442015
CBWD1;CBWD5;CBWD3;CBWD6	0.14131	0.756015	0.427981
GINS4	0.784838	0.132776	0.909861
UTP23	0.497382	-0.20968	0.744361
SLC50A1	0.188058	0.765348	0.480067
SIKE1	0.960323	0.142524	0.994122
PELO	0.018368	0.88836	0.163766
GINS3	0.818809	0.16265	0.92687
FAM213A	0.764084	0.019798	0.899242
WDR83	0.028406	1.448536	0.20366
SLC39A3	0.933487	0.063339	0.984157
TRIM56	0.046008	0.266955	0.252361
CENPK	0.047453	0.913708	0.255923
ANAPC13	0.396112	0.452816	0.67382
ERP44	0.78936	0.05848	0.911483
LXN	0.20535	0.25707	0.49957
NIPSNAP3B	0.677236	0.066123	0.854325
TTYH2	0.488557	-0.33895	0.738428
TMEM175	0.860114	0.192124	0.950165
ATG101	0.430785	0.57514	0.69569
NOL10	0.050102	0.767299	0.26298
NTPCR	0.206529	0.062966	0.500256
AGMAT	0.154635	0.634422	0.444958
C2orf88	0.334121	0.497063	0.621039
C19orf52	0.382127	0.140093	0.661698
RTBDN	0.331588	-0.16577	0.619341
TACO1	0.54177	-0.19362	0.775521
HDHD3	0.67051	-0.09139	0.850681
TINF2	0.479044	0.350729	0.731627
TUBGCP2	0.001956	0.528513	0.04685
C17orf80	0.096685	0.996269	0.354489
ESYT1	0.11568	0.180367	0.387731

UBAC1	0.934273	0.421069	0.984157
TMEM204	0.251789	0.574128	0.555643
CCM2	0.002543	0.961876	0.055387
YIPF4	0.656099	0.323242	0.84209
RTKN	0.226028	0.795343	0.525131
C16orf70	0.002401	1.502524	0.053253
NAA11	0.988991	0.014197	1
TSEN34	0.528478	-0.26252	0.767576
CRACR2A	0.518774	0.476218	0.760272
SYT17	0.081481	-0.75017	0.329697
CHCHD5	0.011508	-1.59975	0.129242
DESI2	0.013631	1.839276	0.14262
CNPY3	0.660922	0.049671	0.844596
MTG1	0.500098	-0.67832	0.74591
ALG1	0.360948	0.119224	0.644411
LIMD2	0.22839	0.99695	0.528484
HAUS8	0.002587	1.912175	0.056149
ALKBH7	0.115663	0.999416	0.387731
INPP5K	0.986843	-0.23075	1
POLR3GL	0.884736	0.13485	0.963721
NDFIP1	0.391035	0.838964	0.669698
PSMG3	0.98684	0.01158	1
UPK3B	0.57907	-0.17815	0.796675
COPS4	0.364426	0.111978	0.646996
TCHP	0.080213	0.98378	0.326714
WAC	0.000821	2.121242	0.027117
DIDO1	0.089414	0.449777	0.341477
MTA3	0.15272	0.637549	0.443165
RBM42	0.154226	0.383046	0.444958
DCTN5	0.517819	-0.45407	0.760014
MCMBP	0.025131	0.448871	0.191949
AARSD1	0.045294	0.669793	0.250872
DCUN1D5	0.439212	0.4815	0.702726
PAGR1	0.431223	0.521253	0.696172
FAM103A1	0.035654	1.180014	0.225452
URM1	0.009344	0.502391	0.117105
ANP32E	0.017726	0.557362	0.161012
MED10	0.000734	1.717825	0.02568
LRRC1	0.407773	0.059127	0.680612
PI4K2A	0.130075	0.328352	0.410899
TMEM43	0.029749	0.249227	0.208169
FSD1	0.157319	0.56099	0.447277
DPH7	0.095807	0.400424	0.352331
TBCD	0.135171	0.326541	0.417833
NDC1	0.936911	0.054542	0.986451
TMEM208	0.031613	1.565043	0.214187
TTPAL	0.799685	0.093412	0.916176
FUCA2	0.051666	0.719696	0.266309
HGH1	0.48231	0.087791	0.733586
DHRS4	0.039276	0.028726	0.236232
THTPA	0.173032	1.161482	0.464476
LMF2	0.048208	0.335877	0.257798
CHRDL1	0.507724	-0.6053	0.75191
NDUFAF3	0.724155	0.158209	0.876654
CENPO	0.15526	0.221181	0.444958
PRR15L	0.902553	-0.04059	0.971583
MMTAG2	0.42777	0.034447	0.69482
TMEM243	0.560494	-0.55576	0.787589

DOHH	0.01724	1.878947	0.158939
ADAT1	0.283087	-0.18427	0.586424
MKNK1	0.803829	-0.13345	0.918848
TMEM70	0.646626	0.271028	0.837353
SPON2	0.060265	1.55128	0.287134
MED18	0.074451	1.08898	0.317957
ISCA1	0.862812	0.175889	0.951367
TUBB6	1.28E-05	0.839569	0.001165
CRB3	0.308969	0.694236	0.609864
C9orf142	0.799534	-0.0399	0.916173
POLR3C	0.029219	0.713448	0.20681
ABHD14A	0.361932	0.489187	0.645575
HNRNPUL1	0.138653	0.211665	0.424105
CHCHD7	0.067155	-1.3393	0.302058
MSTO1	0.500784	0.62465	0.746432
PHF23	0.806572	0.124657	0.920528
PDCD10	0.32085	0.072235	0.613139
RPP25	0.976495	0.034628	0.998921
G6PC3	0.24062	1.031463	0.543858
DERL1	0.314461	0.398213	0.612305
EFHD1	0.00584	1.968819	0.089662
HTATIP2	0.167008	-0.28021	0.457139
DDX23	0.211314	0.167185	0.506632
WRAP53	0.000805	2.169951	0.026988
APOO	0.981581	-0.0045	1
BDH2	0.850885	-0.11686	0.944945
FAM195A	0.749046	0.141331	0.890902
C20orf24	0.84819	0.139972	0.944084
C9orf16	0.792343	0.194475	0.913596
TRAF4	0.740246	0.187168	0.886428
ALG12	0.632383	0.26346	0.830965
C1orf50	0.480311	0.424537	0.732554
MRI1	0.371444	0.127146	0.65423
ABHD6	0.533738	-0.0901	0.771094
C15orf57	0.296204	-0.6937	0.598943
SLC25A23	0.920385	0.127565	0.980269
MLPH	0.004012	-1.18116	0.071889
WDR18	0.228671	0.213003	0.528484
VAMP8	0.44615	-0.04614	0.706795
THUMPD3	0.015062	0.977658	0.150049
ADI1	0.955288	-0.32955	0.993074
RNF126	0.056538	1.289402	0.277829
CEP250	0.42656	0.202915	0.693599
MECR	0.14042	-0.43811	0.426568
EMC6	0.99464	-0.09063	1
NTMT1	0.986843	-0.13216	1
EDEM2	0.431347	0.118478	0.69627
KATNB1	0.658802	0.475756	0.843836
TUBB2B	0.000483	2.454307	0.020165
FICD	0.991073	0.020957	1
DSCC1	0.034185	1.024197	0.221721
MLST8	0.835403	-0.10491	0.935999
C2orf49	0.553712	0.303271	0.783439
TMEM109	0.130075	-0.09689	0.410899
PBDC1	0.175259	0.214599	0.466796
KIFC3	0.064378	0.720693	0.296449
PTDSS2	0.088198	1.160077	0.339301
ST6GALNAC5	0.163707	0.67103	0.453012

NOC4L	0.171095	0.600533	0.461822
UTP14A	0.051672	0.610713	0.266309
DUSP23	0.577274	0.206394	0.796634
ALG8	0.208556	0.851191	0.503054
TMED9	0.000515	0.369186	0.020674
TMEM147	0.069533	1.185225	0.307494
NUPL1	0.010194	0.395435	0.121876
SELO	0.799524	0.298689	0.916173
DPCD	0.758323	0.213023	0.896847
GGACT	0.502215	0.391711	0.747559
RUSC1	0.792499	0.111742	0.913596
GNL3	0.004244	0.694285	0.074174
SPATA5L1	0.036208	0.789741	0.227448
RIOK2	0.685656	0.1979	0.857727
TRMT61B	0.236003	0.130634	0.537858
TMUB1	0.908073	0.373781	0.9734
KIAA0586	0.134221	-0.58403	0.417223
TIMM21	0.022367	2.043925	0.179894
TMEM106C	0.300882	0.318601	0.602797
SARG	0.013497	-1.53572	0.141812
KIFC1	0.006623	1.66265	0.097053
NUP85	0.031269	0.309273	0.212999
TPPP3	0.027821	-0.72967	0.202072
ELOVL1	0.644201	-0.07196	0.837353
DDA1	0.743478	-0.23498	0.888303
KATNAL1	0.923152	0.10417	0.980751
CINP	0.211255	0.880261	0.506632
HIRIP3	0.356692	0.546967	0.641472
HIGD2A	0.167483	0.433691	0.457358
IFT27	0.239029	0.55139	0.541047
CCDC94	0.022171	1.682255	0.178703
NUDT9	0.438315	0.169363	0.701933
TARS2	0.257958	0.253158	0.560462
ACAT2	0.434427	0.209202	0.698194
REPIN1	0.120284	-0.81864	0.39497
RBM4	0.052873	0.360806	0.269122
FUNDC2	0.357486	-0.14125	0.641472
RPAP1	0.007224	1.229829	0.101678
SF3B5	0.023065	0.356113	0.182986
C1orf43	0.135012	0.832653	0.417833
SFXN3	0.004071	0.431245	0.072359
PRR14	0.114594	1.191554	0.386544
COLEC11	0.045488	-0.54636	0.250872
YIPF2	0.604483	-0.29398	0.813002
CHID1	0.637197	-0.03628	0.833511
PAPOLG	0.064261	0.731361	0.296208
CARD10	0.839125	0.01985	0.939212
SLC4A1AP	0.944783	-0.03743	0.988547
CDK19	0.944069	0.008007	0.988547
APOL6	0.254941	-1.11846	0.557593
GTPBP2	0.183025	0.828038	0.474175
LSM14B	0.014224	1.843268	0.145268
TAPBPL	0.637197	-0.2189	0.833511
SORBS1	0.454064	0.194031	0.712914
JAM3	0.03553	1.317992	0.225178
HINT2	0.407778	-0.0915	0.680612
CARD6	0.953004	-0.06593	0.993074
BTBD2	0.737798	0.211841	0.886077

TM2D2	0.05085	1.966449	0.264901
TM2D1	0.563678	-0.15712	0.789479
STRA6	0.282912	0.717097	0.586171
PLA2G12B	0.102462	-0.46695	0.364372
SGPP1	0.455535	0.410076	0.71417
PLVAP	0.085745	-0.69121	0.335537
LGR4	0.568515	0.238479	0.791838
OSBPL11	0.048205	0.645613	0.257798
OSBPL10	0.001063	1.854631	0.031596
BBS2	0.91143	-0.03082	0.975706
NPL	0.158067	0.721964	0.448712
RAB11FIP5	0.001913	0.869016	0.046318
TEKT3	0.007122	-0.94823	0.101139
BBC3	0.336166	0.563688	0.623617
NT5C1A	0.301362	-0.58486	0.603275
TBC1D10A	0.138415	0.871929	0.424105
C1QTNF6	0.124128	0.687473	0.400153
C1QTNF5	0.00563	2.180844	0.088608
C1QTNF7	0.02013	-1.533	0.169778
C1QTNF3	0.001636	2.892176	0.041715
C1QTNF2	0.018767	-1.09854	0.165951
TMEM120A	0.257958	-0.17157	0.560462
NAA15	0.609213	0.041893	0.816529
KLF16	0.147416	0.904924	0.436386
BCL2L13	0.142204	0.326704	0.428816
CARD14	0.487154	-0.42705	0.737011
CARD11	0.064135	0.921485	0.296208
PRX	0.654188	-0.65927	0.84199
FSD1L	0.382877	0.491009	0.662478
ASPN	0.071226	0.569329	0.311134
CLEC7A	0.805837	-0.0207	0.919879
SLC12A9	0.272095	0.150513	0.575039
SRRT	0.955289	0.05051	0.993074
QTRT1	0.148574	0.481253	0.438051
CFHR5	0.123287	0.831217	0.399387
TMEM59	0.495705	-0.52883	0.743024
AP1M1	0.017726	0.246201	0.161012
NUSAP1	0.913093	0.109457	0.975706
SLC26A6	0.160289	0.682034	0.452818
FTHL17	0.969086	-0.07547	0.997592
C14orf142	0.301923	0.324377	0.603275
OSBPL1A	0.4576	-0.05514	0.715224
CECR5	0.939534	-0.11261	0.986498
FANCD2	0.877757	-0.01458	0.959795
EMILIN2	0.374984	0.131208	0.656862
MAK16	0.407191	-0.06566	0.680612
SLC46A2	0.992139	0.012835	1
PACSIN1	0.237385	1.225213	0.539736
SCAPER	0.561277	-0.38891	0.788589
EMR3	0.614841	0.038749	0.820298
ITPA	0.660922	-0.02558	0.844596
HDAC8	0.471304	0.565873	0.725803
RTFDC1	0.19466	0.959585	0.488911
CHMP4A	0.965801	0.006661	0.99616
EIF2A	0.149515	0.393712	0.438811
PECR	0.718727	-0.60637	0.874478
SEC11C	0.434427	0.185581	0.698194
CADM1	0.347172	0.04462	0.632606

ANGPTL4	0.023794	1.820091	0.186463
POLDIP3	0.699592	0.131336	0.864146
KIAA1671	0.855362	0.261936	0.946993
SHANK3	0.994311	0.038244	1
GNB1L	0.115843	1.089925	0.388045
FUT8	0.716764	0.253495	0.873394
MRPL32	0.433059	0.594351	0.698118
MRPL20	0.099299	0.588307	0.358854
MRPL13	0.274985	0.103314	0.577625
MRPL9	0.541765	0.086913	0.775521
MRPL4	0.327323	0.233071	0.617719
CNFN	0.322009	0.221223	0.613139
MRPL1	0.478274	0.037314	0.730754
PCGF6	0.087786	0.828031	0.339301
CDHR2	0.107007	0.547707	0.371691
ACE2	0.258196	-0.38129	0.560621
B3GNT5	0.284348	-0.64195	0.587721
NIFK	0.734024	0.137879	0.882858
PARD6B	0.016491	-1.49347	0.155558
FAM126A	0.004586	1.390189	0.078257
FGFBP2	0.041397	0.583492	0.240826
YTHDF1	0.380774	0.471952	0.661698
HELZ2	0.507292	0.21209	0.75153
RBCK1	0.01019	1.055165	0.121876
SRXN1	0.428066	-0.42201	0.695122
MRPS26	0.876815	0.207383	0.958859
KRTAP2-2;KRTAP2-1;KRTAP2-4;KRTAP2-3		0	
NLN	0.44615	0.258409	0.706795
AGXT2	0.331588	-0.11299	0.619341
CEP41	0.47017	0.522599	0.72456
SETD2	0.008812	1.308705	0.11325
TBC1D2	0.404052	0.198433	0.678271
IFIH1	0.102992	0.793907	0.365649
PANK2	0.885157	0.13982	0.964084
DOCK9	0.809743	0.030208	0.921586
FRMD8	0.061485	0.357024	0.289565
PITPNM3	0.079238	-0.63577	0.32578
PITPNM2	0.278824	0.205826	0.581068
WHSC1L1	0.162378	0.77481	0.453012
ABCA2	0.689884	-0.29549	0.859429
SLC29A3	0.001255	1.534509	0.03481
NUF2	0.002096	1.590422	0.048534
MRPL37	0.466082	0.097578	0.721065
GTPBP4	0.419078	0.143201	0.688796
ASPSCR1	0.255193	0.182966	0.557593
OSBPL8	0.003514	0.473633	0.067901
OSBPL7	0.471593	0.292483	0.726047
OSBPL6	0.318143	0.31393	0.613139
UACA	0.255193	0.353533	0.557593
RAB34	0.010997	0.735681	0.127049
DPH1	0.274747	0.585724	0.577625
WDR11	0.153276	0.372002	0.443165
IRX2	0.207366	-0.64916	0.501941
UPF3B	0.803697	-0.26579	0.918848
CRNKL1	0.486499	0.122023	0.73612
NACAP1	0.635921	0.240979	0.833511
TBL1XR1	0.211314	0.194893	0.506632
UBL5	0.609158	-0.03791	0.816529

PPP1R12C	0.025563	0.465185	0.193268
PRKD2	0.131757	0.280593	0.413476
PLA2G12A	0.687909	-0.30136	0.859286
ULBP3	0.107007	0.574714	0.371691
ULBP2	0.304876	0.650971	0.606083
EDEM3	0.001133	0.730157	0.032731
FAM129A	0.474191	0.152355	0.727018
TRIM8	0.192496	0.287256	0.486094
UBXN6	0.035078	0.284328	0.223337
SLC19A3	0.163438	-0.20957	0.453012
TM6SF1	0.271143	0.826016	0.574669
TSGA10	0.146464	0.628341	0.435621
UCK2	0.039094	1.551641	0.236232
SIGLEC1	0.359171	0.236043	0.643861
API5	0.09581	0.306817	0.352331
NLRP1	0.193248	0.512545	0.487116
NMES1	0.105374	0.63504	0.369374
SPRY4	0.415253	-0.47764	0.686028
DPY30	0.036237	0.828319	0.227448
PKIB	0.122745	0.945856	0.398993
TRIM9	0.410792	0.441557	0.683152
TRIM5	0.001363	1.412371	0.036688
TRIM4	0.54177	0.218366	0.775521
TRIM2	0.277869	-0.24186	0.579677
KRT23	0.982241	-0.05141	1
INO80B	0.018626	0.941114	0.165633
LRRCC1	0.702525	-0.11372	0.866226
SETD5	0.302149	0.339746	0.60359
UNK	0.183743	0.24701	0.474829
FTO	0.036237	0.686559	0.227448
ZDHHC5	0.15535	0.811886	0.445058
TANGO6	0.000747	1.368988	0.025761
TNKS1BP1	0.181644	0.293406	0.472575
SEMA4C	0.035203	1.020496	0.224002
AMBRA1	0.05757	0.771134	0.280499
UBE2O	0.289748	0.252107	0.593134
ZYG11B	0.72401	0.159452	0.876654
TANC1	0.260718	-0.43891	0.56373
EPT1	0.960293	-0.50738	0.994122
XPO4	0.050493	0.697514	0.263221
LNP	0.011636	0.368139	0.129699
CEP44	0.034878	0.964094	0.223337
TTYH3	0.078472	0.844457	0.32578
KLHL4	0.731297	0.140968	0.881419
SRCIN1	0.837634	0.183051	0.938119
MTMR12	0.078771	-0.37537	0.32578
WDR33	0.394781	0.228638	0.672349
BHLHE41	0.186982	-0.28511	0.479162
BCL11B	0.089073	0.720175	0.341477
SLC39A8	0.075938	-1.53356	0.321214
HLA-DRB1	0.997267	-0.14899	1
WDR12	0.021148	0.508075	0.173882
YIPF3	0.876815	-0.13166	0.958859
TINAGL1	0.305036	-0.35232	0.606083
NDEL1	0.47933	-0.45544	0.731833
ACTR6	0.025724	1.368332	0.193808
PRSS22	0.099208	0.78935	0.358779
ROGDI	0.486111	-0.2549	0.73612

C20orf27	0.081589	0.571494	0.329897
PDGFD	0.010576	1.099389	0.124802
PITHD1	0.997369	0.093904	1
IMUP	0.466777	-0.80864	0.721939
DERL2	0.014531	0.983748	0.147184
COMMD5	0.882017	-0.25124	0.961515
MAP1LC3B;MAP1LC3B2	0.008068	1.46022	0.107782
REXO4	0.124148	1.06295	0.400153
DDX24	0.407778	0.111754	0.680612
DNAI2	0.976419	0.03344	0.998921
POLR1E	0.200079	0.692259	0.493237
WDR61	0.044589	0.185648	0.24851
SLIRP	0.157109	-0.27047	0.447022
SRR	0.155958	0.753263	0.44616
CCDC90B	0.274985	0.112193	0.577625
NIF3L1	0.929044	0.070047	0.983115
EGLN1	0.077639	0.528705	0.324641
MCOLN1	0.092536	1.073216	0.347013
TMEM39B	0.954108	0.047644	0.993074
CTDSP1	0.693827	-0.24885	0.861478
FAM192A	0.10745	0.853441	0.372977
EIF5A2	0.000175	2.476585	0.009596
WWTR1	0.001001	1.897558	0.030576
FGF23	0.322009	0.088518	0.613139
CHST6	0.023287	1.28756	0.18431
TWSG1	0.415505	0.621247	0.686235
COA1	0.851978	0.427415	0.945779
LAT2	0.03862	1.15652	0.235412
MFF	0.67051	0.154682	0.850681
NAA50	0.850885	-0.00687	0.944945
LACRT	0.322009	0.163854	0.613139
UBA5	0.206534	0.187475	0.500256
MKRN2	0.079897	0.489572	0.325919
LHPP	0.939534	0.074485	0.986498
MTFR1L	0.515875	0.146932	0.757825
TMEM126A	0.861239	-0.01195	0.950165
MAF1	0.601819	0.29007	0.81176
PAIP1	0.093209	0.288059	0.347256
CLPB	0.749036	-0.08261	0.890902
MIS12	0.021227	1.66154	0.174388
RAB33B	0.709344	0.164959	0.869179
LSG1	0.106075	0.650101	0.370631
NUAK2	0.389717	-0.29643	0.668163
FAM107B	0.950035	0.305223	0.990863
NAT10	0.081043	0.227691	0.328405
COMMD4	0.123514	0.396496	0.399387
SPATC1L	0.845762	-0.10297	0.942239
KLC2	0.05165	0.919636	0.266309
CRISPLD2	0.087621	1.072931	0.339301
BTBD1	0.396223	0.438609	0.67382
ILKAP	0.357478	0.239436	0.641472
XRN2	0.037428	0.347252	0.231646
TOLLIP	0.093209	-0.28111	0.347256
SAP130	0.059591	0.750828	0.285693
BRD8	0.112677	0.650908	0.382523
SHARPIN	0.486301	0.42473	0.73612
ARL6	0.092844	1.252744	0.347256
NSRP1	0.00491	1.826438	0.082133

INTS2	0.175254	0.351789	0.466796
RACGAP1	0.046424	1.247666	0.253695
ENKD1	0.632486	-0.3261	0.830965
PARP12	0.194929	0.180741	0.488911
SIK2	0.186647	0.498293	0.479162
PUS7L	0.095134	0.57087	0.35136
CSTF2T	0.063339	0.472	0.294055
WWP1	0.205263	0.420521	0.49957
PCBD2	0.835401	-0.34192	0.935999
NT5C3A	0.882016	0.34592	0.961515
FAM49A	0.840553	-0.565	0.939212
FXYD6	0.172617	0.490185	0.464157
AP5M1	0.981514	-0.07336	1
TMEM222	0.343799	-0.26152	0.630156
HDHD2	0.892438	0.143664	0.966699
GBP3	0.79579	0.157102	0.914433
QRSL1	0.141963	0.649934	0.428816
GABARAPL1	0.069462	1.154196	0.307321
DDX47	0.236413	0.131172	0.537858
RAB17	0.15276	-0.87734	0.443165
MAGT1	0.114161	0.283671	0.385201
RAB1B	0.709387	-0.03042	0.869179
MRPL18	0.918566	0.181661	0.978657
TSPYL1	0.343912	-0.69887	0.630156
TMEM168	0.274972	0.286335	0.577625
LMAN2L	0.032856	0.398493	0.217297
CCDC8	0.19032	0.660947	0.483515
SMG9	0.001561	1.385065	0.040678
C11orf54	0.03232	-0.59459	0.215938
ITFG3	0.083363	0.448531	0.332962
OSBPL5	0.000756	1.360548	0.025904
RBM38	0.619338	0.299083	0.82294
NAPB	0.675487	-0.2514	0.853186
GZF1	0.1816	0.541829	0.472575
DNTTIP1	0.574003	0.185487	0.795783
ING2	0.506417	0.30775	0.751294
BCL11A	0.393617	0.253337	0.671013
ZBP1	0.38106	0.565887	0.661698
SIL1	0.78936	-0.09609	0.911483
SDCBP2	0.594052	0.312495	0.806337
METTL9	0.136741	1.089896	0.421544
ANAPC1	0.011099	0.908745	0.128038
XYLT2	0.031321	1.011194	0.212999
IRF2BPL	0.415291	0.275971	0.686028
GLT8D2	1.61E-08	4.37618	6.25E-06
UNC93B1	0.754005	-0.05836	0.894351
CYSTM1	0.757749	-0.78763	0.896847
POLR3F	0.061465	0.82903	0.289565
NUCKS1	0.492705	0.649937	0.740961
TMX4	0.986843	0.040162	1
KIF13A	0.238311	0.404498	0.540624
ASCC2	0.028771	0.699029	0.204946
WNT5B	0.304818	0.487253	0.606083
RBSN	0.017992	0.802358	0.162636
ISCU	0.198358	-0.28759	0.49217
SLC25A18	0.17188	0.384361	0.463149
MESDC1	0.161733	0.277056	0.453012
SLC35B3	0.933816	0.04749	0.984157

OSBPL2	0.609193	0.543293	0.816529
MEGF9	0.442398	-0.43515	0.703763
SLC25A51	0.102183	1.045828	0.364372
RSPH9	0.645144	-0.21688	0.837353
DNAJC25	0.336446	0.398183	0.624
ATG5	0.206534	0.2398	0.500256
WDR13	0.314461	-0.13691	0.612305
TSPAN10	0.779828	0.072616	0.907277
EPN3	0.219743	0.429154	0.517665
MED28	0.812333	-0.49818	0.923871
EHD4	0.97106	-0.03469	0.997592
MMP28	0.449805	0.523547	0.709598
P2RY12	0.948081	0.112405	0.990595
C1orf21	0.744057	0.173506	0.888303
CARD9	0.019248	1.647624	0.167058
VPS33B	0.043893	0.280702	0.247494
VPS16	0.004244	0.3267	0.074174
VPS11	0.050495	0.230315	0.263221
SH3BGRL3	0.277896	0.310743	0.579677
ALDH8A1	0.994878	-0.03094	1
CXCL16	0.361038	-0.62577	0.644411
GAN	0.001663	1.770774	0.042203
ARV1	0.4798	-0.54686	0.732278
SLC25A32	0.173267	0.647823	0.464598
TRIOBP	0.675324	0.084956	0.853175
SEMA6A	0.555312	0.067301	0.783439
HSD3B7	0.029729	1.479613	0.208169
EPC1	0.2923	0.485112	0.595788
CCDC68	0.440215	-0.20443	0.70314
SLK	0.149515	-0.1716	0.438811
BLZF1	0.068987	0.771631	0.306571
PPIL3	0.105374	0.21548	0.369374
C10orf11	0.710654	-0.44684	0.870136
PDCL3	0.089413	0.641913	0.341477
MTIF3	0.44953	0.312214	0.709598
TAOK3	0.81486	-0.01251	0.924847
RASSF4	0.930329	0.1283	0.984006
BHMT2	0.319672	0.1668	0.613139
RAB3GAP2	0.007333	0.417758	0.102825
ADNP	0.197211	0.42216	0.490309
DPH5	0.555984	0.193859	0.783439
KCNN2	0.089503	0.363107	0.341477
RANBP17	0.053904	-0.51463	0.271999
DHX36	0.005656	0.478645	0.088608
PPA2	0.103963	-0.2338	0.366863
SPNS1	0.113397	0.137643	0.384261
MS4A6A	0.655968	0.367792	0.84209
MRPL46	0.490639	0.090614	0.738656
CHRD	0.938446	-0.15896	0.986498
CLEC4M		0	
IFI27L2	0.248603	1.44733	0.552225
ZNF106	0.623189	-0.05991	0.825978
PARL	0.264348	0.66423	0.566943
PNN	0.063339	0.414839	0.294055
EPB41L4B	0.818809	-0.25877	0.92687
TMEM245	0.604582	0.054579	0.813002
FOXP1	5.59E-05	2.517493	0.003976
GGNBP2	0.359903	0.157735	0.644411

TP63	0.993518	0.040787	1
SNX25	0.532956	-0.49207	0.7709
KCTD10	0.046529	1.064667	0.253921
CPVL	0.81486	0.051913	0.924847
TRIT1	0.232663	0.710111	0.53459
C11orf68	0.101186	0.629867	0.361901
DPAGT1	0.798119	-0.5245	0.916038
C12orf65	0.43065	0.181558	0.695651
GHITM	0.606871	0.247708	0.815541
BOLA2	0.804634	-0.22798	0.918848
MMADHC	0.053356	1.408835	0.270832
TXNIP	0.257225	0.715559	0.560462
TMX1	0.175259	0.21749	0.466796
NELFA	0.939533	0.176451	0.986498
ACBD3	0.011206	0.328325	0.128397
CDC42EP4	0.588787	-0.67884	0.80405
MUC13	0.767316	-0.22174	0.901915
CENPH	0.312224	0.596402	0.610732
SEMA4A	0.913998	0.074147	0.97604
TPK1	0.633942	0.250958	0.832269
PIGM	0.410867	-0.55438	0.683152
PTPN23	0.068179	0.151182	0.303928
UNC45A	0.694713	-0.07856	0.861478
MFSD1	0.019768	1.479556	0.1684
SMOC2	1.04E-05	2.928652	0.000991
PPDPF	0.746826	0.079814	0.889884
DNAJC5	0.604602	-0.16767	0.813002
LIME1	0.476921	0.493108	0.729993
DSN1	0.009583	1.043478	0.118893
C1orf198	0.090616	0.293203	0.343901
CHMP4B	0.511623	0.105647	0.754376
RWDD1	0.338634	0.645694	0.62586
FZD8	0.77733	-0.16797	0.906372
CUEDC2	0.440215	-0.19851	0.70314
FBXL15	0.518646	-0.39835	0.760272
RBKS	0.835743	0.341005	0.936286
FN3K	0.148584	-0.29822	0.438051
POFUT1	0.001332	0.496983	0.036119
PIGU	0.010589	0.424644	0.124802
MAP1LC3A	0.039169	1.395114	0.236232
TOR3A	0.043824	0.51263	0.247494
WNK1	0.840557	-0.03893	0.939212
RNPEP	0.343873	-0.1735	0.630156
GOLPH3L	0.150437	0.573913	0.440469
GOLPH3	0.404052	0.170519	0.678271
OSGEPL1	0.849688	-0.15925	0.944945
SAV1	0.099814	0.893227	0.359216
TUBB1	0.611978	0.323386	0.81882
RHOJ	0.716148	0.20648	0.873229
DEF6	0.604602	0.100795	0.813002
ST6GALNAC4	0.308182	-0.5843	0.609277
SMOC1	0.121191	0.71912	0.39685
EPB41L1	0.047465	-0.36053	0.255923
GLIPR2	0.301936	0.092318	0.603275
FAM83D	0.256399	0.370887	0.560009
ZHX3	0.064416	-0.93551	0.296449
TRABD	0.70448	-0.19931	0.867291
SMDT1	0.900141	0.605259	0.971583

RIBC2	0.175422	-0.54711	0.466796
MTG2	0.70382	0.187147	0.867291
SENP3	2.95E-05	2.503735	0.002271
OSBPL3	0.11568	0.508937	0.387731
SMARCAD1	0.044385	1.364367	0.24851
FBXO44	0.450001	0.446473	0.709598
PLEKHA4	0.140706	0.714723	0.427319
EHD1	0.934288	-0.08635	0.984157
RNF41	0.090633	0.9838	0.343901
RGCC	0.594272	-0.08437	0.806337
PCIF1	0.09193	0.405759	0.345626
ESF1	0.011106	1.525474	0.128038
ALG2	0.507389	0.068155	0.75153
ACTL8	0.992139	0.024041	1
ZNF644	0.237941	0.385405	0.540557
HEATR1	0.347246	0.205158	0.632606
ZNF768	0.001448	1.38648	0.038801
POMK	0.077145	1.178347	0.324464
RABEP2	0.689846	0.153642	0.859429
TFB2M	0.1422	0.169974	0.428816
CDCP1	0.086268	1.370971	0.336929
CXorf56	0.523284	-0.49301	0.764023
FAM96A	0.223033	0.567992	0.521792
SLITRK6	0.52523	0.294146	0.764479
DHX35	0.047292	1.072057	0.255923
RPP21	0.358581	0.413164	0.642907
NSUN3	0.156489	0.509275	0.44698
IGFLR1	0.070511	0.911715	0.31021
OBFC1	0.205838	-0.55716	0.500256
BICC1	0.042338	1.213601	0.243142
DENND2D	0.923728	0.289761	0.980751
PCNXL3	0.022698	0.764747	0.180981
CLMP	0.218898	0.425441	0.516332
EPHX3	0.520927	-0.34867	0.761212
HAUS4	0.024014	1.359511	0.187298
CCDC134	0.28668	-0.17141	0.590053
TUT1	0.62435	0.278292	0.827221
TMEM38A	0.651513	0.049721	0.839222
CCDC86	0.015451	1.470291	0.152523
REEP4	0.470893	0.133171	0.725372
C6orf106	0.139822	0.431752	0.426504
OPA3	0.923803	0.256853	0.980751
PRR36	0.305919	0.152299	0.607619
TMEM231	0.908077	0.065708	0.9734
ARMC7	0.389868	0.398718	0.668319
MYH16	0.684849	0.212048	0.85709
TASP1	0.15389	0.947895	0.444477
NARFL	0.96843	0.237536	0.997592
DHX33	0.449127	0.422149	0.709482
ACSS3	0.327317	0.312848	0.617719
NOL6	0.157109	0.294481	0.447022
ZDHHC6	0.003786	2.297063	0.07058
C2orf44	0.534225	0.128866	0.771496
YTHDC2	0.000433	0.988578	0.018693
AZI2	0.814632	0.060526	0.924847
EPS8L2	0.426712	-0.18306	0.693599
ESRP2	0.214093	-0.21811	0.50977
RPAP3	0.155183	0.344694	0.444958

BCAS3	0.008345	1.117447	0.110054
ALG9	0.794443	0.026156	0.913708
C2orf43	0.269152	1.020051	0.571371
NO66	0.05451	0.4789	0.273313
ANTXR1	1.71E-07	1.850708	3.80E-05
TMEM134	0.046695	-1.07709	0.254194
WDR55	0.646641	-0.23643	0.837353
MAGIX	0.584665	-0.40249	0.800854
RNF167	0.58855	-0.24908	0.803893
RANBP3	0.024706	0.423155	0.190022
GAREM	0.22359	0.380362	0.521792
ZNF552	0.291769	0.227139	0.595183
CWH43	0.09909	0.284047	0.358568
C12orf49	0.794727	0.073111	0.913708
GREM2	1	-0.13589	1
DCTPP1	0.382101	1.035599	0.661698
SH2D4A	0.713141	0.099653	0.871276
PEAK1	0.000464	1.274981	0.019585
RPF2	0.581411	-0.34688	0.797998
SMYD3	0.182444	1.045528	0.473679
SYNC	0.025084	1.14148	0.191949
AAMDC	0.138259	-0.7026	0.424067
DOCK5	0.002686	1.291993	0.057778
WDR26	0.145824	0.274048	0.434185
TDRD3	0.35484	0.536106	0.638989
C8orf33	0.958385	-0.05523	0.994122
ATP13A3	0.035077	0.656279	0.223337
TMEM185B	0.484075	0.263149	0.735071
METTL17	0.058889	1.313204	0.283482
SUDS3	0.332292	0.257684	0.620028
C10orf54	0.319239	-0.60345	0.613139
SCAF1	0.028958	1.059925	0.20582
MVB12B	0.378165	-0.56069	0.659304
PLEKHG2	0.057155	0.834051	0.279008
ZNF703	0.163438	-0.08491	0.453012
CCSER2	0.088468	0.335114	0.339985
ZNF696	0.540968	0.163123	0.775521
IFT22	0.320389	0.760336	0.613139
CXorf36	0.303347	-0.88007	0.605331
NRDE2	0.118301	0.857308	0.392409
KAT8	0.618774	0.31892	0.822486
PTGES2	0.382127	0.246543	0.661698
TMEM206	0.001245	1.767815	0.034693
PHAX	0.150236	1.046662	0.440175
UBE2Z	0.047096	0.464524	0.255677
GEMIN7	0.793775	-0.17503	0.913708
ACAD9	0.343873	0.15485	0.630156
NT5DC2	0.04253	0.689355	0.243142
VCPKMT	0.163587	0.446488	0.453012
YY1AP1	0.267449	0.44607	0.570195
RMND5A	0.06238	1.212611	0.292127
PRKRIP1	0.273551	0.644276	0.577625
TTC12	0.40818	-0.23825	0.680975
ZMAT4	0.172617	0.444128	0.464157
CAAP1	0.065702	0.989958	0.299507
NOL11	0.404052	0.144874	0.678271
DDX31	0.418612	0.439178	0.688796
METTL7A	0.72417	0.000746	0.876654

MANSC1	0.385408	0.541596	0.664306
MMRN2	0.364426	-0.36801	0.646996
COQ10B	1	-0.0501	1
BRD9	0.410079	0.328719	0.682809
CNNM2	0.026184	0.666341	0.194901
FAM188A	0.306398	-0.58819	0.607918
ZNF395	0.178261	0.301234	0.469506
SRD5A3	0.758577	-0.28576	0.896847
MOB1A	0.044589	0.47981	0.24851
AKTIP	0.192398	0.406299	0.486094
ZFAND3	0.66564	-0.10055	0.847351
ECT2	0.446152	-0.41275	0.706795
PLEKHF2	0.352344	-0.22342	0.637424
ANKZF1	0.43171	0.365513	0.696755
GORASP2	0.520146	0.039036	0.760272
ZWILCH	0.829882	0.042068	0.933615
HN1L	0.49864	0.928715	0.744839
RNF121	0.033229	0.745913	0.219502
SP140L	0.261524	0.63075	0.564861
SLC25A22	0.744041	-0.00965	0.888303
PSTPIP2	0.591686	-0.50626	0.805893
MED20	0.015747	0.853246	0.15374
WDR76	0.167504	0.567531	0.457358
C14orf93	0.18654	-0.90746	0.479026
QTRTD1	0.036232	0.490202	0.227448
WDR54	0.852863	0.203846	0.946466
ACTR8	0.398372	0.292965	0.675933
ARMT1	0.317645	0.184169	0.613139
CNOT10	0.003027	0.830357	0.062294
LRRC40	0.021897	0.425029	0.176756
RMI1	0.310077	0.335507	0.609864
EHMT1	0.104394	0.889124	0.368033
SFXN1	0.600007	0.121859	0.810262
VIPAS39	0.244338	0.300204	0.547425
COG4	0.074384	0.350109	0.317894
ACTR5	0.047734	1.179295	0.256846
AGO3	0.040551	0.376133	0.240216
VPS37B	0.013012	0.45349	0.139171
MRPL44	0.400346	0.149771	0.67622
ISG20L2	0.112796	1.006623	0.38281
KANSL2	0.029097	1.297514	0.206601
UNKL	0.084824	1.105777	0.335537
L2HGDH	0.680152	0.248565	0.85545
COPS7B	0.675324	0.062635	0.853175
NHEJ1	0.82473	0.112264	0.930395
SEC61A2	0.178261	0.313899	0.469506
CAB39L	0.546872	-0.37208	0.779208
FKRP	0.058064	0.976731	0.281
ELP3	0.001789	0.43855	0.044281
JMJD4	0.993518	-0.0077	1
RPF1	0.876747	-0.11005	0.958859
GPN2	0.319799	0.384637	0.613139
POLR1B	0.085728	0.372801	0.335537
LIN28A	0.389093	-0.49819	0.667643
UCK1	0.783548	0.094974	0.909506
FN3KRP	0.809743	0.038796	0.921586
TBC1D17	0.181644	0.266795	0.472575
CALHM2	0.202437	0.681353	0.496184

CARS2	0.500974	0.394261	0.746514
CERS4	0.260755	-0.78763	0.56373
EFCC1	0.479442	0.822437	0.731833
RSAD1	0.09909	0.339884	0.358568
SLC52A2	0.903871	0.209879	0.972246
PPCS	0.490639	-0.11019	0.738656
SUGCT	0.049226	0.987707	0.260606
UBTD1	0.073254	1.468572	0.31592
WDR41	0.003933	1.686268	0.071362
MEAF6	0.150906	0.485124	0.44125
FBRS	0.416936	0.315471	0.687847
SAP30L	0.693244	0.478991	0.861478
NMNAT1	0.590862	0.21195	0.804875
MLXIP	0.023789	1.032266	0.186463
LIN7B	0.01083	1.152029	0.126436
LPHN3	0.447034	-0.3017	0.707261
C17orf75	0.751429	0.182206	0.893066
SIAE	0.809743	0.00627	0.921586
PLEKHA5	0.524434	-0.30587	0.764023
SMURF2	0.122431	0.913723	0.398323
UPF2	0.478274	0.219379	0.730754
GNB4	0.572777	0.159507	0.794481
XPO5	0.025131	0.60584	0.191949
GRPEL1	0.422885	-0.14396	0.691467
UGT1A7	0.291769	0.260026	0.595183
UGT1A10	0.126551	0.726508	0.403998
CLK4	0.253685	0.261655	0.557407
PRDM16	0.198855	-0.43989	0.49217
C12orf10	0.01507	0.806724	0.150049
BCL2L12	0.59722	0.204427	0.808171
PLEKHA2	0.108807	0.374354	0.375709
PLEKHA3	0.375011	0.535557	0.656862
PLEKHA1	0.432138	0.603544	0.697141
SCPEP1	0.71923	0.074716	0.874478
SP110	0.699576	-0.20835	0.864146
NTN4	0.139726	-1.01051	0.426504
CACYBP	0.14042	0.196518	0.426568
RRAGC	0.694713	0.067027	0.861478
TRPV4	0.005841	1.206852	0.089662
PATZ1	0.041395	0.78141	0.240826
ZFYVE1	0.031167	0.654501	0.212999
ATP6V0A4	0.357062	-0.70563	0.641472
IFT122	0.087021	0.818636	0.337936
LY9	0.867668	-0.04116	0.954126
RHOF	0.548329	0.576837	0.780282
PDF	0.176313	-0.55976	0.468258
RDH14	0.498977	-0.10272	0.744839
MKNK2	0.722377	-0.10257	0.876654
PARVG	0.009999	0.709955	0.120967
PARVB	0.656074	-0.20693	0.84209
CYP4F11	0.974738	0.108306	0.998921
AS3MT	0.920433	-0.05297	0.980269
TNS1	0.861239	0.139246	0.950165
PLGRKT	0.311298	0.305686	0.609894
NMRAL1	0.448077	0.324566	0.708126
VEZT	0.063479	0.518415	0.294578
SPC25	0.008061	1.508065	0.107782
TAF9B	0.487804	0.262941	0.737793

SLC38A10	0.729121	0.091017	0.879656
BARX1		0	
ETNK1	0.373317	-0.6544	0.656487
LPAR2	0.001994	1.079125	0.047089
ELTD1	0.595609	0.250615	0.806482
TMEM165	0.257958	0.346297	0.560462
APOBEC3G	0.653758	0.404474	0.841555
SLC25A19	0.006911	0.933671	0.099675
TMBIM4	0.319672	-0.24402	0.613139
EML4	0.81486	0.084393	0.924847
RNMTL1	0.252079	0.611854	0.555643
GLOD4	0.236413	-0.09757	0.537858
CBX8	0.816942	0.382835	0.926736
SENP2	0.428197	0.28516	0.695233
ZBTB20	0.572756	0.223791	0.794481
MUC5B	0.636834	-0.57171	0.833511
NEK6	0.118895	0.749603	0.392576
SPON1	0.021885	1.0443	0.176756
MCCC2	0.228671	-0.14743	0.528484
ZFYVE28	0.481288	0.066539	0.733586
NCOA5	0.537409	0.11119	0.773175
TANC2	0.00226	1.399089	0.051155
EPG5	0.857224	0.141773	0.948093
MOV10	0.006642	0.649761	0.097053
ZNF532	0.176351	0.665039	0.468258
METTL14	0.455535	0.373007	0.71417
ARHGEF10L	0.103953	0.430814	0.366863
SMURF1	0.870327	-0.16803	0.956098
ANO8	0.63741	0.434233	0.833511
GBA2	0.745637	0.157658	0.889041
CWC22	0.008089	0.679906	0.107782
NCKAP5L	0.874055	-0.08186	0.957716
SYTL2	0.247715	1.019503	0.550914
TNRC6C	0.061865	0.742165	0.290811
ANKH	0.010941	1.474125	0.127049
RAVER2	0.1061	0.746571	0.370631
VAT1L	0.299766	0.335042	0.602797
ROBO2	0.665748	-0.22461	0.847351
CHD8	0.182435	0.835099	0.473679
PCDH18	0.15526	0.105739	0.444958
GPAM	0.708911	0.285223	0.869179
ZFP14	0.104629	-1.06378	0.368512
PLXNA4	0.600731	0.220559	0.811041
EPB41L5	0.011369	-0.57921	0.128971
TMEM8A	0.473532	0.361894	0.727018
GPN1	0.197211	0.33959	0.490309
GP6	0.960709	0.128988	0.994122
SDF2L1	0.066208	0.279668	0.300455
CSNK1G1	0.177789	0.751187	0.469455
HHATL	0.438587	-0.36279	0.701933
CYP4F12	0.638802	0.190704	0.834211
EPB41L4A	0.167464	0.413471	0.457358
XAB2	0.422885	0.148898	0.691467
CD248	0.041786	1.411365	0.242066
CELSR2	0.616056	-0.34773	0.821456
PREB	0.161013	0.201146	0.453012
POLD4	0.204523	0.708423	0.49865
BRMS1	0.199824	0.621929	0.492817

S100A14	0.651339	-0.52828	0.839222
SRA1	0.053969	1.165381	0.272201
ATP13A1	0.131757	0.200676	0.413476
MRS2	0.168533	0.935557	0.459489
GOPC	0.057044	0.399017	0.278658
MRPL47	0.396661	0.377659	0.67382
LYRM4	0.830201	-0.54081	0.933615
SEPSECS	0.689552	-0.42456	0.859429
CHMP1A	0.311298	0.115297	0.609894
PTPRH	0.511124	0.20352	0.754376
TM9SF3	0.101186	0.241142	0.361901
RANGRF	0.35278	0.63166	0.637972
XAGE1A	0.5791	-0.34969	0.796675
MYO10	0.862376	-0.18985	0.95104
RETN	0.872772	0.087019	0.956872
JPH1	0.930329	0.088621	0.984006
APMAP	0.71923	0.130107	0.874478
WDR6	0.118762	0.587883	0.392409
TXNRD2	0.11568	-0.34239	0.387731
TUFT1	0.300407	0.74515	0.602797
CD209	0.704719	0.382406	0.867485
SH2D2A	0.028968	0.779397	0.20582
BPIFA1	0.07384	1.636212	0.316943
ABCB6	0.7919	0.011967	0.913596
SLC40A1	0.646844	0.354591	0.837416
ARFGAP3	0.013753	0.579521	0.143228
ZCCHC17	0.964981	-0.57166	0.99616
HMG20A	0.267798	0.27562	0.570195
RAB18	0.434427	0.129894	0.698194
ALG13	0.369669	-0.60033	0.652969
PALMD	0.252262	0.652882	0.555643
SSU72	0.503169	0.601045	0.747596
ABCB9	0.042259	-1.0274	0.243142
VTA1	0.153276	0.22008	0.443165
PNPLA8	0.45805	0.264102	0.715224
SARS2	0.572777	0.509998	0.794481
POLM	0.920812	-0.07311	0.98039
RAB9B	0.541937	-0.21567	0.775521
MRPS30	0.09193	0.342376	0.345626
DYNLRB1	0.72417	-0.00428	0.876654
MYOZ1	0.512878	-0.32312	0.755723
TREM1	0.664648	0.401908	0.847351
EMC7	0.32085	0.187406	0.613139
MMP25	0.942624	-0.17762	0.988547
MID1IP1	0.411887	-0.29376	0.683152
ENY2	0.1068	0.082445	0.371671
SAYSD1	0.609519	-0.3803	0.816842
GPCPD1	0.077749	0.951804	0.324826
A4GALT	0.229395	0.386006	0.529537
EXOSC4	0.78936	-0.16193	0.911483
UBE2T	0.089389	1.172297	0.341477
NGRN	0.491197	-0.52045	0.739395
NOP10	0.784284	-0.13446	0.909506
CD320	0.265497	0.640586	0.568807
CHST11	0.107001	0.808995	0.371691
OSGEP	0.537409	-0.09085	0.773175
DMAP1	0.197211	0.22539	0.490309
ADAP2	0.372948	0.715879	0.656059

FZD3	0.992139	-0.05649	1
UBN1	0.593889	-0.27798	0.806337
PCDH12	0.251916	-0.52655	0.555643
ZDHHC4	0.848267	0.294603	0.944084
ACP6	0.462044	0.724714	0.718747
ISYNA1	0.164991	0.501471	0.454525
IL1RAP	0.143272	0.717955	0.4311
TMEM138	0.130119	0.616956	0.410919
BRD7	0.023293	0.695676	0.18431
DCP1A	0.006911	0.931991	0.099675
KCNJ16	0.354428	0.538401	0.63835
ACOT13	0.38573	-0.20018	0.664306
MED4	0.027819	0.506701	0.202072
NXT2	0.717577	0.335347	0.874289
TIMMDC1	0.148584	0.481193	0.438051
NLRC4	0.498579	0.341391	0.744839
RIC8A	0.062406	0.397395	0.292127
SEMA4B	0.161211	0.952334	0.453012
GPR108	0.216825	-0.35019	0.513662
CD93	0.902877	-0.31543	0.971583
ATP13A2	0.125788	0.87825	0.403148
SLAMF7	0.121935	0.939717	0.397343
LUC7L	0.774093	0.218753	0.903913
ESM1	0.312494	0.439855	0.610732
TMEM9B	0.197379	0.873769	0.490616
NRIP3	0.69426	0.057882	0.861478
SCUBE2	0.303209	0.559466	0.605162
SLC52A3	0.988991	0.013844	1
LZTFL1	0.0799	0.288455	0.325919
MRPL40	0.871617	-0.03825	0.956098
PPAN	0.950027	-0.04793	0.990863
PLCB1	0.307327	-0.57854	0.609175
CASS4	0.285949	0.380927	0.590053
CRTAC1	0.366062	-0.11049	0.648976
GPRC5C	0.242299	-0.78688	0.54532
TRIM36	0.352923	0.20371	0.638094
TIGAR	0.086954	0.650665	0.337854
C12orf4	0.042323	1.059334	0.243142
COPRS	0.727328	0.214601	0.878927
A1CF	0.69426	0.073032	0.861478
WASH6P	0.144838	0.9818	0.43345
TCF7L2	0.925516	0.031431	0.981917
JADE2	0.144422	0.689114	0.432798
RTN4	0.151386	0.207127	0.44125
CYLD	0.000414	1.616812	0.018068
IFT46	0.137959	0.869192	0.423499
HINT3	0.343873	-0.10363	0.630156
MANBAL	0.512869	0.427941	0.755723
RPRD1B	0.192667	0.269337	0.486094
MIEF1	0.182925	0.811761	0.474175
HPS4	0.593889	-0.26405	0.806337
XPNPEP3	0.133456	0.361899	0.416228
PFDN4	0.572777	0.018078	0.794481
SLC35C2	0.093078	0.685314	0.347256
SETD8	0.992139	-0.11742	1
NIT2	0.009264	0.413879	0.116462
AVEN	0.039924	1.206823	0.23845
PVRL3	0.229479	-0.68848	0.529537

INCENP	0.040913	1.031908	0.240826
EXOSC5	0.744041	-0.04204	0.888303
EXOSC3	0.226131	0.138402	0.525131
KIF13B	0.231232	0.213947	0.531962
ANLN	0.061941	0.999988	0.290972
XPNPEP1	0.185996	0.283845	0.478129
GPHN	0.430555	0.218069	0.695651
MYO5C	0.236413	-0.3261	0.537858
ZNF331	0.331588	-0.08711	0.619341
ITM2C	0.008282	1.766538	0.109484
BIN3	0.0856	-1.08612	0.335537
UTP3	0.208708	0.174706	0.503054
STARD7	0.200822	-0.92775	0.494233
C8orf4	0.77512	-0.13835	0.904456
BIRC6	0.314461	0.230523	0.612305
PDLIM7	1.14E-08	1.095335	5.48E-06
CD163L1	0.390897	0.431765	0.669566
ACSS2	0.604598	-0.2679	0.813002
DIABLO	0.244338	0.16528	0.547425
DDX21	0.130075	0.268885	0.410899
SAR1A	0.114161	0.234619	0.385201
POLE4	0.116372	1.285659	0.389223
MAN1C1	0.424623	0.551105	0.693599
NANS	0.784286	-0.08293	0.909506
SH3GLB2	0.950035	0.082824	0.990863
EIF2B3	0.478274	0.167244	0.730754
MBNL1	0.257958	0.249718	0.560462
DLL4	0.322009	0.093579	0.613139
PXMP2	0.901157	-0.0214	0.971583
ARHGEF3	0.030204	0.599075	0.209418
TLR8	0.744142	-0.22102	0.888309
MXRA5	2.74E-09	2.477011	2.03E-06
SPHK2	0.385408	0.415539	0.664306
PDGFC	0.002389	1.855365	0.053103
SLC17A5	0.95522	0.5225	0.993074
EIF4ENIF1	0.030079	1.19344	0.209418
CHST12	0.006608	1.675347	0.097053
ST7	0.642916	0.143597	0.836333
SIRT7	0.904222	-0.36368	0.97253
OTOR	0.322009	0.225747	0.613139
FBXO6	0.352544	0.617366	0.637649
PICK1	0.613484	0.209046	0.819264
DUOX2	0.178261	0.269514	0.469506
DUOX1	0.000846	-1.99895	0.027765
TSHZ2	0.571349	0.112664	0.793891
SH2B1	0.208023	0.599275	0.502982
CTPS2	0.546147	0.052831	0.778738
POLE3	0.031578	1.492462	0.214075
CHRAC1	0.431975	0.788672	0.696978
PRTFDC1	0.216064	0.857473	0.512741
SMYD2	0.649093	0.223127	0.839222
SDR39U1	0.053673	-0.9212	0.271145
AAAS	0.147661	0.379551	0.436386
YAE1D1	0.300882	0.242774	0.602797
SNRK	0.634013	-0.11647	0.832269
DISC1	0.010923	1.372041	0.127049
CCL28	0.760702	0.368567	0.897929
PCDHB16	0.340315	0.324045	0.627258

ABCB10	0.347172	0.420225	0.632606
BAZ1A	0.047453	0.92933	0.255923
STRN4	0.199513	0.132319	0.49217
SLC2A9	0.513476	0.212426	0.75602
IL17RB	0.556057	0.113918	0.783439
OLFML3	0.939534	0.077092	0.986498
AASDHPPT	0.244338	-0.30452	0.547425
METTL5	0.260666	0.542968	0.56373
OSTC	0.646028	1.227596	0.837353
CMC2	0.157086	0.616063	0.447022
SDHAF3	0.294734	-0.9378	0.597932
PLSCR4	0.711814	0.370308	0.870161
SMCO4	0.070995	-1.23476	0.311134
CYTL1	0.992139	-0.06861	1
CDC42SE2	0.299182	0.078088	0.602219
DROSHA	0.619	0.187319	0.822688
UBQLN4	0.000329	0.750555	0.015317
CDC42SE1	0.455561	0.963417	0.71417
TMPRSS4	0.546261	-0.47776	0.778738
SNX15	0.92904	0.56472	0.983115
HEBP1	0.035078	-0.36301	0.223337
RAB6B	0.93369	-0.12532	0.984157
APOBEC3C	0.866424	0.104761	0.953042
DUSP22	0.625609	0.454709	0.828579
VPS45	0.004157	0.394379	0.073531
PNO1	0.892432	-0.10764	0.966699
MRPL17	0.804634	-0.04808	0.918848
NDUFA4L2	0.290981	0.847836	0.595183
PHPT1	0.986843	-0.09226	1
SERINC1	0.26578	1.00345	0.568807
INIP	0.134764	1.132674	0.417833
ARHGAP35	0.192667	0.26047	0.486094
FAM114A2	0.181644	0.156614	0.472575
PLSCR3	0.997368	0.52473	1
AGPAT4	0.011319	1.910425	0.128971
AGPAT3	0.364426	-0.12748	0.646996
HELLS	0.103355	0.912034	0.366609
C1GALT1	0.53709	0.224485	0.773175
LTBP3	1.04E-07	1.742282	2.45E-05
GLRX2	0.638802	0.137613	0.834211
THSD1	0.055814	-0.54705	0.276127
TNFRSF19	0.384669	0.20731	0.66413
TOMM22	0.167008	0.244675	0.457139
MBIP	0.289643	0.898474	0.593134
CHST7	0.69117	0.160228	0.860356
LANCL2	0.632496	0.006869	0.830965
KIF15	0.522527	-0.15374	0.763249
TM7SF3	0.261211	-0.84586	0.564363
SEMA3G	0.638802	0.146595	0.834211
SLC22A11	0.671985	0.231886	0.851286
CTNNBIP1	0.427267	-0.68863	0.69413
KRT82	0.331588	-0.11841	0.619341
HOMER2	0.071983	-0.87892	0.313575
HOMER3	0.014224	1.305053	0.145268
ST6GALNAC1	0.208535	0.800702	0.503054
FARSB	0.665709	-0.05347	0.847351
IARS2	0.759058	0.058086	0.896847
C1orf112	0.149664	0.406866	0.439016

FAM207A	0.025487	1.146991	0.193268
SAMSN1	0.045356	1.156976	0.250872
KLC4	0.244338	-0.0786	0.547425
CENPM	0.626197	0.133924	0.828579
TREX1	0.224659	0.917878	0.523685
NRBP2	0.111611	-1.00648	0.381945
BMP2K	4.91E-05	0.78246	0.003559
STARD5	0.021086	1.414099	0.173882
ATG3	0.305036	0.162697	0.606083
SIRT3	0.684989	-0.18149	0.85709
PDS5B	0.97106	0.003687	0.997592
SMC4	0.552567	0.194008	0.783393
MAN2C1	0.211163	0.425975	0.506632
SACM1L	0.347246	-0.19233	0.632606
OLA1	0.825116	0.109727	0.930395
CUTC	0.437286	-0.48079	0.70193
ECHDC1	0.35064	-0.34557	0.635657
RNF146	0.707463	-0.19828	0.869179
RBM12	0.098469	0.293171	0.357719
TBC1D22B	0.901123	-0.14728	0.971583
MDN1	0.115676	0.468357	0.387731
LYRM2	0.166562	-0.78046	0.457139
GINM1	0.105925	-0.43399	0.370605
ZBTB40	0.437082	0.350899	0.70185
ACSS1	0.199513	-0.35954	0.49217
ZCCHC3	0.064547	1.092227	0.296805
ZDHHC18	0.19682	0.626019	0.490309
CCM2L	0.145176	0.460555	0.433769
PDRG1	0.122338	0.966946	0.398255
TMEM14B	0.322009	0.152298	0.613139
DECR2	0.153213	-0.7342	0.443165
ABHD10	0.026002	0.443255	0.193808
TCP11L1	0.00037	1.542591	0.016741
STAU2	0.078755	0.837924	0.32578
C19orf66	0.848286	0.59819	0.944084
DDX28	0.251236	0.583083	0.555182
SLC39A9	0.652505	0.172248	0.840332
TMEM106B	0.533062	-0.20148	0.7709
LMBRD1	0.586283	-0.48162	0.801141
BLOC1S4	0.462056	0.034244	0.718747
TRMT13	0.416958	0.245952	0.687847
LIN7C	0.45805	-0.16173	0.715224
AGPAT5	0.121914	0.71176	0.397343
TXLNG	0.458045	-0.29912	0.715224
SPATS2L	0.004611	0.821982	0.078316
UFSP2	0.887225	-0.06099	0.96416
ABCF3	0.019715	0.472686	0.1684
FAM49B	0.609213	0.044919	0.816529
AP5S1	0.143605	0.465893	0.431666
ABCB8	0.252443	0.437526	0.555643
FAM105A	0.976317	-0.39015	0.998921
DDX19A	0.031791	0.320497	0.214212
SPTLC3	0.871947	-0.12118	0.95627
GIMAP4	0.804634	-0.15188	0.918848
TDP1	0.120635	0.682305	0.39573
POT1	0.982375	0.068861	1
TBC1D23	0.001789	1.054045	0.044281
DCAF13	0.43831	0.425778	0.701933

TMEM100	0.568515	-0.35959	0.791838
IMP3	0.036531	0.584713	0.229165
NUDT15	0.100924	1.089983	0.361901
MRGBP	0.038864	0.701837	0.236232
TMEM39A	0.073265	0.866973	0.31592
TYW1;TYW1B	0.752463	-0.23157	0.893529
EXOC1	0.364426	0.120185	0.646996
INTS9	0.027355	0.808268	0.199874
TMEM30A	0.32085	-0.29654	0.613139
UQCC1	0.554951	0.037373	0.783439
SEPTIN11	0.001215	0.416629	0.034268
TMEM184C	0.310964	-1.07739	0.609894
SLC38A7	0.00038	2.379036	0.016995
MED17	0.221781	0.346271	0.520147
PARVA	0.001272	0.487571	0.034924
USP40	0.545246	0.347901	0.778738
PANK4	0.09581	0.251546	0.352331
FBXO28	0.246709	0.319562	0.549922
TBC1D13	0.918562	0.13762	0.978657
EXD2	0.419078	0.458659	0.688796
DNAJC11	0.269226	0.314604	0.571371
INTS7	0.133455	0.709909	0.416228
TMLHE	0.199469	0.617172	0.49217
FANCI	0.062726	1.02792	0.292924
ATAD3A	0.934288	0.047787	0.984157
ARL8B	0.255193	-0.27978	0.557593
FGFR1OP2	0.719189	-0.66723	0.874478
EVA1B	0.453566	-0.4663	0.712914
PRMT7	0.201482	0.564885	0.49489
DNAJC17	0.523132	0.485083	0.763931
ASUN	0.153276	0.259491	0.443165
RIC8B	0.281331	0.235747	0.583877
GNL3L	0.048956	1.154928	0.259694
DDX18	0.965801	0.00569	0.99616
ASF1B	0.190786	0.642778	0.484103
DZANK1	0.759161	0.289037	0.896874
FAIM	0.056125	1.342543	0.276683
KLHL11	0.30824	0.547796	0.609284
INTS10	0.0334	0.644761	0.219585
DNAAF2	0.69077	0.202134	0.86005
MRPS18A	0.606075	-0.38825	0.814786
PNPO	0.67051	0.020148	0.850681
ARMC1	0.729121	-0.02914	0.879656
POLR3E	0.074729	0.587175	0.318513
SDAD1	0.017975	1.392465	0.162611
TMEM38B	0.092448	1.08797	0.347013
MTPAP	0.301931	0.439942	0.603275
AIG1	0.551353	0.035942	0.782077
HAUS2	0.244419	0.665267	0.547425
NLE1	0.00635	1.255015	0.094329
KBTBD4	0.932694	-0.04636	0.984157
NECAP2	0.031269	0.381227	0.212999
ZNF358	0.089503	0.389183	0.341477
POLR3B	0.392268	0.533131	0.671013
RBM28	0.128409	-0.55469	0.407846
ANO10	0.09982	0.317747	0.359216
RBM22	0.036237	0.281945	0.227448
BSDC1	0.729121	0.19375	0.879656

GPATCH2		0.006364	0.855522	0.094355
ATP5SL		0.768941	0.120458	0.901915
WDR70		0.498911	0.223301	0.744839
TMEM51		0.046508	1.664334	0.253921
MED9		0.100009	1.2232	0.359674
RBFOX1		0.331588	-0.14783	0.619341
ARGLU1		0.292717	-0.6087	0.595788
IFT57		0.707758	0.269231	0.869179
TMEM248		0.217891	0.453787	0.515383
RNF216		0.096294	0.370644	0.353641
SPATA6		0.186982	-0.28428	0.479162
SLTM		0.809743	0.046666	0.921586
ZNHIT6		0.010683	1.489667	0.125661
ASNSD1		0.292894	0.428203	0.595821
CUEDC1		0.53191	0.353985	0.77068
FKBP14		0.000108	2.870824	0.006676
GPATCH2L		0.291769	0.258774	0.595183
PAG1		0.695622	-0.23857	0.862317
C14orf119		0.238106	0.657692	0.540624
CCDC109B		0.203722	0.746297	0.497963
PIH1D1		0.174016	0.857447	0.466074
FAM118A		0.267436	0.524322	0.570195
RMND1		0.850884	0.351303	0.944945
ZNF446		0.523974	0.35752	0.764023
PAK1IP1		0.031765	0.884371	0.214212
HIF1AN		0.239021	0.777946	0.541047
AURKAIP1		0.711083	-0.1608	0.870136
OXSM		0.908102	-0.14353	0.9734
GID8		0.015914	0.389842	0.153751
MRPL22		0.073311	1.031202	0.31592
C1orf123		0.528742	-0.11765	0.767576
BABAM1		0.121915	0.191592	0.397343
CLN6		0.263334	0.59887	0.565873
NMRK1		0.258501	0.846006	0.560954
ASB6		0.291151	0.409314	0.595183
THG1L		0.46151	0.353252	0.718747
C4orf27		0.399875	0.096301	0.67622
IRAK4		0.071133	0.884923	0.311134
UCKL1		0.568169	0.394461	0.791838
GEMIN8		0.038799	1.169465	0.236048
TMEM160		0.764432	0.126563	0.899556
TXNL4B		0.116476	0.767013	0.389223
NLRP2		0.963206	-0.10775	0.995704
C1orf109		0.808416	0.066735	0.921586
FAM120C		0.94475	0.240202	0.988547
TRNAU1AP		0.315847	0.749995	0.613139
COMMD8		0.382127	-0.07596	0.661698
NDUFB11		0.179497	0.244499	0.471072
SDHAF2		0.151566	1.079345	0.441443
MRPL16		0.144001	0.042554	0.431666
NHP2		0.892438	0.156508	0.966699
FAM206A		0.308969	0.497571	0.609864
OCIAD1		0.902877	0.01334	0.971583
ADPRHL2		0.675324	0.199464	0.853175
	5-Mar	0.109698	0.488048	0.377423
RHBDL2		0.908949	0.1009	0.974118
HYPK		0.404051	0.411429	0.678271
RAB20		0.921149	0.064563	0.980655

LYAR	0.037428	0.640472	0.231646
TMEM161A	0.871811	-0.09765	0.956216
IMPAD1	0.000337	0.698614	0.015578
CHCHD3	0.97106	0.007891	0.997592
MED29	0.123677	-0.70562	0.399565
DUS2	0.071634	0.885274	0.312422
CMTM6	0.006924	2.287681	0.099739
TMEM260	0.028095	1.056921	0.203207
SYBU	0.57342	-0.22307	0.795273
SIRT5	0.546147	0.033659	0.778738
MKS1	0.518766	0.278707	0.760272
GFOD1	0.042808	-0.97488	0.244479
MIOS	0.010589	-0.45048	0.124802
MTMR10	0.992034	-0.02924	1
SMPD4	0.520146	0.097478	0.760272
CWC25	0.662423	0.279623	0.845642
TEX10	0.008575	0.72313	0.111496
ZDHHC7	0.863483	0.100974	0.951788
THUMPD1	0.908103	-0.18117	0.9734
P4HTM	0.012846	1.358029	0.138596
PPP1R14D	0.041924	1.617424	0.242175
TOR4A	0.840557	0.033092	0.939212
TRMT1	0.565757	0.314793	0.79107
PGPEP1	0.369906	0.789927	0.653181
FBXL12	0.12446	0.984363	0.40081
ARHGEF38	0.52842	-0.34398	0.767576
GDAP2	0.524424	0.146638	0.764023
NDE1	0.000172	0.968158	0.009509
ANKRD10	0.81719	-0.0678	0.926828
BRE	0.419078	-0.05383	0.688796
ING3	0.672694	0.437567	0.851987
QPCTL	0.093206	0.619925	0.347256
ARL15	0.300393	0.202547	0.602797
KCTD5	0.001776	1.716686	0.044269
CDKN2AIP	0.819984	0.363421	0.92687
DNAJB12	0.44615	-0.27897	0.706795
ALKBH4	0.513352	0.417531	0.75602
NSMCE4A	0.04962	0.383009	0.261364
DDX43	0.382354	-0.24701	0.661883
GAR1	0.684992	0.026579	0.85709
STAB1	0.09193	0.480005	0.345626
CLEC5A	0.005097	2.071464	0.083708
SLC39A1	0.000677	2.225345	0.024383
PPP4R2	0.222358	0.28295	0.520729
DPP3	0.609213	-0.0366	0.816529
CLDND1	0.249041	0.254516	0.552533
CACNA2D2	0.005794	-1.83114	0.089494
SMPD3	0.008147	-1.27726	0.108342
AATF	0.043543	0.742558	0.247494
SLC2A8	0.574397	0.38294	0.796031
DDX56	0.46607	0.563396	0.721065
B3GNT2	0.427256	0.525535	0.69413
TERF2IP	0.000626	0.578047	0.023434
ABI2	0.016258	1.271143	0.154646
DNAH9	0.133198	0.606181	0.416228
FAM53C	0.257625	0.451829	0.560462
BCLAF1	0.098469	0.291137	0.357719
ZDHHC3	0.782768	0.158924	0.909458

UTP6	0.216168	0.285951	0.512741
PSD3	0.037619	0.826482	0.232686
COA4	0.124833	-0.581	0.401334
TAB2	0.057721	1.0604	0.280499
TLR7	0.087181	0.854896	0.338202
MRPL39	0.211314	0.199508	0.506632
ZAK	0.42286	0.436223	0.691467
FKBP11	0.053686	0.405205	0.271145
TMOD3	0.024706	0.202658	0.190022
BET1L	0.038035	0.53711	0.233324
RASL12	0.351465	-0.41664	0.636424
ELOVL5	0.354055	0.378443	0.638094
MIS18A	0.810389	-0.03156	0.921889
CELSR1	0.212057	-0.58724	0.507864
NKIRAS2	0.746288	-0.22259	0.889626
NKIRAS1	0.420028	-0.34122	0.690152
WSB2	0.993518	0.026772	1
PLEK2	0.111705	1.097315	0.382146
UGGT2	0.025281	1.04735	0.192573
UGGT1	0.015071	0.251071	0.150049
CDK12	0.011127	1.375943	0.128152
RRN3	0.453311	-0.41706	0.712914
FASTKD2	0.665709	0.250002	0.847351
TVP23B	0.147197	0.893729	0.436386
SLC25A37	0.638802	0.106703	0.834211
GTSE1	0.726238	-0.26961	0.878089
SIGLEC8	0.414362	0.317797	0.685923
TECR	0.400346	-0.06563	0.67622
ERAP1	0.255193	0.172376	0.557593
UBAP1	0.033373	0.718481	0.219585
PLA2G3	0.322009	0.097535	0.613139
ACTR10	0.887225	0.194683	0.96416
PSENEN	0.322009	0.19731	0.613139
USE1	0.482377	0.119935	0.733586
CISD1	0.052873	0.210902	0.269122
GGA3	0.604241	0.178224	0.813002
PODXL2	0.066032	0.671878	0.300455
C9orf78	0.161512	1.135253	0.453012
RTEL1	0.045973	0.417219	0.252361
CLIC5	0.00237	-2.51829	0.052884
FAM120A	0.72417	0.027847	0.876654
MOCS1	0.852939	-0.10132	0.946466
TREM2	0.273877	0.746762	0.577625
GDE1	0.550538	-0.05214	0.781327
EHF	0.739418	-0.24439	0.886294
WWOX	0.533391	0.626449	0.7709
SMARCAL1	0.044568	0.75851	0.24851
GLTP	0.965801	-0.07301	0.99616
AHSP	0.087408	-1.46725	0.338845
SPG21	0.031791	0.302478	0.214212
MRPL35	0.841813	-0.14982	0.940237
PLAC8	0.545521	-0.56922	0.778738
NINJ2	0.241572	0.847022	0.544139
GPRC5B	0.657812	0.295496	0.842898
IL37	0.937694	0.175847	0.986498
GRHL1	0.798715	-0.07279	0.916173
UBP1	0.017412	0.430483	0.159467
IGF2BP1	0.792343	0.140316	0.913596

EIF2AK3	0.220856	0.578027	0.519064
COQ3	0.770826	0.084577	0.903336
MTCH1	0.955289	-0.00974	0.993074
NUDT4	0.041861	0.68311	0.242066
CECR1	0.9081	-0.40759	0.9734
HSPBP1	0.093209	0.377589	0.347256
RGL1	0.123603	0.516769	0.399439
MAT2B	0.71923	-0.03733	0.874478
MYOF	0.002838	0.27384	0.060074
ITSN2	0.897655	-0.13849	0.969793
GLTSCR1	0.051109	1.326195	0.265139
GLTSCR2	0.353046	0.776252	0.638094
EHD3	0.419078	0.075159	0.688796
EHD2	0.528742	-0.15272	0.767576
ARHGEF12	0.71923	-0.05267	0.874478
CNOT2	0.041135	0.881597	0.240826
C1RL	0.964486	0.057745	0.99616
NCKIPSD	0.623137	0.061449	0.825978
CD274	0.41479	0.664881	0.686028
TMOD2	0.894964	0.223746	0.968678
CALML5	0.356774	0.858666	0.641472
OGFR	0.181644	0.167712	0.472575
FLRT3	0.468053	-0.3942	0.722799
LMCD1	0.131757	0.28292	0.413476
CRIM1	0.277593	0.333063	0.579677
SEPN1	0.464187	0.350422	0.721065
MPP6	0.871617	0.127395	0.956098
CHMP5	0.060571	0.390105	0.287134
COMMD9	0.161013	0.212497	0.453012
CNIH4	0.923804	-0.16704	0.980751
CWC15	0.654347	0.656997	0.842019
MRPL15	0.277896	0.408688	0.579677
THYN1	0.311298	-0.2327	0.609894
CRIPT	0.380513	-0.68294	0.661698
NDUFAF4	0.590866	0.230788	0.804875
HACD3	0.422885	0.143731	0.691467
MED11	0.947074	-0.15513	0.989967
CCDC167	0.076138	1.195767	0.321815
KLK14	0.980555	0.112746	1
EMC3	0.108241	0.323865	0.374055
NDUFA13	0.247021	0.246396	0.549922
PDP1	0.181644	0.249147	0.472575
KCMF1	0.887113	-0.01286	0.96416
ADAM22	0.99433	-0.09006	1
RAI14	0.000502	0.498365	0.020488
FOXJ2	0.123954	0.862264	0.400153
VAPA	0.799534	-0.03037	0.916173
AKAP7	0.796103	-0.20889	0.914603
H2AFY2	0.145824	0.561661	0.434185
MRPL27	0.509493	-0.31077	0.753337
TBC1D7	0.042259	1.144094	0.243142
RNF181	0.960185	0.092986	0.994122
C6orf203	0.507366	-0.54607	0.75153
GSKIP	0.213664	1.051339	0.509228
COX16	0.073618	1.399492	0.316471
ORMDL1	0.927689	0.082118	0.983115
TMEM14C	0.604241	0.965828	0.813002
TMEM9	0.19883	1.053595	0.49217

TOMM7	0.645537	-0.43266	0.837353
SENP1	0.287108	0.665255	0.590535
CXXC1	0.41358	0.183388	0.685243
SH3BP4	0.000248	1.242503	0.012326
SLAMF8	0.312629	0.432689	0.610776
SEPTIN10	0.145824	-0.31927	0.434185
HMG20B	0.137886	0.671983	0.423394
PIPOX	0.533465	0.165675	0.7709
GMIP	0.586319	0.332225	0.801141
NTM	0.000904	2.102834	0.028881
ABRACL	0.347246	0.207197	0.632606
VPS54	0.687798	0.215819	0.859243
MDFIC	0.017026	1.12479	0.157888
ZNRD1	0.363607	0.485826	0.646996
ACTR3B	0.080193	-0.91318	0.326714
TMEM63C	0.973327	-0.02685	0.998921
PIM2	0.322009	0.135232	0.613139
CAMSAP3	0.316413	-0.41723	0.613139
PHRF1	0.491562	0.740316	0.739769
ZBTB4	0.385624	-0.55617	0.664306
CALCOCO1	0.10353	1.053675	0.366863
KIAA1522	0.121449	-0.48603	0.397343
POGK	0.126056	1.055174	0.403148
CCDC88C	0.565699	-0.28388	0.79107
ARHGAP23	0.797226	-0.19911	0.915298
STIM2	0.231078	0.459411	0.531962
VPS18	0.077655	0.202663	0.324641
RCC2	0.45805	0.109774	0.715224
KIAA1468	0.200669	0.436517	0.494233
DIP2B	0.06524	0.245031	0.298136
KIAA1462	0.007608	1.061095	0.10525
SLAIN2	0.006002	1.192059	0.090802
TENM3	0.896819	-0.04468	0.969793
USP36	0.751732	0.24116	0.89314
BCCIP	0.960544	0.1292	0.994122
STK26	0.694713	-0.03395	0.861478
ARMCX1	0.148492	1.067938	0.438051
HIGD1B	1	-0.02956	1
COPZ2	1.74E-08	2.868948	6.25E-06
ABI3	0.863801	-0.32424	0.951898
PTGFRN	0.001527	0.85955	0.040048
CTTNBP2NL	0.135945	1.027375	0.419757
CFAP97	0.452169	-0.62474	0.712157
TMEM181	0.406729	-0.47834	0.680612
IBTK	0.240566	0.515941	0.543847
CHD7	0.304504	0.583849	0.606083
HEATR5B	0.434427	0.260679	0.698194
ZNFX1	0.01453	0.828258	0.147184
CHPF2	6.99E-05	1.804794	0.00473
RRBP1	0.206534	0.186516	0.500256
SIPA1L2	5.33E-06	2.512505	0.000591
ZNF319	0.756763	-0.06488	0.896333
ANKIB1	0.011592	1.073653	0.129699
IFT80	0.8035	-0.01787	0.918848
CPSF2	0.028771	0.355171	0.204946
KLHL9	0.33829	0.50013	0.625538
LARS	0.478274	0.031674	0.730754
TXNDC16	0.377688	0.494839	0.659304

RCOR3	0.056205	1.331348	0.276683
MYEF2	0.315145	-0.2385	0.613139
KLHL42	0.005219	1.180573	0.085005
EIF2AK4	0.108028	0.852126	0.374055
WDR35	0.400124	0.394647	0.67622
TBC1D14	0.438587	-0.22593	0.701933
CGN	0.694713	-0.3465	0.861478
ARHGAP28	0.167109	0.416084	0.457139
RBM27	0.088176	0.58805	0.339301
KANSL3	0.018048	1.134966	0.162744
NYNRIN	0.981892	-0.08478	1
HECW2	0.221922	-0.72027	0.520366
FRMD4A	0.4531	0.080135	0.712914
ANKFY1	0.179497	0.120486	0.471072
RERE	0.942956	0.01943	0.988547
SUCLA2	0.934288	-0.03932	0.984157
WRAP73	0.057896	1.25746	0.280499
GMPR2	0.434427	-0.20903	0.698194
PSMC3IP	0.657906	-0.15571	0.842898
STX18	0.908103	0.006519	0.9734
DPM3	0.809738	1.040733	0.921586
IMPACT	0.245568	0.403491	0.549555
ZNF219	0.031304	0.999696	0.212999
UVRAG	0.222159	0.38044	0.520592
HLA-DRB1	0.026002	-0.63454	0.193808
ATXN10	0.149515	0.416486	0.438811
MBD2	0.057903	1.04571	0.280499
NCDN	0.267786	0.455998	0.570195
TFIP11	0.382127	0.282553	0.661698
NFKBIL1	0.270581	0.376066	0.573699
EPS15L1	0.305036	0.161913	0.606083
SPRR3	0.310183	0.306316	0.609864
ORC3	0.201821	0.276861	0.495032
SAE1	0.317645	0.275074	0.613139
MAGEC2	0.988991	0.048467	1
COPG2	0.627809	-0.03188	0.828932
RNF7	0.876741	-0.15743	0.958859
PI4KB	0.035509	0.937336	0.225173
MRC2	8.05E-08	1.023186	2.13E-05
XPR1	0.001272	1.363205	0.034924
COMMD3	0.034511	0.256912	0.222148
STOML1	0.533043	-0.38812	0.7709
GNG12	0.162993	0.211161	0.453012
HECA	0.017913	1.428844	0.162312
MTRR	0.105233	1.241473	0.369374
UXT	0.221618	0.700268	0.519874
ASH2L	0.026447	0.59597	0.195939
CPNE7	0.571349	0.076797	0.793891
PEMT	0.399322	-0.68805	0.67622
DHCR7	0.78936	-0.10848	0.911483
CACNG4	0.988991	0.013302	1
HDAC6	0.882017	0.033202	0.961515
SPAST	0.216679	0.893543	0.513611
DKK3	0.042469	1.460002	0.243142
METTL1	0.91835	-0.20558	0.978657
GULP1	0.960544	0.252549	0.994122
VPS29	0.301936	0.092042	0.603275
EIF3K	0.013502	0.415048	0.141812

EXTL2	0.046544	1.403461	0.253921
GRHPR	0.81486	0.011733	0.924847
CTSZ	0.173168	0.176153	0.464476
CKLF	0.012696	1.444558	0.13791
RPS6KB2	0.171765	1.110566	0.463065
DNAJB9	0.347655	0.71702	0.632935
DNAJB11	0.000268	0.52743	0.01304
RNF14	0.167925	0.575369	0.458281
SUCO	0.069315	0.878138	0.30702
UBA2	0.577274	0.132582	0.796634
CTNNAL1	0.464493	-0.2987	0.721065
DKK2	0.009531	1.42535	0.118512
FAM8A1	0.568237	0.264802	0.791838
MORF4L1	0.568237	0.211478	0.791838
NXF1	0.051278	0.310308	0.265139
SEL1L	0.025563	0.254917	0.193268
B4GALT7	0.121079	0.893624	0.396833
PEF1	0.784286	0.071465	0.909506
BIN2	0.554951	-0.05053	0.783439
ZMYM2	0.485039	0.170416	0.735617
COPS7A	0.830256	-0.0255	0.933615
CTSF	0.859019	-0.13041	0.949415
SLC25A10	0.739057	-0.1076	0.886253
FBLN5	0.133456	-0.46836	0.416228
KLK11	0.810508	0.045945	0.921889
CLN8	0.154416	0.963957	0.444958
HSPB7	0.175042	0.776569	0.466796
PPP1R1B	0.503438	-0.57852	0.747774
AASS	0.12513	-0.38657	0.401334
CLIP2	0.825116	-0.0265	0.930395
UQCR10	0.162993	0.262358	0.453012
ZMAT5	0.163587	0.458713	0.453012
SEC14L4	0.203111	-0.54771	0.497506
MTFP1	0.453859	0.433174	0.712914
TJP2	0.159052	-0.32205	0.449897
DNAJB4	0.005764	1.105551	0.089283
MALT1	0.080575	0.72422	0.327829
STK17A	0.019439	1.280059	0.167517
CFDP1	0.004336	1.863683	0.075379
ZNF629	0.060283	1.156164	0.287134
DAXX	0.445875	0.293607	0.706795
FTSJ1	0.178168	-0.81902	0.469506
VTI1B	0.35064	0.181617	0.635657
MARCO	0.930697	0.115792	0.984157
STK39	0.249724	0.35061	0.552833
ADD3	0.902877	-0.05753	0.971583
PLEKHB1	0.481732	0.16551	0.733586
FBXL17	0.947123	-0.1495	0.989967
ZBTB47	0.217891	0.277048	0.515383
LRWD1	0.019032	0.713977	0.166597
CNOT8	0.080922	1.14547	0.328405
C19orf25	0.330591	-0.03883	0.619341
NIPSNAP3A	0.017724	1.011573	0.161012
CGGBP1	0.025123	0.705139	0.191949
IFT172	0.68188	0.091999	0.856967
GIMAP2	0.087761	1.145482	0.339301
PISD	0.874117	0.310201	0.957716
ABCF2	0.020065	0.457694	0.169555

MTRF1L	0.945776	0.129181	0.989025
SLC23A2	0.182882	0.545945	0.474175
TES	0.554955	-0.07081	0.783439
PRKAG2	0.667993	0.216578	0.849281
TUBGCP4	0.344551	0.377505	0.630804
STAP2	0.03305	1.284538	0.21845
SERGEF	0.003948	1.634892	0.071362
KDM5B	0.058278	0.937724	0.281667
DMBT1	0.091605	-1.40213	0.345626
FETUB	0.729616	0.498409	0.880062
WARS2	0.392996	0.086773	0.671013
CD300A	0.112485	1.043782	0.382523
PARP2	0.971687	0.173346	0.997592
LIMD1	0.061485	-0.37042	0.289565
POLL	0.696142	-0.16144	0.862864
SEC63	0.382127	0.100392	0.661698
CACFD1	0.472922	0.315473	0.727018
SLC2A6	0.427094	0.573193	0.694051
ZC3H7B	0.470127	0.193512	0.72456
SUSD2	0.108958	-0.30041	0.375995
TCF20	0.001385	1.721843	0.037197
HMGXB4	0.550442	0.359452	0.781327
NDRG3	0.073319	0.524394	0.31592
SEPTIN3	0.049205	1.323352	0.260606
APOBEC3B	0.033781	1.506861	0.221141
ARMCX3	0.486499	0.181619	0.73612
SWAP70	0.1068	0.178208	0.371671
EBF1	0.937694	0.17138	0.986498
MLX	0.574275	0.319145	0.795974
SUN2	0.343873	0.245803	0.630156
SS18L2	0.68818	0.245518	0.859429
RSL24D1	0.509733	0.363737	0.753592
LAMTOR3	0.981581	-0.03248	1
NDOR1	0.204944	0.464079	0.499348
LIMA1	0.007049	0.368752	0.100623
AFF4	0.5726	0.337973	0.794481
SRP68	0.43056	0.127049	0.695651
CNTNAP2	0.575621	0.188909	0.796634
MKRN1	0.251715	-0.6899	0.555643
CHORDC1	0.374984	0.288953	0.656862
TBK1	0.004244	0.449316	0.074174
SEPTIN9	0.153276	0.147416	0.443165
UBQLN2	0.070198	0.333282	0.309184
STEAP1	0.000849	2.54312	0.027766
EGFL7	0.355703	-0.71178	0.640269
TRPS1	0.650206	0.09187	0.839222
PCSK1N	0.889831	-0.1433	0.96614
PCYOX1	0.939534	-0.03039	0.986498
SLC7A8	0.087897	1.283465	0.339301
DDX20	0.668036	0.593054	0.849281
ADAMTS1	1	-0.01409	1
SHPK	0.424783	-0.00795	0.693599
PGAP2	0.247451	0.423418	0.550658
AMACR	0.879415	-0.09086	0.96104
DPP7	0.913333	0.060931	0.975706
POLG2	0.003221	1.915931	0.065092
TMEM2	0.32085	0.445182	0.613139
USP25	0.05293	0.871633	0.269285

RSPH14	0.980555	0.028235	1
NARF	0.009931	1.142908	0.120767
BCAP29	0.255147	-0.16533	0.557593
CYB5R1	0.554955	-0.09705	0.783439
BAIAP2L1	0.764071	-0.06686	0.899242
SAP30BP	0.133396	0.5775	0.416228
ZNHIT2	0.285467	0.736953	0.589814
MED13	0.124063	0.69778	0.400153
PFDN2	0.442223	0.064376	0.703704
GPN3	0.016912	1.605657	0.157224
SLC12A6	0.165222	1.004345	0.455049
PUF60	0.003297	0.506874	0.065524
EMR2	0.473115	0.508606	0.727018
NRBP1	0.012538	0.593534	0.136468
ENOPH1	0.764084	0.191499	0.899242
FEZ2	0.762717	0.270608	0.899242
EVL	1	-0.02372	1
NDUFA12	0.632496	0.04542	0.830965
EIF2B4	0.965801	-0.12099	0.99616
ATP6V1H	0.44615	0.074074	0.706795
RABAC1	0.010038	0.991809	0.12115
TAGLN3	0.037785	1.742853	0.233205
DMGDH	0.657397	0.089937	0.842898
IPO11	0.14113	0.802299	0.427981
TRMT112	0.78936	-0.15367	0.911483
GLS2	0.441773	-0.671	0.703704
DACH1	0.421971	0.320493	0.691467
FTSJ2	0.695601	0.248086	0.862317
DNAI1	0.027176	0.768331	0.199264
CYTH4	0.095845	1.057035	0.352341
XPO7	0.035078	0.38081	0.223337
CD84	0.117978	0.232606	0.392116
LCMT1	0.608408	-0.08489	0.816529
VPS51	0.490639	0.153969	0.738656
TEKT2	0.716634	-0.20178	0.873394
MUTYH	0.312494	0.136675	0.610732
BAZ2B	0.250602	-0.43831	0.554113
BAZ2A	0.387864	0.547811	0.666532
BAZ1B	0.694713	0.06772	0.861478
SLCO3A1	0.711134	0.140592	0.870136
ATPIF1	0.079337	-1.37525	0.32578
HERC5	0.096862	0.681151	0.354489
AK3	0.223612	-0.21169	0.521792
DAPK2	0.469887	-0.42935	0.72456
SCOC	0.020421	0.645142	0.170514
PHF11	0.293316	0.652174	0.596247
FKBPL	0.553176	0.232058	0.783439
LNPEP	0.000673	0.561548	0.024319
MBD1	0.771635	0.195844	0.903336
SIX4	0.385295	0.535412	0.664306
CNOT7	0.035068	0.111458	0.223337
SERPINB13	0.992139	0.00454	1
PLXNA1	0.000596	1.103364	0.022651
KCNG1		0	
GGT7	0.660099	-0.03113	0.844596
RABGEF1	0.000962	0.562804	0.02981
MSRA	0.076552	-0.37541	0.322705
NAGK	0.05791	0.334911	0.280499

CD207	0.332769	0.919902	0.62046
ANXA10	0.707926	0.353242	0.869179
HACL1	0.875933	-0.10041	0.958859
STAG3	0.375847	0.289865	0.657392
TRMT6	0.448077	-0.24224	0.708126
ENPP5	0.342453	0.539478	0.629771
HOOK1	0.082151	-0.34925	0.330671
SH3BGRL2	0.347233	-0.29197	0.632606
RIMS3	0.322009	0.128247	0.613139
RASAL2	0.007037	1.473971	0.100623
MOSPD1	0.296639	-0.43613	0.599275
METRN	0.051649	-1.06056	0.266309
RPUSD1	0.331588	-0.0998	0.619341
GNPTG	0.381753	0.691133	0.661698
TSR3	0.408552	0.325417	0.681493
ERRFI1	0.45638	0.356028	0.714931
HAO1	0.988991	0.079806	1
LAMP5	0.149822	0.711982	0.439247
SLC25A13	0.955289	-0.00656	0.993074
LEF1	0.079293	0.387414	0.32578
FOXD3	1	-0.03497	1
DBNL	0.094502	0.176435	0.349959
MID2	0.513881	-0.39094	0.756193
DDX41	0.694713	0.089275	0.861478
DCTN4	0.774166	-0.08083	0.903913
TINAG	0.440215	-0.15051	0.70314
CDC23	0.19721	0.536321	0.490309
ANAPC7	0.002	0.877794	0.047089
ANAPC5	0.11027	0.487588	0.378797
ANAPC4	0.001458	1.008045	0.038912
ANAPC2	0.009163	1.481436	0.116274
HSPB8	0.781585	0.084306	0.908746
GGA2	0.897645	0.332234	0.969793
GGA1	0.43056	-0.0584	0.695651
STOML2	0.175259	0.225424	0.466796
FBXO2	0.034089	-1.09465	0.221621
NAGPA	0.19661	0.81081	0.490309
ZNF580	0.181133	-0.33397	0.472575
CCRN4L	0.993518	-0.03456	1
VPS28	0.029749	0.217594	0.208169
LSM7	0.686515	-0.67838	0.858125
ING1	0.125859	0.779492	0.403148
SERPINA10	0.280679	0.619185	0.583434
CCNL1	0.456484	0.318853	0.714992
DBR1	0.228671	0.324688	0.528484
FAM208A	0.028056	0.824883	0.203111
FEM1B	0.988072	-0.01216	1
HN1	0.114834	1.411642	0.387168
FBXO9	0.178963	0.44174	0.471072
FBXO3	0.052048	0.652941	0.267367
FBXL4	0.392539	-0.37801	0.671013
AKAP11	0.078996	0.736125	0.32578
PTBP2	0.592726	0.305057	0.806337
FBXW11	0.010209	1.328088	0.121876
DNAJC12	0.502926	-0.52355	0.747596
GMEB2	0.071301	1.23596	0.311339
MRTO4	0.897655	-0.00101	0.969793
TNIK	0.755324	0.136299	0.895563

CPSF3	0.208914	0.212765	0.503054
PITPNC1	0.2536	0.736703	0.557407
APPL1	0.44615	-0.09894	0.706795
CROT	0.003883	1.505148	0.071121
CDC42EP3	0.957511	0.019437	0.994122
TLK1	0.512152	-0.67923	0.754954
PILRA	0.493121	-0.47867	0.741084
GPATCH8	0.09143	0.544013	0.345626
PARP4	0.231232	0.160013	0.531962
NXT1	0.035396	1.302631	0.224714
NUDT5	0.918567	0.094608	0.978657
RCOR1	0.389353	0.26129	0.667643
PCTP	0.903871	0.075336	0.972246
MAN1B1	0.130075	0.390624	0.410899
RALY	0.45805	0.109869	0.715224
GTF3C4	0.216173	0.239981	0.512741
ADAMTS7	0.460714	0.416601	0.718473
ADAM28	0.000798	1.974609	0.026879
KLK12	0.310183	0.376919	0.609864
C14orf1	0.40063	1.286363	0.676417
PACSIN3	0.043207	-0.29567	0.246126
IKZF2	0.211643	0.474743	0.507094
FBXO4	0.898878	0.28576	0.970641
FBXL3	0.083536	0.341265	0.333074
FBXW2	0.053305	1.097856	0.270697
IKZF3	0.980639	-0.00515	1
ACSL6	0.829403	0.044456	0.933615
ACAD8	0.015348	-0.77516	0.151713
ANGPTL2	0.000132	1.826994	0.007794
ACIN1	0.057044	0.297422	0.278658
AMFR	0.000513	1.215	0.020674
AGO2	0.266378	0.142747	0.568807
VAV3	0.045416	0.853394	0.250872
MYH2	0.287388	0.168792	0.590811
ITGA11	9.87E-11	4.302804	2.74E-07
NUP50	0.077655	0.451017	0.324641
PRND	0.429099	-0.37837	0.695651
ZHX1	0.439122	0.369825	0.702684
POMT2	0.085223	0.868939	0.335537
CDV3	0.145824	0.486997	0.434185
CNOT11	0.000229	1.305876	0.011713
PCOLCE2	0.339533	0.567976	0.626687
DSE	0.12807	0.673276	0.407582
INTS6	0.16099	0.307772	0.453012
SARDH	0.465247	0.333272	0.721065
BAG5	0.181644	0.252115	0.472575
CFAP45	0.928788	0.115549	0.983115
TBX21	1	-0.05612	1
AGO1	0.153275	0.351422	0.443165
RAB21	0.117214	0.175237	0.389929
RAB22A	0.374984	0.256882	0.656862
TRAPPC2L	0.013858	1.288119	0.144006
ZNF346	0.199864	0.694037	0.492817
PNMA3	0.319672	-0.24741	0.613139
PNMA2	0.085907	-0.21691	0.335964
BLOC1S6	0.079437	0.029037	0.325919
PSME2	0.442223	0.126896	0.703704
TMPRSS11E	0.834322	0.0745	0.935999

TAOK2	0.294061	0.248447	0.597112
MKLN1	0.360629	0.587523	0.644411
DNPEP	0.04253	-0.32348	0.243142
EMCN	0.124055	0.487769	0.400153
RAB23	3.20E-05	1.083542	0.00243
MCTS1	0.027356	0.424164	0.199874
ACSL5	0.097132	-0.38998	0.354489
PADI1	0.559081	0.091431	0.78633
ZDHHC8	0.22359	0.356607	0.521792
OGDHL	0.767772	0.015325	0.901915
MTUS1	0.665225	-0.11447	0.847351
BRPF3	0.480014	0.307475	0.732402
WWC3	0.013989	1.157792	0.144006
DENND2A	0.084914	-0.6166	0.335537
FAM184B	0.496663	0.441296	0.74366
PALD1	0.322021	0.601131	0.613139
SLC39A10	0.002023	1.960545	0.047528
INO80	0.592662	0.249924	0.806337
CCPG1	0.586195	1.015171	0.801141
KIDINS220	0.266378	0.118	0.568807
ASAP1	0.055344	0.596741	0.274516
MKL2	0.238156	0.732181	0.540624
ATAD2B	0.649217	0.266414	0.839222
HEG1	0.113954	0.726225	0.385201
ODF2L	0.420246	-0.24426	0.690386
ZBTB21	0.189865	0.477628	0.483202
ZMIZ1	0.02786	0.790984	0.202223
ANKRD50	0.536165	-0.32323	0.773175
PPP1R9A	0.039	-1.28843	0.236232
GRID1	0.094945	-0.49555	0.35136
MED23	0.007479	0.69053	0.10395
VANGL2	0.216541	0.86087	0.513504
PLEKHG1	0.049132	0.839263	0.260355
PLXNB3	0.940415	-0.11277	0.987142
PRR12	0.085768	0.809313	0.335537
SHROOM4	0.593687	0.276121	0.806337
YEATS2	0.039105	0.904829	0.236232
PNMAL2	0.867645	-0.02199	0.954126
NDRG4	0.540968	0.218229	0.775521
TBC1D24	0.075458	-0.96584	0.320055
STRIP2	0.993518	-0.13558	1
TPCN1	0.850863	-0.10457	0.944945
ISY1	0.061482	0.593001	0.289565
PPM1H	0.550501	0.437788	0.781327
PAIP2B	0.933487	0.152778	0.984157
TMCC3	0.569282	0.329882	0.792806
TTC7A	0.665692	0.041378	0.847351
HECTD1	0.008575	0.372711	0.111496
ZMYND8	0.563832	0.309946	0.789479
CADPS	0.902553	-0.0053	0.971583
MYO5B	0.337116	-0.62595	0.624371
FZD4	0.960709	0.135608	0.994122
CIZ1	0.134935	0.663174	0.417833
CORO1C	0.003027	0.402499	0.062294
CBLC	0.517846	0.052972	0.760014
TPX2	0.309175	0.553612	0.609864
ABT1	0.02979	1.534297	0.208193
PADI3	0.540968	0.17121	0.775521

NOB1	0.108191	0.974007	0.374055
AKAP8L	0.101186	0.340793	0.361901
MAFF	0.035298	1.345992	0.224243
CLEC4E	0.322009	0.115804	0.613139
PYCARD	0.257958	0.206015	0.560462
TMCO1	0.627809	-0.05706	0.828932
SLC7A7	0.017868	1.629328	0.162035
PADI4	0.370809	0.636029	0.65423
FZR1	0.10747	0.592994	0.372977
ANAPC10	0.961416	0.006171	0.994503
HPCAL4	0.399008	0.513639	0.67622
MGAT4A	0.028373	0.899556	0.20366
EPDR1	0.295754	-0.57853	0.598344
HHLA2	0.229605	0.451995	0.529537
NOTCH3	4.14E-06	1.534008	0.000478
MYO6	0.247021	-0.30032	0.549922
ALK	0.473815	0.329209	0.727018
COL17A1	0.405656	0.532728	0.679973
ICAM5	0.39165	-0.43403	0.670235
KMT2B	0.015904	0.82613	0.153751
DDX19B	0.004247	1.334498	0.074174
PPT2	0.375847	0.25589	0.657392
CLEC4A	7.23E-05	2.059799	0.00483
NFU1	0.887225	0.075331	0.96416
PRPF19	0.169043	0.130005	0.459676
SYNPO2	0.374958	0.481588	0.656862
USP18	0.993646	0.048665	1
UBQLN1	0.030249	0.409757	0.209418
SUFU	0.088907	0.794157	0.341082
BOK	0.340995	-0.34232	0.628026
NENF	0.298858	0.308099	0.601676
NOL7	0.683323	0.998041	0.85709
SNX12	0.438315	-0.00715	0.701933
SYNRG	0.520009	0.504363	0.760272
DAPP1	0.079329	0.899942	0.32578
NDRG2	0.761569	-0.00179	0.898761
VPS4A	0.194929	0.114446	0.488911
PCDHB8	0.022585	0.769993	0.180886
PCDHGC3	0.798715	0.049262	0.916173
SLC6A14	0.190925	-1.15496	0.484342
SOX13	0.31899	0.19542	0.613139
L1RE1	0.233222	1.062902	0.535403
G3BP2	0.083367	0.239106	0.332962
ADAMTS5	0.088427	0.815184	0.339985
ARHGAP26	0.002058	-1.12027	0.048148
POLI	0.285292	-0.29281	0.589561
RPH3AL	0.019765	-1.19617	0.1684
STUB1	0.098469	-0.24012	0.357719
PACSIN2	0.068179	-0.23756	0.303928
MAGED2	0.131757	0.2592	0.413476
SNX7	0.014839	1.723854	0.149757
SNX6	0.073319	0.217959	0.31592
DUSP12	0.083058	1.225491	0.332962
STX8	0.04253	0.339417	0.243142
PLA2G2D	0.496019	0.374969	0.743024
ANGEL1	0.346723	0.294172	0.632606
SSR3	0.466082	0.209083	0.721065
ING4	0.807146	-0.1525	0.920997

PSMD13	0.09581	0.233517	0.352331
FAF1	0.507378	0.67555	0.75153
PROCR	0.632477	0.308812	0.830965
ST3GAL5	0.790941	-0.21426	0.912928
PPIE	0.929044	0.051853	0.983115
ABCG2	0.807541	0.152042	0.921159
DIMT1	0.355724	0.703074	0.640269
TIMELESS	0.01917	0.74511	0.166914
COPS3	0.12513	0.147718	0.401334
RABL2B	0.179341	0.812065	0.471072
MINPP1	0.097022	0.563758	0.354489
NOVA2	0.668274	0.287792	0.849395
RPL26L1	0.834232	0.002678	0.935999
WDR3	0.269226	0.253523	0.571371
TTF2	0.083101	0.946658	0.332962
NSFL1C	0.44615	0.179805	0.706795
C19orf53	0.200849	1.024988	0.494233
FZD1	0.012959	1.318796	0.139171
PLA2G4C	0.130772	0.813223	0.412748
COG5	0.241676	0.182775	0.544139
SLC12A4	0.03232	0.526804	0.215938
SRPK3	0.992139	-0.02845	1
FLVCR2	0.041397	0.533207	0.240826
AP4E1	0.137385	0.821616	0.422524
MACF1	0.632496	0.139698	0.830965
CEP131	0.085603	0.973936	0.335537
SCAF8	0.374768	0.759941	0.656862
PPP6R1	0.021148	0.338158	0.173882
TRIM33	0.03339	0.907165	0.219585
PHF8	0.59287	0.273806	0.806337
KIAA1107	0.092612	-0.40663	0.347013
LIMCH1	0.121915	-0.33815	0.397343
AGAP1	0.675967	-0.17486	0.853501
PDZRN3	0.133624	0.54816	0.416228
DOLK	0.003629	1.795312	0.069037
TNRC6B	0.986123	-0.04605	1
PLCL2	0.003864	1.710455	0.071121
SMG5	0.380902	0.615122	0.661698
EXOC7	0.426712	0.131391	0.693599
MAPK8IP3	0.319836	-0.63079	0.613139
ZC3H4	0.759058	0.334844	0.896847
USP22	0.323436	0.523748	0.614309
USP24	0.462056	0.268292	0.718747
TBC1D2B	2.26E-05	0.850126	0.001867
SAMD4A	0.019997	1.284338	0.169555
CEP164	0.670751	0.099609	0.850681
KIAA1045	0.988991	0.01826	1
FOXJ3	0.005589	1.798149	0.088298
AGTPBP1	0.197193	-0.56588	0.490309
SHANK2	0.762934	-0.09221	0.899242
DICER1	0.047431	0.817486	0.255923
SLC7A11	0.683128	0.212847	0.85709
WASF3	0.661627	0.247728	0.84482
MAPRE3	0.366046	-0.28628	0.648976
HPS5	0.055291	0.836144	0.274516
THSD7A	0.405498	-0.08557	0.679973
CORO2B	0.766003	-0.21191	0.901309
SHOC2	0.162	0.218766	0.453012

DNM3	0.809394	-0.38111	0.921586
SRRM2	0.028293	0.446659	0.203235
NEU3	0.430203	0.178793	0.695651
MGAT4B	0.165529	0.838612	0.455554
PA2G4	0.871617	-0.00894	0.956098
CDK11A	0.102567	0.381527	0.364372
SPG7	0.045294	0.512864	0.250872
CTNND2	0.957511	-0.15679	0.994122
BAIAP2	0.734083	0.012157	0.882858
GAB2	0.179535	-0.43828	0.471072
CLCA2	0.153189	-0.48361	0.443165
SMC3	0.694713	0.030932	0.861478
HDAC5	0.34526	0.115365	0.631895
CHMP2B	0.235002	1.163441	0.537072
TNN	0.429962	0.437906	0.695651
SCML2	0.148117	0.351303	0.437487
ZNF148	0.000176	1.955659	0.009596
LAMP3	0.015314	-1.55201	0.151713
TRPC6	0.247903	-0.4023	0.551221
MTMR6	1.89E-05	0.833674	0.001637
NIP7	0.053272	0.737422	0.270654
GNE	0.918559	0.16769	0.978657
C14orf166	0.000876	0.322946	0.028311
ENTPD4	0.045616	1.387948	0.251457
TRAF3IP3	0.651372	-0.74022	0.839222
RUVBL2	0.049723	0.226036	0.261364
CDYL	0.038447	1.172652	0.235202
LIPT1	0.937694	0.061581	0.986498
PIN4	0.819974	0.033998	0.92687
CLEC11A	0.000748	1.917335	0.025761
HIGD1A	0.595262	0.18708	0.806337
AKT3	0.000577	1.943443	0.022453
POMP	0.457206	0.39567	0.715224
FAM50B	0.085979	0.541516	0.336126
GINS2	0.029349	1.293096	0.207066
LZTS1	0.001887	1.761734	0.045986
HPSE	0.993964	0.029662	1
RCE1	0.530116	-0.73668	0.768664
CCL26	0.331588	-0.14231	0.619341
CHKB	0.153213	-0.40905	0.443165
FOXA2	0.055017	-1.29022	0.274516
EIF3L	0.066208	0.166356	0.300455
PLAA	0.039276	0.302024	0.236232
RUVBL1	0.001243	0.305229	0.034693
NUDC	0.554955	0.106431	0.783439
TNFSF13B	0.11247	0.525951	0.382523
BCS1L	0.680152	0.105103	0.85545
VDAC3	0.378546	0.210085	0.659304
VSIG4	0.872945	-0.06156	0.956872
CFL2	0.183806	-0.54149	0.474829
ERGIC3	0.11117	0.351435	0.380668
FARSA	0.744041	0.051631	0.888303
SIGLEC7	0.136065	0.655007	0.419892
ITM2B	0.394825	0.230149	0.672349
SLC5A6	0.111383	1.118007	0.381279
MRPS33	0.791059	0.631671	0.91297
ASF1A	0.111125	1.140207	0.380668
DRG1	0.97106	0.121907	0.997592

TRAPPC4	0.524435	0.062007	0.764023
NCKAP1	0.581788	0.038375	0.797998
B3GNT3	0.055065	1.292107	0.274516
CNPY2	0.537409	0.07058	0.773175
PIGL	0.057403	0.796693	0.279974
VPS9D1	0.974304	-0.04392	0.998921
PKIG	0.642368	0.241501	0.835817
EXOG	0.003047	1.553568	0.062575
CA5B	0.472864	0.765817	0.727018
SLC35A3	0.233812	0.368383	0.535403
EXOC6B	0.568296	0.15823	0.791838
AKAP2	0.646644	-0.00259	0.837353
ZNF652	0.933998	-0.11426	0.984157
DIP2C	0.092406	0.825686	0.347013
MAN2B2	0.144731	0.49655	0.433371
SLC9A8	0.922878	0.106065	0.980751
ICE1	0.016622	0.649487	0.156319
EFR3B	0.328	0.268228	0.618682
CARD8	0.08167	1.073988	0.330094
ATP11B	0.034509	0.791052	0.222148
POFUT2	0.400341	0.412352	0.67622
DNAJC16	0.453791	0.062829	0.712914
SBNO2	0.31652	0.160889	0.613139
DLGAP4	0.035078	0.774373	0.223337
STK38L	0.069091	0.880445	0.306571
INPP5F	0.57577	0.226523	0.796634
PLEKHA6	0.902522	0.019918	0.971583
FNDC3A	0.046008	0.356301	0.252361
NISCH	0.365955	-0.25758	0.648976
NTNG1	0.94171	0.083586	0.988127
PIKFYVE	0.00505	1.026552	0.083219
WDR37	0.371444	-0.25991	0.65423
TBC1D30	0.918445	-0.12101	0.978657
EPB41L3	0.360948	0.03829	0.644411
AMOTL2	0.083601	0.86318	0.333173
PADI2	0.252443	0.16785	0.555643
ZBTB1	0.181771	0.559568	0.47269
SIK3	0.054817	0.835645	0.274162
R3HDM2	0.468001	0.171197	0.722799
USP20	0.07481	0.845629	0.318513
KDM2A	0.876808	0.110141	0.958859
DIS3	0.013012	0.6942	0.139171
TRAPPC8	0.14042	0.249115	0.426568
LRCH1	0.000723	1.045928	0.025365
SSUH2	0.319672	-0.33998	0.613139
SLC27A6	0.696781	-0.00193	0.863213
RCL1	0.25656	-0.06589	0.560028
ATP8A1	6.68E-05	-2.01699	0.004599
GSTK1	0.520146	0.039659	0.760272
LAMTOR2	0.298858	-0.16627	0.601676
MRPS28	0.759022	0.273623	0.896847
COA3	0.236413	-0.21877	0.537858
DDX52	0.174016	0.417522	0.466074
MRPS17	0.59083	-0.3677	0.804875
MRPS7	0.089414	0.166005	0.341477
CRYL1	0.415291	0.073668	0.686028
TMA7	0.837756	0.255487	0.938161
POLDIP2	0.404052	0.121415	0.678271

AP3M1	0.006009	0.353428	0.090802
GDA	0.484075	0.29931	0.735071
YBX2	0.751648	0.058438	0.893136
MAP3K2	0.099288	0.638979	0.358854
LEMD3	0.32085	0.133075	0.613139
CARHSP1	0.311298	0.228506	0.609894
COG6	0.563832	0.044265	0.789479
THRAP3	0.239034	0.221502	0.541047
WBP11	0.14042	0.420288	0.426568
TDRKH	0.590866	0.245464	0.804875
MED16	0.152643	0.770911	0.443165
NOP58	0.709387	0.057256	0.869179
GIT1	0.194929	0.198248	0.488911
UBE2D4	0.161733	0.503507	0.453012
ZNF281	9.75E-05	2.208876	0.006217
ARL2BP	0.022643	1.874348	0.180886
POLR3K	0.57907	-0.13793	0.796675
PRG3	0.744045	0.314415	0.888303
KLF13	0.207523	0.656402	0.502212
SUGT1	0.098469	0.295632	0.357719
MTO1	0.037838	1.200775	0.233324
YARS2	0.083367	0.289113	0.332962
COQ6	0.955288	-0.35756	0.993074
AMDHD2	0.535235	0.163129	0.772552
ACOT9	0.167008	0.140922	0.457139
AAR2	0.001956	0.657425	0.04685
NOSIP	0.001708	1.012185	0.043008
DERA	0.327323	0.157464	0.617719
MEMO1	0.035078	0.322347	0.223337
TMX2	0.646641	-0.22363	0.837353
FCF1	1	0.055932	1
LSM2	0.466082	0.07094	0.721065
SIGLEC9	0.150297	0.959308	0.440175
KLK5	0.322009	0.145162	0.613139
PLLP	0.055647	-1.21361	0.275603
SNX24	0.060289	1.031008	0.287134
STARD10	0.043529	-0.62907	0.247494
IFT52	0.406996	0.443536	0.680612
SH3GLB1	0.019371	0.228933	0.167192
NDUFAF1	0.442968	0.275593	0.704366
CAB39	0.228671	0.184992	0.528484
LUC7L2	0.577274	0.079418	0.796634
UBE2J1	0.446106	0.479727	0.706795
RBMX2	0.810487	-0.15929	0.921889
DHRS7	0.604602	-0.03056	0.813002
MRPS2	0.494787	-0.32935	0.741997
COQ4	0.526467	-0.30002	0.766077
UTP11L	0.885338	-0.14583	0.96416
MOB4	0.18381	0.211174	0.474829
RRP7A	0.379751	0.55951	0.661197
SBDS	0.637197	-0.15891	0.833511
TMED5	0.646644	0.121726	0.837353
EXOSC1	0.507055	-0.41357	0.75153
TMED7	0.007779	0.262734	0.105988
SF3B6	0.546147	0.155777	0.778738
EMC9	0.76149	0.499071	0.898761
MRPL11	0.845718	-0.00686	0.942239
REXO2	0.03232	0.642461	0.215938

RRP15	0.267194	0.958011	0.570195
CCDC53	0.162989	0.399551	0.453012
NOP16	0.627279	-0.36184	0.828932
TPRKB	0.373199	0.478138	0.656382
RNF11	0.244817	0.896698	0.548206
PPIL1	0.277896	0.136401	0.579677
MED31	0.02515	1.650516	0.191949
UFC1	0.960544	-0.04179	0.994122
FAM96B	0.204174	0.229116	0.497963
MSRB2	0.554941	0.058413	0.783439
MRPS16	0.048208	0.544646	0.257798
MRPS18C	0.181386	1.245408	0.472575
FIS1	0.97106	-0.00121	0.997592
PAM16	0.819984	-0.18123	0.92687
AK6	0.312593	0.694391	0.610776
MRPS23	0.324076	0.1431	0.614309
GOLT1B	0.003662	1.191914	0.06938
HDGFRP3	0.454064	0.426675	0.712914
BOLA1	0.029703	1.700302	0.208169
PTRH2	0.347246	0.130811	0.632606
CHMP3	0.702028	-0.40846	0.866168
STRAP	0.112657	0.238315	0.382523
RTCB	0.000897	0.225242	0.028766
FBXO7	0.093209	0.213504	0.347256
SH3BP1	0.106641	0.554951	0.371671
RAP2C	0.314456	0.627897	0.612305
CBY1	0.563678	-0.2148	0.789479
STARD13	0.037979	0.878932	0.233324
RHBDD3	0.070222	1.235436	0.309184
SIT1	0.050503	0.989007	0.263221
RABGAP1	0.161013	0.269055	0.453012
NAALAD2	0.341849	-0.43969	0.629182
TMED3	0.002963	0.693045	0.062294
TSC22D4	0.541731	0.422677	0.775521
DOPEY2	0.269226	0.338583	0.571371
ZNF330	0.399927	0.692367	0.67622
R3HCC1	0.026542	1.219631	0.196381
NOC2L	0.850885	0.249708	0.944945
RPL36	0.595428	0.106195	0.806337
CCDC9	0.038337	0.709343	0.234656
CHTOP	0.997369	-0.03414	1
SAMHD1	0.007779	0.322329	0.105988
FAM32A	0.473815	0.385142	0.727018
C2CD2	0.774093	-0.02617	0.903913
PKP3	0.430555	0.065089	0.695651
KNSTRN	0.672064	0.17963	0.851286
HBS1L	0.014268	0.500805	0.145444
SALL2	0.923017	-0.03429	0.980751
PRKAB1	0.581788	0.041783	0.797998
MTF2	0.974393	0.015779	0.998921
WDR45	0.582909	0.423264	0.799042
DMXL1	0.095123	0.627608	0.35136
ATP6V0A2	0.024495	0.486708	0.189451
TLN1	0.044589	0.152947	0.24851
KIF3A	0.180046	0.640589	0.472077
JRKL	0.040567	0.660784	0.240216
TRRAP	0.083367	0.428085	0.332962
RAD54L2	0.407428	-0.29934	0.680612

MTCL1	0.07345	0.587341	0.316346
VPRBP	0.490635	0.098527	0.738656
KDM3A	0.195954	0.594104	0.490309
TCAF1	0.074227	0.676042	0.317894
CHST2	0.115283	0.503385	0.387731
RBM19	0.351444	0.444094	0.636424
DAAM1	0.432479	0.323415	0.697404
PLXND1	0.175247	0.480494	0.466796
HECTD4	0.981553	0.256602	1
FAM21C	0.004707	0.504463	0.079703
ZNF451	0.013882	1.302736	0.144006
WDR7	0.019715	0.332754	0.1684
USP15	0.177368	0.303556	0.468602
FARP1	0.199513	0.180674	0.49217
KIAA0430	0.015033	1.084107	0.150049
CEP170B	0.160465	0.803108	0.453012
FAM65B	0.622203	0.043416	0.825978
PLEKHM1	0.12241	0.461642	0.398323
TLN2	0.001187	0.755294	0.03367
RAPGEF2	0.439669	-0.61551	0.70314
IRS2	0.171326	0.711747	0.462219
GPSM3	0.559364	-0.3984	0.78633
MYO5A	0.003093	0.535773	0.063058
DTNA	0.030755	-0.43556	0.211475
LOXL2	6.57E-05	2.832314	0.004554
AIM1	0.590866	0.066032	0.804875
TRAF6	0.292737	0.680606	0.595788
MAP4K5	0.000162	1.553409	0.009119
HYOU1	0.055344	0.221351	0.274516
RNF115	0.175699	0.750446	0.467197
ATG4B	0.44221	0.215586	0.703704
TBL2	0.407778	0.124107	0.680612
WIPI2	0.684947	0.223149	0.85709
TELO2	0.006344	1.490652	0.094329
MMACHC	0.995277	-0.03718	1
LAS1L	0.009626	0.517279	0.118893
AFG3L2	0.809743	0.017713	0.921586
AMMECR1	0.986749	-0.03144	1
ARIH1	0.026898	0.650743	0.198225
LSM5	0.055343	1.068629	0.274516
LSM4	0.192662	-0.45876	0.486094
RNF114	0.091926	0.540586	0.345626
SAMM50	0.559385	0.103936	0.78633
PRRC2C	0.00719	0.755135	0.101457
OARD1	0.547275	-0.20529	0.779573
POLR3H	0.465993	0.590673	0.721065
LRRC42	0.24809	0.675923	0.551415
HSPB11	0.665519	0.593547	0.847351
YIPF1	0.992006	0.17658	1
PPME1	0.089414	0.479976	0.341477
RIPK3	0.064259	0.967934	0.296208
ASB3	0.24354	0.358365	0.547425
ASB1	0.245238	-0.28009	0.548928
RBM7	0.37652	-0.75196	0.658199
TIMM22	0.225417	1.293772	0.52468
AP4S1	0.040543	0.952867	0.240216
CEP83	0.594139	-0.22186	0.806337
KCTD3	0.005738	1.10356	0.089283

NUB1	0.470127	0.079155	0.72456
YTHDF2	0.144006	0.291443	0.431666
CTDP1	0.459489	-0.12201	0.716966
PAXBP1	0.252448	0.311576	0.555643
NME7	0.001828	1.145587	0.044883
SUPT16H	0.074384	0.303673	0.317894
PCDHB2	0.384669	0.274547	0.66413
PCDHGB7	0.079293	0.308786	0.32578
PCDHGB2	0.229605	0.583779	0.529537
PCDHGA2	0.331588	-0.10306	0.619341
PCDHA3	0.398667	0.302783	0.676088
PCDHAC2	0.26324	-0.29791	0.565873
UTP18	0.204174	0.333541	0.497963
PHLDA3	0.218277	0.886236	0.515816
TIMM10B	0.176211	-0.07837	0.468218
TIMM9	0.581788	0.039945	0.797998
TIMM8B	0.845683	0.022275	0.942239
PCYT1B	0.690406	0.316784	0.85979
UCHL5	0.255193	0.189555	0.557593
CD2AP	0.646644	0.144445	0.837353
ATP6V1D	0.71923	-0.2385	0.874478
TNPO3	0.078771	0.317855	0.32578
HILPDA	0.322009	0.136722	0.613139
ENTPD2	0.49063	-0.19981	0.738656
TIMM13	0.577274	-0.15611	0.796634
SRPRB	0.023873	0.319982	0.186463
N6AMT1	0.451193	0.521989	0.711024
COL4A3BP	0.908103	-0.00631	0.9734
GMPPB	0.739057	-0.00218	0.886253
FADS3	0.739884	-0.14977	0.886374
MAFB	0.210922	0.704988	0.506632
GTF3C5	0.04934	0.553325	0.260958
GTF3C3	0.003027	0.729821	0.062294
HEMK1	0.949005	0.066125	0.990863
DMRT2	0.008326	1.192205	0.109932
DMRT1	0.322009	0.142371	0.613139
TRAPPC1	0.003899	1.262931	0.071121
TRPV2	0.192667	0.213342	0.486094
CDC42BPB	0.007479	0.342679	0.10395
RBM8A	0.568296	0.124587	0.791838
DNAJC15	0.133765	0.477343	0.416228
USP16	0.298784	0.592036	0.601676
TSSC4	0.53908	0.690057	0.774975
TNFRSF18	0.039604	0.839575	0.237431
MPC1	0.653736	0.040901	0.841555
IER3IP1	0.064284	0.621982	0.296208
ZNF706	0.048893	1.784813	0.259694
MAGED1	0.007538	1.618879	0.104632
WIF1	0.175422	-0.55645	0.466796
SNX14	0.554066	0.009471	0.783439
SNX13	0.918415	-0.1108	0.978657
SNX11	0.938369	0.013806	0.986498
SNX9	1.07E-06	0.696277	0.000157
SNX8	0.81486	0.097617	0.924847
SNX5	0.779221	0.02664	0.906856
LIPG	0.455572	0.20105	0.71417
FLVCR1	0.99047	0.008238	1
NUBP2	0.298858	0.199505	0.601676

PEX16	0.320366	0.738489	0.613139
ST14	0.535225	-0.37673	0.772552
LYVE1	0.888841	-0.02254	0.965603
BACE2	0.595981	0.277815	0.806888
HEBP2	0.466082	-0.14537	0.721065
HCFC2	0.035765	0.980711	0.225894
UBIAD1	0.185033	-1.08449	0.477255
CSAD	0.169846	-0.44855	0.461029
MRFAP1	0.194427	1.22027	0.488911
PUS1	0.354055	0.248998	0.638094
LRRFIP2	0.231232	-0.22904	0.531962
FHOD1	0.660922	-0.02956	0.844596
IRAK3	0.451167	0.459631	0.711024
PSAT1	0.052057	0.467678	0.267367
NCOR2	7.46E-05	1.309521	0.004958
SLC25A15	0.356681	0.958967	0.641472
F11R	0.147661	-0.3029	0.436386
GPC6	2.01E-06	1.835928	0.00026
NPTN	0.072266	0.254592	0.313701
RFNG	0.870327	-0.08675	0.956098
CPQ	0.3985	0.282405	0.676012
GPR56	0.005438	1.784832	0.086993
SPIN1	0.017954	1.431325	0.162555
KPTN	0.259612	0.685652	0.563057
SLC12A7	0.680152	0.202067	0.85545
ALG6	0.342193	0.336536	0.629606
ALG5	0.71923	0.107311	0.874478
MRPS18B	0.378519	0.018828	0.659304
COPG1	0.053686	0.227529	0.271145
AUP1	0.079333	0.486888	0.32578
FKBP7	2.85E-05	1.122784	0.00221
ARL5A	0.257625	0.824323	0.560462
GMEB1	0.532637	0.416418	0.7709
LHFP	0.430232	-0.35177	0.695651
CLIC4	6.32E-05	0.61939	0.004408
NFS1	0.908103	0.030031	0.9734
POMT1	0.122179	0.833719	0.397854
CFAP20	0.011631	0.737506	0.129699
TACC3	0.960097	0.188823	0.994122
SPCS1	0.233812	0.287563	0.535403
SAR1B	0.046732	0.301951	0.254194
AP4B1	0.23586	0.625372	0.537858
EMILIN1	0.00171	0.390191	0.043008
MTCH2	0.845718	0.021314	0.942239
SELK	0.144585	-1.09996	0.433053
ARFGEF2	0.199513	0.107707	0.49217
ARFGEF1	0.324076	0.144707	0.614309
MAD1L1	0.021897	0.424787	0.176756
STK24	0.97106	0.020977	0.997592
BZW2	0.228671	0.424	0.528484
MRVI1	0.00069	1.996871	0.024536
TMEM14A	0.322009	0.151524	0.613139
MRPL42	0.050265	1.555475	0.263221
COMMD10	0.668107	-0.05254	0.849281
DYNC1LI1	0.067188	0.18635	0.302058
CHCHD2;CHCHD2P9	0.314461	0.061569	0.612305
XRCC6BP1	0.451681	-0.34549	0.711655
EPN1	0.051278	0.270435	0.265139

USP3	0.032487	1.275421	0.216666
PXMP4	0.380902	-0.24537	0.661698
TEX264	0.486497	0.456175	0.73612
CABIN1	0.174622	-0.60357	0.466796
TAF6L	0.246735	0.305366	0.549922
CEPT1	0.187853	1.007807	0.480067
DNMT3A	0.003626	1.762239	0.069037
OAS3	0.419078	0.187583	0.688796
IKBKG	0.550543	0.001667	0.781327
IGF2BP2	0.109581	1.272107	0.377423
CSNK1G3	0.194782	0.784836	0.488911
SLC30A1	0.213733	0.301602	0.509228
SLC4A7	0.009253	0.874122	0.116462
NDUFB9	0.950035	0.002465	0.990863
COX11	0.866222	0.040738	0.953042
SQRDL	0.835403	-0.11274	0.935999
LAMC3	0.11228	-0.93133	0.382523
ROBO1	0.010119	1.267392	0.12173
USH1C	0.995826	-0.02851	1
SESN1	0.77733	-0.05	0.906372
CAPN6	0.767026	0.026544	0.901915
STON1	0.000639	1.887689	0.023555
AP1M2	0.595424	0.080158	0.806337
NCOA3	0.1891	0.58134	0.481831
NUMBL	0.403768	0.905146	0.678271
SLC4A4	0.943096	-0.29098	0.988547
MAP3K4	0.041308	1.141851	0.240826
FCGBP	0.254903	0.69517	0.557593
CCDC61	0.064718	-0.78078	0.297421
SCIN	0.403897	0.879697	0.678271
DDX49	0.663171	-0.57252	0.846498
CAPN7	0.104509	0.654106	0.368322
WASF2	0.861239	0.055897	0.950165
PIAS3	0.060265	0.925674	0.287134
MAU2	0.131524	0.54542	0.413476
FAM169A	0.461959	0.175791	0.718747
ENPP4	0.774166	-0.10887	0.903913
ZHX2	0.794829	0.00715	0.913708
MORC2	0.627036	0.261988	0.828932
IVNS1ABP	0.581411	0.57015	0.797998
SEC23IP	0.78936	0.014393	0.911483
LY96	0.843352	0.049204	0.941517
